# Demography and life histories across the Roman frontier in Germany 400–700 ce

**DOI:** 10.1038/s41586-026-10437-3

**Published:** 2026-04-29

**Authors:** Jens Blöcher, Leonardo Vallini, Maren Velte, Raphael Eckel, Léa Guyon, Laura Winkelbach, Mark G. Thomas, Nadia Gharehbaghi, Cassandra T. Mitchell, Jonas Schümann, Sophie Köhler, Elsa Seyr, Katharina Krichel, Sophie Rau, Jana Hirsch, Jana Duras, Paul Cloarec-Pioffet, Andreas Füglistaler, Kristin Klement, Miriam Wilkenhöner, Lisa Vetterdietz, Francesca Gentilin, Melany Müller, Anna-Lena Mücke, Nicoletta Zedda, Youssef Tawfik, Eveline Saal, George McGlynn, Barbara Bramanti, Jörg Orschiedt, Regina Molitor, Barbara Fliß, Ines Spazier, David Shankland, Claus Vetterling, Kurt Karpf, Vera Planert, Stefan Hölzl, Silvia Codreanu-Windauer, Dieter Quast, Ilija Mikić, Sven Fiedler, Bernd Päffgen, Maxime Brami, Thomas Richter, Raphaëlle Chaix, Susanne Brather-Walter, Peter Steffens, Markus Marquart, Thomas Becker, Jochen Haberstroh, Mischa Meier, Sebastian Schmidt-Hofner, Sebastian Brather, Michaela Harbeck, Steffen Patzold, Daniel Wegmann, Joachim Burger

**Affiliations:** 1 Palaeogenetics Group, Institute of Organismic and Molecular Evolution (iomE), Johannes Gutenberg University Mainz, Mainz, Germany; 2 SNSB, Bavarian State Collection of Anthropology, Munich, Germany; 3 Department of Biology, University of Fribourg, Fribourg, Switzerland; 4 Swiss Institute of Bioinformatics, Fribourg, Switzerland; 5 Institute of Evolutionary Biology, CSIC–Pompeu Fabra University, Barcelona, Spain; 6 Eco-Anthropologie (UMR 7206), Muséum National d’Histoire Naturelle, CNRS, Université Paris Cité, Paris, France; 7 Department of Genetics, Evolution and Environment, University College London, London, UK; 8 Department of Environmental and Prevention Sciences, University of Ferrara, Ferrara, Italy; 9 Hessian State Office for Monuments and Sites, hessenArchäologie, Marburg, Germany; 10 State Office for Heritage Preservation and Archaeology Saxony-Anhalt, Halle, Germany; 11 Leibniz-Zentrum für Archäologie (LEIZA), Mainz, Germany; 12 Institute of Legal Medicine, University Medical Center of the Johannes Gutenberg University Mainz, Mainz, Germany; 13 Thüringer Landesamt für Denkmalpflege und Archäologie, Weimar, Germany; 14 Royal Anthropological Institute, London, UK; 15 ReVe Büro für Archäologie GbR, Bamberg, Germany; 16 Stadt Villach–Museum und Archiv, Villach, Austria; 17 Department of Early Historical Archaeology and Medieval Archaeology, Albert-Ludwigs-Universität Freiburg, Freiburg, Germany; 18 SNSB, RiesCraterMuseum Nördlingen, Nördlingen, Germany; 19 Bavarian State Office for the Conservation of Historical Monuments and Sites, Munich, Germany; 20 Institute of Archaeology, National Institute of the Republic of Serbia, Belgrade, Serbia; 21 Landratsamt Deggendorf, Kreisarchäologie, Deggendorf, Germany; 22 Institut für Vor- und Frühgeschichtliche Archäologie und Provinzialrömische Archäologie, Ludwig-Maximilians-Universität München, Munich, Germany; 23 Pre- and Early Historical Archaeology, Institute of Classical Studies, Johannes Gutenberg University Mainz, Mainz, Germany; 24 Landratsamt Landshut, Kreisarchäologie, Essenbach, Germany; 25 Hessian State Office for Monuments and Sites, hessenArchäologie, Darmstadt, Germany; 26 Museen der Stadt Aschaffenburg, Aschaffenburg, Germany; 27 Institute of Ancient History, Eberhard Karls University Tübingen, Tübingen, Germany; 28 Institute of Medieval History, Eberhard Karls University Tübingen, Tübingen, Germany

**Keywords:** Archaeology, Genomics, Genetic variation, History

## Abstract

The emergence of new political and social structures in Western and Central Europe during the transition from Antiquity to the Middle Ages has long been attributed to large-scale migrations. Yet emerging evidence increasingly emphasizes the role of small-group mobility in reshaping the Roman world^1–3^. Here we present 258 ancient genomes from the former Roman frontier of southern Germany, which we analyse alongside 2,500 ancient and 379 modern genomes. Population genetic analyses reveal a major demographic shift coinciding with the late fifth century collapse of Roman state structures, when a founding population of northern European ancestry mixed with genetically diverse Roman provincial groups. Pedigree reconstruction and filia, a method for inferring the ancestry of unsampled relatives, indicate widespread intermarriage and minimal cultural differentiation. Genetic structure persisted through the sixth century, with admixture forming a population resembling modern Central Europeans by the early seventh century. Using Chronograph to refine the chronology of genealogically linked individuals, we estimate a generation time of 28 years, life expectancies of 39.8 years for women and 43.3 years for men, high infant mortality, and a society in which nearly one quarter of children lost at least one parent by age 10, yet most still grew up with grandparents. Pedigrees further reveal a society centred on nuclear families that practiced lifelong monogamy, strict incest avoidance, flexible lineage continuation and no levirate unions, indicating continuity with Late Roman social practices that later shaped the European family.

## Abstract (web summary)

Analysis of 258 ancient genomes from southern Germany reveals a major demographic shift during the late fifth century, yet family structures persisted from Late Roman times, demonstrating cultural continuity despite profound demographic reorganization.

## Main

During the transition from Late Antiquity to the Early Middle Ages (fourth to seventh century ce), Central Europe experienced profound political, cultural and demographic changes, marked by the dissolution of Roman rule, the spread of Christianity, and new settlement patterns. The political landscape shifted dramatically, with the emergence of new polities in Western and Central Europe. Yet knowledge of local societies and the lives of non-elite people remains limited, as written sources are scarce and few settlements have been fully excavated^4^. Cemeteries linked to rural settlements therefore provide key information on this transition. From about 450 ce onwards, distinctive furnished ‘Row-Graves’ appeared across the former Roman frontier regions, from Northern France and the Netherlands to Northern Italy and Western Hungary^5^. These burials, often furnished with clothing, weapons, jewellery or vessels, offer unique insights into everyday life and death in fifth to seventh century Europe^6^. The local societies associated with the Row-Graves in Southern Germany are usually characterized as small agrarian communities, sustained by crop cultivation and livestock (pigs and cattle), yet embedded in wider networks and developing social hierarchies. Some graves show Christian symbols by the end of the fifth century^7–10^.

**Fig. 1:**
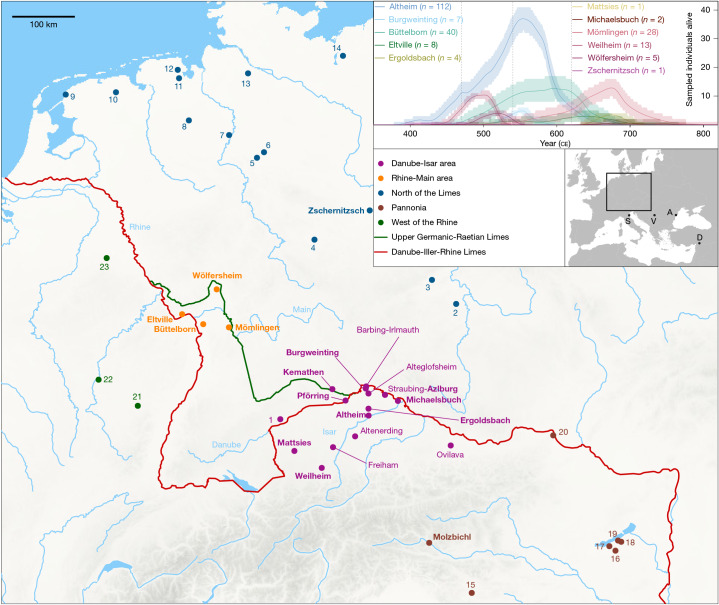
Location and chronology of the sites examined in this study. The Upper Germanic-Raetian Limes (green) marked the border of the Roman Empire until the second half of the third century ce, then replaced by the Danube-Iller-Rhine Limes (red) until the late fifth century ce. Late Antique sites (Azlburg, Pförring and Kemathen) and Early Medieval sites with newly reported genomes are in bold characters, whereas sites from outside the core region are marked by letters: Viminacium (V), Argamum (A), Spina (S) and Doliche (D). Published reference sites are numbered: Niederstotzingen (1), Brandysek (2), Konobrze (3), Hassleben (4), Hiddestorf (5), Anderten (6), Liebenau (7), Drantum (8), Midlum (9), Groningen (10), Zetel (11), Schortens (12), Issendorf (13), Häven (14), Ljubljana (15), Hács (16), Fonyód (17), Szólád (18), Balatonszemes (19), Klosterneuburg (20), Sarrebourg (21), Metz (22) and Alt-Inden (23). Top right inset, mean number (with 90% credible intervals indicated by shaded region) of sampled individuals alive at a given time according to Chronograph posterior estimates.

To broaden our understanding of demographic processes in this shifting socio-cultural landscape, we sequenced 221 Early Medieval genomes from multiple archaeological sites, with a focus on the northern frontier zone of the Roman Empire in present-day Southern Germany. We investigated Row-Graves from two regions: (1) the Danube-Isar area in Upper and Lower Bavaria, with a focus on Weilheim and Altheim—the latter notable for its securely dated early fifth century layers and extensive archaeological study^11^; and (2) the Rhine-Main area, mainly represented by Büttelborn and Mömlingen (Fig. 1). During Antiquity, both regions were part of the Roman Empire. The Rhine-Main area belonged to the province of Germania Superior until the late third century ce, when the border was moved westwards to the river Rhine and the remaining provincial territory was reorganized as Germania Prima and Sequania until the dissolution of Roman rule during the fifth century. The Danube-Isar region belonged to Rhaetia Secunda until the collapse of the Western Roman Empire and probably came under Ostrogothic control in the late fifth century, though the extent of its reach remains debated^12^. By around 540 ce, the region fell under Frankish influence, with a military command led by a duke (dux) that later evolved into the Duchy of Bavaria^12,13^.

**Extended Data Fig. 1:**
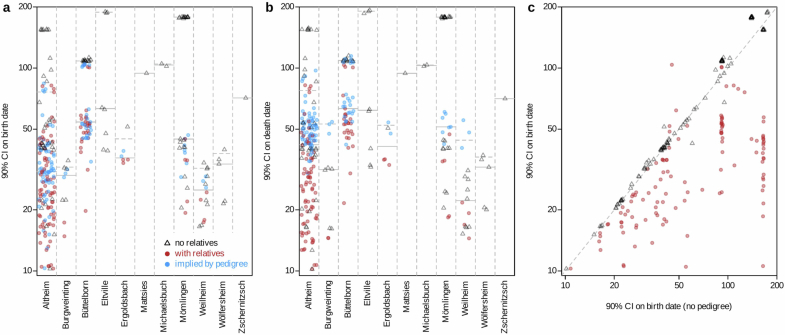
Estimation of Chronograph accuracy. **a** and **b**) Shown are the 90% CI on the birth (**a**) and death (**b**) dates per individual per site. Symbols and colour distinguish between sampled individuals without relatives (“no relatives”, black open triangles), sampled individuals with relatives (“with relatives”, red circles) and unsampled individuals implied by pedigree information (“implied by pedigree”, blue circles). Horizontal bars indicate the 50% and 90% quantile for each site. Credible intervals are generally much narrower for samples with relatives, i.e. for individuals whose dates are constrained by that of other individuals. This is also true for individuals that were not sampled but only implied by the pedigree, whose CI were generally larger than for sampled individuals with relatives, but still smaller than for sampled individuals without relatives. **c**) The 90% CI on the birth date for the full run (y-axis, as in **a**) against a run that ignores pedigree information (x-axis). The strong reduction in 90% CI for most individuals with relatives illustrates the strong benefit of using pedigree information. The one outlier with strongly increased 90% CI is individual Btb73, for which pedigree information renders secondary peaks of its ^14^C likelihood function more plausible. Note that without pedigree information, no estimation is possible on the “implied” individuals.

**Extended Data Fig. 2:**
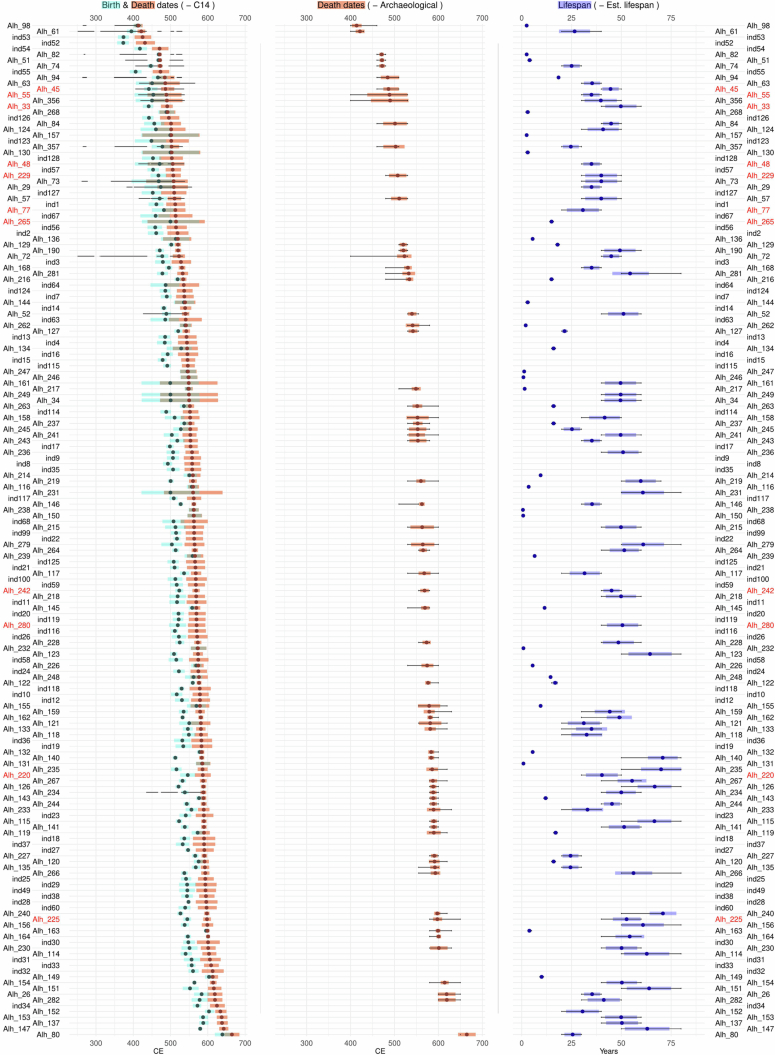
Life history estimates for individuals from Altheim. First column: posterior estimates (dot: mean, bar: 90% CI) of birth (turquoise) and death (red) dates; 90% CI of the recalibrated ^14^C date (black line, potentially disjunct), if available. Second column: posterior estimates of death dates; 90% CI of the archaeological date of the grave (black line), if available. Third column: posterior estimates of lifespan; 90% CI on the osteological age-at-death estimate (black line), if available. Red-coloured labels denote individuals identified as non-locals by isotopic analysis. Individuals whose names start with “ind” have not been sampled but were implied by familial relationships.

To assess the demographic and societal impact of these socio-political changes, we developed a novel Bayesian method, Chronograph, which jointly infers birth and death dates of all individuals by combining chronological signals from archaeological grave dating, radiocarbon measurements, genetic relationships and osteological age-at-death estimates (Supplementary Information 3). This approach yields precise birth date estimates: 50% of samples have 90% credible intervals narrower than 39.1 years, and 90% of samples have intervals smaller than 108.2 years. For individuals with identified close relatives, including those inferred from pedigree data, these intervals further reduce to 33.8 and 57.5 years, respectively (Extended Data Figs. 1 and 2, Supplementary Figs. 3.3–3.6 and Supplementary Table 2.3). In downstream analyses, we fully accounted for the remaining uncertainty using posterior samples of all individuals alive at a given time or period (Fig. 1, top right, and Supplementary Fig. 3.7).

For comparative purposes, we supplemented the dataset with newly generated and published genomes from the fourth to eighth centuries in present-day Southern and Eastern Germany, Austria, Italy and Hungary^14–21^. To explore genomic variability in earlier periods, we additionally sequenced 20 genomes from nearby Late Antique sites (Azlburg, Kemathen and Pförring) and 16 genomes from key sites across Europe and beyond (Viminacium, Serbia; Argamum, Danube Delta; Doliche, Anatolia; and Spina, Italy) (Fig. 1, Supplementary Information 1 and Supplementary Table 1), yielding a total of 258 newly generated genomes with a median depth of 2.25×. For Altheim, an additional 114 strontium isotope ratios were measured to further investigate patterns of individual mobility and migration (Supplementary Information 2).

## Ancestry shifts and population structure

**Fig. 2:**
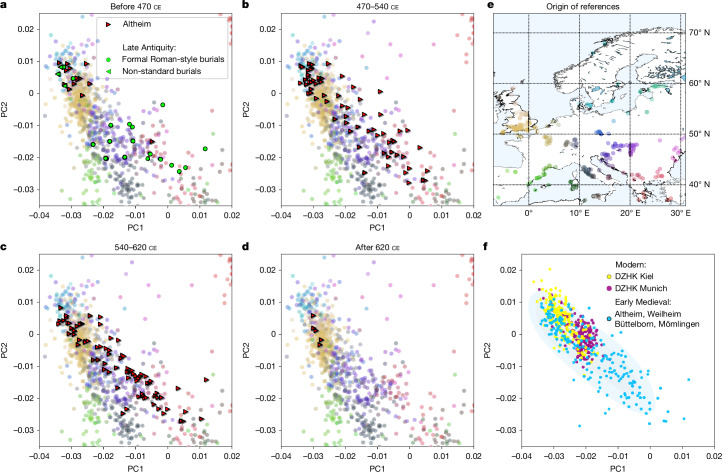
PCA of genetic variation in Altheim across time, alongside comparisons with Late Antique and modern populations. **a**–**e**, The chronology is based on individuals potentially alive during the time-window, estimated with Chronograph: before 470 ce (**a**), 470–540 ce (**b**), 540–620 ce (**c**) and after 620 ce (**d**). The background reference genomes represent genetic variation across Europe mainly from 800 bce to 1 bce, colour-coded by geographic origin (depicted in **e**; for a larger version, see Supplementary Fig. 7.4). Genomes from Late Antiquity from the Danube-Isar region and Upper Austria shown in **a** are Azlburg and Ovilava (regular Roman-style burials); Pförring and Kemathen (non-standard burials). **f**, PCA of Early Medieval genomes with 75% contour lines compared to modern individuals sampled at two German hospitals: Munich (Southern Germany) and Kiel (Northern Germany).

We first performed principal components analysis (PCA) to compare individuals from our main site, Altheim in the Danube-Isar region, with a dataset of more than 1,600 Iron Age and pre-Roman genomes from western Eurasia (Fig. 2c, Supplementary Tables 2 and  2.1 and Supplementary Fig. 7.4). This analysis revealed three distinct ancestry phases in the Altheim graveyard (400–470 ce, 470–620 ce and 620–660 ce), indicative of major demographic shifts (Fig. 2).

**Extended Data Fig. 3:**
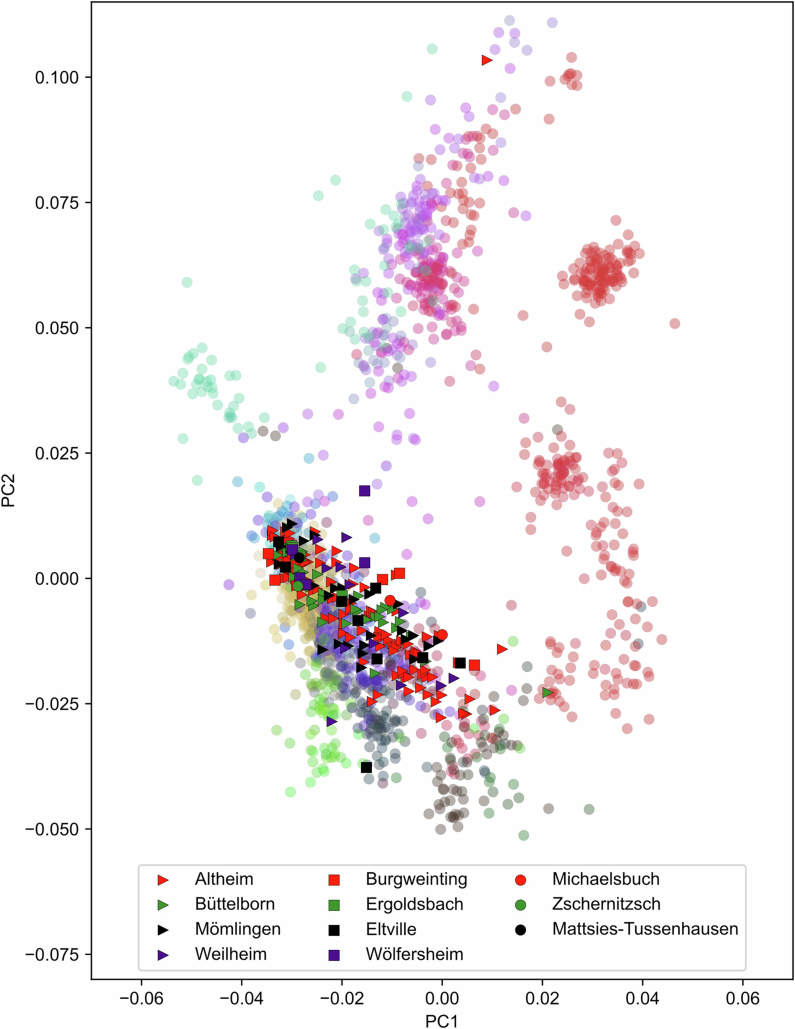
Principal Component Analysis. First two principal components for newly sequenced Early Medieval genomes plotted alongside previously published genomes dating between 800 and 1 bce from western Eurasia, shown as individual coloured points in the background. Colours for the reference genomes were assigned based on the latitude/longitude coordinates listed in the AARD file and match those in Fig. 2e. Archaeological sites are symbol-coded by sample size: triangles (≥10 individuals), squares (4–9 individuals), circles (<4 individuals).

In the earliest phase (400–470 ce), fewer than 20 individuals (either directly sampled or implied by connecting sampled relatives up to third degree) were alive at any given time (Fig. 1, top right, and Supplementary Fig. 3.7). These individuals cluster with Iron Age northern Europeans (Fig. 2a, Extended Data Fig. 3 and Supplementary Figs. 7.3 and 7.4) and with present-day populations, above all from Northern Germany (Fig. 2), the Netherlands and Denmark (Supplementary Figs. 7.1 and 7.2), consistent with northern ancestral origins^22^. Hereafter, we use the term ‘northern ancestry’ to denote this genetic background. In the subsequent phase (470–620 ce), the number of identified individuals increased rapidly, reaching a peak of 70 around 550 ce (among whom 37 were sampled). Individuals from this period display a markedly broader distribution in PCA space than those from either the preceding or the following phases. Whereas some still overlap with Iron Age populations from Northern Europe, others show affinities to groups from the western Mediterranean or Southeast Europe, or fall entirely outside European genomic variation (Supplementary Figs. 1.2, 7.2 and  7.3), indicating a substantial influx of people with diverse ancestries. The PCA also shows that the Frankish takeover around 540 ce caused no detectable shifts in population structure (Fig. 2b,c).

**Fig. 3:**
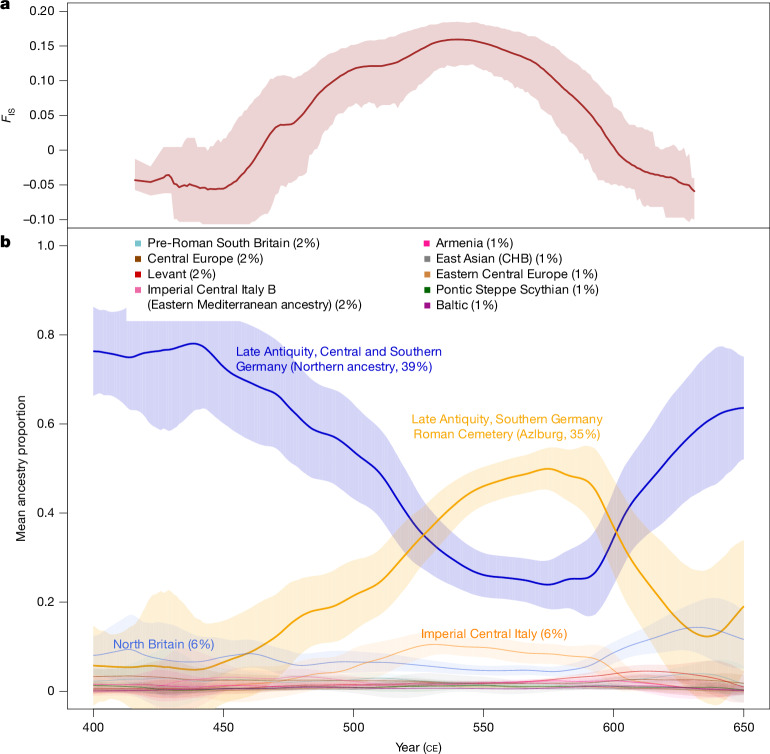
Temporal dynamics of substructure and ancestry in Altheim. **a**, Deviation from Hardy–Weinberg equilibrium over time in Altheim measured as mean *F*_IS_ (solid line) for unrelated individuals sampled from 15,000 iterations For a fine-scaled characterization of theshaded area. **b**, Mean ancestry proportions of individuals alive at a given time in a model with 13 source populations, including local reference groups from the region; 95% credible intervals are indicated by the shaded areas.

The high inter-individual diversity observed in PCA space between 470 and 620 ce is mirrored in uniparental loci and in comparisons with present-day Germans from northern and southern cities (Kiel and Munich; Fig. 2f), highlighting the genetic heterogeneity of the population. However, overall autosomal genetic diversity estimates (*θ*) are slightly higher in present-day populations (Extended Data Table 1). Although *θ* may be underestimated in historic genomes, this pattern suggests that Altheim during this period was more genetically structured, with individuals being less admixed. To examine this further, we quantified deviations from Hardy–Weinberg equilibrium (*F*_IS_), and found that it increased sharply after about 470 ce, peaked around 550 ce, and then gradually declined towards the seventh century ce, consistent with transient substructure and a process of social integration (Fig. 3a and Extended Data Table 1).

For a fine-scaled characterization of the ancestral components contributing to the high genetic diversity at Altheim, we used the genealogy-based ancestry painting approach in twigstats^22^, based on ancestral recombination graphs from Relate^23^, leveraging the resolution of whole-genome sequencing data (Fig. 3b, Supplementary Information 8 and Supplementary Tables 2.4–2.6). At a continental scale, the 470–620 ce population at Altheim can be modelled by source populations spanning Northern, Southern and Southeastern Europe (Supplementary Figs. 8.2–8.5), a pattern also supported by ancestry inference with ChromoPainter2 (ref. ^24^) and PANE^25^ (Supplementary Information 8 and Supplementary Tables 2.7–2.10). More than three quarters of the ancestries identified can be attributed to co-occurring northern sources (Northern Europe (34%), North Britain (9%), Pearson’s *r* = 0.48, *P* < 0.001; Supplementary Fig. 8.3) and southern or southeastern European sources (Roman Southeastern Europe (20%), Iron Age Central Italy (16%), *r* = 0.21, *P* < 0.01; Supplementary Fig. 8.3). Roman Southeastern Europe stands out as a principal source population, a pattern further supported by Y-chromosomal haplogroup frequencies (Supplementary Information 6 and Supplementary Fig. 6.2), probably reflecting the role of the Balkans as a major recruitment hub for the Roman army^26^. Contributions from Central Europe, Pontic Steppe and Baltic source populations remain minor.

**Extended Data Fig. 4:**
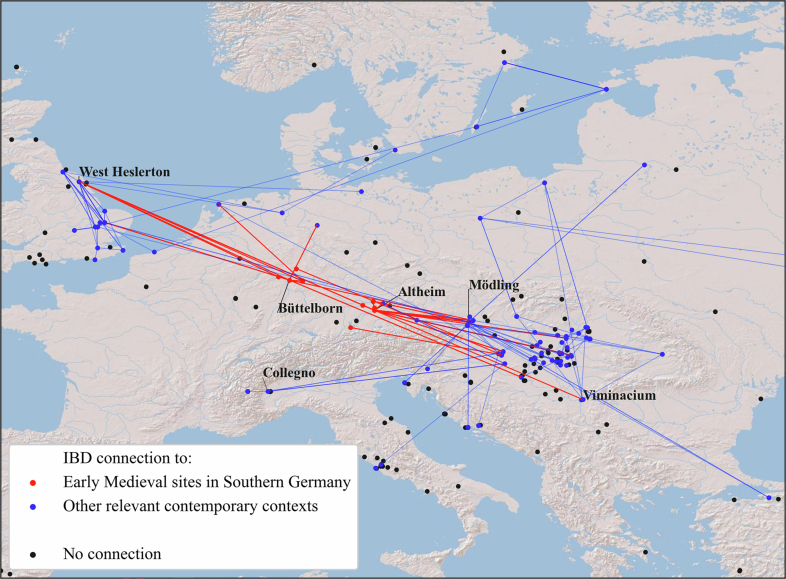
IBD connections. Map of pairwise identical-by-descent (IBD) fragments shared between individuals from Antiquity and the Early Middle Ages (1–800 ce). Red lines connect newly published Late-Antique and Early-Medieval individuals from southern Germany who share at least one ≥ 20 cM genomic segment; light-blue lines indicate analogous links among previously published contemporaneous genomes. The overwhelming majority of IBD tracts occur between individuals with a northern-European ancestry profile. Sites with the highest number of sampled individuals in each region are labelled by name.

Notably, however, the major ancestry sources, Northern Europe, Roman Southeastern Europe and Iron Age Central Italy were already represented in local groups within the study region before the emergence of the earliest Row-Grave cemeteries in Southern Germany (Fig. 2a). Prior palaeogenetic studies have suggested southward movements of northern European groups into Central Europe and Pannonia by the late fifth century^15,19,22,27^, but the precise timing remained uncertain. Several newly sequenced individuals presented here, with northern ancestry, including those from Pförring and Kemathen, as well as the earliest Altheim burials, Alh_61 (400–425 ce) and Alh_98 (412–414 ce), predate the Row-Grave horizon. Sharing patterns of chromosomal identical-by-descent (IBD) segments further corroborate evidence for an earlier migration from the North into the Roman frontier zone: long IBD segments (>20–400 cM) connect sites within the Danube-Isar area, whereas only shorter segments (<20 cM) are shared between individuals from Danube-Isar and Rhine-Main sites, consistent with a degree of regional isolation (Extended Data Fig. 4 and Supplementary Fig. 10.1). Together, these findings indicate that many individuals with northern ancestry were already established in the Roman frontier zone by the late fourth century.

Roman Southeastern European ancestry was likewise established in the region by the fourth century ce. At the nearby Roman military base of Azlburg, most individuals carried this component alongside Iron Age Central Italian ancestry, while others also exhibited northern European sources, reflecting the diverse composition of the Late Antique Roman military. The population of Altheim can be modelled well using these local sources (Fig. 3b).

**Extended Data Fig. 5:**
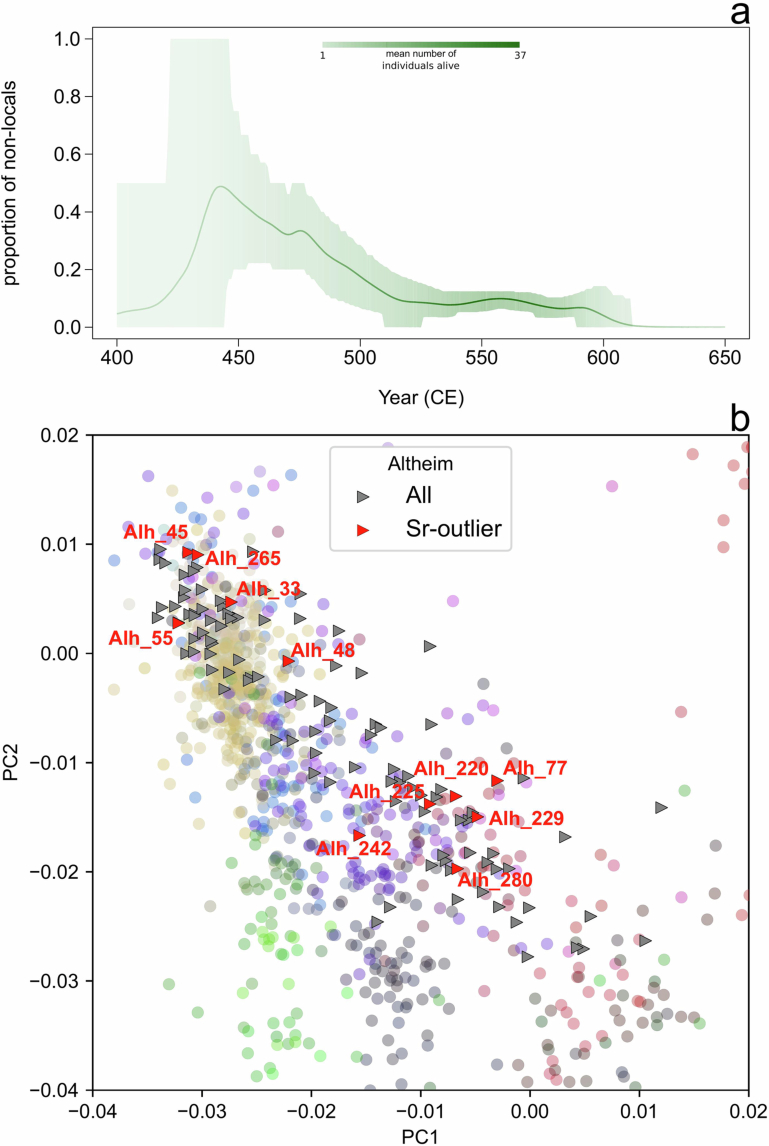
Non-local individuals. **a**. Fraction of non-local individuals in the Altheim population, inferred from ^87^Sr/^86^Sr ratios. Values were computed annually across 15000 MCMC iterations generated by Chronograph; the solid line shows the mean, and the shaded area represents the 90% credible interval (5th–95th percentiles). Plot transparency reflects the mean number of individuals alive at each time point across all iterations. **b**. Principal component analysis (PCA) of Altheim individuals (grey triangles), with those exhibiting non-local strontium isotope signatures highlighted in red.

Strontium isotope data are consistent with a predominantly local origin of most Altheim individuals. Only a minority of Altheim individuals show non-local signatures, that could theoretically derive from several regions outside of the Bavarian Alpine foothills but in light of the archaeological and historical context, are more likely to originate from less distant areas north of the Danube. Notably, the earliest six identified non-locals were all women. The proportion of non-locals declines from about 35% (90% credible interval: 21–50%) around 470 ce to about 7% (90% credible interval: 3–12%) by 540 ce, and disappears entirely by 620 ce (Extended Data Fig. 5 and Supplementary Fig. 2.2), indicating reduced mobility during the phase of increased population structure and subsequent admixture, and in line with trends in the wider region^28^.

**Extended Data Fig. 6:**
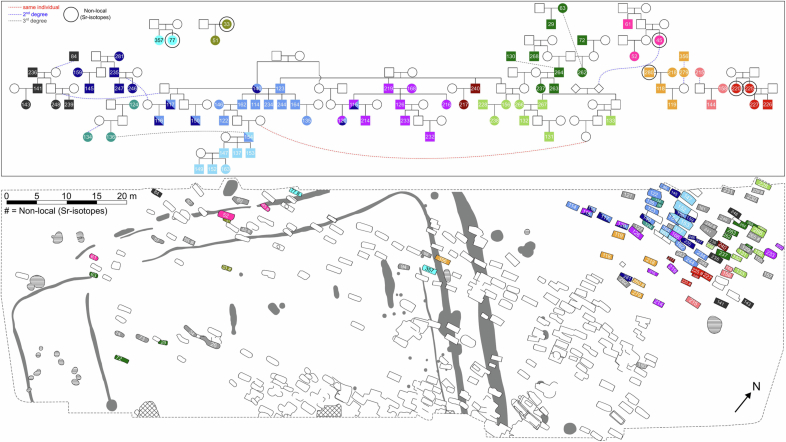
Spatial distribution of family lineages in the Altheim graveyard. Grave plan courtesy of Bayerisches Landesamt für Denkmalpflege; Dr. Johannes Sebrich.

The emerging scenario depicts a population of northern ancestry, already established in the frontier zone and potentially supplemented by continued influx from the North, incorporating substantial Azlburg-like ancestry from neighbouring Roman contexts from 470 ce onwards. Concurrently, burial activity at Altheim shifted to the northeastern section of the cemetery (Extended Data Fig. 6, Supplementary Fig. 14.1 and Supplementary Video 1). Over the following 150 years, interactions between the founding groups with northern ancestry and later arrivals from Roman provincial communities produced high genetic diversity and population substructure. As admixture proceeded on site (direct evidence presented below), both inter-individual diversity and substructure gradually declined. Individuals buried after 620 ce again cluster with modern and Iron Age northern and central European genomes, but compared with the first phase they are shifted slightly towards southern and southeastern European individuals in PCA space. During this period, the number of sampled individuals drops to around 10, either reflecting a true demographic contraction or more likely a shift in burial practices to as-yet unsampled or unexcavated areas^11^. However, the presence of several close relatives among these samples may exaggerate the apparent speed at which genetic ancestry converges during this period towards what later forms the characteristic genetic signature of southern Germany (Fig. 2f).

## Similar demography across South Germany

Data from other Early Medieval cemeteries in Southern Germany (Fig. 1) align with the model established for Altheim, indicating comparable demographic processes across regions. Although smaller sample sizes limit chronological resolution, sixth century sites consistently show wide ancestry variation, ranging from Northern to South or Southeastern Europe (PCAs in Supplementary Information 1 and Supplementary Fig. 7.2). This applies also to inland sites deep within long-settled former Roman territory and far from the frontier, such as Weilheim (480–540 ce), whose ancestry, as for post-470 ce Altheim, can be modelled by these proxy sources: 37% from the Roman cemetery of Azlburg, 34% northern ancestry sampled in Central Europe, and 12% from Northern Britain (Supplementary Fig. 8.8). If a proportion of northern ancestry plausibly derived from distinctly Roman military or related civilian contexts, then the majority of ancestry in the late fifth to early sixth century Row-Grave cemetery would have been genuinely Roman in origin. In the Rhine-Main region, sixth century genomes from Eltville, near Moguntiacum (modern Mainz), similarly resemble Altheim’s middle phase (470–620 ce). A comparable composition is observed two to three generations later at Büttelborn and Mömlingen (Supplementary Fig. 9.1).

Alongside broadly similar patterns, comparison of the Rhine-Main and Danube-Isar regions reveals a few notable differences. The Danube-Isar Early Medieval genomes carry higher Iron Age Central Italy ancestry, whereas those from the Rhine-Main region show higher British (both Northern Britain and Pre-Roman south Britain), Eastern Central Europe and Baltic ancestry (one-sided Mann–Whitney *U* test, *P* < 0.05, Supplementary Fig. 8.4). This pattern corresponds to the variation observed in PCA space: the Rhine-Main cluster lacks the southernmost component of the gradient, indicating partly distinct ancestral sources rather than simply changing proportions of the same ones. Consistent with these differences, *F*_ST_ values between settlements are high and clearly exceed those observed between modern northern or southern German populations (Extended Data Table 1).

Although an overall blending of ancestries is evident throughout, reflective of broad supra-regional demographic shifts, distinct individual ancestries persist well into the later phases of the Row-Grave horizon. At Altheim, six individuals dating to the sixth century show ancestries predominantly attributed to Italian sources, with only minimal northern admixture. At Michaelsbuch, two individuals still, even in the eighth century, carry ancestries that are strikingly atypical for the region today, reflecting lingering pockets of post-Roman structure, later gene flow from outside the area, or a mixture of both. More distant ancestries are also apparent: a male from Altheim (Alh_245; 528–553 ce) who shares long IBD segments with individuals from the Berel necropolis in modern Kazakhstan, derives roughly two-thirds of his ancestry from East Asian sources and one-third from populations of the western Steppe. A contemporary male from Wölfersheim (W67) carries similar, albeit less of this Asian ancestry, whereas late fifth century females with artificial cranial deformation (Wh4 and Wh59) lack Steppe-related ancestry and instead exhibit patterns consistent with post-Roman admixture^14^.

From a historical perspective, individuals from Altheim predating 470 ce may have been descendants of Roman soldiers or peasants who had lived in the frontier zone, or they may have been settled in the Altheim area as agrarian workers by Roman authorities in the early fifth century^2^. As Roman authority waned in the later fifth century, the collapse of military and economic structures loosened the social and legal bonds that had tied dependent peasants, such as *coloni* and slaves, to their landlords, thereby facilitating regional mobility. Although long-distance migration cannot be ruled out in individual cases, it is not necessary to explain the emerging genetic patterns. Instead, the regional mobility of day labourers, merchants, and others likely sustained a steady influx into Altheim during the sixth century. Furthermore, military conflicts in the Danube-Isar region may have displaced local peasants and mobilized armed groups. A similar scenario may explain the genomic patterns observed in sixth century individuals in the Rhine-Main region: with the dissolution of Roman military structures along the Rhine frontier in the fifth century and the accompanying collapse of economic and social networks, individuals, families and groups began to move, some of them across the river Rhine, establishing new communities or joining existing ones.

## Demographic shift linked to intermarriage

**Fig. 4:**
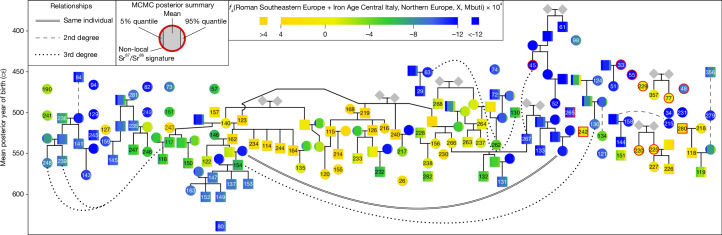
Reconstructed pedigrees of Altheim with colour-coded *f*_4_-statistics presented in a chronological context. Sampled individuals are numbered and inferred individuals are depicted by empty markers. Individuals not connected to a pedigree are displayed for contextual reference. All *f*_4_-statistics were inferred with filia. Red outlines indicate individuals of non-local origin based on strontium isotope analysis. MCMC, Markov chain Monte Carlo.

To evaluate the consequences of the inferred demographic shifts at the family level, we reconstructed pedigrees and applied filia, a novel method that leverages familial relationships to infer *f*_4_-statistics for unsampled individuals (Supplementary Information 13 and Supplementary Table 2.11). At Altheim, this approach reveals immediate intermarriage between individuals of predominantly northern European ancestry and those with Roman Azlburg-like ancestry, indicating rapid integration of newcomers, regardless of their individual ancestry, into the local community (Fig. 4). Analysis of the 24 individuals with the most extreme *f*_*4*_ values shows considerable individual and chronological variation in grave furnishings, but no systematic differences between burials of individuals at the high versus low ends of the distribution (Supplementary Table 2.12). Although the archaeological interpretation is somewhat complicated by the apparent reopening of many burials^11^, this pattern suggests that material culture was largely decoupled from genetic ancestry. These findings contrast with those from Szólád in Pannonia and Collegno in Northern Italy^15,29^, as well as with Burgweinting near Altheim, where two adjacent burial groups differ sharply in ancestry and material culture: four women of nearly exclusive northern ancestry were buried with lavish goods, whereas a neighbouring mixed-ancestry group had modest furnishings (Supplementary Fig. 1.9). By contrast, Altheim reflects a simpler rural lifestyle, without comparable social or cultural differentiation.

## Life-history parameters

**Fig. 5:**
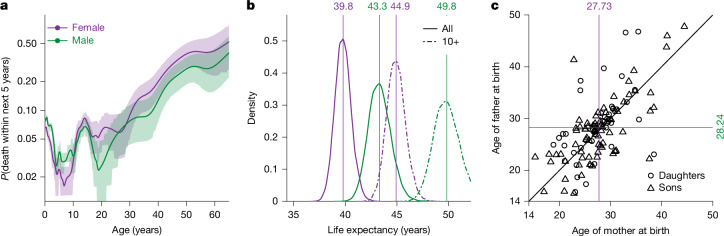
Life history traits inferred by Chronograph. **a**, Posterior mean (solid line) and 90% credible interval (shaded region) of the fraction of individuals alive at a given age that die within the next five years. **b**, Posterior distribution (curve) and mean (vertical lines) life expectancy (average lifespan) for all individuals (solid curves) or those that survived at least until 10 years of age with more than 90% posterior support (dot-dashed curves). **c**, Posterior estimates of the mean age of mother and father at birth for each identified child (multiple children per parent). Solid coloured lines indicate the resulting posterior mean estimate of the generation time for women (purple) and men (green).

Fine-grained demographic data on local societies in Late Antique and Early Medieval Europe remain scarce. For the Roman Empire, epigraphic and literary sources provide diverging estimates of mortality and marriage ages^30,31^. In Early Medieval Provence, a study of more than 1,000 peasants indicates that boys and girls became eligible for marriage at around the age of 12, life expectancy at birth was about 20 years, and women married earlier than men while generally avoiding remarriage^32^. At Altheim, using birth and death dates inferred by Chronograph, we could generate reliable estimates of key life-history parameters for a local Early Medieval community. Both infant and child mortality were higher for boys than for girls, with 9.7% (90% credible interval: 9.7–9.7%) and 7.8% (90% credible interval: 7.7–8.3%) of all identified boys and girls, respectively, not reaching the age of seven (Supplementary Fig. 3.8). Nonetheless, men had a higher life expectancy (43.3 years, 90% credible interval: 41.6–45.2) than women (39.8, 90% credible interval: 38.5–41.1), driven by a higher mortality of females after about 10 years of age (Fig. 5 and Supplementary Fig. 3.8), suggesting that giving birth was a major risk factor. The mean generation time was 28.0 years (90% credible interval: 26.4–29.6; Fig. 5 and Supplementary Fig. 3.9). Half-orphans were relatively common, with 11.3% (90% credible interval: 6.4–17.0%) and 25.5% (90% credible interval: 17.7–34.0%) of all children having lost at least one parent by the age of five and ten, respectively, whereas only 0.5% (90% credible interval: 0.0–1.9%) and 2.5% (90% credible interval: 0.0–5.9%) had lost both parents by the same age, respectively (Supplementary Fig. 3.11). However, the majority of children grew up with grandparents: 81.8% (90% credible interval: 54.6–100%) of all children had at least one grandparent alive at birth and 67.4% (90% credible interval: 30.0–90.0%) had at least one grandparent alive at ten years of age (Supplementary Fig. 3.11). Although we estimate that mothers were on average 0.7 (90% credible interval: −6.5–11.7) years younger than their partner, posterior support for an older father exceeded 90% for only 5 out of 77 couples (6.5%; Supplementary Fig. 3.10).

## Family structure and cultural legacy

**Fig. 6:**
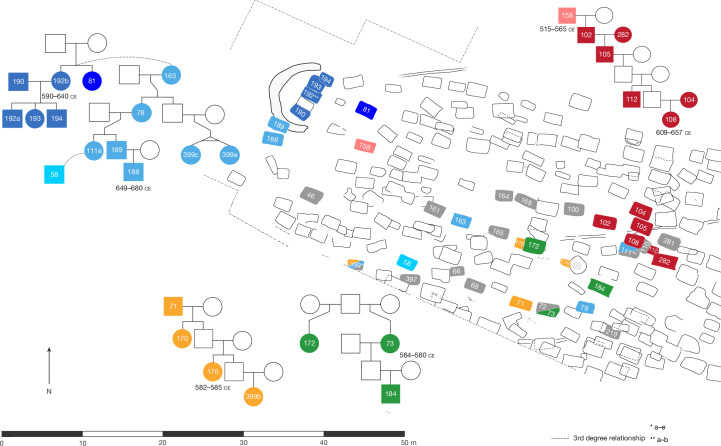
Spatial arrangement of reconstructed pedigrees in the Büttelborn cemetery. Colour-coded pedigrees mapped onto individual graves; graves of unrelated individuals are shown in grey. Chronograph estimates of birth and death dates are shown for individuals with a ^14^C date. A corresponding depiction for Altheim is provided in Supplementary Fig. 14.1. Grave plan courtesy of Thomas Becker, hessenARCHÄOLOGIE, Außenstelle Darmstadt.

There is a long history of research examining family structures between Late Antiquity and the Early Middle Ages, as well as the influence of Christianity, as key factors in the development of modern European kinship systems^30,33–36^. However, sources are mostly normative texts or document elite practices, whereas little is known about non-elite local societies beyond the findings of a small number of genomic studies, all from geographically, chronologically or culturally distinct regions^37–41^. To evaluate spatial clustering among kin, we analysed grave distribution at Altheim (Supplementary Fig. 14.1) and Büttelborn (Fig. 6). At Altheim, first- to fourth-degree relatives (N = 77–137) were buried significantly closer together than unrelated individuals (*n* = 3,007; Mann–Whitney *U* test, two-sided, *P* < 1.08 × 10^−17^ to 1.04 × 10^−3^), with spouses (*n* = 6) also interred in close proximity (Supplementary Information 14). At Büttelborn, spatial placement was also shaped mainly by close biological relatedness, with distant kin exerting little influence. For example, a father, mother and three children occupy a circular cluster separated from more-distant relatives, such as an aunt and distant cousins (Fig. 6). Assuming that burial proximity reflects social relations, these patterns are consistent with communities being organized around nuclear or stem families, sometimes complemented by extended kin, such as half-siblings. This is reflective of global patterns in agrarian societies, in which small core family units are typically embedded within broader kin networks^42^, and aligns with historical research on local societies since the early Roman Empire^35^ and in Southern Germany after about 750 ce ^(ref. (43^).

**Extended Data Fig. 7:**
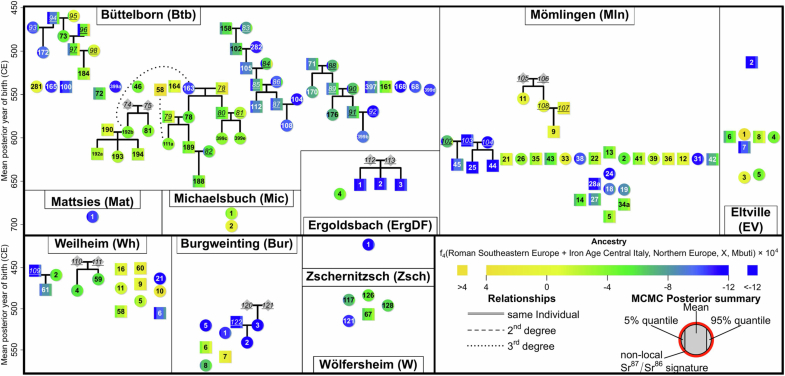
Reconstructed pedigrees from sites other than Altheim. Pedigrees are color-coded according to f_4_-statistic results and displayed in chronological order. Sequenced individuals are shown in bold, individuals inferred from pedigree structures, are shown in italics and underlined. Individuals not connected to any pedigree are included for contextual reference.

Drawing on reconstructed pedigrees from Altheim (Fig. 4) and Büttelborn (Fig. 6, Extended Data Fig. 7 and Supplementary Fig. 12.2), we examined which rules of residence and descent are compatible with such data. Pedigrees more often continued through sons (20 and 9 cases in Altheim and Büttelborn, respectively; Supplementary Table 14.3), although continuation through daughters also occurred (9 and 2 cases, respectively; Supplementary Table 14.3). At Büttelborn, five generations of male relatives (Fig. 6, red pedigree), were buried in a tight cluster. By contrast, much less spatial clustering can be observed for the individuals of a pedigree connecting mostly women over four generations (Fig. 6, orange). Across sites, women share significantly larger IBD segments with individuals buried elsewhere than men (Mann–Whitney *U* test: two-sided, *P* < 0.0442; Supplementary Table 10.3 and Supplementary Figs. 10.8–10.11). Within sites, however, they share far fewer IBD segments (>8 cM) in comparison to men (Altheim: 30.92 ± 5.28 versus 170.55 ± 13.08; Mann–Whitney *U* test: two-sided, *P* < 1.47 × 10^−^^61^; Supplementary Table 10.2), and their relatedness coefficient is 6-fold lower at Altheim and 1.5-fold lower at Büttelborn. These inferred patterns are consistent with a flexible patrilocal system in which most women resided near their husband’s family, whereas some men settled near their wives (Supplementary Information 14), particularly in the absence of a son. However, the high Y-chromosomal and mitochondrial diversity at both sites is incompatible with strict patrilineal descent and strict patrilocality^44,45^. Instead, it indicates a flexible patrilineal or a bilateral system with flexible patrilocality in which pedigrees were continued mainly through sons but occasionally also through daughters. We further observed that, at least in Altheim, unions were predominantly exogamous when the pedigree continued through sons (16 out of 21 cases), whereas endogamy was more common when continuity was through daughters (5 out of 9 cases), a pattern that echoes those documented in modern matrilocal or matrilineal societies^46^. Together, patterns of residence and descent in Early Medieval Southern Germany closely mirrored those established during the Late Roman period. Written sources from the Roman Empire show a constant trend towards a bilateral inheritance system: in Roman law, daughters could, under certain circumstances, inherit equally with sons in cases of intestate succession. Yet restrictions on the ability of women to bequeath property, together with other succession rules, generally ensured that assets passed through the male line. From the early centuries ce onward, however, inheritance through daughters was gradually reinforced, a development that continued into Late Antiquity^47^. Wills sometimes privileged male descendants who received a larger share, especially when land was concerned, but daughters were often treated equally and, in the absence of sons, even became primary beneficiaries (ref. ^48^, pages 62–71). In the fifth and sixth centuries, both Western Roman and Justinianic law, as well as legal practice in western post-Roman kingdoms, provided further security for the inheritance rights of daughters and their children by will or on intestacy, and thus strengthened bilateral succession (ref. ^48^, pages 71–72). At the same time, in Late Roman society, family tradition was increasingly conceived of as deriving from both paternal and maternal lines^35^.

In the Late Roman and post-Roman West, Christian societies increasingly emphasized lifelong monogamy: while divorce faced stricter legal regulation, widowhood was elevated and remarriage was considered morally problematic^30,33,36,47^. Marriages between close kin were condemned, prompting numerous prohibitions by church councils and secular authorities from around 500 ce onward^34^. Christianity thus reinforced earlier social trends: literary and epigraphic studies indicate that close-kin marriage was already largely avoided in the pre-Christian Roman West, and lifelong monogamy had become a widely promoted ideal^30,34,49^. The *Lex Baiuvariorum* (tit. VII, 1), issued in the eighth century but reflecting earlier norms, confirms strict prohibitions of incest and levirate unions for Bavaria, where historical and archaeological research indicates the continued influence of Christianity after the collapse of Roman rule^13^. In line with these written sources, our data suggests that lifelong monogamy, with limited divorce or remarriage of widows, was the prevailing norm in sixth century Southern Germany: we identified 68 probable single-partner unions but only five individuals in Altheim and Büttelborn (three men and two women) who had children with multiple partners, and none of the cases is chronologically incompatible with serial monogamy (Supplementary Fig. 3.12). Although we cannot entirely rule out that children from other partnerships either did not exist or were buried elsewhere, the substantial coverage of our sample at Altheim (38%, 90% credible interval: 22–55%; Extended Data Table 1, Supplementary Information 12 and Supplementary Fig. 12.4) makes it unlikely that such a pattern would have systematically escaped detection.

The near absence of long (>12 cM) runs of homozygosity (Supplementary Table 2.13) and the lack of shared IBD segments (>8 cM) between spouses support strict incest avoidance, excluding relationships closer than the sixth degree (Supplementary Table 11.1). Given the small estimated community size (maximum of around 70 individuals per settlement per generation; Extended Data Table 1 and Supplementary Information 16), such avoidance probably encouraged intermarriage across broader social networks. However, a comparable absence of relatedness between spouses was also observed in endogamous unions, that is, among partners from Altheim. Recent genetic research suggests that incest avoidance was also present in non-Christian societies, such as Iron Age populations from the British Isles^50^, and Avar societies in the Western Pannonian plains^37,51^. However, the latter show evidence for levirate unions, which are absent in Altheim and Büttelborn, in accordance with the *Lex Baiuvariorum*.

Overall, Altheim is characterized by patrilocal tendencies and either patrilineal or bilateral descent rules, with pedigrees continuing through daughters more frequently than at previously studied sites (Supplementary Table 14.4), except in a matrilocal Iron Age society in southwest England^50^. The rate of multiple reproductive unions is low (Supplementary Table 14.4), and only a Neolithic community in north-central France shows a lower rate^52^. These findings align with written sources and suggest that by the sixth century ce, Central European agrarian societies already maintained residence, and marriage practices that were to become characteristic of Latin Christian Europe.

## Rethinking the transition

Although the transition from Late Antiquity to the Early Middle Ages has traditionally been framed as a conflict between northern ‘barbarians’ and a Roman Empire in decline, newer studies reveal a multifaceted transformation^1,3,19,29,53–55^. The integration of genomic, isotopic, written and archaeological evidence now provides a clearer view of demographic, cultural, and social dynamics in Central Europe, particularly regarding the everyday life of agrarian societies. Although most of the demographic processes described here occurred locally, they developed within the framework of earlier north-to-south migrations towards the Roman frontier. Analysis of long IBD segments (>20 cM) among individuals confirms extensive transregional migrations well before the emergence of the Row-Grave cemeteries. Thirty-seven individuals from the Rhine-Main and Danube-Isar regions share long segments with contemporaries from sites more than 200 km apart, spanning Northern Germany, the Netherlands, England, Austria, Hungary, Croatia and as far as Viminacium in eastern Serbia (Extended Data Fig. 4, Supplementary Figs. 10.1–10.7 and Supplementary Information 10). IBD sharing is strongest among northern ancestry individuals and decreases along PC1 (Spearman’s *R* = –0.89, *P* < 9.04 × 10^−8^). Biological kinship data indicate that this migration involved individuals and small kin groups rather than entire populations, with likely second cousins buried more than 270 km apart (Büttelborn–Hiddestorf), and multiple links connecting the Danube-Isar region to elite burials at Szólád (around 500 km away) and cemeteries near Vienna (for more details on transregional IBD networks, see Supplementary Information 10 and  12). Subsequent movements unfolded against this demographic landscape, largely established by the turn of the fourth to fifth century. Although it remains unclear whether these biological relationships correspond to social ties recognized by the individuals themselves, they may reflect transregional networks that fostered familial and social connections across Central Europe, extending to Pannonia and Northern Italy^15^. The persistence of such networks, embedded within Roman-influenced traditions and infrastructure, may have facilitated the later rapid and cohesive spread of the Row-Grave horizon across Early Medieval Europe.

At Altheim, burials first appeared around 414 ce, initially isolated in the landscape and belonging to individuals of northern European ancestry with genomic profiles distinct from those of central European Iron Age populations (Supplementary Fig. 7.9). Although migration from the north probably continued, the majority of these early individuals and those who followed probably descended from families long established within or near Roman territories. They may represent descendants of Roman soldiers who had lived in the frontier zone or peasants who were settled in the wider Altheim area as agrarian workers by Roman authorities in the early fifth century^2^. Although their ethnic or social self-identification remains unclear^55^, their predominantly intra-group marriages until the pivotal 470 ce horizon suggest a shared sense of belonging, possibly reinforced by legal restrictions on marriage applied to *coloni* and other non-elite groups^56^. Their burials combined Roman-influenced and other practices forming a hybrid culture of inhumation and furnishing of graves with clothing and jewellery that foreshadowed features of the Row-Grave horizon^13^.

The demographic shift around 470 ce saw people with ancestry typical of Roman towns and forts begin to move into the hinterland, being buried across Row-Grave cemeteries of Early Medieval Southern Germany. In line with findings for other regions of the Late Antique West^2^, the collapse of Roman structures in the late fifth century had probably weakened ties binding peasants to their land in Southern Germany, enabling regional mobility and sustaining a steady influx into local communities throughout the sixth century. Although ancestries are not identical between sites, reflecting the distinct genetic composition of Roman settlements in each region, the general process of derivation from Roman provincial populations is broadly similar across Southern Germany. Although long-distance migration probably occurred in individual cases or small groups—for instance, links between Altheim and sites such as Mödling and Czocorgasse^37^ probably reflect stepwise or direct movements into the Vienna Basin during the sixth century—large-scale migrations are not necessary to explain the broader emerging genetic patterns in Southern Germany after 470 ce.

**Extended Data Fig. 8:**
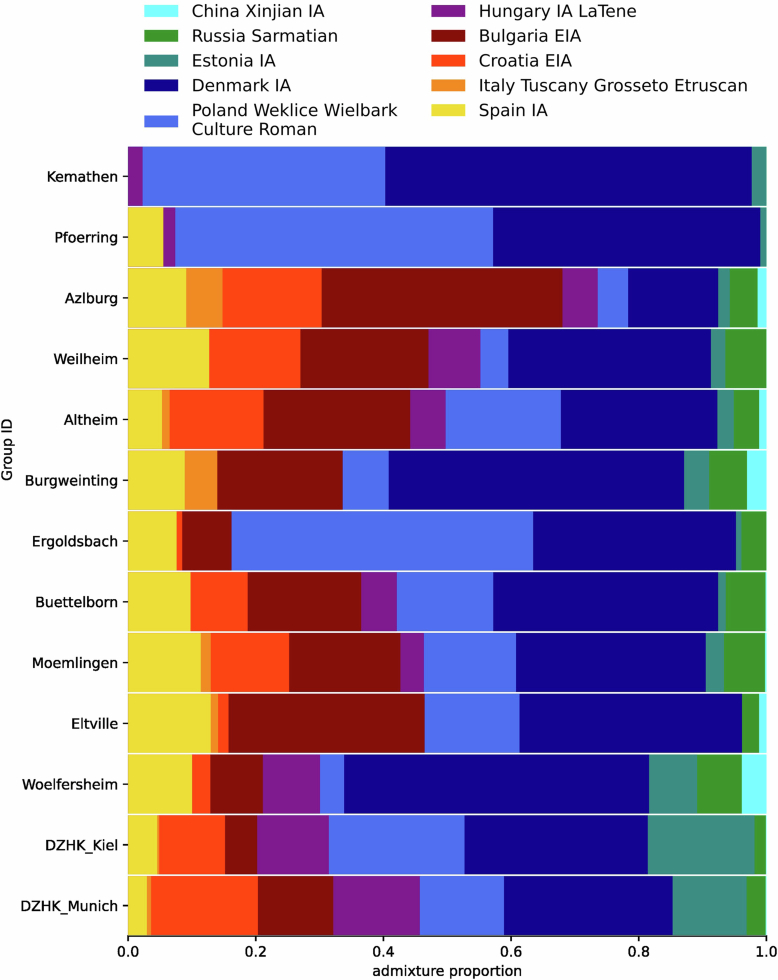
Ancestry estimates from PANE using a 10-population model. Bars represent average ancestry proportions for genomes from archaeological sites from Germany (sequenced in this study) spanning Late Antiquity to the Early Medieval period, as well as for the two DZHK reference cohorts from Munich and Kiel.

Over the following 150 years, intermarriage produced a population already genetically resembling modern Central Europeans, including southern Germans, with individuals of northern ancestry contributing disproportionately due to their numerical dominance, probably maintained by ongoing mobility. Roman-related ancestry left a modest but enduring signal in our study region, subtly modified by later northern and eastern inputs^41,57^, all of which remain detectable in genomes from present-day Germany (Extended Data Fig. 8 and Supplementary Fig. 8.22). Culturally, these local societies continued Roman practices of flexible inheritance and appear to have embraced Christian ideals such as lifelong monogamy, with minimal divorce or remarriage after widowhood. They also strictly avoided incest and levirate unions. Whether this pattern was unique to the Rhine-Danube frontier or part of broader trends across former northern frontier zones of the Roman empire remains to be investigated, but evidence from the Rhine-Main area presented here suggests wider applicability.

The genomic patterns in Southern Germany may also shed light on the region’s medieval vernacular, the everyday language spoken by people^15,19,22,27^. Networks among individuals of northern ancestry could have facilitated the spread of early Germanic dialects into Southern Germany, where Latin and local languages such as Gaulish had probably predominated. Although these older languages persisted mainly in personal and place names^58^, the relatively large contribution of northern ancestry may have promoted the emergence of pre-literary Old High German as the dominant vernacular in Southern Germany and neighbouring regions.

## Methods

Ancient DNA extraction, library preparation, sequencing, raw-read processing and variant calling followed ref. ^59^ (see Supplementary Information 4 and 5). Modern genomes from Deutsches Zentrum für Herz-Kreislauf-Forschung (DZHK; German Centre for Cardiovascular Research) Kiel and Munich cohorts were provided by the DZHK Heart Bank (https://dzhk.de/dzhk-heart-bank/daten-und-bioproben/dzhkomics-ressource). Reads were merged with ATLAS^60^, duplicates were removed with sambamba^61^, and realigned with GATK 3.8 (ref. ^62^). Phasing and imputation were performed with GLIMPSE2 (refs. ^63,64^) using bi-allelic loci from the 1000 Genomes dataset, with a minor allele frequency >1% for shotgun genomes and autosomal 1240k SNPs^65^ for array data. Mitochondrial and Y-chromosome haplotypes were assigned with haplogrep3 (ref. ^66^) and Yleaf2 (ref. ^67^).

To estimate individual birth and death dates, we developed Chronograph, a Bayesian method that integrates archaeological, radiocarbon, genetic, stratigraphic and anthropological age at death estimation (Supplementary Information 3). We used 20,000 posterior samples of birth and death dates to propagate the remaining uncertainty into downstream analyses.

Ancient genomes were projected onto a PCA of modern west Eurasian individuals taken from version 54 of the Allen Ancient DNA Resource (AARD)^68,69^ using smartpca from the EIGENSOFT package^70^. Ancient individuals were taken directly from the AARD if available, or were obtained from their respective ENA repository and processed as described^59^ (Supplementary Information 5). *f*_4_-statistics were computed using Admixtools2 (refs. ^71,72^).

Relate^23^ and twigstats were run following the approach of ref. ^22^. We kept only transversion sites where no more than 5% of the genomes showed a genotype probability <0.8 when running Relate, then used twigstats to compute pairwise *f*_2_-statistics on inferred genealogies with a cut-off of 1,000 generations. All pairwise qpAdm models were tested, using the five European populations, plus YRI and CHB from the 1000 Genomes project^73^ as right-populations. Individuals were clustered into sources by building a graph combining qpAdm *P* values and geographic information and applying Louvain clustering (see Supplementary Information 8 for details). We validated the Relate/twigstats results by re-running the same model on the same set of markers using ChromoPainter2 (ref. ^24^) in combination with the Bayesian ancestry inference approach implemented in sourcefindV2 (ref. ^74^). ChromoPainter2 was additionally run with a larger number of source individuals by adding available pre-Roman genomes sequenced on the 1240k array^65^ and subsetting the dataset to only contain autosomal regions of the capture array. We used a similar clustering approach based on geography and pairwise Euclidean distances between haplotype copying vectors to identify potential source groups (see Supplementary Information 8 for details). PANE^25^ was run using groups of individuals defined by previous publications as sources (see Supplementary Information 8 for details).

We identified runs of homozygosity using hapROH^75^ on genomes with more than 400,000 SNPs on the 1240k panel^65^. AncIBD was used on autosomal SNPs of the 1240k panel to detect identity-by-descent segments^76^ between newly sequenced genomes and a curated set of published European individuals (Supplementary Information 10). Biological relatedness was estimated using KIN^77^ and READ2 (ref. ^78^) with default parameters, and pedigrees reconstructed following ref. ^79^. Spatial coordinates for burials within graveyards were determined by overlaying the graveyard plan with a grid of arbitrary units and then recording the middle of each grave. Correlations between relatedness and burial position were assessed using Euclidean distances after grouping pairs by their degree of relatedness.

Pedigree-aware interpolation of *f*_4_-statistics were obtained with filia (Supplementary Information 13).

Theta was estimated using ATLAS^60^, on neutral genomic regions identified in ref. ^80^. Deviations from Hardy–Weinberg equilibrium (*F*_IS_ and *F*_IT_) and Hudson’s *F*_ST_ were estimated using scikit-allele 1.3.1 (https://github.com/cggh/scikit-allel), on a set of bi-allelic transversions to minimize the effect of post-mortem damage.

SLiM^81^ simulations were used to estimate community sizes by simulating populations with monogamous mating pairs of size *N* [250–500] distributed across *n* villages [2,5], connected by migration (*m* − [0–1]) for 10 generations in 1,000 replicates. In each replicate S individuals were sampled (Altheim: *S* = 112, Büttelborn: *S* = 40) and relatedness (*r*) over three generations was calculated. A regression analysis with the Python package statsmodels (v.0.14.4)^82^ was used to determine the relationships between *N*, *m* and *r* for both communities, given the observed values (see Supplementary Information 16).

Strontium isotope analyses followed ref. ^28^ (Supplementary Information 2).

Data for maps in Fig. 1 and Supplementary Fig. 8.1 were obtained from: GEBCO Compilation Group (2022) GEBCO_2022 Grid (https://www.gebco.net/data-products/gridded-bathymetry-data/gebco-2022)^83^ and Natural Earth (https://www.naturalearthdata.com), and plotted using R. All other maps, including Fig. 2e, Extended Data Fig. 4 and Supplementary Figs. 7.4, 8.17 and 10.1–10.7, were created using the basemap toolkit from the matplotlib library^84^ in Python 3, which uses cartographic data from Generic Mapping Tools (https://www.generic-mapping-tools.org/).

### Reporting summary

Further information on research design is available in the Nature Portfolio Reporting Summary linked to this article.

## Online content

Any methods, additional references, Nature Portfolio reporting summaries, source data, extended data, supplementary information, acknowledgements, peer review information; details of author contributions and competing interests; and statements of data and code availability are available at 10.1038/s41586-026-10437-3.

## Supplementary information

Supplementary Information This file contains Supplementary Information

Reporting Summary

Supplementary Table 1 Meta-Information for all individuals investigated for this study, sorted by archaeological sites.

Supplementary Table 2 This file provides a key to the tabs in the Supplementary Table 2 Excel spreadsheet. Individual-level genomic profiles—ancestry, quality metrics, and analytical outputs across methodologies.

Supplementary Table 3 Results of ancIBD analysis, containing IBDs shared among individuals buried at different archaeological sites.

Supplementary Table 4 Results of ancIBD analysis, containing IBDs shared among individuals buried within archaeological sites.

Supplementary Video 1 Evolution of Altheim grave field. This animation visualizes the emergence of graves over time. Grave opacity reflects the probability (from Chronograph MCMC traces) that an individual had already died by a given date: full colour = certainly dead; transparency = uncertainty. Grave plan courtesy of Bayerisches Landesamt für Denkmalpflege; Dr. Johannes Sebrich.

Supplementary Video 2 Spatiotemporal ancestry dynamics relative to the Roman Limes. 3D visualization of ancestry patterns for Late Antiquity and Early Medieval individuals (genomes from regions in Supplementary Fig. 9.1, excluding Pannonia and Viminacium), plotted against chronology and proximity to the Roman frontier. A locally weighted regression surface models the trivariate relationship between ancestry, time, and geographic distance.

## Figures and Tables

**Extended Data Table 1:**
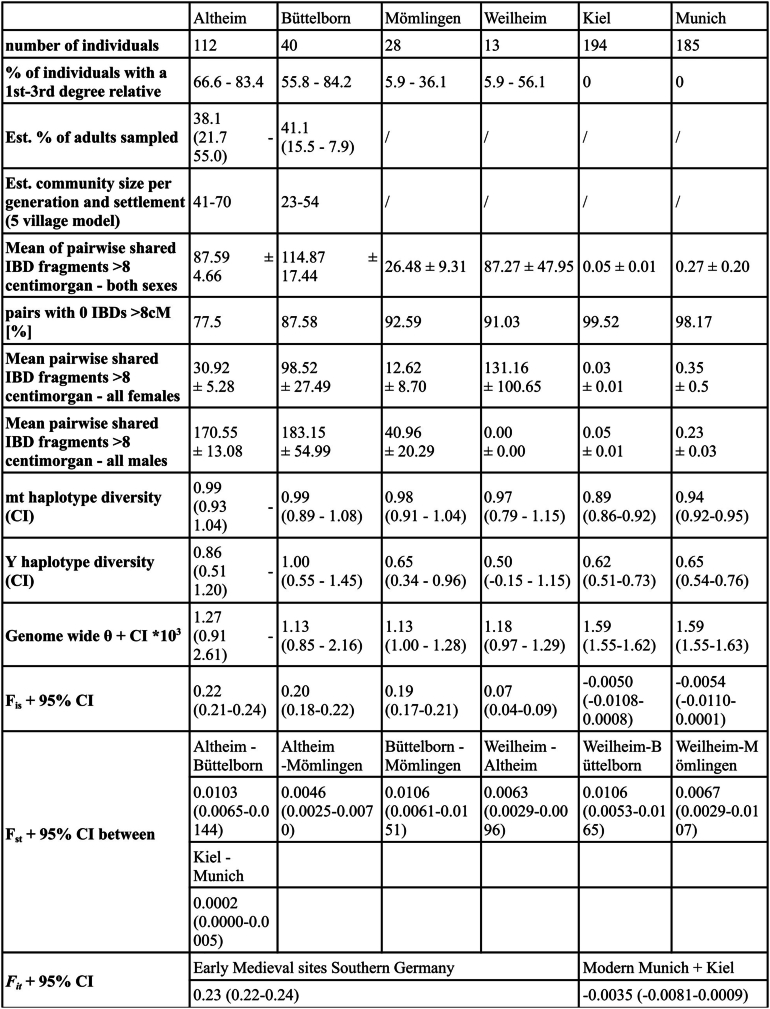
Population genetic statistics

Calculated from unrelated individuals at the four main Early Medieval sites and from modern DZHK genomes from Kiel and Munich, Germany. Only transversions were used for estimates on autosomal markers, except for theta (θ), which was estimated on a set of “neutral” regions defined in^80^.

## Data Availability

All newly reported genome sequences have been deposited in the European Nucleotide Archive, under the accession PRJEB87112. The human reference genome (hg19) used during alignment is available via the 1000 Genomes Project^73^ repository (https://ftp.1000genomes.ebi.ac.uk/vol1/ftp/technical/reference/phase2_reference_assembly_sequence/). Genome sequences for the 379 modern-day individuals from Germany are available upon request at the DZHK (https://dzhk.de/en/dzhk-heart-bank/data-and-biospecimens/dzhkomics-resource). The 1000 Genomes Project phase 3 reference panel^73^ used for imputation and Relate can be downloaded from https://ftp.1000genomes.ebi.ac.uk/vol1/ftp/release/20130502/. Previously published genotype data for present-day and ancient individuals is available through the Allen Ancient DNA Resource at the Harvard dataverse (https://dataverse.harvard.edu/dataset.xhtml?persistentId=doi:10.7910/DVN/FFIDCW).

## Code availability

Chronograph and filia are available as git repositories at https://bitbucket.org/wegmannlab/chronograph and https://bitbucket.org/wegmannlab/filia, respectively. Additional code used in the described analyses can be found at Zenodo (10.5281/zenodo.17192653)^85^.
