# Supplementary material for: Demography and life histories across the Roman frontier in Germany 400–700 ce
Source: Nature. 2026 Apr 29;654(8120):984–93. doi: 10.1038/s41586-026-10437-3 (PMC13293882; doi:10.1038/s41586-026-10437-3)
Supplement: Supplementary file 1 — This file contains Supplementary Information [file 41586_2026_10437_MOESM1_ESM.pdf]

nature portfolio

https://doi.org/10.1038/s41586-026-10437-3

Supplementary information

Demography and life histories across the Roman frontier in Germany 400–700 CE

In the format provided by the authors and unedited

Demography and Life Histories across the Roman Frontier in Germany 400-700 CE

Jens Blöcher, Leonardo Vallini, Maren Velte, Raphael Eckel, Léa Guyon, Laura Winkelbach, Mark G. Thomas, Nadia Gharehbaghi, Cassandra T. Mitchell, Jonas Schümann, Sophie Köhler, Elsa Seyr, Katharina Krichel, Sophie Rau, Jana Hirsch, Jana Duras, Paul Cloarec-Pioffet, Andreas Füglistaler, Kristin Klement, Miriam Wilkenhöner, Lisa Vetterdietz, Francesca Gentilin, Melany Müller, Anna-Lena Mücke, Nicoletta Zedda, Youssef Tawfik, Eveline Saal, George McGlynn, Barbara Bramanti, Jörg Orschiedt, Regina Molitor, Barbara Fliß, Ines Spazier, David Shankland, Claus Vetterling, Kurt Karpf, Vera Planert, Stefan Hölzl, Silvia Codreanu-Windauer, Dieter Quast, Ilija Mikić, Sven Fiedler, Bernd Päffgen, Maxime Brami, Thomas Richter, Raphaëlle Chaix, Susanne Brather-Walter, Peter Steffens, Markus Marquart, Thomas Becker, Jochen Haberstroh, Mischa Meier, Sebastian Schmidt-Hofner, Sebastian Brather, Michaela Harbeck, Steffen Patzold, Daniel Wegmann, Joachim Burger

SUPPLEMENTARY INFORMATION

S1. Description of archaeological sites....3
Essenbach-Altheim, Andreasweg (N=112)....3
Büttelborn (N=40)....6
Mömlingen (N=28)....8
Weilheim (N=13)....10
Eltville (N=8)....12
Burgweinting (N=7)....14
Wölfersheim-Berstadt "Über den Holdergärten" (N=5)....17
Ergoldsbach - "Dörnbacher Feld" (N=4)....20
Michaelsbuch (N=2)....22
Mattsies (N=1)....24
Zschernitzsch (N=1)....26
Straubing Azlburg (N=17)....28
Pförring, Eichstätt District (N=2)....30
Kemathen, Eichstätt District (N=1)....32
Viminacium, Serbia (N=10), Late Antiquity and Migration Period....33
Argamum, Romania (N=3), Late Antiquity....36
Molzbichl, Austria (N=3), Late Early Middle Ages....39
Spina, Italy (N=2), Iron Age Etruscan site....41
Doliche, Turkey (N=1), Antiquity....43
S2. Strontium isotope analysis of Altheim-Essenbach....45
S3. Inferring life histories on pedigrees....50
S4. Whole Genome Data Production....75
Sample preparation....75

DNA extraction........75
DNA library preparation and whole genome sequencing........75
S5. Bioinformatic Processing........76
Variant detection........77
Imputation of ancient genomes........77
S6. Uniparental markers, genetic sex determination and contamination estimation........78
Haplogroup diversity........78
Y haplogroup frequencies........80
S7. Principal Component Analysis........81
S8. Ancestry Modeling........91
Relate ancestry painting (distal sources)........91
Relate ancestry painting (local sources)........100
ChromoPainter2/sourcefind........106
PANE........114
S9. High genetic diversity in border regions........117
S10. Analyses of shared fragments identical by descent (IBD)........119
Modern genomes........119
Ancient genomes: Intra-site comparisons........119
Ancient genomes: Inter-site comparisons........124
S11. Runs of homozygosity........136
S12. Genetic relatedness estimation and Pedigree reconstruction........138
Estimating biological relatedness with KIN and READ2........138
Pedigree reconstructions........138
Long distance connections........141
Estimating sampling completeness........143
S13. Refining ancestry estimates on pedigrees........145
The Model........145
Inference........146
S14. The origin of the modern Central European kinship system........147
Association of inferred genetic relatedness and burial locations........147
Ancestry and proximity of related individuals........150
Long lasting family traditions........150
Pedigree continuity........151
Evolution of the Altheim grave field........153
S15. Identifying a set of unrelated individuals for population genetic analyses........155
S16. Estimating community sizes for Altheim and Büttelborn........156
S17. Population genetic estimates of diversity........157
Estimation of deviation from Hardy-Weinberg-Equilibrium (Fit)........157
Estimation of pairwise differences (Dxy, π)........157
References........159

S1. Description of archaeological sites

Essenbach-Altheim, Andreasweg (N=112)

Sebastian Brather, Susanne Brather-Walter, Steffen Patzold, Maren Velte

The "Essenbach-Altheim, Andreasweg" (hereafter *Altheim*) excavation site is a row-grave cemetery dating from the 5th to 8th centuries, located in Altheim-Essenbach in the Lower Bavarian district of Landshut, Germany. In its vicinity, several additional Merovingian-period burial grounds have been uncovered, all situated on a terrace overlooking the Isar Valley. To the south of the cemetery, traces of a Roman villa from the 1st to 3rd centuries have been found, close to an ancient Roman road along the Isar River. Approximately fifty meters west of the cemetery stands the Church of St. Andreas, a Late Medieval structure incorporating both Romanesque and Gothic elements. The burial ground was first identified in 1953 and subsequently excavated between 1989 and 1992 in response to construction activities. It is divided into four excavation sectors, the largest of which lies in the northern portion of the cemetery and covers roughly 5,000 m^2. The chronological framework and detailed archaeological analysis of the site were established by Johannes Sebrich (2019) [11] (according to Koch (2001) [85]) and later revised by Susanne Brather-Walter and Sebastian Brather. Over 400 graves have been documented, with 95% concentrated in the main area. However, only part of the cemetery has been explored, and it is estimated that it contains up to 900 burials in total. Grave density varies widely, ranging from closely packed burials to areas with larger open spaces.

The grave goods recovered include jewelry (such as bow brooches, S- and bird-shaped brooches, earrings, needles, and beads), tools (combs, spindle whorls, knives), dress accessories (belt buckles, fittings, and calf binding sets), weapons (swords, seaxes, lances, and arrows), and vessels [11]. Approximately 85% of the graves were reopened during the Early Middle Ages, which complicates chronological and social analyses due to the removal of objects. Recent studies suggest that these reopenings were not acts of grave robbery but rather part of burial rituals and social practices of the time.

Osteological standard findings for all individuals were obtained following the guidelines of the *Staatssammlung für Anthropologie München* (SAM, state collection for Anthropology in Munich) [86] by Elsa Seyr and Maren Velte. The skeletal samples for genomic analysis were provided by the SNSB, SAM. Petrous bone samples were primarily collected from the western and northern parts of the burial ground. In the western section, the individuals buried date back to the 5th century and are among the earliest in the cemetery. Their dating is largely based on ^{14}C analysis, as these graves contain no grave goods and cannot be dated using other methods. It remains uncertain whether burials began as early as 400 CE. Genetic pedigree information has led to the re-dating of some individuals, initially thought to belong to the early 5th century, to the second half of the 5th century instead. In contrast, the burials in the northern section date to the 6th and 7th centuries.

Genomic data from four individuals were already published by Veeramah *et al*. (2018) [14]. For this study, another 98 genomes from petrous bones were sequenced, resulting in a total of 102 genomes. The mean endogenous DNA content of the samples is 58.76 % (median: 70.28 %), ranging from 7.06 % to 81.22 %. The full genomic sequencing depth ranges from 0.12 X to 8.17 X with an average of 2.74 X (median: 2.67 X).

Further details on isotopic investigations can be found in chapter S2.

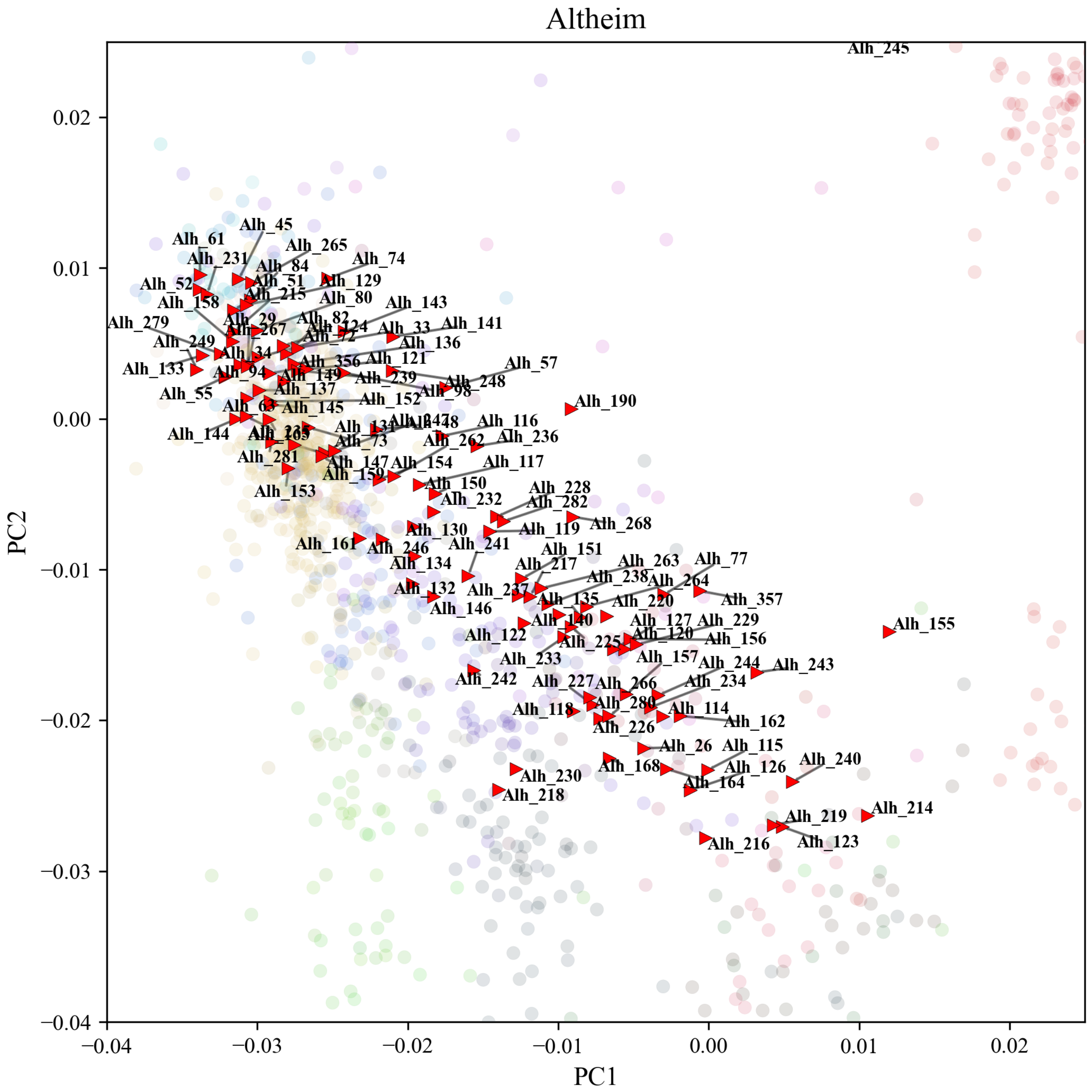

Fig. S1.1: *PCA of Altheim genomes plotted against a Central European pre-Roman/Iron Age reference. Newly sequenced genomes from the Altheim site (individually labeled) are projected onto a reference panel composed predominantly of Central European genomes. The geographic origins of the Iron Age reference genomes are colour-coded, as shown in the map in Fig. S7.4.*

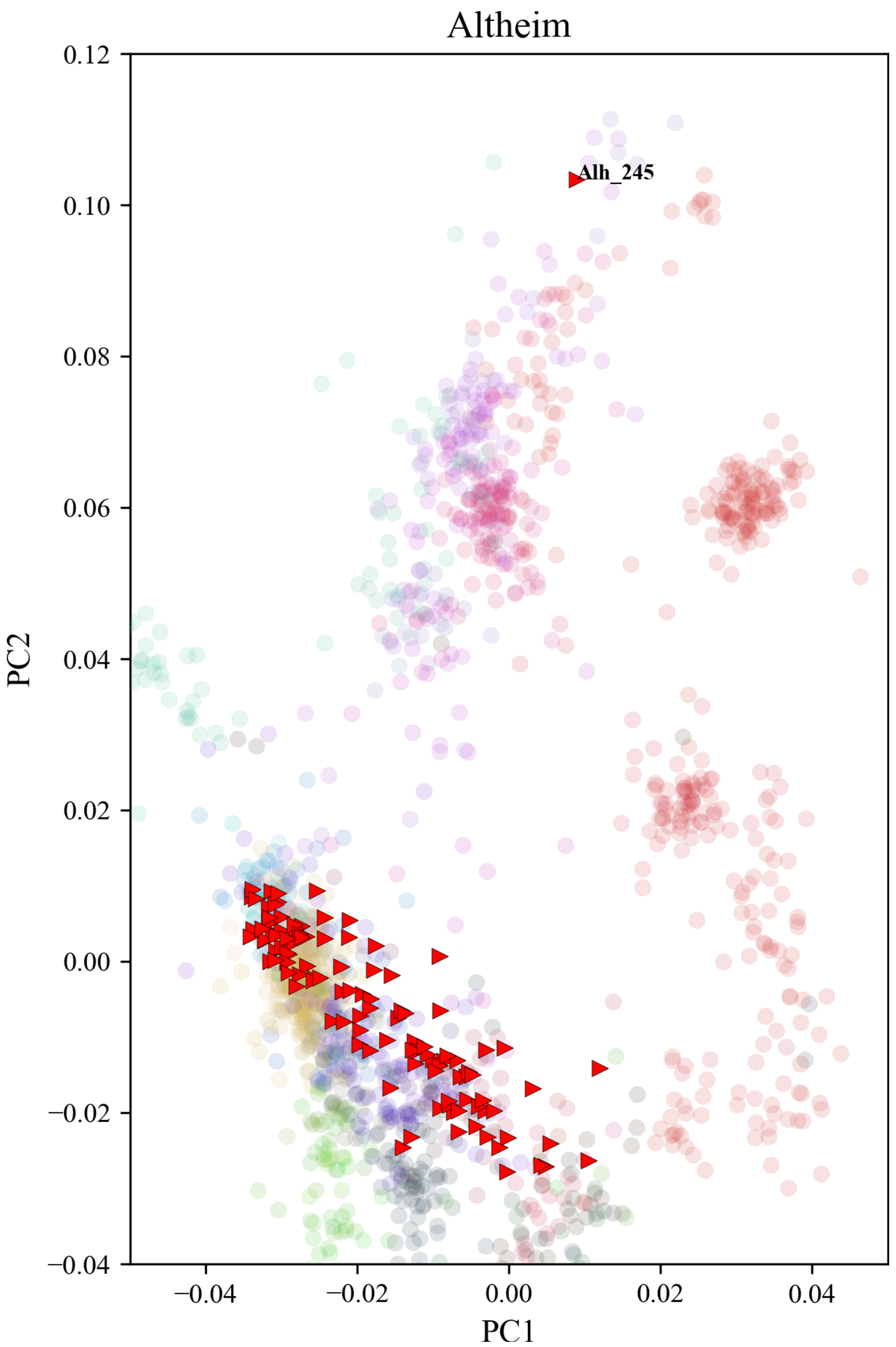

Fig. S1.2: *PCA depicting the full spread of the newly sequenced genomes from the Altheim site. Only individual 245 is labeled, which is found in PCA space close to Iron Age individuals from Eastern Asia. The geographical origin of the Iron Age reference genomes is color-coded, as shown in the map in Fig. S7.4.*

Büttelborn (N=40)

Thomas Becker, Peter Steffens

Individuals from 44 graves were examined from the excavation site "Winkelseite", an Early Medieval row-grave-field near Büttelborn. Büttelborn is a municipality in the Groß-Gerau district in Hesse and is located close to the border (Limes) of the former Roman Empire. The samples were provided by the Hesse State Office for Monument Preservation (hessenARCHÄOLOGIE).
The grave site was discovered in 1900, and 43 graves were documented in the first excavations in 1903, 1904 and 1906. Six more campaigns were carried out in 1998-2000, 2002, 2007 and 2013 covering more than 7000 m². In total, 455 graves were discovered, mostly individual burials in supine positions with West to East orientation with varying depths and furnishings [87–89]. Despite a very high grave robbery rate of approximately 90%, the archaeological dating of grave goods suggests that the sampled graves date to the 6th-8th centuries A.D. Six individuals were selected for radiocarbon (^{14}C) dating in a way that allowed the dates of other family tree members to be estimated. The ^{14}C dates are listed below in table S1.1.
There are a few distinct differences between the estimated birth year and the archaeological dating of the graves (for example Büttelborn Grave 282), which can not be reconciled at the moment. Based on the overview of the grave goods excavated in Büttelborn the occupation time of the graveyard dates from around 500 up to the beginning of the 8th century.
Sampling of the petrous bones for ^{14}C dating was conducted by Laura Winkelbach and the ^{14}C dating was done by the Curt-Engelhorn-Zentrum Archäometrie gGmbH (www.ceza.de). 50 skeletons were examined anthropologically and forensically by Sophie Rau and Barbara Fliß with a focus on the determination of age at death, sex, body height, and pathological changes.
Sampling and genome generation were conducted by Katharina Krichel, Jana Hirsch, Laura Winkelbach, and Cassandra Mitchell. 44 samples were chosen for genomic analysis based on their chronological placement. Out of these, 40 genomes were created. Four samples, Btb219, Btb110, Btb111b, and Btb66, were excluded due to a low molecule number. The endogenous DNA content ranges from 23.01 % to 80.29 %, with an average of 66.45 % (median: 73.30 %). The mean sequencing depth is 1.55 X (median: 1.4 X) with values ranging from 0.70 X to 8.00 X.

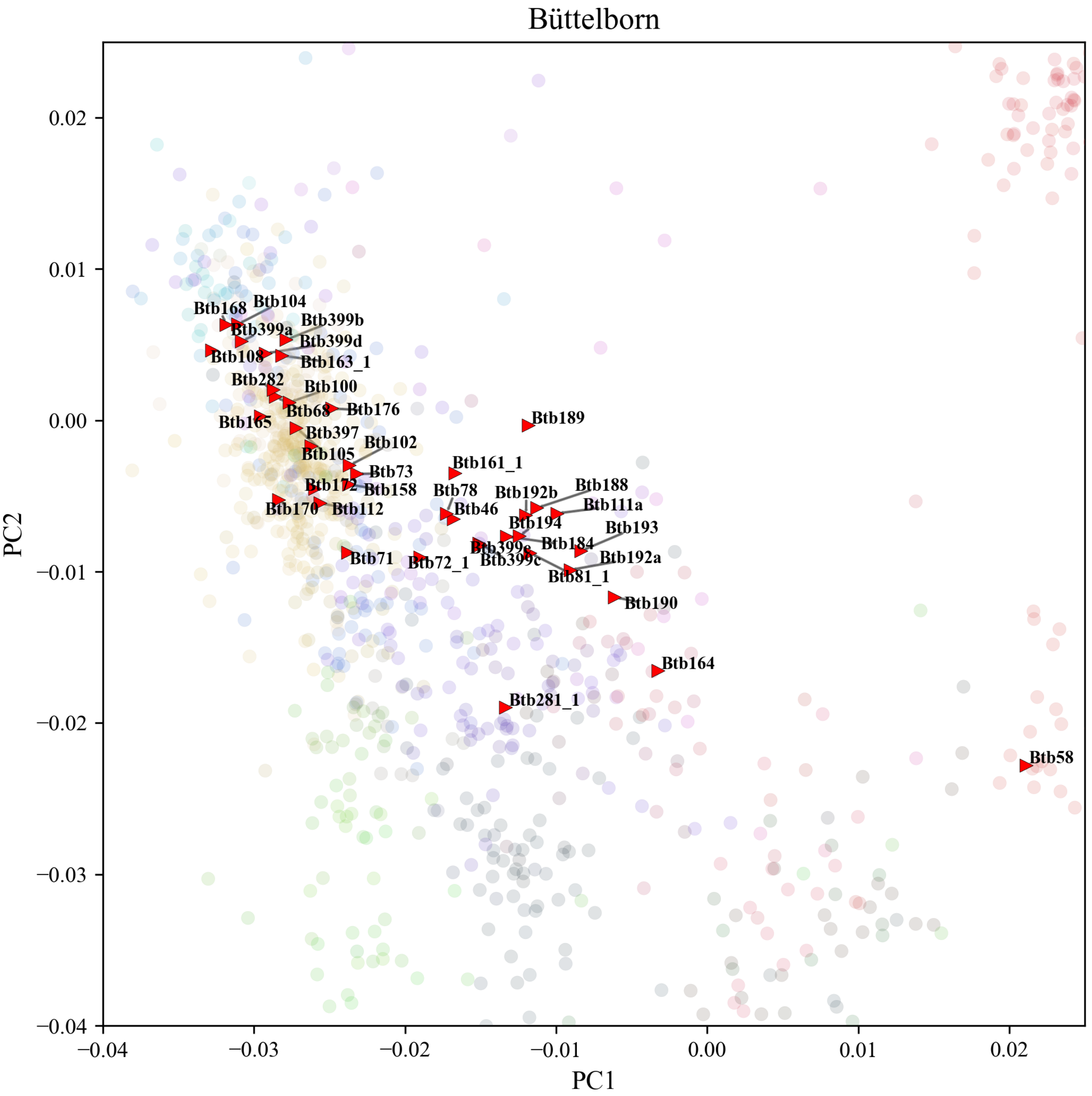

Fig. S1.3:*PCA of Büttelborn genomes plotted against a Central European pre-Roman/Iron Age reference. Newly sequenced genomes from the Büttelborn site (individually labeled) are projected onto a reference panel composed predominantly of Central European genomes. The geographic origins of the Iron Age reference genomes are colour-coded, as shown in the map in Fig. S7.4.*

Table S1.1: *C14 Dating of 6 bone samples from Büttelborn. The datings were conducted in 2024 at the Curt-Engelhorn-Center for Archaeometry gGmbH.*

					Kalibrierte Alter					
Lab Nr MAMS	Sample Name	^{14}C Age [yr BP]	±	δ13C AMS [‰]	Wahrscheinlichkeit 68%	Wahrscheinlichkeit 95%	C:N	C [%]	Collagen [%]	Material
68392	Btb_73	1519	20	-21.5	cal AD 548-583	cal AD 485-603	3.3	49.7	8.8	bone
68393	Btb_108	1486	17	-20.8	cal AD 572-602	cal AD 554-639	3.2	40.4	5.1	bone
68394	Btb_158	1570	18	-22.3	cal AD 436-547	cal AD 431-554	3.2	45.8	16.4	bone
68395	Btb_176	1508	18	-23.5	cal AD 560-591	cal AD 546-602	3.0	37.8	17.0	bone
68396	Btb_188	1305	18	-18.7	cal AD 669-772	cal AD 661-775	3.2	44.3	10.0	bone
68397	Btb_192b	1357	18	-23.5	cal AD 653-667	cal AD 645-758	3.0	37.0	4.3	bone

Mömlingen (N=28)

Markus Marquart

The Early Medieval row-grave-field of Mömlingen, a village in the Miltenberg district in the Bavarian Lower Main region, is located in the former parcel “Hollerstrauch”. It was discovered during railroad constructions in 1911 and 1912. The first at least seven burials were reburied without archaeological examination (page 141, Taf 5.1 and 5.2, as per grave catalogue) [90]. Excavations took place between 1951 and 1980. Out of 48 graves, only four have not been robbed. Archaeological dating of the site based on grave goods places the burials in the sixth to eighth century A.D. The grave goods include pearls like amber and glass pearls which were found in eight graves [91].

The burials were aligned in West - East orientation with the exception of three graves 9, 27, and 40 with South - North orientation. These graves were laid out in a more ornate way as wooden chambers and are assumed to have been rich in furnishing. This is indicated by the pattern of robbing which suggests the men in grave 9 and 27 to have borne weapons [92].

Osteological analyses were conducted by Franziska Immler of AnthroArch GbR, commissioned by Johannes Gutenberg University. 31 samples were provided for DNA analysis by the Aschaffenburg City-Museums. They consisted of 28 petrous bones and three navicular bones resulting in the generation of 28 genomes as the DNA content of the navicular bones was not sufficient for sequencing. The mean endogenous DNA content is 68,01 %, ranging from 40.55 % to 80.32 % with the median at 75.16 %. The obtained sequencing depth ranges from 1.54 X to 4 X with the mean of 2.99 X and the median at 2.87 X.

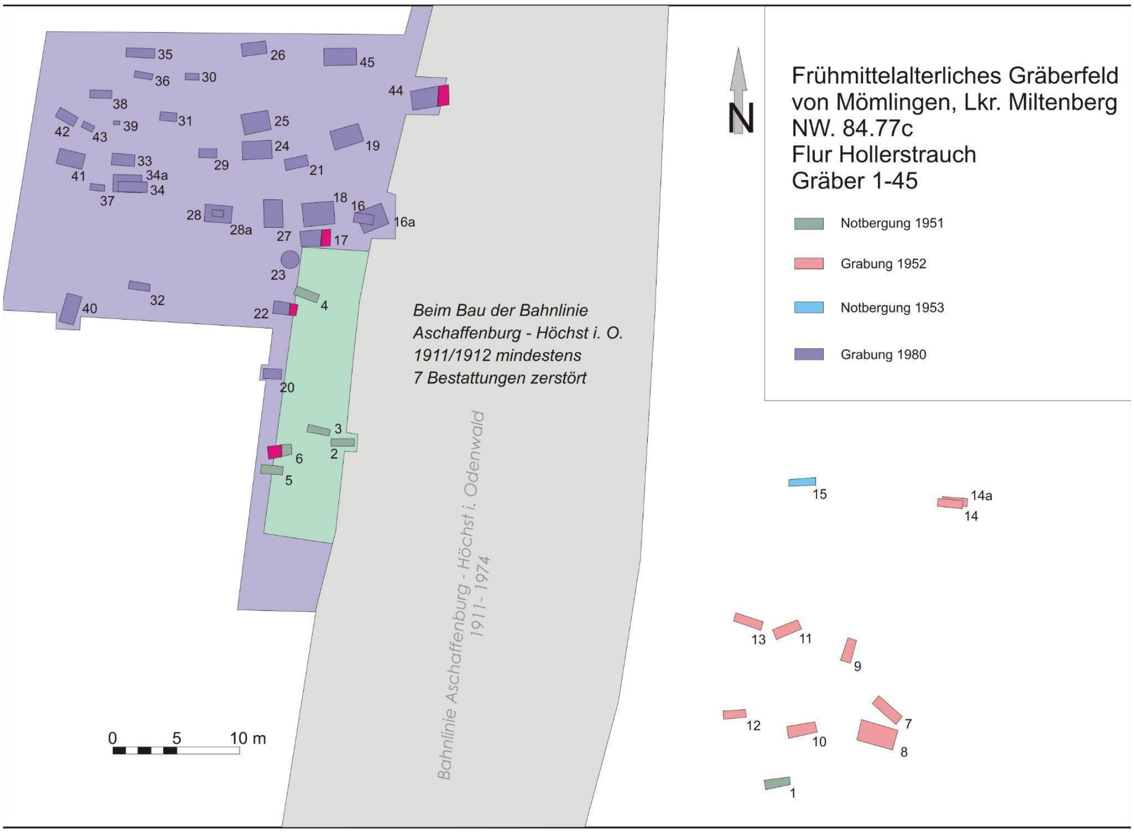

Fig. S1.4: *Plan of the Early Medieval Cemetery of Mömlingen; grave plan courtesy of Markus Marquart.*

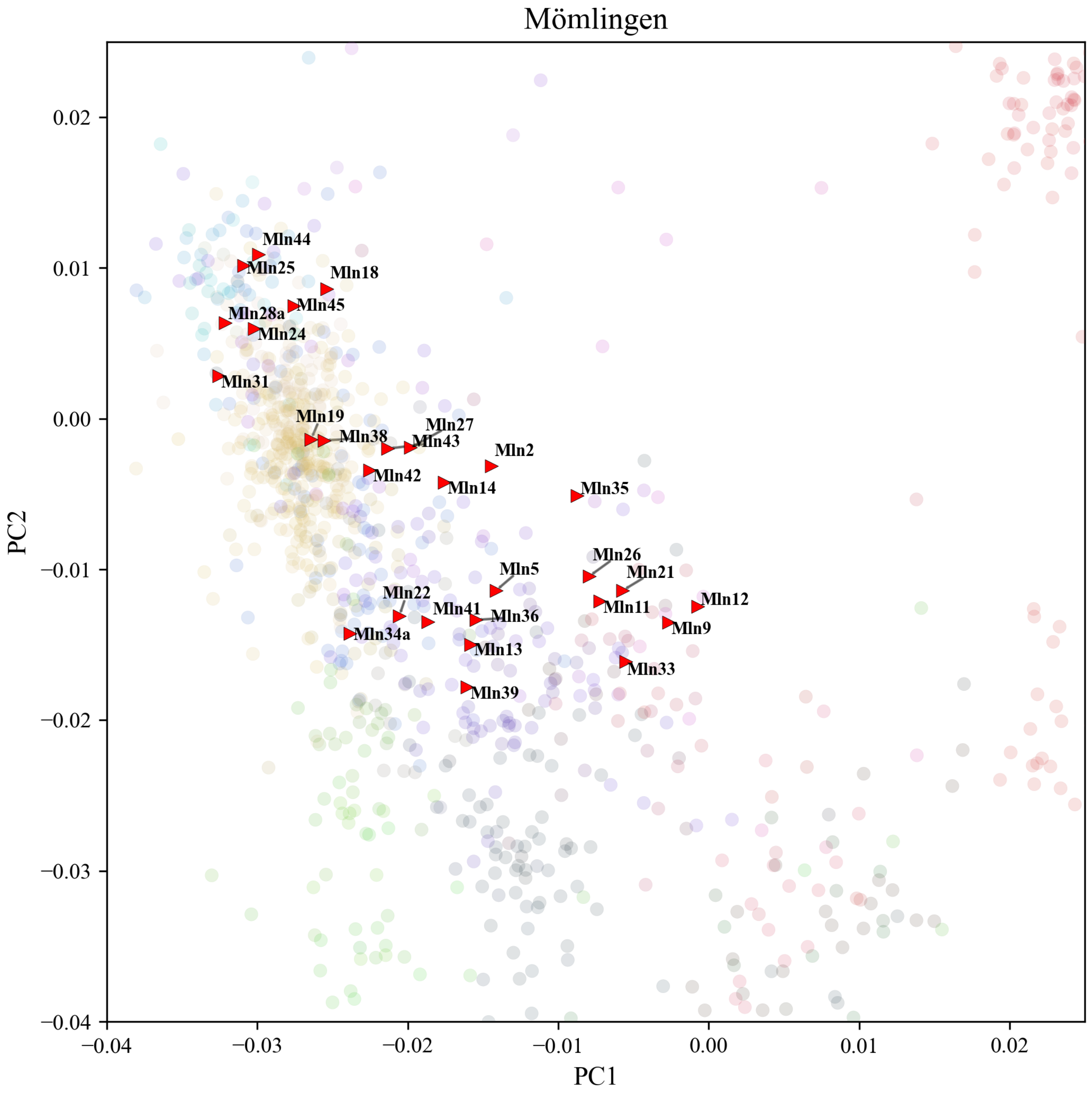

Fig. S1.5: *PCA of Mömlingen genomes plotted against a Central European pre-Roman/Iron Age reference. Newly sequenced genomes from the Mömlingen site (individually labeled) are projected onto a reference panel composed predominantly of Central European genomes. The geographic origins of the Iron Age reference genomes are colour-coded, as shown in the map in Fig. S7.4.*

Weilheim (N=13)
Jochen Haberstroh

The Early Medieval cemetery of Weilheim “Am Meisteranger” is located close to the city center of Weilheim and to the ancient Roman road from Augsburg to Brenner. in the southwest of the street *Am Meisteranger*. Excavations took place in September and October 2019. During these, 15 burials were discovered. According to the excavation report by Norbert Piller (*Planateam Archäologie, Wasserburg),* they consisted of individual burials in supine position which were mostly aligned in West—East -orientation [93]. The grave site is presumed to extend further to the east and south; no more burials could be identified in the north. The skeletons were anthropologically examined in situ by Eva Kropf (Anthro Analytics)and samples were taken from rib fragments for ^{14}C dating. Further in-depth anthropological examinations were conducted by Dr. Nadine Carlichi-Witjes and Dr. Kristin v. Heyking (AnthroArch) in February of 2023. Artificial cranial deformations were found in three individuals with the report numbers 2, 4, and 59 [94]. The graves show no signs of secondary opening or grave robbery. Grave goods include brooches from three graves with the report numbers 2, 5, and 11, belt buckles (numbers 21, 59), bone combs (numbers 9, 10, 21), and a Roman coin from grave 9.
Especially remarkable is the belt from the women's grave Bef. 59, adorned with cross-shaped inlays. The decoration has only a few parallels on belt buckles from Globasnitz (Kärnten) and Sirmione (Lake Garda) [95,96]. The fitting of a Late Roman belt set that was repurposed as a brooch by the woman in grave 11. No weapons were found in the men's graves. The distribution of key finds and burial practices suggests connections to the middle Danube region. All burials were 14C dated. In combination with the grave goods, this indicates an occupation period between approximately 480 and 530 CE. Notably, the furnishings of the women's graves exhibit significant signs of wear and repair at the time of burial.
15 samples were provided by the *SNSB, Staatssammlung für Anthropologie München* (SAM, state collection for anthropology in Munich) which consisted of 13 petrous bones and two navicular bones. Sampling took place in January 2024 in the Dornach depot near Munich. The navicular bones (Wh12 and Wh7) were excluded from sequencing due to the library preparation not being successful.
We generated a total of 13 genomes. The mean endogenous DNA content of the samples is 65.80 %, ranging from 26.98 % to 80.24 % with a median at 69.20 %. The sequencing depth ranges from 1.66 X to 6.33 X with a mean of 4.31 X and a median at 4.27 X.

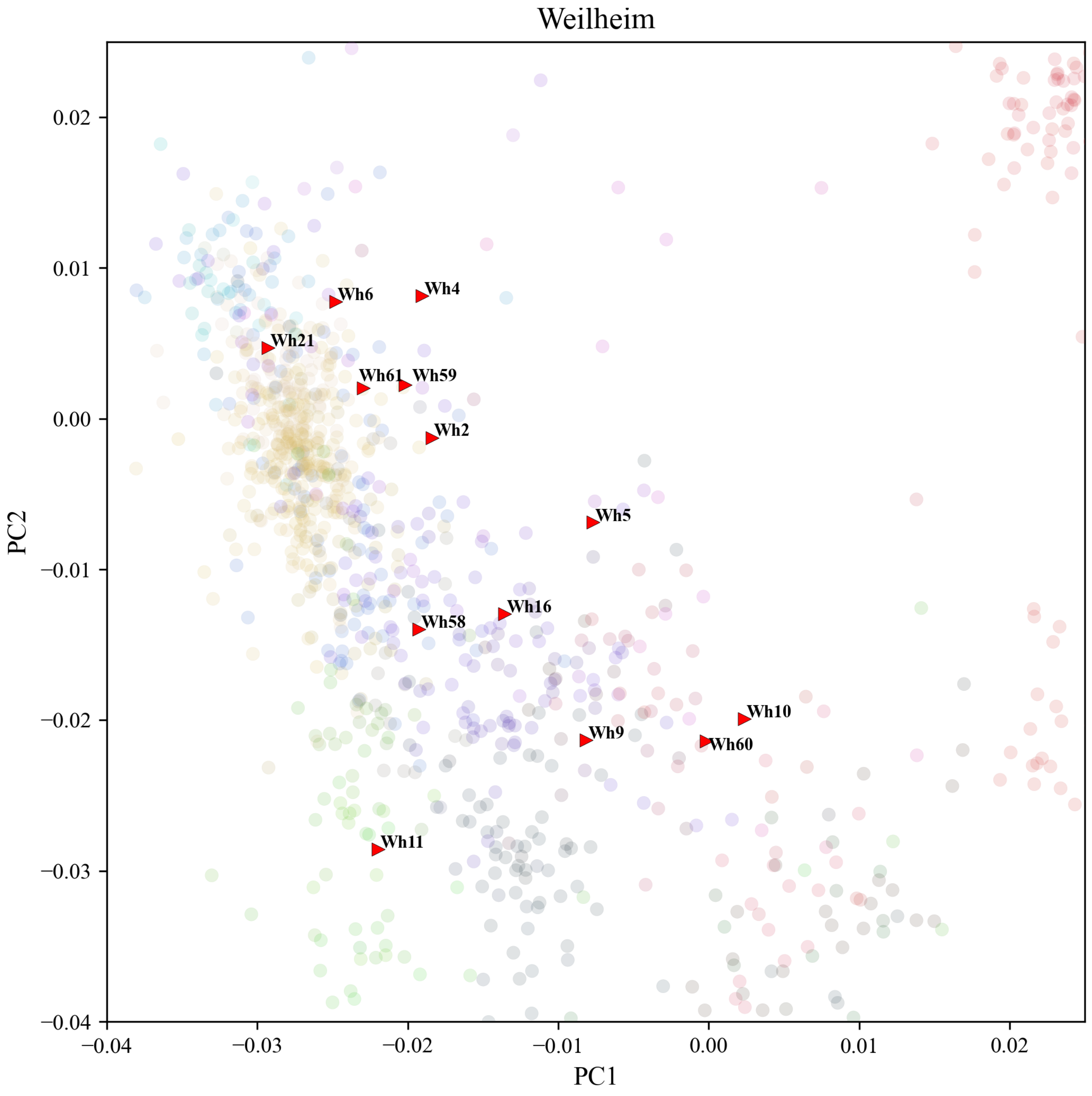

Fig. S1.6: *PCA of Weilheim genomes plotted against a Central European pre-Roman/Iron Age reference. Newly sequenced genomes from the Weilheim site (individually labeled) are projected onto a reference panel composed predominantly of Central European genomes. The geographic origins of the Iron Age reference genomes are colour-coded, as shown in the map in Fig. S7.4.*

Eltville (N=8)
Thomas Becker

The Eltville site is situated in western Germany, within the Rheingau - Taunus district of Hesse, along the Rhine River. It dates back to the Early Middle Ages, specifically the Merovingian period. Eltville's necropolis is one of the largest Early Medieval burial sites in this region [97] and the largest Merovingian cemetery in Hesse, both in terms of the number of graves and its size [98]. The graveyard was discovered in 1940, during construction work for a machine factory in the eastern part of the burial ground. Excavations conducted in the 1950s, 1960s and mainly in the 1970s uncovered over 600 graves [97], and additional digs by the Archaeological unit of the Hessian State Office for Monuments and Sites in 2015 and 2016 revealed 297 more graves [98]. To date, at least 943 graves have been identified. The cemetery was established at the end of the 5th century (around 470/480 AD) and burials took place up to the mid of the 8th century (around 750 AD). The preservation of these graves varies, with some being well-preserved with extensive grave goods, while the majority is heavily disturbed. Most individuals were buried in an extended supine position, with orientations ranging from West - East to SouthWest - NorthEast. During the 2015-2016 excavations, some inhumations were identified as secondary burials, leading to the disruption of primary graves in many cases. Only a few secondary burials, mostly those of children, left the primary burials intact. Additionally, 50-60% of the burials excavated in 2015-2016 showed signs of intentional alterations or grave robbery [98]. Anthropological analyses of the remains were carried out by multiple investigators and are concentrated on the graves, which were excavated up to 1976; a summarizing overview can be found in Blaich (2006) [99]. No anthropological analysis has been performed to date on the graves excavated later. However, Blaich notes that significant swaps of the skeletal remains took place in the 1980s, which were not fully reversible, leading to confusions that clarified. The sample analysed in this study was taken from the early and published excavations up to 1976. Dating details of individual graves are based on the results of the work of M.C. Blaich [99].

The endogenous DNA content for the libraries of the eight individuals from Eltville analysed here ranged from 36.98 % to 75.62 % (median: 54.82 %; mean: 54.65 %) and genome sequencing resulted in eight genomes with a sequencing depth of 0.81 X - 13.81 X, (median: 1.05 X; mean: 2.62 X).

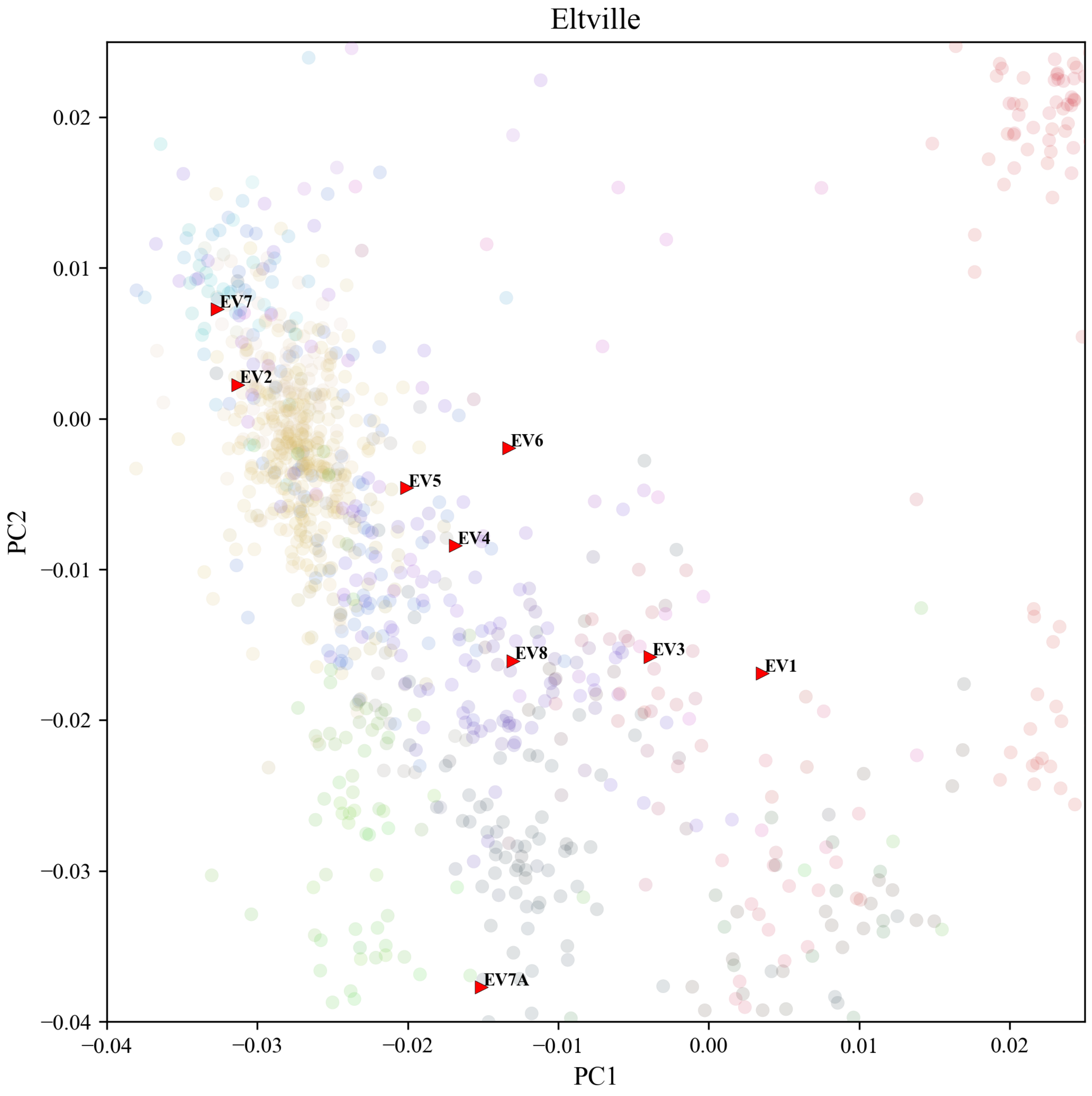

Fig. S1.7: *PCA of Eltville genomes plotted against a Central European pre-Roman/Iron Age reference. Newly sequenced genomes from the Eltville site (individually labeled) are projected onto a reference panel composed predominantly of Central European genomes. The geographic origins of the Iron Age reference genomes are colour-coded, as shown in the map in Fig. S7.4.*

Burgweinting (N=7)
Silvia Codreanu-Windauer

Burgweinting, now incorporated into the town of Regensburg, the capital of the Upper Palatinate in Bavaria, was formerly an independent village. In the Roman period, it lay on the frontier between the province of Raetia II and the "Germania libera," demarcated by the Danube River. By the mid-6th century, the settlement had become part of the Bavarian Duchy. The site encompasses five Early Medieval burial grounds, organized into distinct grave groups; the individuals examined in this study derive from Grave Group C [100], which consists of 11 burials dating to the 6th century. These burials can be further divided into two groups: the older group, dated to 550–560/570, includes graves 2450, 2451, 2452, 2453, and 2454, arranged in two rows and richly furnished. A sixth grave (individual 3749) with poor furnishings was located nearby. The second group, dating from 560/570 to 600, is located southeast in a row and includes graves no. 3701 and 3700, which are richly furnished, while graves 3699 and 3698 have standard furnishings. A fifth grave (individual 3697) was located a few meters away.
The samples were provided by the *SNSB, Staatssammlung für Anthropologie München* (SAM, state collection for anthropology in Munich) in 2020. The skeletons were anthropologically examined by Maren Velte.
Initially, nine bone samples were genetically analyzed but genetic kinship analyses revealed that four samples most likely originated from only two individuals; two individuals would therefore have been sampled twice. This is consistent with the samples likely belonging to one individual being the left and the right *pars petrosa ossis temporalis*; there is hence no indication that identical twins were sampled. Kinship analyses were carried out with three different programs (READ [101], NgsRelate [102] and lcMLkin [103]) and the results (Table S1.2) consistently show that the samples belong to the same individual or are identical twins; genomic data was therefore merged.
The samples' endogenous DNA content ranged from 12.48 % to 69.11 % (median: 44.83 %; mean: 44.67 %) allowing us to generate 7 genomes with sequencing depth from 0.83 X to 3.49 X (median: 1.27 X; mean: 1.73 X).

Table S1.2: *Kinship analysis of double-sampled individuals from Burgweinting. Theta values calculated using ngsRelate and lcMLkin, indicating the degree of relatedness, are consistently above the threshold for first-degree relatives (θ = 0.25).*

Pair	READ	NgsRelate (theta)	lcMLkin (theta)
Bur1 - Bur4	Identical Twins/ Same Individual	0,306415	0,3208
Bur7 - Bur9	Identical Twins/ Same Individual	0,291286	0,2785

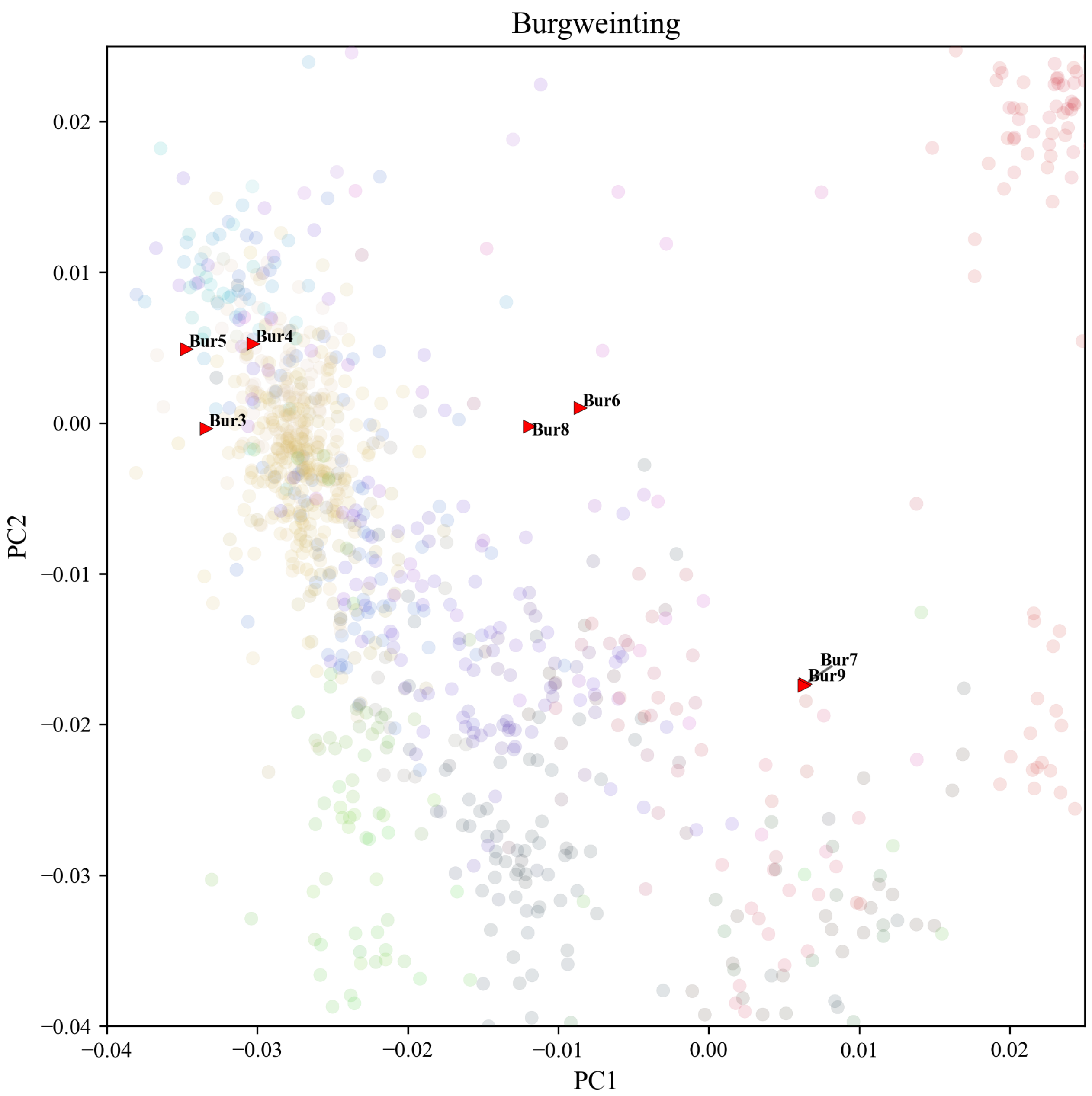

Fig. S1.8: *PCA of Burgweinting genomes plotted against a Central European pre-Roman/Iron Age reference. Newly sequenced genomes from the Burgweinting site (individually labeled) are projected onto a reference panel composed predominantly of Central European genomes. The geographic origins of the Iron Age reference genomes are colour-coded, as shown in the map in Fig. S7.4.*

f_4-statistics (*Roman Southeastern Europe + Iron Age Central Italy*, *Northern Europe*, X, Mbuti) show that the northern group of graves has a higher affinity towards *Northern European* ancestry, while the southern ones are found to be intermediate between Northern and Southern European ancestry (Fig. S1.9). Two individuals, Bur3 and Bur2 (grave numbers 2452 and 2451) are mother and daughter. Furthermore we identified a sister of Bur3, AED1119 [14], buried in Altenerding, located ~80 km south of Burgweinting.

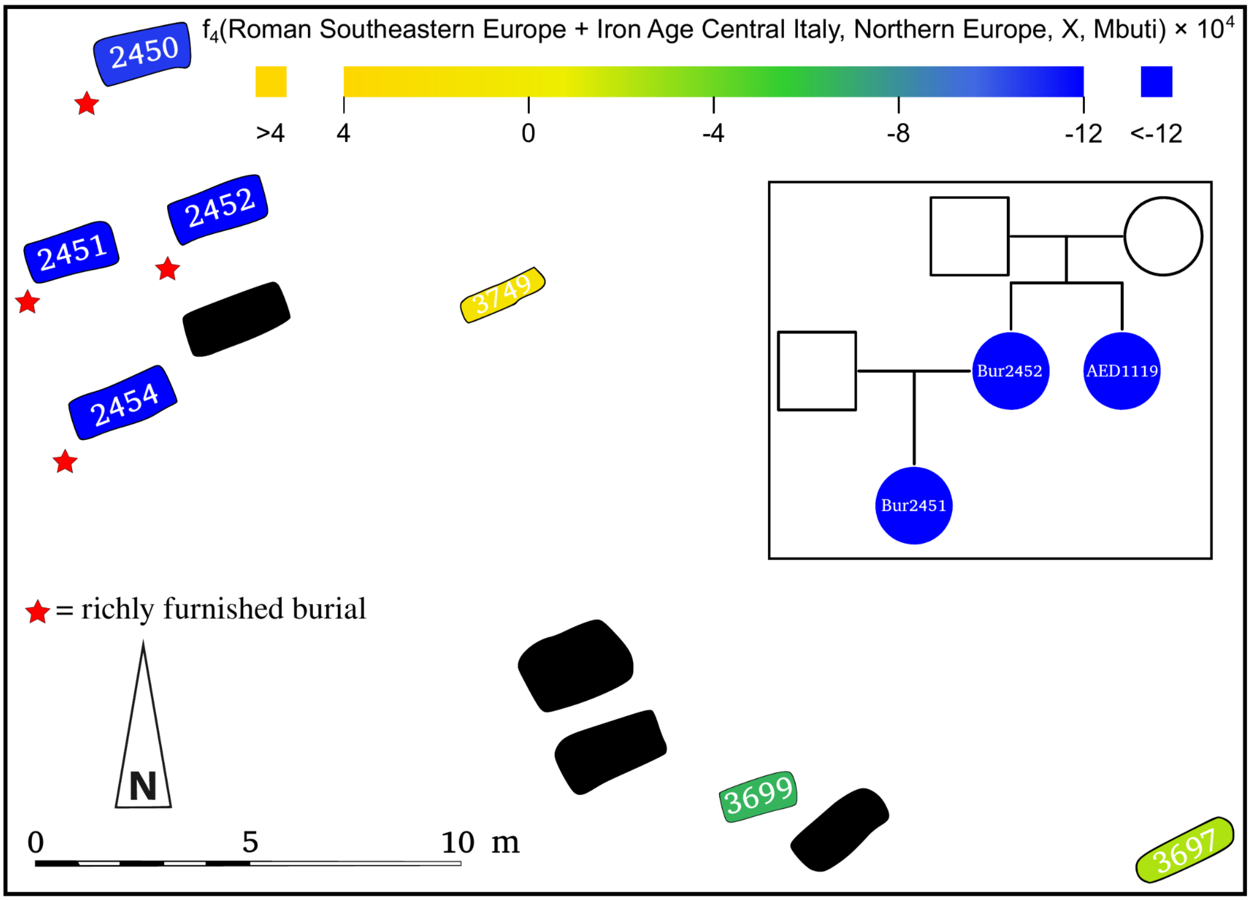

Fig. S1.9: *Grave group C in Burgweinting and kinship reconstruction. Plan of grave group C, showing the sampled burials, alongside the reconstructed pedigree of related individuals. Grave colors reflect f4-statistic values, with blue indicating higher genetic affinity to Northern Europe. Individual AED1119 was sampled from the Altenerding cemetery. Grave plan courtesy of Silvia Codreanu-Windauer.*

Wölfersheim-Berstadt "Über den Holdergärten" (N=5)
Eveline Saal, Jörg Orschiedt

The site of Wölfersheim-Berstadt "Über den Holdergärten," located in the Wetterau district of Hesse, dates to the Merovingian period. With nearly 380 burials, it represents one of the largest known row-grave cemeteries of this period in Hesse.The grave field was discovered in 2006 during development work for the building area "Über den Holdergärten" at the eastern edge of the municipality of Wölfersheim - Berstadt. Excavations were carried out in 2006-2007 by the archaeological monument preservation service of the Wetterau district (Archäologische Denkmalpflege des Wetteraukreises), the municipality Wölfersheim and the Hesse State Office for Monument Preservation (Landesamt für Denkmalpflege Hessen; hessenARCHÄOLOGIE) leading to the grave field being almost completely excavated [104–107].
Based on the grave goods, the site can be classified as a Merovingian burial ground with an occupation period from the late 5th to first half of the 8th century. The cemetery shows a sequence of burials from East to West and the graves have an East - West orientation. The burials took place in wooden burial chambers, which in some cases had a surrounding stone packing, in tree coffins, in canvas sacks or completely without a cover. Preservation and extent of grave robbery varied. By 2007 a total of around 360 individuals in around 380 burials were found on the approximately 2.8 - hectare area. Horse and dog burials as well as circular trenches were also documented. In addition to the Merovingian finds, a few finds at the edge of the development area date to the Iron Age: posts and truncated cone pits, of which some contained animal skeletons and two contained special inhumations [104,105,107].
Men were usually buried with components of their equipment, armor and weapons, while grave goods for women were mainly jewelry such as fibulas, pearls, needles, ear and finger rings, arm rings and chatelaines but also tools for textile production. Other finds include objects made of metal, glass, bones, ceramic and other materials [107,108].
Scientific evaluations carried out by hessenARCHÄOLOGIE (Landesamt für Denkmalpflege Hessen) and KAL (Kommission für Archäologische Landesforschung in Hessen e.V.), are taking place since May 2016, are supplemented by analyses carried out by experts from different fields (such as textile and material sciences and anthropology) and are supposed to be published as a catalogue in the "Materialien zur Vor- und Frühgeschichte von Hessen" series [106–108].
Five individuals were selected for ancient DNA analysis, all of whom were archaeologically dated to the first half of the 6th century, were buried in the oldest part of the cemetery and had very heterogeneous grave goods (Table S1.3). This sample consisted of four women and one man, with sex being determined archaeologically via the grave goods (Table S1.3) and being confirmed by genetic sex determination.
The endogenous DNA content of the five samples analyzed here ranged from 13.43 % to 40.42 % (median: 14.02 %; mean: 23.09 %) and we produced five genomes with 0.46 X to 0.94 X sequencing depth (median: 0.66 X; mean: 0.65 X).

Table S1.3: *Additional archaeological information on individuals from Wölfersheim-Berstadt "Über den Holdergärten." Supplementary details for the individuals analyzed in this study.*

Lab ID	Grave goods	archaeological dating	archaeological sex	Notes
W67	Sax, axe, arrowheads, jug, 4 coins - including a coin of the Burgundian king Gundobad (tpq 516)	1st half of 6th century	♂	Skull crushed, bones moderately preserved
W117	2 almandine disc brooches, 2 bow brooches, iron pin, beads, jug, biconical pot	1st half of 6th century; SD5 (530 - 555)	♀	Skull well preserved
W121	2 almandine disc brooches, 2 bow brooches, iron pin, polyhedron capsule earrings, beads, 4 coins, including one from eastern Rome (tpq 491 - 518), Roman glass bottle	1st half of 6th century; SD5 (530 - 555)	♀	Skull well preserved
W126	2 bird brooches, needle (Irmlaut type), beads, Visigothic belt buckle, 2 coins, biconical pot, glass bowl	1st half of 6th century	♀	Skull well preserved
W128	2 almandine brooches, 2 bow brooches, needle, beads, beaker, biconical pot, glass beaker, bronze bead-edged basin	1st half of 6th century; SD4 (510 - 530) - SD5 (530 - 555)	♀	Skull heavily crushed, important grave with separate location in the early part of the cemetery

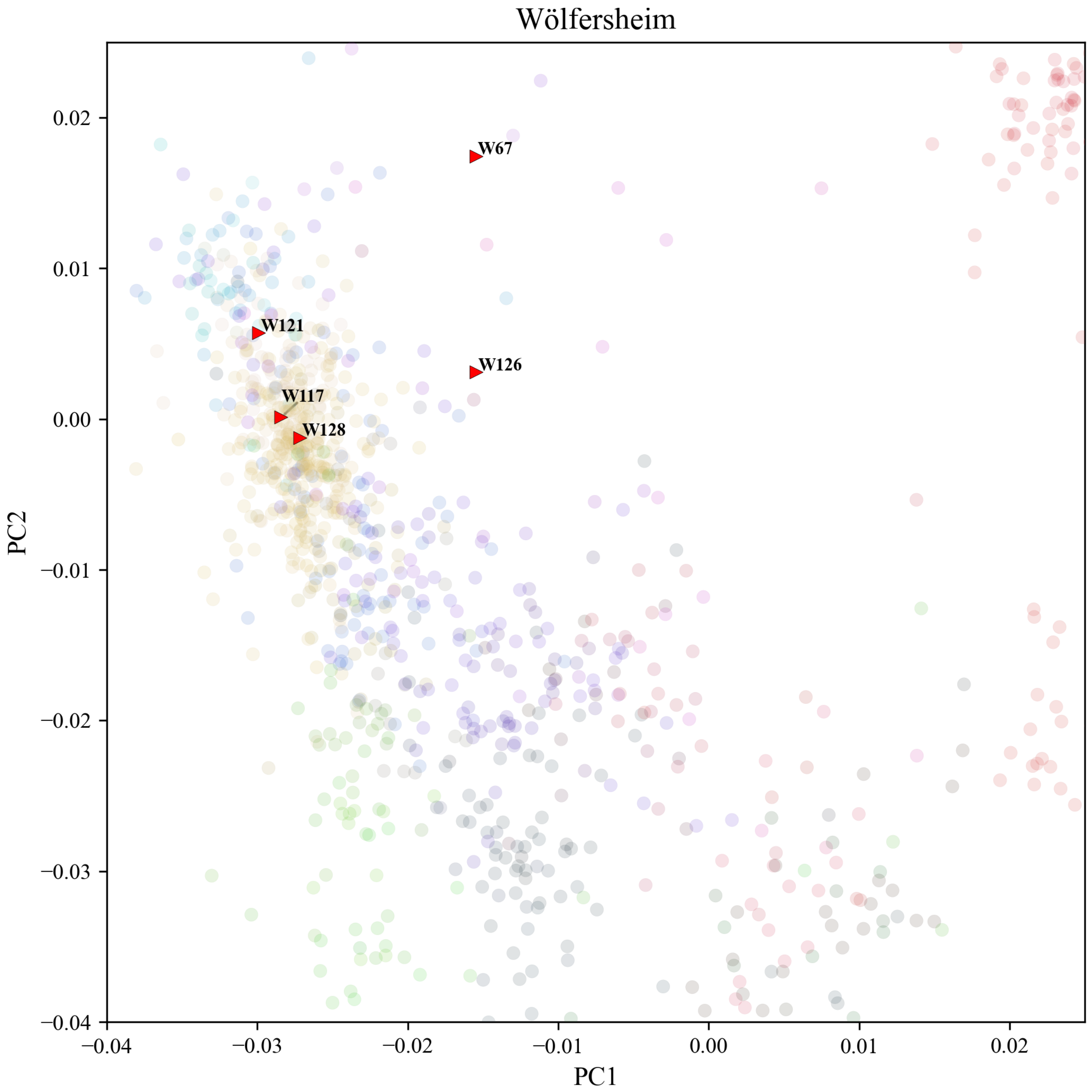

Fig. S1.10: *PCA of Wölfersheim genomes plotted against a Central European pre-Roman/Iron Age reference. Newly sequenced genomes from the Wölfersheim site (individually labeled) are projected onto a reference panel composed predominantly of Central European genomes. The geographic origins of the Iron Age reference genomes are colour-coded, as shown in the map in Fig. S7.4.*

Ergoldsbach - “Dörnbacher Feld” (N=4)
Thomas Richter

As part of a large-scale archaeological excavation in the Ergoldsbach - "Dörnbacher Feld" area, located on the southern edge of Ergoldsbach, a market town in the Lower Bavarian district of Landshut, Germany, two known archaeological sites identified through aerial photographs were explored. These included a settlement from the Münchshöfen culture, an Urnfield period farmstead, and two Early Medieval burial groups, which were partially surveyed in 2018. The excavation (B-2017-059, site "SG u. GG Ergoldsbach Süd", measure number: M-017-1857-2_0) focused on two hilltops (northern and southern areas) that had been identified as rich in features through prior sondages. The discovery of the two Early Medieval burial groups, totaling 20 graves with 22 burials, was an unexpected find, while the older settlement features were anticipated based on previous excavations conducted in 1960.

The grave group in the southern area consists of nine graves with eleven burials, arranged in two rows oriented north-south and a single grave in the west of the western row [109]. In the northern area, the grave group is divided into two smaller groups. One with six graves in three rows, each of them with two graves. In the east of the group there is a single grave. In the other group there had been four graves in two rows. All graves have an east-west orientation and the dead were buried in extended supine position with their arms parallel to their bodies and facing east. Grave goods are generally rare in both groups. They consist of belt buckles, some knives and only a few pieces of jewelry, combs and arrowheads. Although some graves have been robbed, the generally observed small amount of grave goods can be interpreted chronologically. Therefore, the two groups of graves, which have a simultaneous but relatively short occupation period, can overall be dated to the late Merovingian period, more precisely to the period from 670/680 to 720/730 AD [110]. We here produced and analyzed the genomes of four individuals from two graves: find 32 and find 59.

Find 32 is a triple burial in the group of graves in the southern area. The archaeological context of the find indicates that it was a simultaneous burial, in which the individuals who presumably died at the same time were buried close together in a burial chamber made of wood. The few grave goods found with the northernmost individual were a comb, a belt buckle and a knife; the other individual on the outside was also buried with a knife. Multiple burials are a special case in the late Merovingian period. The present triple burial is therefore interpreted as the grave of a family or social unit that died at the same time and possibly belonged to a social elite, with possible causes being accident, illness or - especially in the case of multiple burials of men - combat [110]. However, the anthropological examination of the three individuals revealed no traces of violence [111–113]. Kinship analysis revealed that the three individuals are indeed brothers (see SI10). ^{14}C dating of the remains of the middle of the three deceased (find 32-II) was performed by the Mass Spectrometry Laboratory, Center for Physical Sciences and Technology, in Vilnius (Lab. code: FTMC-GR88-1) and resulted in an age of 651-775 calAD (95.4 % probability), which can be further narrowed down to 651-708 calAD (54.6 % probability) and 726-775 calAD (40.8 % probability) based on the plateau of the calibration curve. From an archaeological point of view, however, the period 726-775 calAD is unlikely, as the use of the Early Medieval row graves in this region ends around 720/730 AD (see for example: [11] for Essenbach-Altheim and [114] for Ergolding-Hagnerleite). The period 651-708 calAD, on the other hand, is consistent with the dating of the two-line triple comb with

triangular handle plate found in the grave, which dates to the late 7th to early 8th century AD [115]. The triple burial therefore probably took place between 651 and 708 calAD.

Find 59 is a female burial in the grave group of the northern area. The grave is the most richly furnished of both Medieval burial groups, indicating that the 20-25/30 year-old woman [116] probably was a member of the local elite, same as the individuals in the triple burial. The grave also has the deepest burial pit, but overall there is no correlation between burial depth and grave goods. The woman was buried in a coffin with numerous grave goods: two silver temple rings of the "Lauterhofen" type, which can be dated to the second third of the 7th to the beginning of the 8th century [117], a comb, a belt buckle, a small iron knife and several rows of beads, which were probably attached to the clothing as jewelry and can be dated to the period between 640 and 700 AD [110].

The endogenous DNA content of the four individuals analysed here ranged from 28.92 % to 73.12 % (median: 65.95 %; mean: 58.49 %) and sequencing resulted in four genomes with a depth of 0.53 X - 10.07 X (median: 3.91 X; mean: 4.61 X).

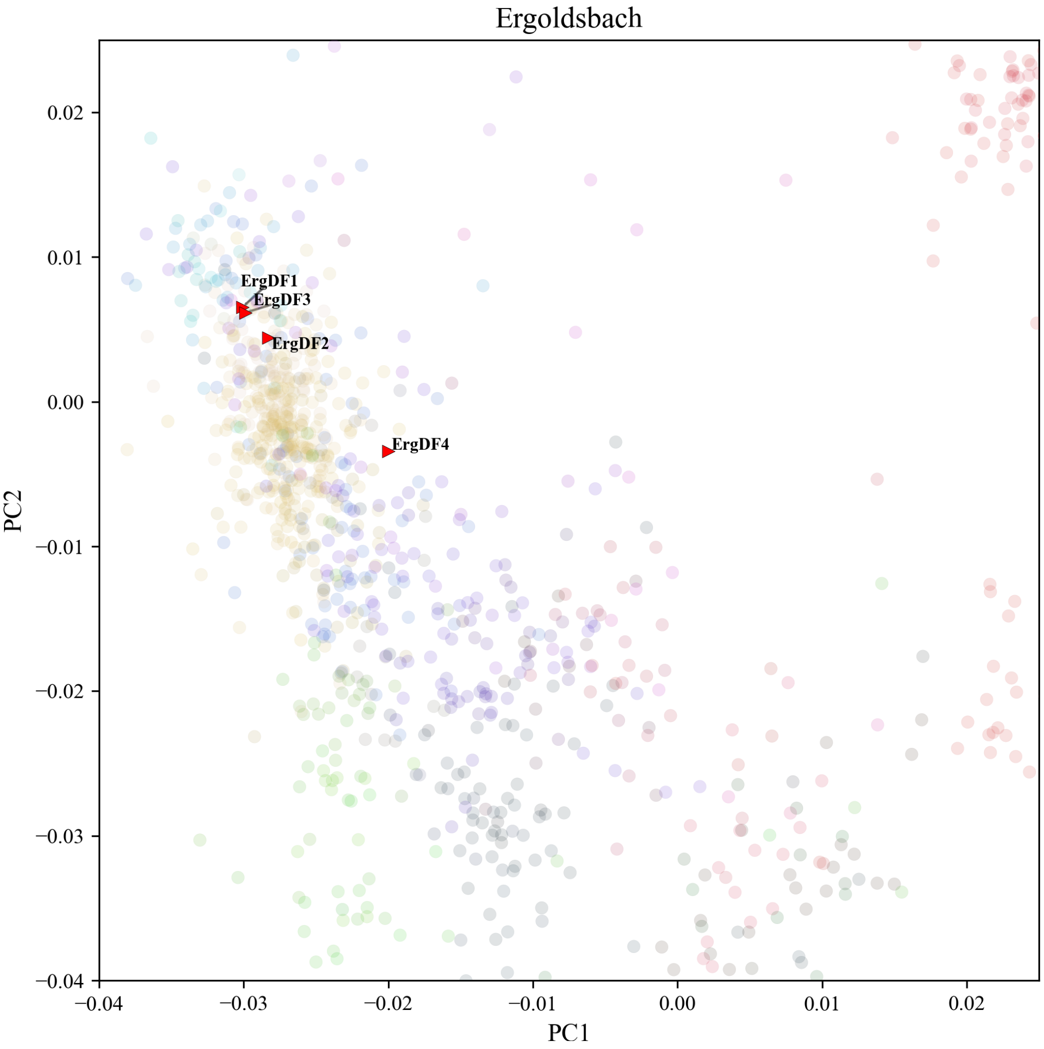

Fig. S1.11: PCA of Ergoldsbach genomes plotted against a Central European pre-Roman/Iron Age reference. Newly sequenced genomes from the Ergoldsbach site (individually labeled) are projected onto a reference panel composed predominantly of Central European genomes. The geographic origins of the Iron Age reference genomes are colour-coded, as shown in the map in Fig. S7.4.

Michaelsbuch (N=2)

Sven Fiedler, Bernd Päffgen

The site of Michaelsbuch, a borough of the municipality Stephansposching, is located in the Lower Bavarian district of Deggendorf. The burial ground was excavated by Stefan Hanöffner, Kreisarchäologie Deggendorf, as part of pre-construction archaeological investigations between November 20th and December 5th, 2014. Michaelsbuch is conveniently located not far from where the Isar flows into the Danube and comprises a small group of 8th century graves belonging to the estate of a historically documented noble family. Gamelbert von Michaelsbuch founded the Metten monastery before 766, where his relative Utto took over as abbot. The scientific processing of the excavation is currently being carried out at the University of Munich.

The burial site consisted of a total of four graves, of which three were single burials (features 11, 20, 21) and one a double burial (feature 16). The graves contained no grave goods and were dated to the Early Middle Ages (8th century, between the second half of the 7th century and the first half of the 9th century). ^{14}C dating was performed by the AMS laboratory Erlangen, Friedrich-Alexander Universität Erlangen-Nürnberg, Germany (Table S1.4 and Supplementary Table 1 for the calibrated ages of the analyzed individuals). The buried individuals were all lying in an extended position on their backs. Their heads were orientated to the west and all individuals were facing east. Graves 20 and 21 show signs of modern-day disturbances.

Postholes were found near the burials, but interpreting their function is difficult due to the limited space of the excavation area. Pottery finds from the various features have been dated to the Early Middle Ages.

The endogenous DNA content of the two analyzed samples was 59.18 % and 69.06 %, respectively, and the two genomes were sequenced to a depth of 0.86 X and 0.75 X.

Table S1.4: *Results of ^{14}C dating of four individuals from Michaelsbuch*

Lab code	archaeological ID	14C lab code	PMC	PMC error	14C date [BP]	Delta C13	Delta N15	C/N
-	Befund-Nr. 11	Erl-20310	84,99	0,42	1306 ± 40	-18,6	9,3	2,82
-	Befund-Nr. 16	Erl-20311	85,64	0,40	1245 ± 37	-18,1	9,0	2,65
Mic1	Befund-Nr. 20	Erl-20312	85,01	0,43	1304 ± 41	-18,2	8,4	2,61
Mic2	Befund-Nr. 21	Erl-20313	85,46	0,43	1262 ± 40	-18,6	9,3	2,71

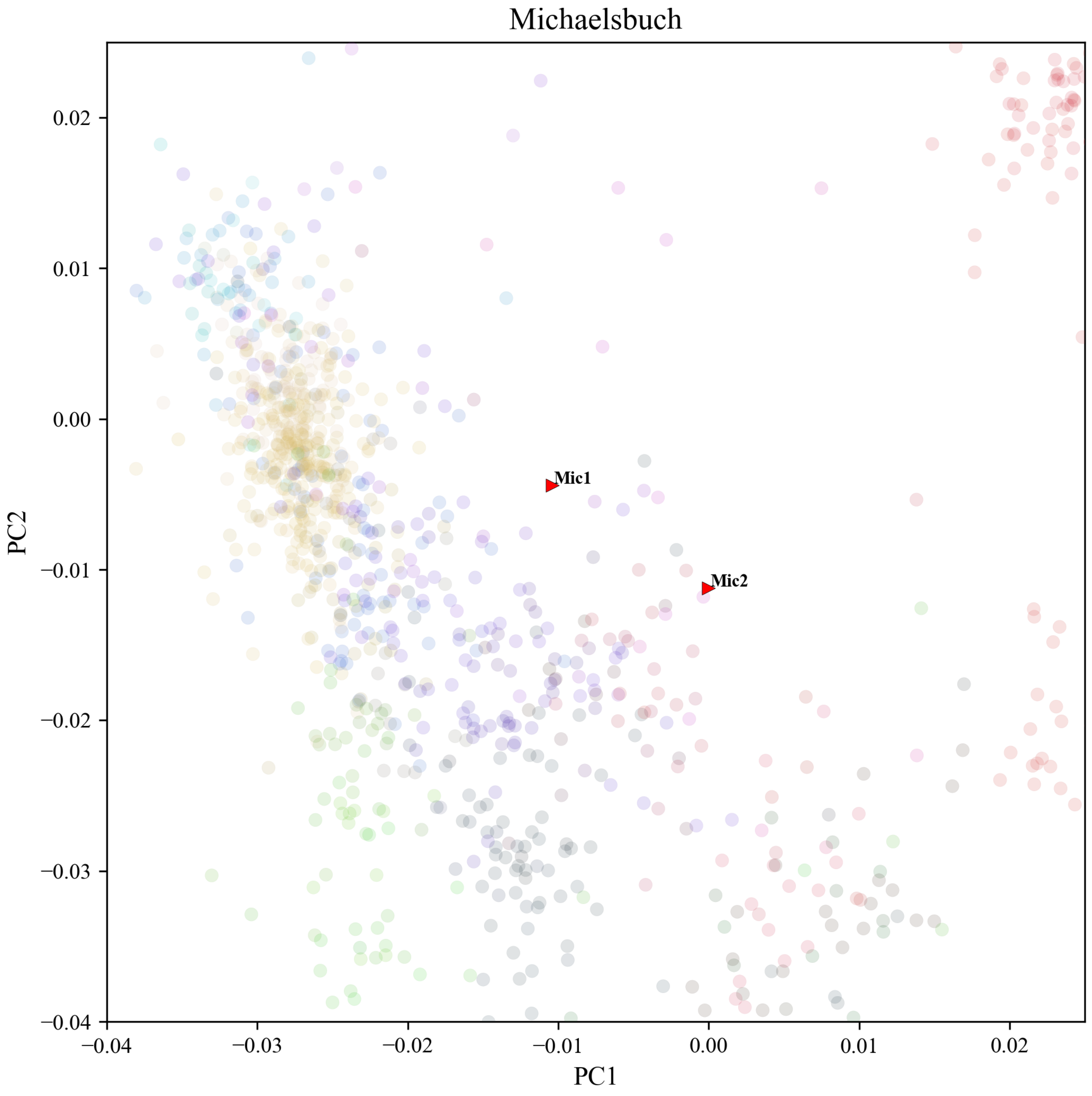

Fig. S1.12: *PCA of Michaelsbuch genomes plotted against a Central European pre-Roman/Iron Age reference. Newly sequenced genomes from the Michaelsbuch site (individually labeled) are projected onto a reference panel composed predominantly of Central European genomes. The geographic origins of the Iron Age reference genomes are colour-coded, as shown in the map in Fig. S7.4.*

Mattsies (N=1)
Johann Friedrich Tolksdorf

Sample „Mat1" derives from an infant burial at Mattsies (10.550694°E/ 48.088988°N), district Unterallgäu, Bavaria. A child, aged approx. 1.5 years, was placed in a stone-lined grave within an older Roman building structure. At least two subsequent roofed structures were built over the grave, probably to be used as memorial buildings. Sealing of the grave chamber with lime mortar prevented later influx of sediment and the burial was recovered in 2021 *en bloc* after the bones and objects had been fixed to the tiled grave floor by layers of ice. Documentation and conservation of the archaeological objects is still in progress. From the ^{14}C analysis of a rib an age of 1314±18 bp (MAMS-61457) was estimated, which matches the archaeological expectation. Based on the $d^{15}N$ values the child was still weaned, and anthropological analyses indicate chronic mastoiditis as the likely cause of death. The $^{87}Sr/^{86}Sr$-ratio is within the broad local range and does not contradict a local origin. The quality of the objects and the burial mode probably mirror an elevated social status of the individual and/or the family.
After initial screening revealed an endogenous DNA content of 30.61 %, the individual's genome was sequenced to a depth of 3.18 X.

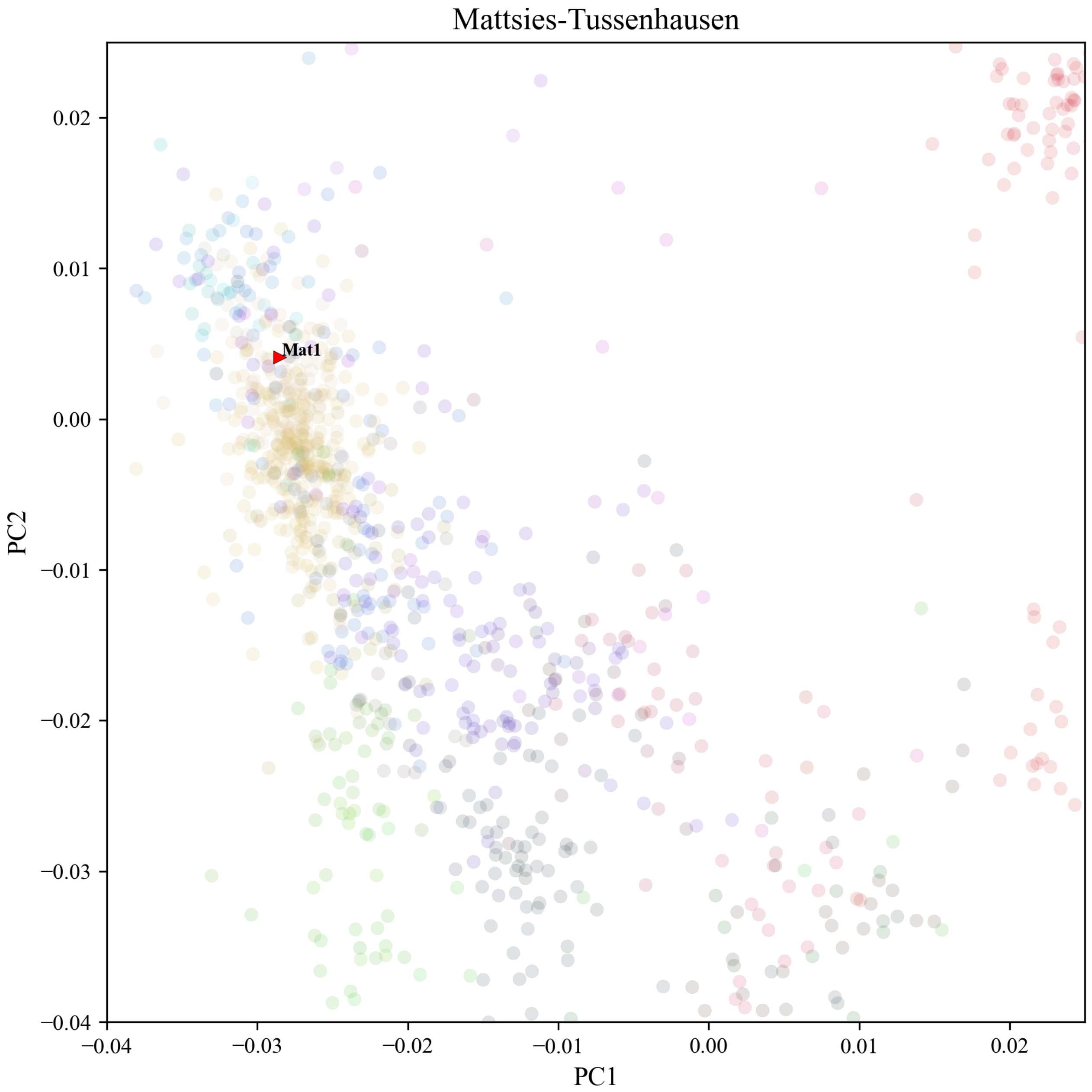

Fig. S1.13: *PCA of Mattsies genome plotted against a Central European pre-Roman/Iron Age reference. The newly sequenced genome from the Mattsies site (individually labeled) is projected onto a reference panel composed predominantly of Central European genomes. The geographic origins of the Iron Age reference genomes are colour-coded, as shown in the map in Fig. S7.4.*

Zschernitzsch (N=1)
Ines Spazier, Sandra Bock

Excavations at Zschernitzsch in the Altenburger Land district in Thuringia were carried out as part of the archaeological investigations at the long-distance gas pipeline in north-eastern Thuringia (FGL 32). It was carried out by the Thuringian State Office for the Preservation of Monuments and Archaeology (Thüringer Landesamt für Denkmalpflege und Archäologie) in Weimar. Burials at the site are assigned to the Linear pottery culture and the late Bronze Age - Early Iron Age. There is also one grave, which was genetically analyzed, identified as coming from the early Merovingian period [118–120].
Anthropological analysis of the skeleton revealed that it was an 18-24 year old (juvenile/early adult) female individual [121]. The individual was buried in a wooden coffin in West - Southwest to East - Northeast orientation and in an extended supine position. The head leant slightly to the right, the right arm was straight and the left arm slightly bent. Her grave was furnished with rich grave goods including a silver neck ring, two gold-plated silver fibulas, a finger ring made of bronze and a roll made of sheet bronze with a perforated sepiolite bead indicating that she was a member of the local elite.
Radiocarbon Dating (MAMS-50425, 1646 ± 25 BP; 383-529 calAD (68 %), 265-536 calAD (95 %)) and the grave goods place the burial in the 3rd quarter of the 5th century (450-475 AD). In 2022 a second grave dated to this period (MAMS-60932, 1639 ± 24 BP; 405-531 calAD (68 %), 377-538 calAD (95 %)) was recovered [118,119].
The endogenous DNA content of the one sample analyzed here was 20.60 % and the individual’s genome was sequenced to a depth of 4.50 X.

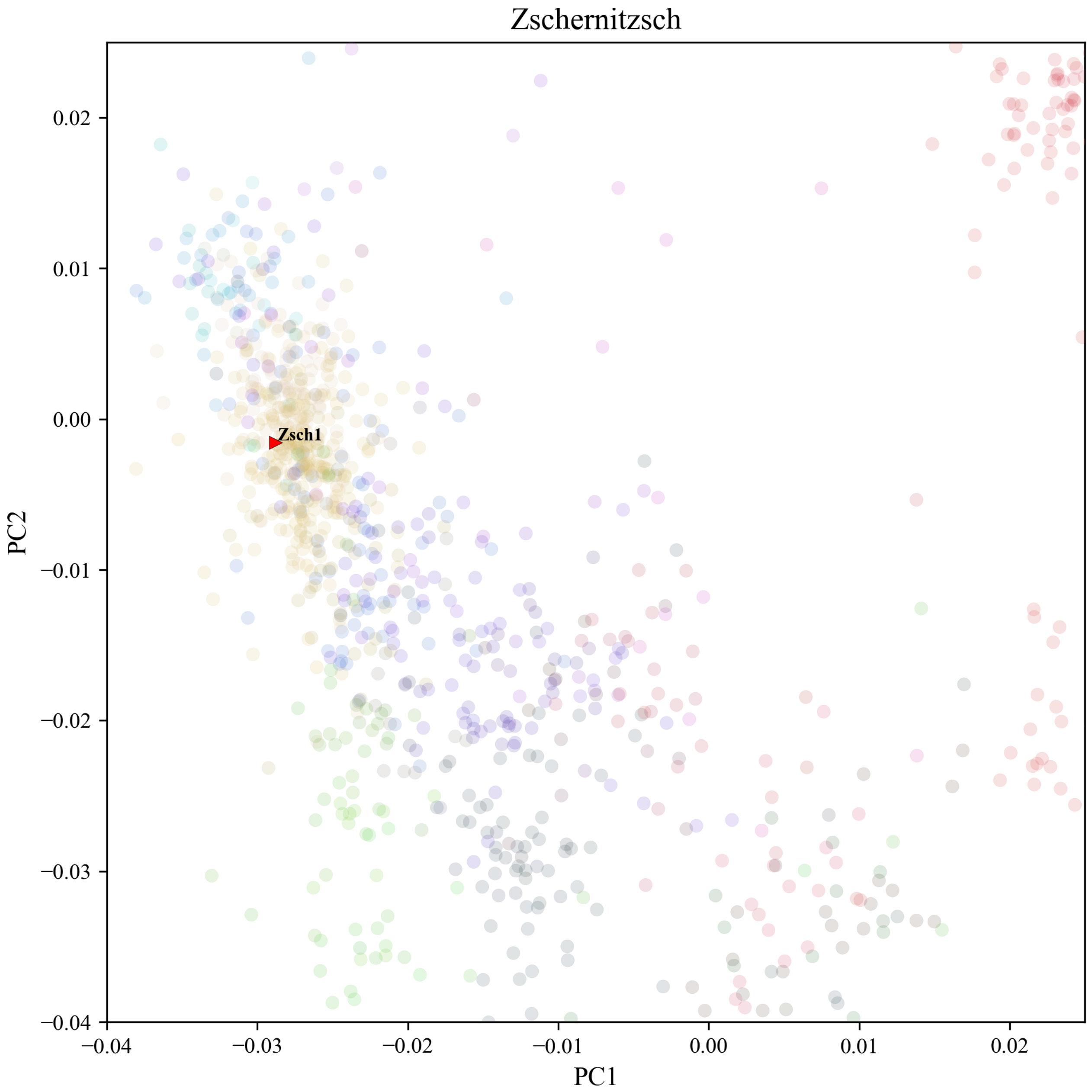

Fig. S1.14: *PCA of Zschernitzsch genome plotted against a Central European pre-Roman/Iron Age reference. The newly sequenced genome from the Zschneritzsch site (individually labeled), projected onto a reference panel composed predominantly of Central European genomes. The geographic origins of the Iron Age reference genomes are colour-coded, as shown in the map in Fig. S7.4.*

Straubing Azlburg (N=17)
Maren Velte

The Late Roman burial sites Straubing Azlburg I and Azlburg II were occupied from the end of the 3rd century to the first half of the 5th century and are associated with the Roman border fort in Straubing (*Sorvidurum*) (e.g., [122,123]) situated in today's city area on the church hill of St. Peter. The two cemeteries are found in close proximity to the former fort, separated only by the Allechbach stream from the church hill of St. Peter. Discovered during construction work and excavated in the 1980s, nearly 160 skeletons were unearthed (111 individuals from 107 burials at Azlburg I, 45 individuals from 43 burials at Azlburg II), although the full extent of the original area was not captured. The burial practices and grave goods vary significantly, reflecting both Roman and Germanic influences, which suggests interactions between “Romans” and “Germans” in the Straubing region during the Late Roman period. Furthermore, the findings imply that the nearby Late Roman fort was occupied by auxiliary troops [124] - units of the Roman army recruited from allied peoples or free inhabitants of the border provinces or beyond - underscoring the diversity of the population at Roman military centers during that time.
In this study, 17 skeletons from Azlburg (14 burials of Azlburg I and three burials of Azlburg II) were selected for genetic analysis. The petrous bones for genomic analysis were provided by the *SNSB, Staatssammlung für Anthropologie München* (SAM, state collection for Anthropology in Munich).
The endogenous DNA content of the samples analyzed here ranged from 38.95 % - 79.82 % (mean: 66.94 %; median: 74.60 %) and their genomes were sequenced to a depth of 1.77 X - 7.49 X (mean: 4.25 X; median: 4.25 X).
Available archaeological datings of investigated burials [123] were revised by Susanne Brather-Walter and Sebastian Brather. Osteological standard findings for all individuals were obtained following the guidelines of the SAM [86] by Maren Velte. Strontium isotope data of individuals was available from a previous study [125] indicating that one sampled individual (burial 99) originated from a geologically diverse region.

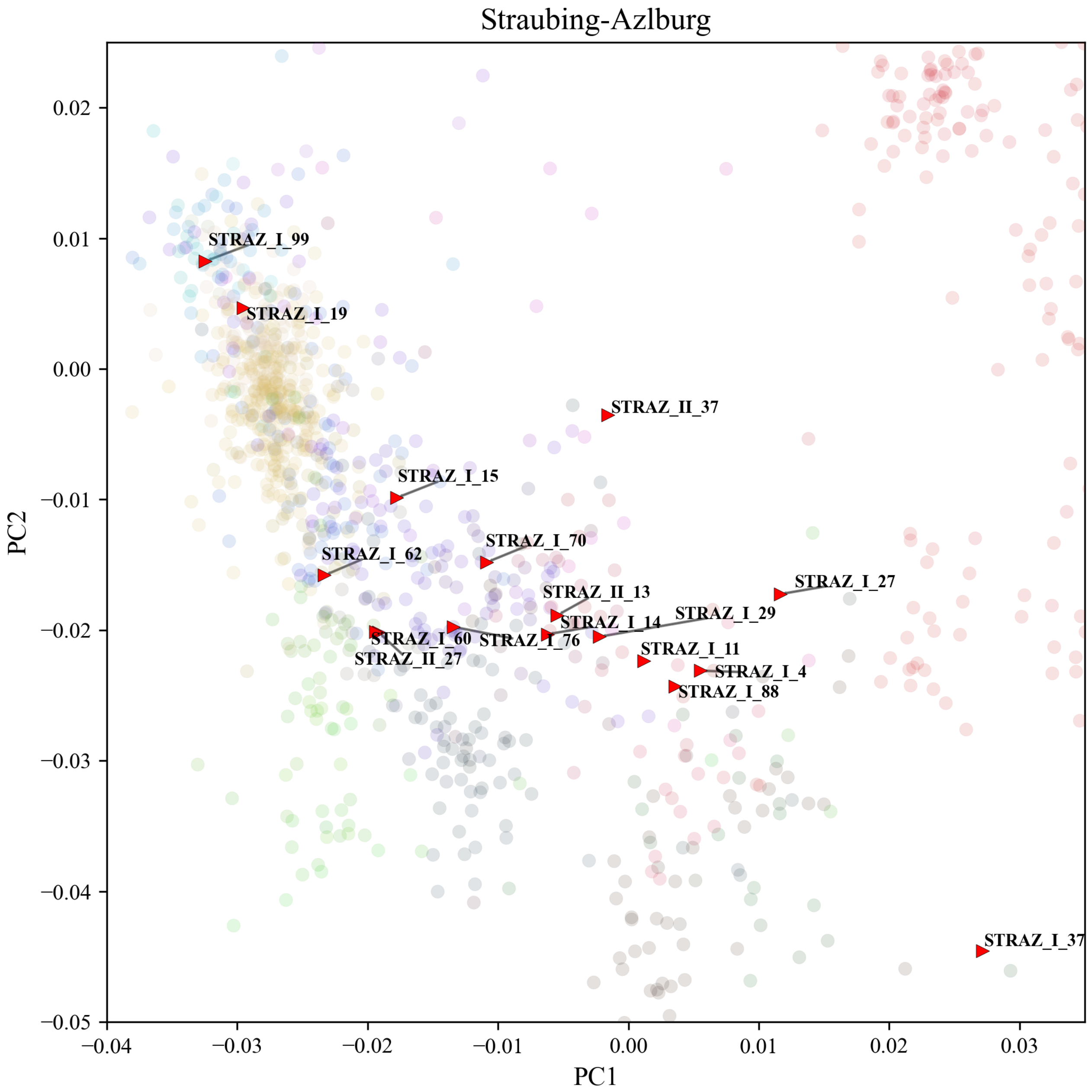

Fig. S1.15: *PCA of Straubing-Azlburg genomes plotted against a Central European pre-Roman/Iron Age reference. Newly sequenced genomes from the Straubing-Azlburg site (individually labeled) are projected onto a reference panel composed predominantly of Central European genomes. The geographic origins of the Iron Age reference genomes are colour-coded, as shown in the map in Fig. S7.4.*

Pförring, Eichstätt District (N=2)
Vera Planert

The Late Antique site is located on the western outskirts of Pförring in the Hallertau region, near the northern edge of the Danube wetlands. It lies close to the former Roman cavalry fort *Celeusum*, the easternmost of the trans-Danubian forts in the province of Raetia, positioned at a key crossing point of the Danube. The site was outside but within sight of the contemporary imperial border, in a narrow eastern strip between the Danube and the former Raetian Limes, approximately 9 km to the north.
Three graves have been identified northwest of what appears to be a contemporaneous but only partially excavated and not yet analyzed settlement. Graves 1 and 2 were located adjacent to each other, while Grave 3 was about 70 m away. They were excavated between 2016 and 2018 during three separate construction projects:

- M-2016-241-2_0, Feature 276 (Grave 1; Pfoe1)
- M-2018-1793-2, Feature 1 (Grave 2; Pfoe2)
- M-2018-1986-2, Feature 1 (Grave 3; Pfoe3)

To date, only preliminary reports have been published, while a comprehensive analysis, including scientific studies by V. Planert, is ongoing. The chamber grave from Pförring is one of the most completely preserved and richest examples of its time in Central Europe. The two simpler graves are also significant, as Late Antique chamber graves are often found in isolation without directly comparable individuals from the same population.
<u>Grave 1:</u> The chamber grave of Pförring – an undisturbed, exceptionally well-preserved wooden chamber grave containing the body of a young woman. The large and deep north - south oriented wooden chamber was identifiable through soil discoloration and partially preserved wooden remains. The deceased lay in a supine position on a bed in the western part of the chamber, richly dressed and adorned with elaborate necklaces and chest ornaments, including beads, brooches, and clasps, as well as a headdress with embossed metal fittings, a hairpin, a finger ring, and an extensive belt ensemble. Organic remains, including textile and fur fragments, were preserved. The chamber was furnished with various items, including a bed, chest, box, wooden container, weaving sword, comb, glass beaker, ceramic vessels, and food offerings.
<u>Grave 2:</u> An oversized, shallow inhumation grave, disturbed in the northern half from the pelvis upward. Its arrangement was similar to the chamber grave. The skeleton of a young woman laid in a supine position at the bottom of the rectangular north-south-oriented grave pit. The incomplete grave inventory included a comb, glass and coral beads, a simpler belt ensemble, and several small metal objects as grave goods.
<u>Grave 3:</u> A deep, simple, undisturbed inhumation grave. The skeleton of an older woman laid centrally within the rectangular North-Northwest – South-Southeast oriented grave pit, in a slightly right-turned supine position with slightly flexed legs. The only grave goods were a glass bead necklace, a bone tube, and a ceramic vessel.
Grave 1 is likely dated to the late 4th to very early 5th century. A *terminus post quem* of AD 352 is provided by three coins, which are heavily worn and have significantly enlarged perforations, suggesting a date no earlier than the last quarter of the 4th century. Unpublished radiocarbon dating

indicates a date at the latest around or shortly after AD 400. Some grave goods also suggest an early 5th-century date.

Due to their spatial proximity and similarities in grave construction and furnishings, Graves 1 and 2 may be roughly contemporaneous. However, Grave 2 could theoretically date within a broader range from the second half of the 4th century to the first quarter of the 5th century.

The 14C date for Grave 3 points more towards the 3rd to 4th century, while its artifacts fall within the 4th to early 5th century, showing some parallels to Graves 1 and 2. Given the varying 14C results for Grave 1—one of which is nearly as old as that of Grave 3—it remains possible that Grave 3 also dates to around AD 400.

The endogenous DNA content of the three samples analyzed here ranged from 0.73 % - 79.75 %. One sample was not suited for genome sequencing; the genomes of the other two samples were sequenced to a depth of 3.40 X and 3.98 X.

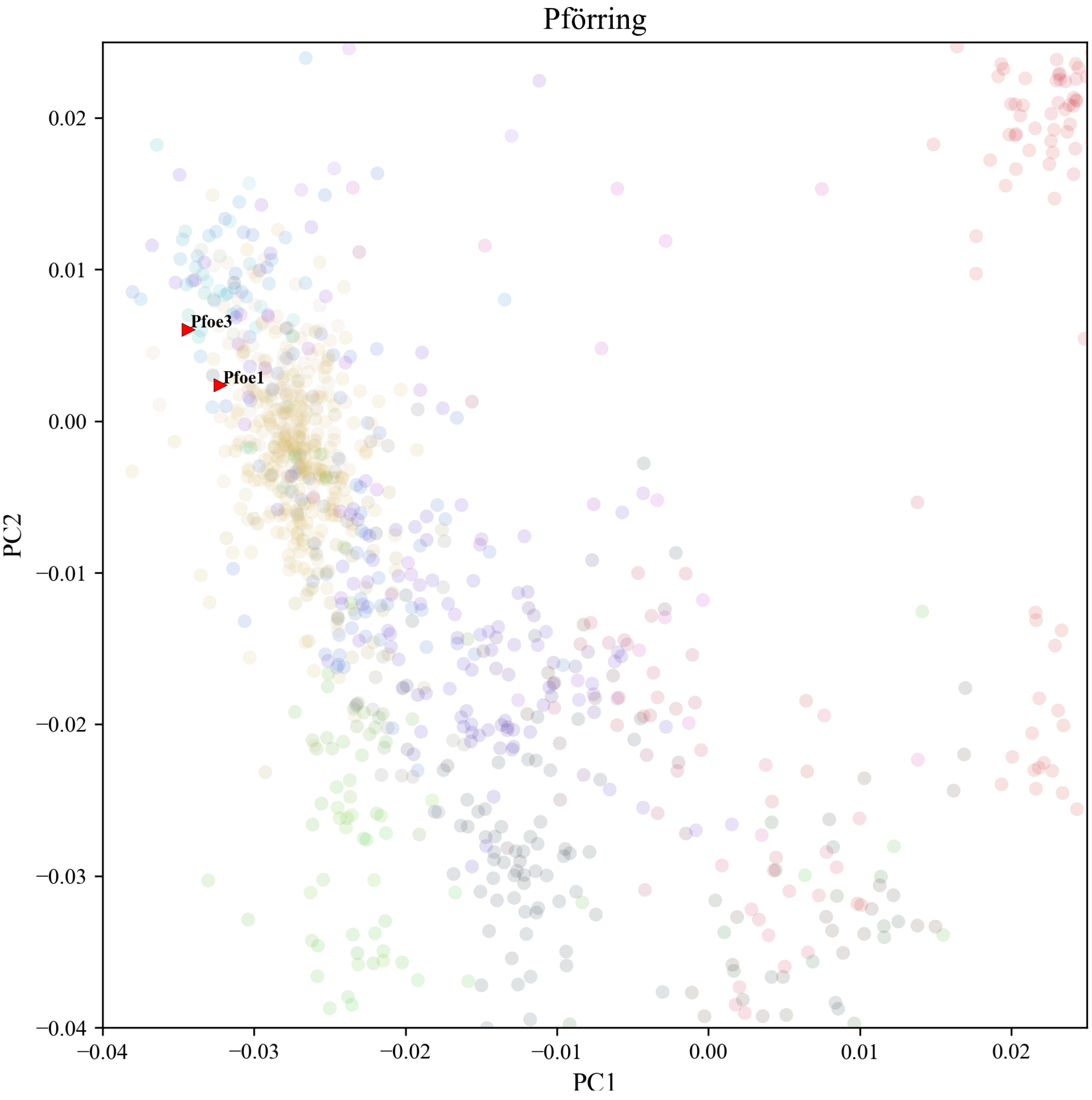

Fig. S1.16: *PCA of Pförring genomes plotted against a Central European pre-Roman/Iron Age reference. Newly sequenced genomes from the Pförring site (individually labeled) are projected onto a reference panel composed predominantly of Central European genomes. The geographic origins of the Iron Age reference genomes are colour-coded, as shown in the map in Fig. S7.4.*

Kemathen, Eichstätt District (N=1)
Vera Planert

The Late Antiquity chamber grave of Kemathen, located in Markt Kipfenberg in the Altmühl Valley, was excavated in 1990 but has only been partially analyzed and published in preliminary reports. The buried adult male has been described as a "Baiuvarian" warrior in Roman military service. The site is situated along the Altmühl River, approximately 25 km north of the Danube (the Late Antique imperial border) and just north of the former Raetian Limes. According to the excavator K. H. Rieder, the grave represents an individual burial within a contemporaneous settlement area, though the latter has not been systematically investigated.
Despite the absence of observable grave construction elements and the shallow depth of the finds, the burial is interpreted as a North - South oriented wooden chamber grave. The deceased was interred in a supine position in the western part of the chamber, richly dressed and equipped with a spatha, shield, wooden casket, probable horse harness, and a "military belt" with a pouch or pendant. Additional grave goods include a ring, comb, brooch with fur remains, a glass beaker, pig bones (meat offering), and five ceramic vessels. The grave is archaeologically dated to the first quarter of the 5th century [126–128]. The endogenous DNA content of the sample analyzed here was 2.03 % and genome sequencing resulted in a depth of 0.09 X.

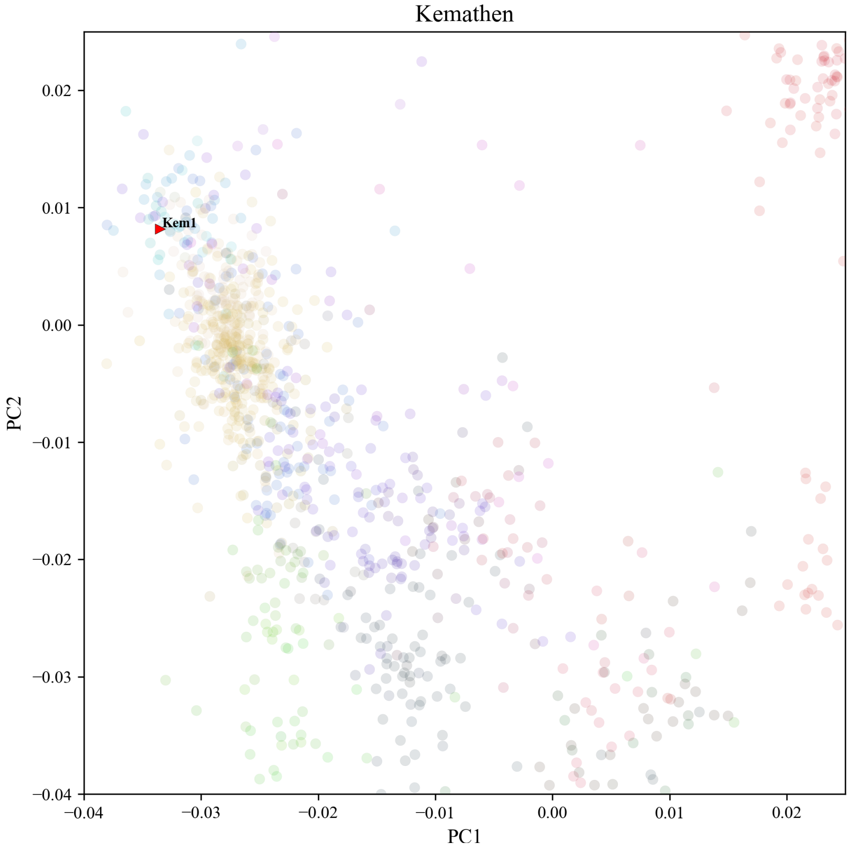

__Fig. S1.17:__ PCA of Kemathen genome plotted against a Central European pre-Roman/Iron Age reference. Newly sequenced genome from the Kemathen, Kipfenberg, site (individually labeled), projected onto a reference panel composed predominantly of Central European genomes. The geographic origins of the Iron Age reference genomes are colour-coded, as shown in the map in Fig. S7.4.

Viminacium, Serbia (N=10), Late Antiquity and Migration Period
Ilija Mikić, Regina Molitor

The ancient city Viminacium, in today's Serbia, was the capital of the Roman province *Moesia Superior* as well as a significant military camp. It was located within the Northern borders of the Roman Empire at the confluence of the Mlava River in the Danube and at a strategic crossing of roads. Excavations started in 1882 and are still ongoing. Investigations in Viminacium are carried out by a multidisciplinary team and documented by the Documentation Center Viminacium. There are several burial grounds in Viminacium with over 14,500 graves. The individuals analyzed here belong to the Anthropological Collection Viminacium and date to the period from the 4th to the 6th century (sources: Anthropological Collection Viminacium, [129–138]).

In the Pirivoj necropolis, used during the Roman period, 445 inhumations and 80 cremations were uncovered. Based on the bustum rite, some archaeologists consider it possible that the emperor Hostilianus was cremated here, a practice generally associated with individuals of high status. Grave G-152 was excavated in 2003 and was very well preserved. It can be dated indirectly to the middle of the 4th century based on its stratigraphic position within the necropolis and the form of its grave construction. The grave had a brick structure which was breached in the western part during robbery and contained no grave goods. The body was buried in West - East orientation. A three-edged iron arrow was stuck in the neck and the great trochanter of the right femur of the deceased and was likely the cause of death.

Više Grobalja is the second largest necropolis in Viminacium with 2913 excavated graves with inhumed and 1947 cremated deceased. A large number of about 30 individuals with artificially deformed skulls were found in this necropolis, which was in use during the Roman period, the Migration Period, as well as the Middle Ages. Grave G2-121, excavated in 1980 and in a very good state of preservation, dates to the second half of the 6th century based on characteristic grave goods, including a buckle related to the Sucidava type. Most likely it was a warrior's grave because the inventory contained an umbo, an iron sword, an iron knife and an iron spear. In addition, a bone comb, two buckles, and remains of ceramics were also found. The grave has no construction, and the deceased was freely buried in West - East orientation, lying on their back with their arms along the body. Burial G2-1770 was also very well preserved, and excavated in 1985. Although no grave goods were documented, the grave can be indirectly dated to the second half of the 5th century based on its position within the necropolis and the dating of neighboring burials. The grave has a northwest - southeast orientation and lacks any construction, with the deceased buried in a simple pit, their head resting on the right cheek.

The other graves from Više Grobalja genetically investigated in this study were excavated in 2020. Only one burial contained chronologically relevant grave goods, while the others, based on their grave construction style and stratigraphic location within the necropolis, are indirectly dated to the 4th century. All exhibit a consistent West - East orientation. Grave G-2697 was very well preserved, contained no grave inventory and had no grave construction. The deceased was laid on his back with his arms bent at the elbows. Both forearms were in a dislocated position, the right hand on the chest, the left dislocated. The head rests on the left cheek. Grave G-2706, which was well preserved, is a grave without grave construction and inventory. The deceased was freely buried, lying on his back and his knees slightly bent. The right hand was placed next to the body, while the left was missing. Grave G-2712 was poorly preserved, contained no inventory and had no grave

construction. The deceased was freely buried and laid on her back; only the bones of the skull, right arm and upper torso were preserved. The right arm was bent at the elbow and placed on the chest. The very well preserved grave G-2726 was built with a brick construction and contained a glass necklace, two bronze coins, a bronze earring and a bronze bracelet as grave inventory. The coins, including one from Emperor Valens (364–378), date the grave to the second half of the 4th century. The deceased was laid on her back, with the arms bent at the elbows and placed on the pelvis. Grave G-2756 was poorly preserved, had no grave construction and the inventory consisted of a glass bead; the deceased also laid supine. Grave G-2774 was very well preserved. The deceased was buried inside a brick structure laying on his back, with arms along the body. The upper part of the skeleton was dislocated to a greater extent during grave robbery, while the skull was placed on the chest. Grave G-2780 was poorly preserved and was built without construction. This body was also placed in supine position; her dislocated skull was found next to the legs.

The ten samples genetically analyzed here contained 61.11 % - 79.87 % (median: 77.22 %; mean: 74.68 %) endogenous DNA, allowing us to sequence 10 genomes with depth ranging from 2.04 X to 8.41 X (median 2.92 X; mean: 3.53 X).

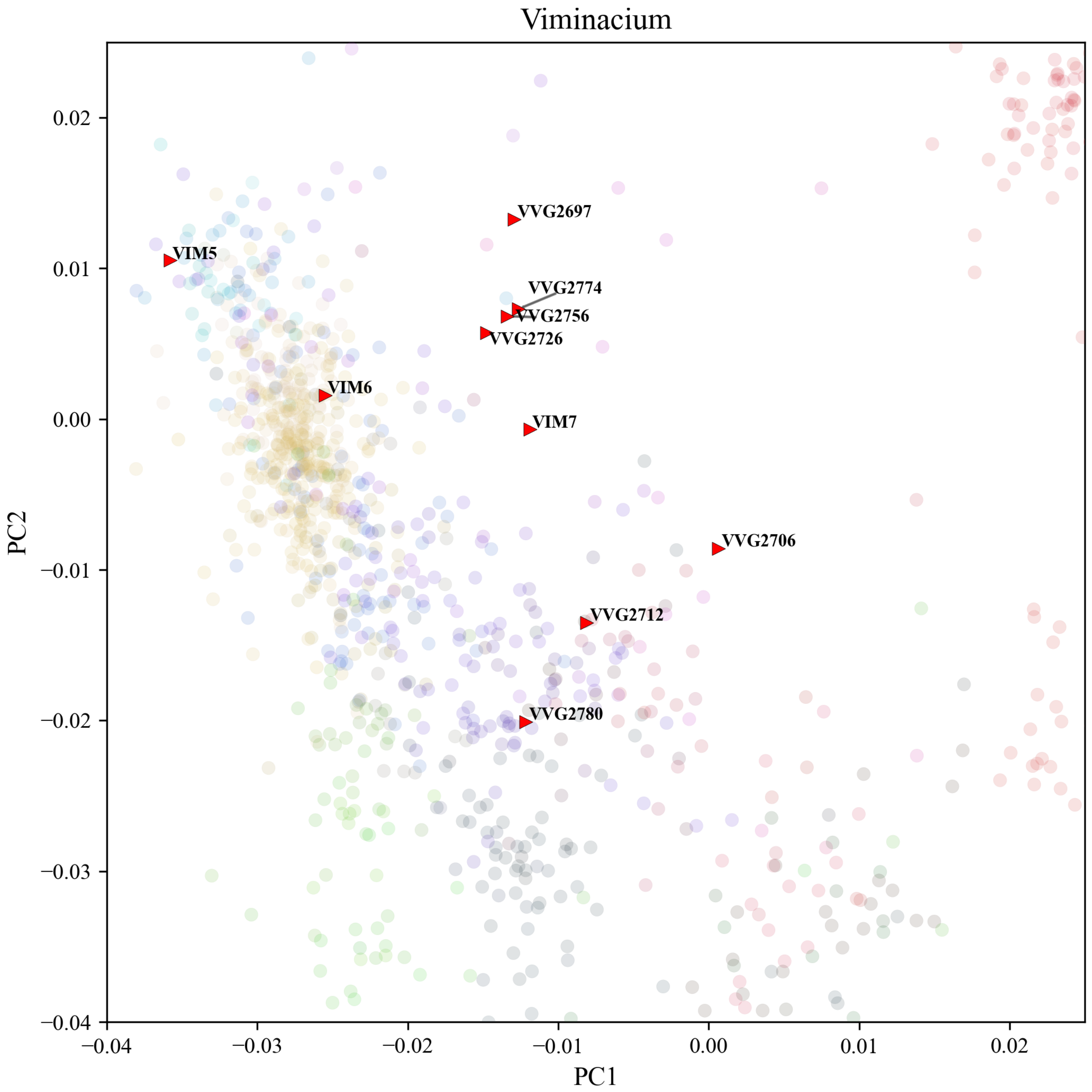

Fig. S1.18: *PCA of Viminacium genomes plotted against a Central European pre-Roman/Iron Age reference. Newly sequenced genomes from the Viminacium site (individually labeled) are projected onto a reference panel composed predominantly of Central European genomes. The geographic origins of the Iron Age reference genomes are colour-coded, as shown in the map in Fig. S7.4.*

Argamum, Romania (N=3), Late Antiquity
Bernd Päffgen

Argamum (Latin) or Orgame (ancient Greek) was an ancient port city in today's Romania. During Late Antiquity, Argamum was a city complex located directly on a bay (now Lake Razelm) of the Black Sea. The city was founded by the Greeks and later occupied by the Romans. The entire city area, which includes at least three churches, is poorly recorded and barely documented. Today it is a deserted site close to the commune of Jurilovca (Tulcea county, Northern Dobruja).
The samples analyzed here originate from a Late Antique to early Byzantine urban necropolis of the 4th to 6th century AD, which was located *extra muros* (=outside the city walls). A total of 6 individuals were analyzed from this site, but 3 samples (graves 18, 22 and 23) were not suited for genome sequencing due to low endogenous DNA content (<2 %; Arg2, Arg6) or lack of suitable material (Arg4). The mean endogenous DNA content of the remaining samples was 64.07% (54.36 % - 70.49 %, median: 67.36 %) with an average sequencing depth of 0.94 X (0.69 X - 1.15 X; median: 0.98 X).
Information on the grave orientation, grave goods and sex assigned based on grave goods is listed in table S1.5.
Sample Arg5_1 belongs to a Late Antique-early Byzantine children's grave from the 5th to early 6th centuries (Grave 30 general inventory of the necropolis). It was discovered during the 2009 fieldwork by several fragments discovered in squares 11-12, north profile/cross-section of the trench: maxilla, clavicle, and ribs. It was fully excavated in 2010. The skeleton has a preserved length of 0.64 m and a width in the thoracic area of 0.13 m; lying on its back, oriented West - East, with the head bent to the left. The bones were found in anatomical position up to the level of the pelvis, below this it was strongly disturbed; it presents, as arrangement, on the right side, two large limestone slabs (0.60x0.45 m; 0.50x0.55 m) to which the arrangement of Grave 27 is then attached. Assessment of the distributions of reads aligned to each chromosome using *BeXY* [139] indicated a trisomy of chromosome 21 (Fig. S6.1).
Sample Arg3 is from Grave 33 (general inventory of the necropolis) and was discovered in the area of the graves excavated in 2009. It is somewhat younger and belongs to the second half of the 6th century. The skeleton was in a dorsal decubitus position, with the hands beside the body, in a W - E orientation. Initially, it must have been in the anatomical position, but later disturbed by an external factor – animal? - so that the left side of the skeleton - the scapula and the left arm - were displaced to the lower limb. As a delimitation it had a string of stones on the north side and a stone at the foot. There was no inventory.
The burial remains of Grave 22a (sample Arg1) are stratigraphically younger, overlaying grave 22 and belong to a subsequent use of the necropolis, dating to the late 9th or probably the 10th century. Based on PCA and ancestry analyses it was found to differ from the two earlier individuals.

Table S1.5: *Archaeological information on the investigated individuals from Argamum.*

Lab ID	Archaeological ID	Grave orientation	Grave goods	archaeological sex
Arg1	P1, Argamum 2006, trench VIII, grave without number (near grave 22, general inventory)		no inventory	
Arg2	P2, Argamum 1995, trench V, grave 18	W-E	Late Roman coin and bones of sheep/ goat	
Arg3	P3, Argamum, 2010, trench VIII, grave 7 (= grave 33, general inventory)	W-E	no inventory	
Arg4	P4, Argamum 2006, trench VIII, squares 4-6, grave 1 (= grave 22, general inventory)	W-E	glass paste beads around the neck	female
Arg5_1	P5, Argamum 2010, squares 11-12, grave 3 (= grave 30, general inventory)	W-E	no inventory	
Arg6	P6, Argamum 2006, trench VIII, squares 6-7, grave 2 (= grave 23, general inventory)	W-E	glass paste beads around the neck	female

Table S1.6: *^{14}C Dating of 3 bone samples from the Argamum site. The datings were conducted in 2024 at the Curt-Engelhorn-Center for Archaeometry gGmbH.*

					Kalibrierte Alter					
Lab Nr MAMS	Sample Name	^{14}C Age [yr BP]	±	δ13C AMS [‰]	Wahrscheinlichkeit 68%	Wahrscheinlichkeit 95%	C:N	C [%]	Collagen [%]	Material
72422	Arg1	1131	16	-17.5	cal AD 890-973	cal AD 884-980	3.2	42.9	14.8	bone
72423	Arg3_1	1503	16	-14.5	cal AD 564-594	cal AD 549-602	3.2	42.7	15.0	bone
72424	Arg5_1	1613	17	-20.8	cal AD 420-531	cal AD 416-538	3.2	41.9	19.0	bone

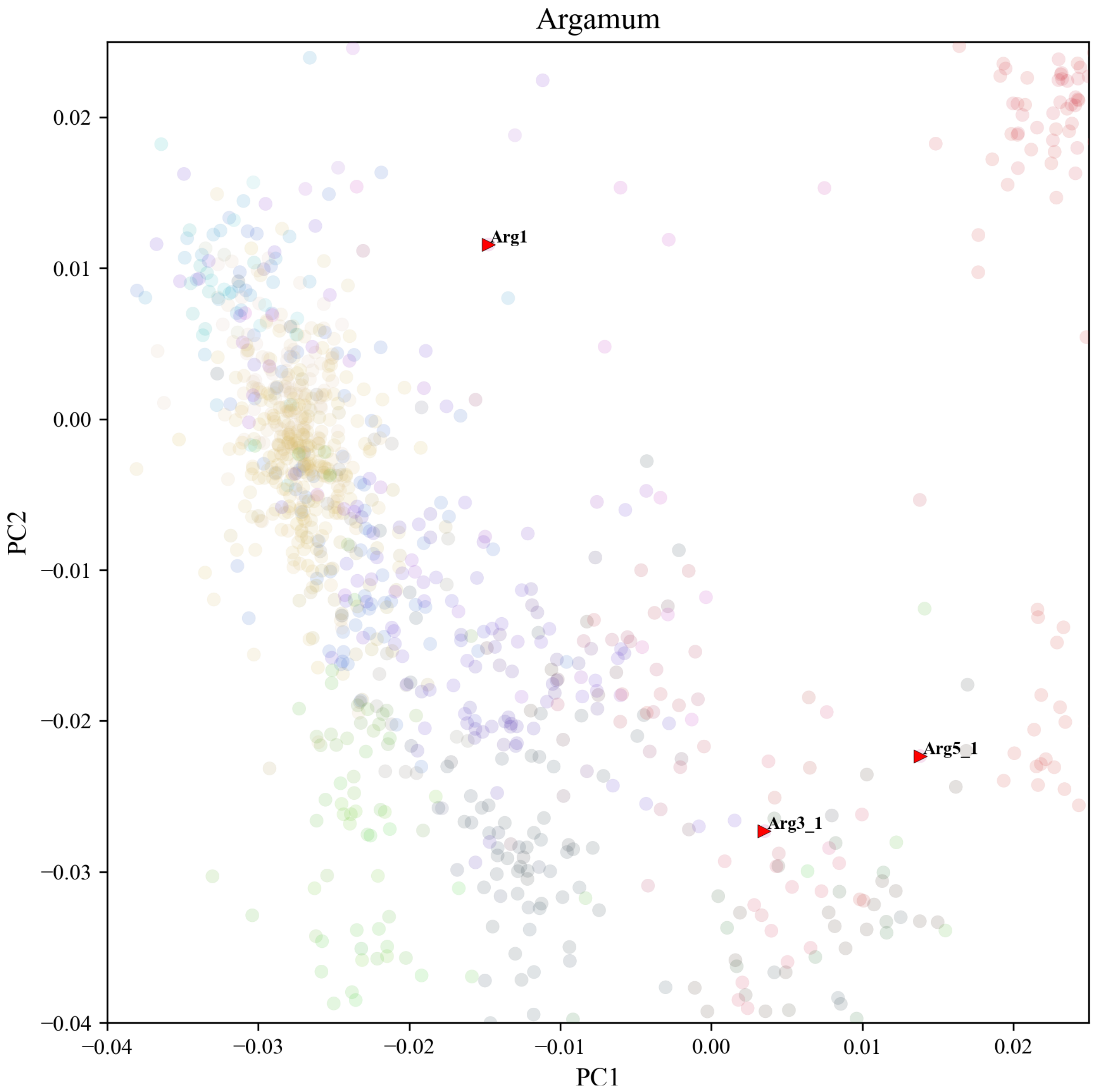

Fig. S1.19: *PCA of Argamum genomes plotted against a Central European pre-Roman/Iron Age reference. Newly sequenced genomes from the Argamum site (individually labeled) are projected onto a reference panel composed predominantly of Central European genomes. The geographic origins of the Iron Age reference genomes are colour-coded, as shown in the map in Fig. S7.4.*

Molzbichl, Austria (N=3), Late Early Middle Ages
Claus Vetterling, Kurt Karpf

Molzbichl, located in the federal state of Carinthia (Austria), is a district of the municipality of Spittal an der Drau and located around 5 km east of the district capital Spittal an der Drau. Archaeological excavations took place in KG 73413 Molzbichl, plot number 13/1Nr and were carried out by the association „Historisches Molzbichl“ and ReVe - Büro für Archäologie Bamberg und München.
In 1985/86 excavations were carried out in the vicarage garden and were continued in 1991 and 2013/14. The excavation site is located south of the parish church of St. Tiburtius and adjoins the current church cemetery. The initial purpose of the investigation was to provide structural evidence of a Tassilonian/Carolingian monastery, the existence of which has been confirmed by excavations in the church and historical sources [140–142]. The excavations 1991 and 2013/14 [143,144] were carried out due to construction work.
The archaeological investigations were limited to a small section of the site. So far, a total of 48 burials have been unearthed, with significantly more graves to be expected. The state of preservation of the skeletons is good to moderate. The burials showed no evidence of robberies, but post-mortem burial practices pointing to revenant superstitions (impalement, covering with stones) were present. Grave 3 is one of these special burials: a single grave of a mature woman in whose chest area a sharpened wooden stake was vertically hammered.
Jewelry and costume elements typical of the south-eastern Alps date the burials to the Early Middle Ages (8th - 11th century). They are mostly women's jewelry such as finger and head rings, earrings, glass beads and (disc) brooches.
The Early Medieval date is supported by ^{14}C analyses (Table S1.7). They give a time frame from the 9th to the 11th century. However, recent investigations have assigned three burials to the 13th century. It appears that parts of the area were still being used as a burial ground in the High Middle Ages.
Anthropological analyses revealed a moderate preservation of the three skeletons investigated here and did not find indications for pathologies [145].
Sr, C and N isotope analyses were performed for four individuals, two of which were also genetically analyzed here, and suggest a local origin for the individuals buried in grave 5, grave 9 (Molz3) and grave 12. The individual from grave 3 (Molz1) appears to have grown up in a different place than the previously mentioned individuals, but a local origin cannot be ruled out with certainty. The analyses indicate a mixed diet of plant and animal components. The proportion of animal proteins in the diet of Molzbichl grave 3 was apparently higher than in those of the other three individuals examined.
The endogenous DNA content of the three individuals genetically analyzed here ranged from 51.48 % to 75.64 % and sequencing resulted in three genomes with a depth of 1.45 X - 13.15 X.

Table S1.7: *Results of* ^{14}C *dating of three individuals from Molzbichl.*

Lab code	Grave number	^{14}C lab code	^{14}C date [BP]	calibrated age - 1 Sigma [calAD]	calibrated age - 2 Sigma [calAD]
Molz1	Grab 3	Erl-18894	1094 ± 37	942-989 (41,8%)	881-1019 (95,4%)
Molz2	Grab 2	Erl-18893	1056 ± 37	967-1021 (63,0%)	938-1027 (78,7%)
Molz3	Grab 9	Erl-18895	1050 ± 37	968-1022 (67,7%)	939-1031 (81,5%)

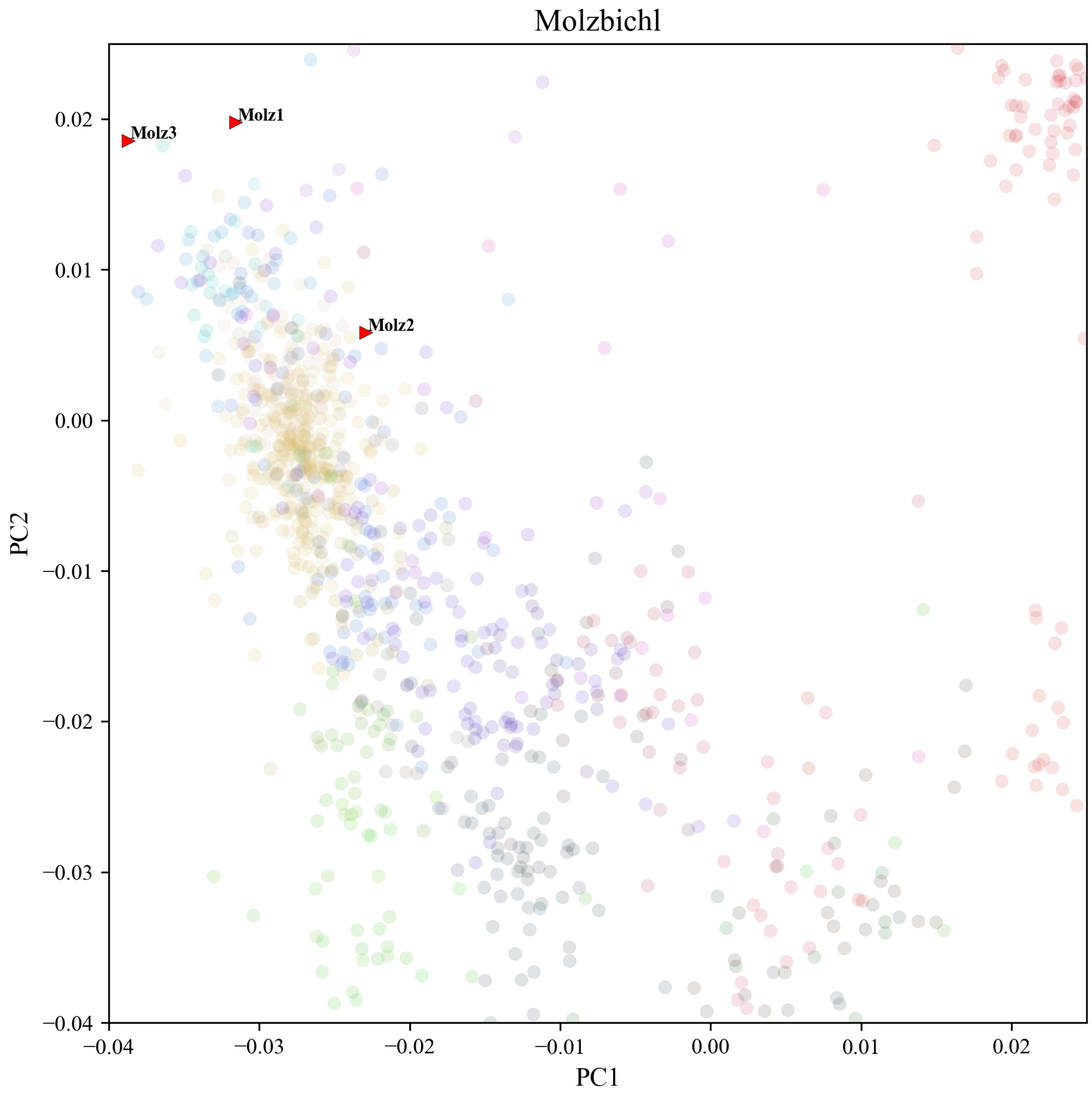

Fig. S1.20: *PCA of Molzbichl genomes plotted against a Central European pre-Roman/Iron Age reference. Newly sequenced genomes from the Molzbichl site (individually labeled) are projected onto a reference panel composed predominantly of Central European genomes. The geographic origins of the Iron Age reference genomes are colour-coded, as shown in the map in Fig. S7.4.*

Spina, Italy (N=2), Iron Age Etruscan site
Barbara Bramanti, Nicoletta Zedda

Spina was an Etruscan trading center on the Adriatic coast near the Po delta, in today's province of Ferrara, in the north-eastern Italian region of Emilia Romagna. One of the richest cities in Etruria Padana, it was founded in the 6th century BCE, a period in which the Etruscan population expanded considerably and strengthened its trade, acting as a bridge between the Po Valley area and the Greek area. In this climate of strong intermingling, it gradually became a cosmopolitan city, dedicated above all to trade, as evidenced by the extremely rich grave goods found. Spina and the other Etruscan cities of the Adriatic, such as the centers of Mantua and Adria, maintained their importance even after the 4th century BCE, a period from which the arrival of the Celts considerably weakened the Etruscan power [146,147].
The necropolis was located east of the settlement, beyond the ancient river Spinete, on some sandy paleo-dunes.
The city and necropolis were in use until the mid-3rd century BCE, when, probably because of hydrogeological changes, Spina lost its political and commercial function [148,149].
Starting in 1920, the burial area of Valle Trebba, the northern part of the necropolis, came to light thanks to reclamation works. Subsequently, the area of Valle Pega, in the southern part, was identified, which has been the focus of research since 1953. Originally, however, the Valle Pega and Valle Trebba areas corresponded to a single necropolis that stretched along the coast. Only more recently, in the 1970s, the settlement was also brought to light [146,147].
The total extent of the Spina necropolis included at least 4,000 graves, comprehensive of inhumations and incinerations. Biritualism is thus attested at Spina, although inhumation seems to be the prevalent form of deposition. The pit burials, generally placed on the raised areas emerging from the lagoon waters, contained skeletons oriented in a North - West/South - East direction with the head facing North - West. Cremations were instead placed inside containers, made of coarse or even refined pottery, such as Attic vases in the most expressive forms, and enclosed, as for inhumations, inside wooden boxes [150,151]. Skeletal remains are not well preserved, often individuals are represented by a few bone fragments with clear signs of taphonomic changes.
Anthropological remains from inhumations and cremations are currently being studied at the Laboratory of Archaeoanthropology and Forensic Anthropology at the University of Ferrara. The study is still in progress, but some preliminary data are available [152–159].
Two individuals were genetically analyzed here. Their endogenous DNA content was 45.25 % and 66.62 % and the two genomes were sequenced to a depth of 10.41 X and 11.81 X.

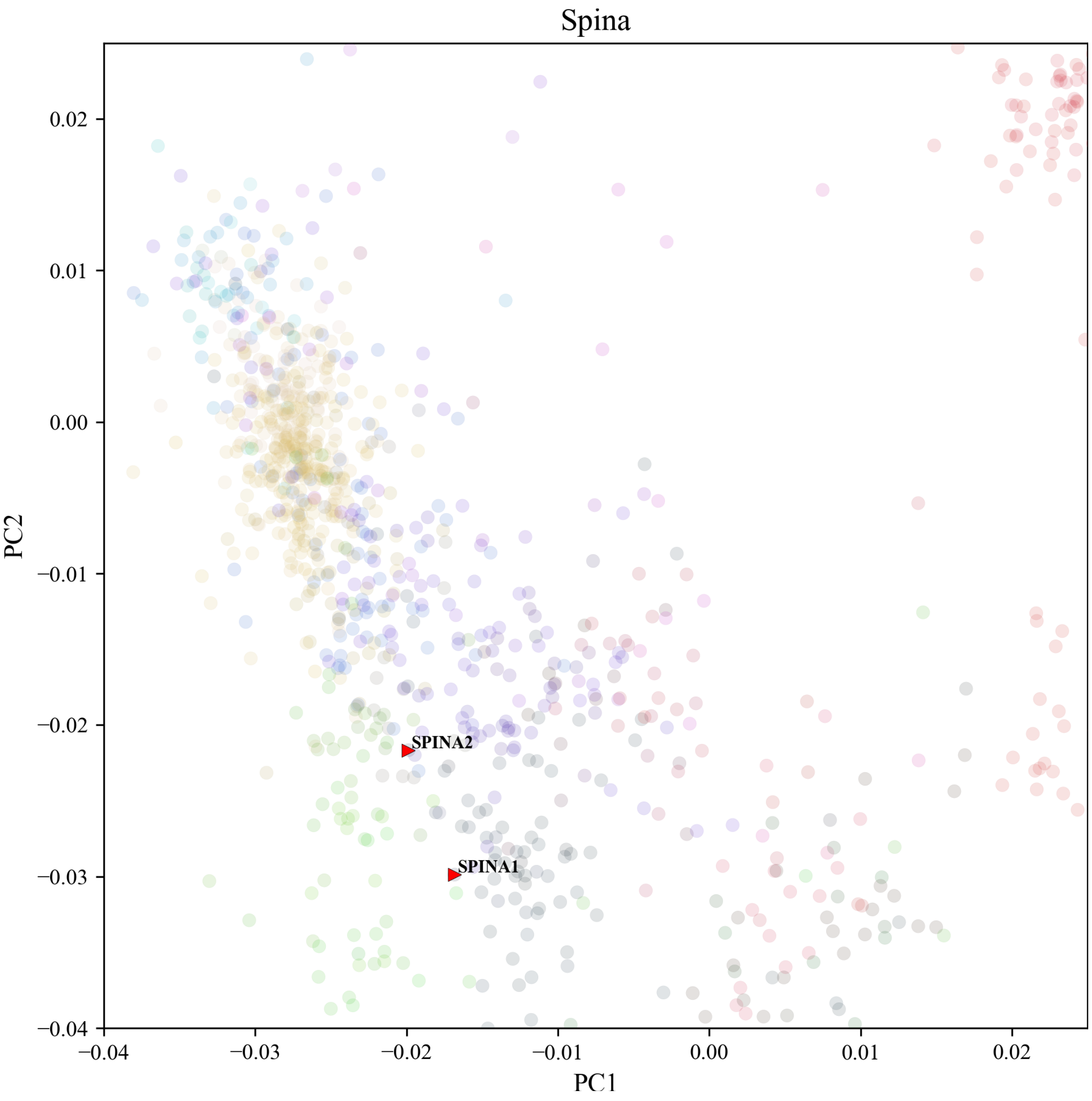

Fig. S1.21: *PCA of Spina genomes plotted against a Central European pre-Roman/Iron Age reference. Newly sequenced genomes from the Spina site (individually labeled) are projected onto a reference panel composed predominantly of Central European genomes. The geographic origins of the Iron Age reference genomes are colour-coded, as shown in the map in Fig. S7.4.*

Doliche, Turkey (N=1), Antiquity
George McGlynn

The individuals analyzed here are part of an anthropological cohort of human skeletal remains found during a 2017 rescue excavation in Doliche, a suburb of Gaziantep, Turkey, within a Roman provincial hypogeum dating to the 3rd-6th centuries AD. The hypogeum, used for burial over a period of 200-300 years, likely underwent multiple phases of interment, exhumation, and disturbance, suggesting it was utilized by a socially elite group. Despite limited artifact recovery, the structure's isolation and solid stone construction provided favorable conditions for skeletal preservation, although taphonomic factors caused varying degrees of damage. The disarticulated and incomplete remains reflect a healthy population with minimal signs of stress or disease, indicating an environment with adequate nutrition and low pathogen exposure [160].
We analyzed samples from two individuals. The genome of one individual (Lab ID: Dol2) could not be reconstructed due to insufficient endogenous DNA content (< 1 %). The other sample had an endogenous DNA content of 37.29 %, and its genome was sequenced to a depth of 0.72 X.

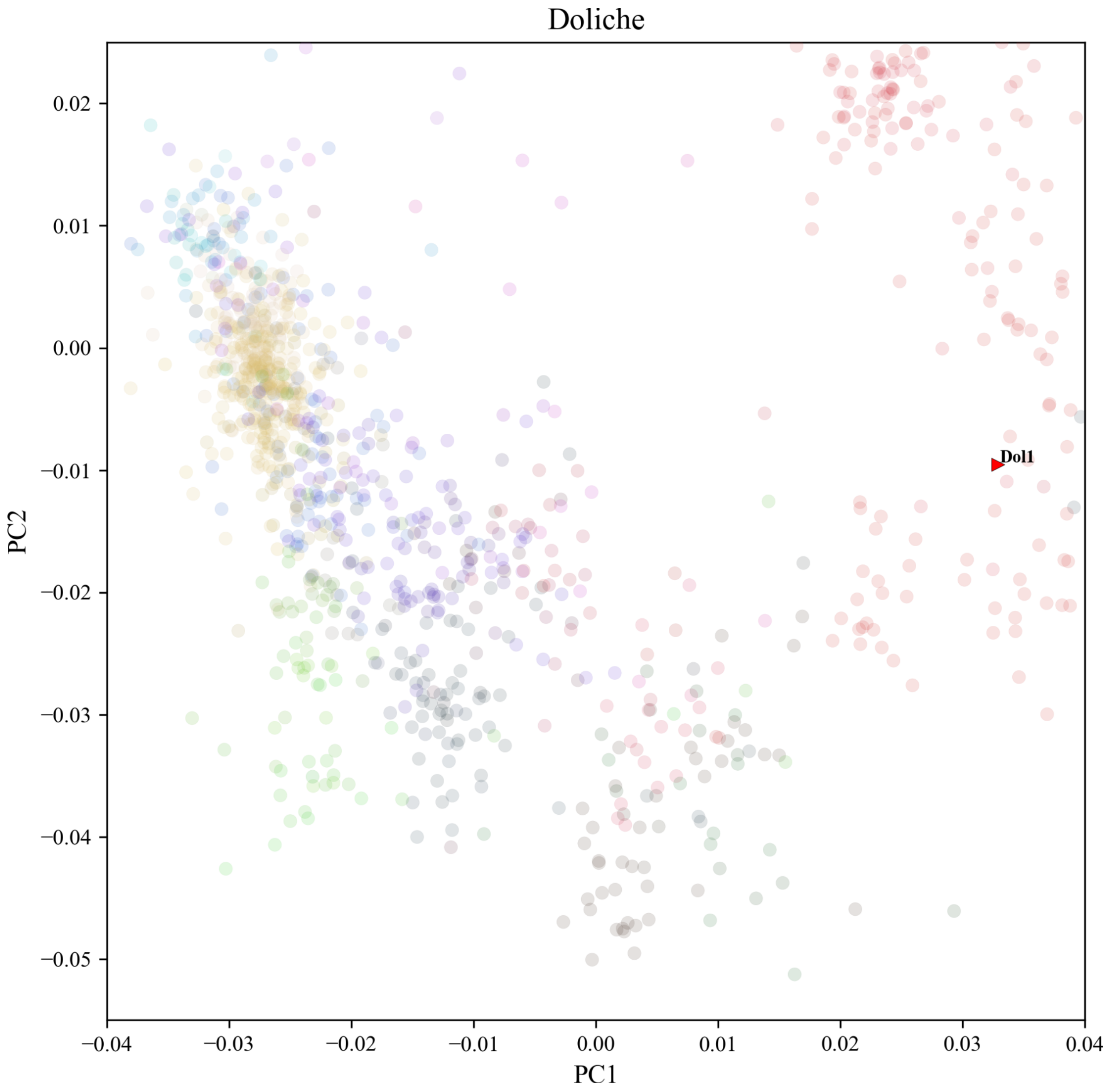

Fig. S1.22: *PCA of Doliche genome plotted against a Central European pre-Roman/Iron Age reference. Newly sequenced genome from the Doliche site (individually labeled), projected onto a reference panel composed predominantly of Central European genomes. The geographic origins of the Iron Age reference genomes are colour-coded, as shown in the map in Fig. S7.4.*

S2. Strontium isotope analysis of Altheim-Essenbach

Maren Velte, Stefan Hölzl, Michaela Harbeck

Strontium isotope analysis is a valuable tool in Anthropology for investigating prehistoric human migration (e.g. [161]). The method leverages the principle that geological landscapes have distinct strontium isotope signatures embedded in local bedrock. Through weathering processes, these signatures are transferred into soils and enter the food chain, ultimately becoming incorporated into the skeleton of animals and humans. Consequently, the bioavailable strontium isotope ratio ($^{87}Sr/^{86}Sr$) in teeth and bones primarily reflects the geochemical environment in which individuals lived and consumed food. The deviation of an individual's $^{87}Sr/^{86}Sr$ ratio from the local $^{87}Sr/^{86}Sr$ baseline is interpreted as evidence of migration.

However, correspondence between an individual's $^{87}Sr/^{86}Sr$ ratio and the local $^{87}Sr/^{86}Sr$ range cannot be taken as evidence of local origin. Individuals can also originate from other regions with similar geology and biologically available $^{87}Sr/^{86}Sr$ signatures. This geographical redundancy implies that a certain proportion of non-local individuals likely remain undetected, such that a minimum proportion of non-locals can be identified. Moreover, for detected non-locals, it is not possible to specify a specific region of origin.

The establishment of local $^{87}Sr/^{86}Sr$ baselines remains a matter of ongoing debate. Researchers employ various proxies (geological information, $^{87}Sr/^{86}Sr$ ratios of modern environmental samples, archaeological animal remains) or directly investigate $^{87}Sr/^{86}Sr$ data of human remains (e.g. [162–164]). For systematically identifying migrants within cemetery populations, individual $^{87}Sr/^{86}Sr$ ratios of tooth enamel are preferred over those from bones for two main reasons. First, enamel's high mineralization makes it more resistant to taphonomic changes, whereas bone is susceptible to contamination from the surrounding soil and groundwater that can alter its isotopic signature (e.g. [161]). Second, tooth enamel forms during childhood and changes very little after that (e.g. [165]). As a result, it reflects the geochemical environment of an individual's childhood, likely corresponding to his or her region of origin. In contrast, bones are constantly remodeled over the course of an individual's life (e.g. [166]), and their isotope signal reflects the later life environment, potentially masking earlier migration through adaptation.

In this study, $^{87}Sr/^{86}Sr$ ratios were obtained from tooth enamel of 109 individuals of the genetically investigated sample with at least one tooth present, as well as of five additional burials (Alh_128, Alh_142, Alh_148, Alh_160, Alh_180).

Second molars were prioritized for sampling; if these were not available, first molars or premolars were used.

Strontium extraction followed the protocol outlined by Toncala *et al.* (2020) [162]. Enamel was carefully isolated from dentine, cleaned through sonication in concentrated formic acid and washed with double distilled water, then incinerated at 500˚C. The samples were dissolved in concentrated nitric acid (69%) at 100˚C for at least 24 h and, after evaporation of the acid, in nitric acid (6 N) at 100˚C for 20 min. Strontium was isolated using a specialized resin SR-B25-S (Eichrom) and eluted from the matrix with nitric acid (0.05 N).

Mass spectrometry was carried out at the RiesKraterMuseum Nördlingen (ZERIN) using a thermal ionization mass spectrometer (MC-TIMS, MAT 261, Finnigan). Extracts were loaded onto wolfram single filaments and each sample was measured once, counting the isotopes 19 times in three blocks. Possible isotope mass fractionations were corrected by normalizing the $^{88}Sr/^{86}Sr$ ratio to

8.37521 [167]. The Strontium standard SRM 987 from the National Institute of Standards and Technology ($^{87}Sr/^{86}Sr$ 0.71034±0.00026) served as quality control reference. The certified reference value was adjusted to the generally accepted value of 0.71025±0.00001 [168,169], the mean $^{87}Sr/^{86}Sr$ ratio of SRM 987 during the measurements was 0.71022±0.00005 (n = 25, 1σ), ensuring analytical precision.

$^{87}Sr/^{86}Sr$ enamel ratios of the recent study sample are given in Supplementary Table 1. $^{87}Sr/^{86}Sr$ data and basic information of not genetically investigated burials from Altheim are summarized in table S2.1.

Table S2.1: *Basic information and $^{87}Sr/^{86}Sr$ data of individuals not included in genetic analysis.*

ID	Age at death [years]	Morphological sex	$^{87}Sr/^{86}Sr$ enamel	Sampled tooth
Alh_128	~5	NA	0.70980	46
Alh_142	30-50	female	0.70933	47
Alh_148	30-50	male	0.71024	27
Alh_160	~4	NA	0.70952	46
Alh_180	1-1,5	NA	0.70938	65?

Furthermore, 13 $^{87}Sr/^{86}Sr$ ratios of archaeological finds (human bone=2, human tooth dentine=4, human tooth enamel=6, animal tooth enamel=1) from two other sites less than 10 km away from Altheim-Essenbach (Altdorf, Ergolding) were available as reference data from the literature [170–172].
The entire data set of $^{87}Sr/^{86}Sr$ ratios (N=127) was used to determine the local range of bioavailable $^{87}Sr/^{86}Sr$ in the "Altheim region" following the procedure in Velte *et al.* (2023) [28]. The central tendency of the data was assessed using Gaussian kernel density estimates (KDEs), with the mode of the KDEs likely reflecting the values of the primary population. The biologically available $^{87}Sr/^{86}Sr$ range was defined as the Highest density interval covering the majority (99%) of the distribution (99% HDI) below the mode. Individuals with $^{87}Sr/^{86}Sr$ enamel ratios outside this range are considered to be non-local.
The local range of the Altheim region in South Bavaria is determined as 0.70816 – 0.71066. Eleven out of the 114 individuals from Altheim-Essenbach are identified to be non-local through increased $^{87}Sr/^{86}Sr$ enamel ratios above the local range (see Fig. S2.1).

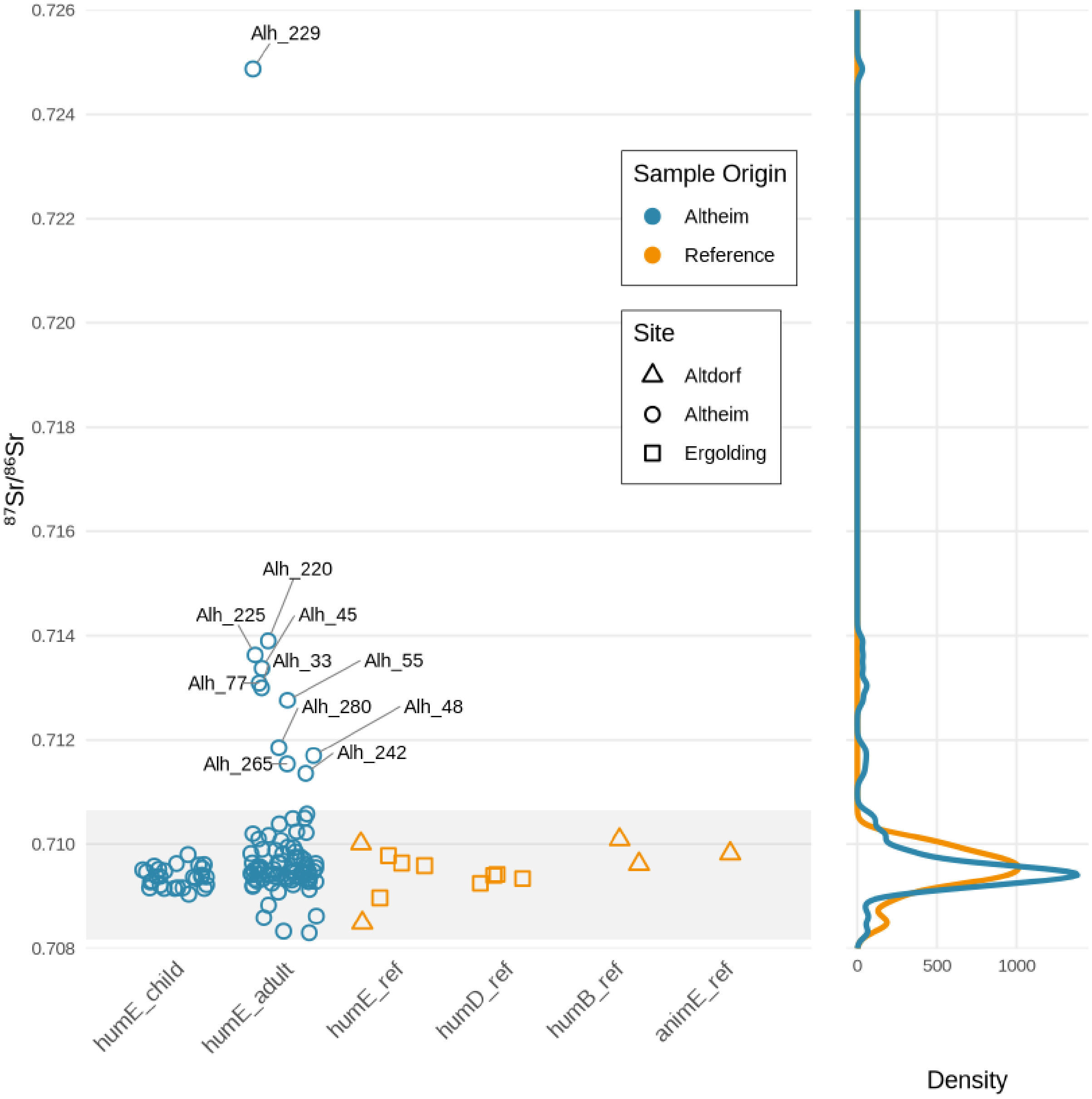

Fig. S2.1: Distribution of $^{87}Sr/^{86}Sr$ ratios and local range in the Altheim region. *$^{87}Sr/^{86}Sr$ (y-axis) of samples from the predefined study region divided into groups (x-axis): (1) Human samples from Altheim (sample set and additional burials) displayed in blue: humE_child= human tooth enamel of children (0-12 years), humE_adult= human tooth enamel of juveniles and adults (12+ years), (2) Human and animal samples from Altdorf and Ergolding (reference samples) displayed in orange: humE_ref= human tooth enamel of juveniles and adults (12+ years), humD_ref= human tooth dentine of adults (20+ years), humB_ref= human bone of juveniles and adults (12+ years), animE_ref= tooth enamel of a pig. Symbols indicate the archaeological site of samples. The local range of the Altheim region is calculated as the 99% HDI of the KDE with bandwidth=0.0001088802 [28] of the combined $^{87}Sr/^{86}Sr$ data from Altheim-Essenbach and reference data (N=127) and displayed as grey area. Data labels correspond to the ID of the individuals with $^{87}Sr/^{86}Sr$ ratios outside the local range that are considered to be non-local.*

The entire pre-Alpine foreland south of the Danube has a fairly uniform, relatively young geological surface and is surrounded by regions of, at least partly, different geology (e.g., [173]). Based on an extensive data set of over 900 skeletal samples from various time periods, the local $^{87}Sr/^{86}Sr$ range of the entire region is estimated to be 0.7081 - 0.7110 [28], which similarly corresponds to the

range of the Altheim region. Thus, small scale mobility within South Bavaria stays mostly invisible in $^{87}Sr/^{86}Sr$ ratios. In turn, the increased $^{87}Sr/^{86}Sr$ ratios of identified migrants in Altheim-Essenbach correspond to regions with higher strontium signatures than in the Northern Alpine foothills, such as geologically older regions, that can be found in different distances (e.g. the Bavarian Forest, Bohemia, the Odenwald, the Scandinavian Peninsula). Thus, identified migrants in Altheim-Essenbach likely originate from outside South Bavaria but their geographical origin remains unknown. Besides, it cannot be ruled out that there are additional migrants among the Altheim individuals showing $^{87}Sr/^{86}Sr$ ratios within the local range, who originate from regions with a similar geology and potentially similar $^{87}Sr/^{86}Sr$ local ranges (e.g. the Franconian and Swabian Alb, the North German lowlands, the Eastern European lowlands, the Eurasian steppe, the Po Valley in northern Italy).

Considering the chronology of burials from Altheim-Essenbach, focusing on the current study sample (n=109), it becomes apparent that the highest fraction of non-local individuals is found in the 5th century (Fig 2.2).

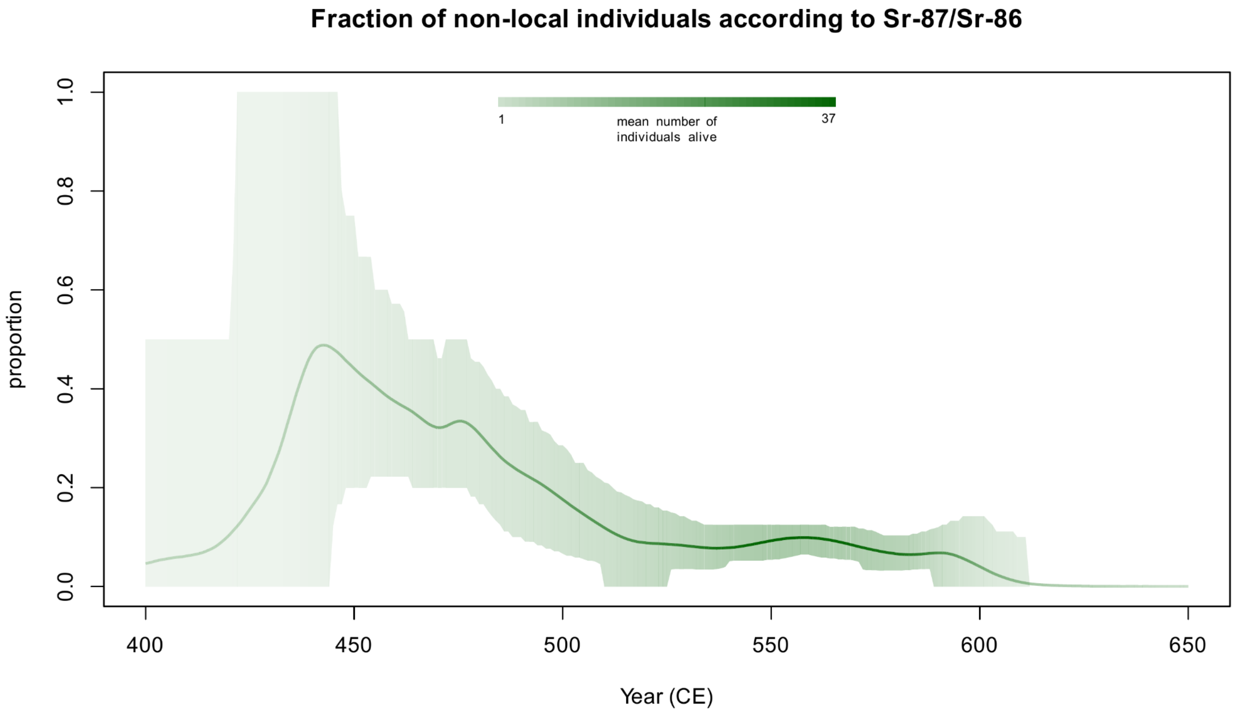

Fig. S2.2: Fraction of non local individuals *in the Altheim population according to* $^{87}Sr/^{86}Sr$. *Values were computed for each year from the 15000 MCMC iterations generated by Chronograph, the line indicates the mean computed over all iterations with the 90% CI given by the shaded area as the 5th and 95th percentiles of the distribution of values obtained. The transparency of the plot indicates the mean number of individuals alive at a given time over all the iterations.*

This corresponds well with the results of an earlier study suggesting a comparably high average minimum frequency of migrants (MFM: 23%; CI: 17-30%, n=150) in several other burial sites in South Bavaria for the 5th century [28]. Compared to other time periods, that is an above-average rate of migration into the region [28]. In the 6th century a considerably lower proportion of migrants is

observed in Altheim-Essenbach indicating decreased migration, at least from geologically diverse regions.

The identified migrants are exclusively individuals who have reached at least juvenile age, which is consistent with the general assumption that younger children were less mobile than adults. More migrants are females (n=8) than males (n=3), but the difference in the proportion of female (15%; 95% CI: 6-26%) and male migrants (5%; 95% CI: 0-12%) remains non-significant (Fisher's Exact Test p=0.2027).

S3. Inferring life histories on pedigrees

Recently, two approaches have been proposed to combine ^{14}C data with genetically inferred pedigree information to refine the dates of death of archaeological samples [174,175]. Here, we propose a novel approach `Chronograph` (Chronology inference using graphical models) that aims at inferring the dates of both birth and death of archaeological samples by combining information available through a large number of methods, including

1. archaeological dates of graves,
2. anthropological estimates of the age-at-death of individuals,
3. ^{14}C data of individuals,
4. constraints based on the stratigraphy of graves,
5. and relationships of individuals as given by a genetically inferred pedigree, and in particular parent-offspring relationships.

We note, however, that the modelling framework we propose is flexible regarding the data used and is easily extended to account for additional types of data.

Pedigrees

Consider a set of pedigrees. We denote by $\mathcal{I}$ the set of all individuals of these pedigrees, of which the subset $\mathcal{F} \subseteq \mathcal{I}$ are considered *founders* as their parents are not part of the pedigrees and the remaining subset $\mathcal{O} \subset \mathcal{I}$ are considered *offspring*, for which both parents are part of a pedigree. The smallest pedigree may consist of a single founder, $\mathcal{F} \cup \mathcal{O} = \mathcal{I}$ and $\mathcal{F} \cap \mathcal{O} = \varnothing$. Note that founders are sometimes also denoted as *originals* in the literature, while offspring may be referred to as *non-originals* or *non-founders*.

We denote several subsets for convenience:

- Let $\mathcal{S}(i) \in \{♀, ♂\}$ denote the biological sex of individual i and $\mathcal{S}_♀ \subseteq \mathcal{I}$ and $\mathcal{S}_♂ \subseteq \mathcal{I}$ the subsets of individuals that are women and men, respectively, with $\mathcal{S}_♀ \cap \mathcal{S}_♂ = \varnothing$.
- Let $\mathcal{P}(o) = \{\mathcal{P}_♀(o), \mathcal{P}_♂(o)\}$ denote the pair of mother and father of offspring $o \in \mathcal{O}$ with $\mathcal{P}_♀(o), \mathcal{P}_♂(o) \in \mathcal{I}$. We further denote by $\mathcal{P} = \bigcup_{o \in \mathcal{O}} \mathcal{P}(o)$ the pairs of mothers and fathers that have at least one common offspring. Note that an individual $i \in \mathcal{I}$ may occur in multiple such pairs if they had children with multiple other individuals.
- Let $\mathcal{O}(i)$ denote the set of offspring $\mathcal{O}(i) \subseteq \mathcal{O}$ of individual $i \in \mathcal{I}$. Analogously, let $\mathcal{O}(p) = \mathcal{O}(i,j) = \mathcal{O}(i) \cap \mathcal{O}(j)$ denote the set of common offspring for any pair of parents $p = \{i,j\} \in \mathcal{P}$. Finally, let $\mathcal{O}_s(i)$ denote the set of all pairs of siblings $\{o, o'\}$ of individual i such that $\mathcal{O}_s(i) = \{\{o, o'\} \subseteq \mathcal{O}(i), o \neq o'\}$
- Let $\mathcal{D} \subseteq \mathcal{I}$ denote the subset of all individuals with data.

Gibbs Random Field

We seek to model the life history $h_i = (b_i, l_i, d_i)$ of all individuals $i \in \mathcal{I}$, where we denote by b_i the birth date of individual i, by l_i their lifespan and by $d_i = b_i + l_i$ their death date, respectively, with $l_i > 0$ and hence $b_i < d_i$. We model all life history parameters as continuous numbers in years (e.g. $b_i = 755.5$ implies that the individual was born in the middle of year 755).

We model the distribution of the vector $\boldsymbol{h} = (h_i | i \in \mathcal{I})$ of all individuals across the pedigrees as a Gibbs Random Field (Fig. S3.1), in which each individual is a node and two individuals $i, j \in \mathcal{I}, i \neq j$, are connected by an edge

- if one individual is a parent of the other individual, i.e. if $i \in \mathcal{P}(j)$ or $j \in \mathcal{P}(i)$,
- if both individuals are parents of at least one common offspring, i.e. if $\mathcal{O}(i, j) \neq \varnothing$,
- or if i and j have the same mother, i.e. if $\mathcal{P}_{♂}(i) = \mathcal{P}_{♂}(j)$.

The joint density of this Gibbs Random Field with parameters $\boldsymbol{\theta}_G = (\boldsymbol{\theta}_l, \boldsymbol{\theta}_f, \boldsymbol{\theta}_p, \boldsymbol{\theta}_o, \boldsymbol{\theta}_s)$ factorizes as follows:

$$\begin{aligned}\pi(\boldsymbol{h}|\boldsymbol{\theta}_G) \propto &\prod_{i \in \mathcal{I}} f_l(h_i|\boldsymbol{\theta}_l) \prod_{i \in \mathcal{F}} f_f(h_i|\boldsymbol{\theta}_f) \prod_{(i,j) \in \mathcal{P}} f_p(h_i, h_j|\boldsymbol{\theta}_p) \\ &\prod_{i \in \mathcal{O}} \left[f_o(h_i, h_{\mathcal{P}_{♀}(i)}|\boldsymbol{\theta}_o) f_o(h_i, h_{\mathcal{P}_{♂}(i)}|\boldsymbol{\theta}_o) \right] \\ &\prod_{m \in \mathcal{S}_{♀}} \prod_{\substack{(i,j) \in \mathcal{O}(m) \\ i \neq j}} f_s(h_i, h_j | h_m, \boldsymbol{\theta}_s).\end{aligned}$$

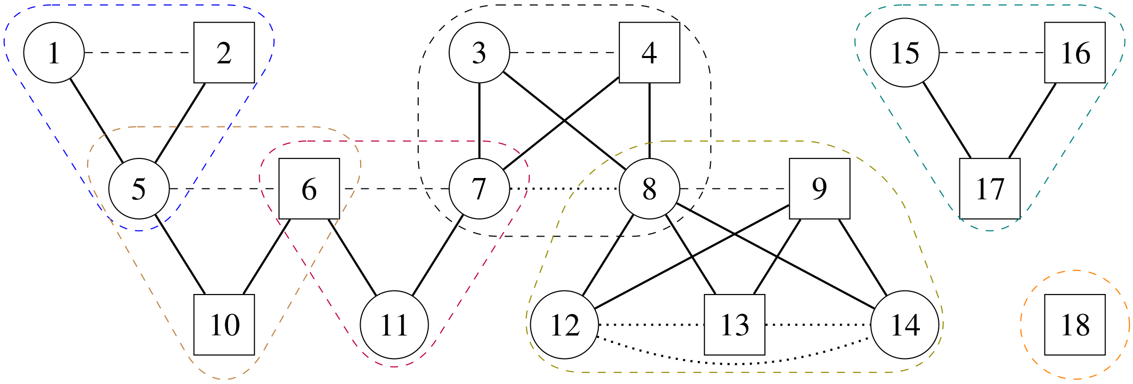

Fig. S3.1: *Pedigrees as Gibbs Random Fields. Each node represents an individual, of which circles are women and squares are men. Edges represent different types of relationships: 1) solid lines connect parents with their offspring, 2) dashed lines connect parents that have common offspring, and 3) dotted lines connect siblings with the same mother. Dashed and colored boxes represent the set of maximal cliques in this example.*

It consists of the following five parts:

- The lifespan potential function $f_l(h_i|\boldsymbol{\theta}_l)$ applied to each individual $i \in \mathcal{I}$.
- The founder potential function $f_f(h_i|\boldsymbol{\theta}_f)$ applied to each founder $i \in \mathcal{F}$.
- The parental potential function $f_p(h_m, h_f|\boldsymbol{\theta}_p)$ applied to each pair of parents $(m, f) \in \mathcal{P}$.
- The offspring potential function $f_o(h_i, h_j|\boldsymbol{\theta}_o)$ applied to each pair of offspring i and their parents $p \in \mathcal{P}(i)$.
- The sibling potential function $f_s(h_i, h_j|h_{\mathcal{P}_♀(i)}, \boldsymbol{\theta}_s)$ applied to all age-ordered pairs of siblings $\{i, j\} \subset \mathcal{O}(m), m \in \mathcal{S}_♀, b_i < b_j$ that have the same mother and of which individual i was born prior to individual j.

Factorization over maximal cliques

We note that the joint density of the Gibbs Random Field defined here also factorizes over the set $\mathcal{C}$ of maximal cliques as

$$\pi(\boldsymbol{h}|\boldsymbol{\theta}_G) \propto \prod_{c\in\mathcal{C}} \phi_c(\boldsymbol{h}_c), \tag{1}$$

where each unconnected individual as well as each nuclear family (a mother, a father and their common offspring) represent a maximal clique (Fig S3.1), $\boldsymbol{h}_c$ denotes their pairs of birth and death dates of all individuals $i \in c \subseteq \mathcal{I}$ and ϕ_c denotes the potential function of clique c. We denote by $n_c(i)$ the number of maximal cliques containing individual $i \in \mathcal{I}$ and by $\mathcal{P}(c)$, $\mathcal{P}_♀(c)$ and $\mathcal{O}(c)$ the parents, the mother and their offspring among the individuals of clique c such that $c = \mathcal{P}(c) \cup \mathcal{O}(c)$ for maximal cliques of nuclear families but $\mathcal{P}(c) = \mathcal{P}_♀(c) = \mathcal{O}(c) = \varnothing$ for maximal cliques of single, unconnected individuals. The potential function of clique c is then given by

$$\begin{aligned}\phi_c(\boldsymbol{h}_c) = &\prod_{i\in c} f_l(h_i|\boldsymbol{\theta}_l)^{\frac{1}{n_c(i)}} \prod_{i\in c\cap\mathcal{F}} f_f(h_i|\boldsymbol{\theta}_f)^{\frac{1}{n_c(i)}} \\ &\times \prod_{i\in\mathcal{O}(c)} \left[f_o(h_i, h_{\mathcal{P}_♀(i)}|\boldsymbol{\theta}_o) f_o(h_i, h_{\mathcal{P}_♂(i)}|\boldsymbol{\theta}_o)\right] \\ &\times f_p(h_{\mathcal{P}(c)\cap\mathcal{S}_♀}, h_{\mathcal{P}(c)\cap\mathcal{S}_♂}|\boldsymbol{\theta}_p)^{\mathrm{I}(\mathcal{P}(c)\neq\varnothing)} \\ &\times \prod_{m\in\mathcal{P}_♀(c)} \prod_{\{i,j\}\in\mathcal{O}_s(m)} f_s(h_i, h_j|h_{\mathcal{P}_♀(i)}, \boldsymbol{\theta}_s).\end{aligned} \tag{2}$$

Additional global time constraints

In some cases there is additional external knowledge regarding pairs of individuals that may or may not be connected in the Gibbs Random Field defined above. One such example is grave order constraints: if one grave was found on top of another, the upper one must be younger than the lower one. Similarly, if some individuals died at the same or very similar time, for instance in case of an accident or violent act, their death dates must be identical or at least very similar.

Let $\mathcal{T}$ denote the set of all pairs of individuals $(i,j) \in \mathcal{T}$, $i \neq j$, for which time constraints exist, and let $f_T^{(ij)}(h_i, h_j)$ be the potential function associated with the pair (i,j). Any individual may be part of several pairs.

The full joint density of the Gibbs Random Field is given by

$$\pi(\boldsymbol{h}|\boldsymbol{\theta}_G, \mathcal{T}) \propto \pi(\boldsymbol{h}|\boldsymbol{\theta}_G)\pi(\boldsymbol{h}|\mathcal{T})$$

with

$$\pi(\boldsymbol{h}|\mathcal{T}) = \prod_{(i,j)\in\mathcal{T}} f_T^{(ij)}(h_i, h_j). \tag{3}$$

In case of grave order constraints, for instance, one may use the indicator function $f_T^{(ij)}(h_i, h_j) = \mathrm{I}(d_i < d_j)$ if individual i is known to have been buried prior to individual j. However, more permissive functions may lend themselves more readily for optimization.

Inference

As any Gibbs Random Field, the model proposed above lends itself for Bayesian inference under an MCMC scheme. We denote by D_i the full set of data for individual $i \in \mathcal{I}$, for which the density is given by $\pi(D_i|h_i, \boldsymbol{\theta}_D)$ with parameters $\boldsymbol{\theta}_D$. Note that for individuals $i \notin \mathcal{D}$, $\pi(D_i|h_i, \boldsymbol{\theta}_D) = 1$. Denoting by $\boldsymbol{D} = \{D_i | i \in \mathcal{I}\}$ the set of all data, the posterior density of interest is then given by

$$\pi(\boldsymbol{\theta}_G, \boldsymbol{\theta}_D|\boldsymbol{D}, \mathcal{T}) \propto \pi(\boldsymbol{D}|\boldsymbol{h}, \boldsymbol{\theta}_D)\pi(\boldsymbol{h}|\boldsymbol{\theta}_G)\pi(\boldsymbol{h}|\mathcal{T})\pi(\boldsymbol{\theta}_D)\pi(\boldsymbol{\theta}_G),$$

where

$$\pi(\boldsymbol{D}|\boldsymbol{h}, \boldsymbol{\theta}_D) = \prod_{i\in\mathcal{D}} \left[\pi(D_i|h_i, \boldsymbol{\theta}_D)\right],$$

$\pi(\boldsymbol{h}|\boldsymbol{\theta}_G)$ is given by (eq. 2), $\pi(\boldsymbol{h}|\mathcal{T})$ is given by (eq. 3) and $\pi(\boldsymbol{\theta}_D)$ and $\pi(\boldsymbol{\theta}_G)$ are prior distributions.

Specific choices of potential functions and prior distributions

The model and inference scheme proposed above is flexible regarding the choices of potential functions. Here we use the following functions:

- *Lifespan potential function* $f_l(h_i|\boldsymbol{\theta}_l)$

  We assume that lifespans l_i of all individuals $i \in \mathcal{I}$ follow a Gompertz distribution with parameters $\eta_♀$ and $\beta_♀$ for women and $\eta_♂$ and $\beta_♂$ for men such that

$$f_l(h_i|\boldsymbol{\theta}_l) = \begin{cases} \beta_♀\eta_♀ \exp\left(\eta_♀ + \beta_♀ l_i - \eta_♀ e^{\beta_♀ l_i}\right) & \text{if } i \in \mathcal{S}_♀, l_i > 0 \\ \beta_♂\eta_♂ \exp\left(\eta_♂ + \beta_♂ l_i - \eta_♂ e^{\beta_♂ l_i}\right) & \text{if } i \in \mathcal{S}_♂, l_i > 0 \\ 0 & \text{otherwise} \end{cases},$$

  where $\boldsymbol{\theta}_l = (\eta_♀, \beta_♀, \eta_♂, \beta_♂)$. We assumed exponential priors $\eta_♀ \sim \mathrm{Exp}(\lambda_{\eta_♀})$, $\eta_♂ \sim \mathrm{Exp}(\lambda_{\eta_♂})$, $\beta_♀ \sim \mathrm{Exp}(\lambda_{\beta_♀})$ and $\beta_♂ \sim \mathrm{Exp}(\lambda_{\beta_♂})$.

- *Founder potential function* $f_f(h_f|\boldsymbol{\theta}_f)$
  We assume that the birth dates b_f of all $f \in \mathcal{F}$ follow a normal distribution $b_f \sim \mathcal{N}(\mu_f, \sigma_f^2)$ with mean μ_f and variance σ_f^2 such that

$$f_f(h_f|\boldsymbol{\theta}_f) = \begin{cases} \frac{1}{\sqrt{2\pi\sigma_f^2}} \exp\left(-\frac{1}{2\sigma_f^2}(b_i - \mu_f)^2\right) & \text{if } f \in \mathcal{F} \\ 1 & \text{otherwise} \end{cases},$$

  with $\boldsymbol{\theta}_f = (\mu_f, \sigma_f^2)$. We assumed a normal prior $\mu_f \sim \mathcal{N}(\mu_{\mu_f}, \sigma_{\mu_f}^2)$ on the mean and an exponential prior $\sigma_f^2 \sim \text{Exp}(\lambda_{\sigma_f^2})$ on the variance.

- *Parental potential function* $f_p(h_i, h_j|\boldsymbol{\theta}_p)$
  We define the age difference between mothers and fathers

$$\delta_b(i,j) = \begin{cases} b_i - b_j & \text{if } i \in \mathcal{S}_♀, j \in \mathcal{S}_♂ \\ b_j - b_i & \text{if } i \in \mathcal{S}_♂, j \in \mathcal{S}_♀ \end{cases},$$

  which is positive ($\delta_b(i,j) > 0$) if the mother was younger than the father. We then assume that $\delta_b(i,j) \sim \mathcal{N}(\mu_p, \sigma_p^2)$ follows a normal distribution with mean μ_p and variance σ_p^2 such that

$$f_p(h_i, h_j|\boldsymbol{\theta}_p) = \begin{cases} \left(\frac{1}{\sqrt{2\pi\sigma_p^2}} \exp\left(-\frac{1}{2\sigma_p^2}(\delta_b(i,j) - \mu_p)^2\right)\right)^{\frac{1}{2}} & \text{if } (i,j) \in \mathcal{P}, \\ 1 & \text{otherwise} \end{cases},$$

  with $\boldsymbol{\theta}_p = (\mu_p, \sigma_p^2)$ and where the square root is taken to reduce the weight of this potential function, reflecting that for each individual of a mother, father and offspring trio, not all pairwise birth date comparisons are independent (see also the offspring potential function below). We use improper priors $\mu_p \propto 1$ and $\sigma_p^2 \propto 1$ on both parameters.

- *Offspring potential function* $f_o(h_i, h_p|\boldsymbol{\theta}_o)$
  We distinguish between the sex of the parent:

$$f_o(h_i, h_p|\boldsymbol{\theta}_o) = \begin{cases} f_♀(h_i, h_p|\boldsymbol{\theta}_o)^{\frac{1}{2}} & \text{if } p = \mathcal{P}_♀(i) \\ f_♂(h_i, h_p|\boldsymbol{\theta}_o)^{\frac{1}{2}} & \text{if } p = \mathcal{P}_♂(i) \\ 1 & \text{otherwise.} \end{cases}$$

  As for the parental potential function, we use the square root to reflect that for a mother, father and offspring trio, not all pairwise birth date comparisons are independent.

  For the maternal offspring potential function $f_♀(h_i, h_p|\boldsymbol{\theta}_o)$, we assume that women are fertile (able to conceive) 9 months prior to the known age range $(\phi_♀, \chi_♀]$. For an offspring i with mother $p = \mathcal{P}_♀(i)$, the birth must thus lie within the interval $b_i \in (u_♀(h_p), v_♀(h_p)]$, where the lower bound

$$u_♀(h_p) = b_p + \phi_♀$$

is given by the date of the start of the fertility window and the upper bound

$$v_{♀}(h_p) = \min\left(b_p + \chi_{♀}, d_p\right)$$

reflects that b_i must predate both the end of the fertility window and the death of the mother.

We assume that the age $a_{♀}(h_i, h_p)$ of mother p at birth of offspring i relative to her fertility window follows an unimodal Beta distribution with shape parameters $1 + \kappa_{1♀}$ and $1 + \kappa_{2♀}$:

$$a_{♀}(h_i, h_p) = (b_i - b_p - \phi_{♀})/(\chi_{♀} - \phi_{♀}) \sim \text{Beta}(1 + \kappa_{1♀}, 1 + \kappa_{2♀}).$$

Under a strict interpretation, and denoting by $\text{I}(\cdot)$ the indicator function, we have

$$f_{♀}(h_i, h_p|\boldsymbol{\theta}_o) = \text{I}(b_i \in (u_{♀}(h_p), v_{♀}(h_p)]) \frac{a_{♀}(h_i, h_p)^{\kappa_{1♀}} \left[1 - a_{♀}(h_i, h_p)\right]^{\kappa_{2♀}}}{B(1 + \kappa_{1♀}, 1 + \kappa_{2♀})}.$$

However, such a strict definition does not lend itself easily for automated optimization. We therefore adopt a more permissive approach by using the mixture model

$$\begin{aligned} f_{♀}(h_o, h_p|\boldsymbol{\theta}_o) =& (1 - \pi_o)\text{I}(b_i \in (u_{♀}(h_p), v_{♀}(h_p)]) \frac{a_{♀}(h_i, h_p)^{\kappa_{1♀}} \left[1 - a_{♀}(h_i, h_p)\right]^{\kappa_{2♀}}}{B(1 + \kappa_{1♀}, 1 + \kappa_{2♀})} \\ &+ \pi_o \exp\left(-r_o \left(b_i - \frac{u_{♀}(h_p) + v_{♀}(h_p)}{2}\right)^2\right), \end{aligned}$$

where we set $\pi_o = 10^{-20}$ and $r_o = 0.1$ to ensure both numerical stability and that solutions with $b_i \notin (u_{♀}(h_p), v_{♀}(h_p)]$ get strongly penalized.

We proceed analogously for the paternal offspring potential function $f_{♂}(h_i, h_p|\boldsymbol{\theta}_o)$, using the known fertility range $(\phi_{♂}, \chi_{♂}]$, the bounds

$$u_{♂}(h_p) = b_p + \phi_{♂}, \quad v_{♂}(h_p) = \min\left(b_p + \chi_{♂}, d_p + \frac{9}{12}\right)$$

to reflect that fathers need to be alive at conception, but not at birth of their offspring, paternal shape parameters $\kappa_{1♂}$ and $\kappa_{2♂}$ and shared parameters $\pi_o = 10^{-20}$ and $r_o = 1$.

We have $\boldsymbol{\theta}_o = (\kappa_{1♀}, \kappa_{2♀}, \kappa_{1♂}, \kappa_{2♂})$ and use exponential priors $\kappa_{1♀} \sim \text{Exp}(\lambda_{\kappa_{1♀}})$, $\kappa_{2♀} \sim \text{Exp}(\lambda_{\kappa_{2♀}})$, $\kappa_{1♂} \sim \text{Exp}(\lambda_{\kappa_{1♂}})$ and $\kappa_{2♂} \sim \text{Exp}(\lambda_{\kappa_{2♂}})$.

- *Sibling potential function* $f_s(h_i, h_j|h_{\mathcal{P}_{♀}(i)}, \boldsymbol{\theta}_s)$
  We assume that the birth dates b_i and b_j of all pairs of siblings $(i, j) \in \mathcal{O}(m), m \in \mathcal{S}_{♀}, i \neq j$ with the same mother must lie at least nine months apart. We therefore penalize each sibling pair with birth dates closer than nine months using the permissive mixture

$$f_s(h_i, h_j|h_{\mathcal{P}_{♀}(i)}, \boldsymbol{\theta}_s) = (1 - \pi_s)\text{I}(|b_i - b_j| > \frac{9}{12}) + \pi_s \left(1 - \exp\left(-r_s\left(b_i - b_j\right)^2\right)\right),$$

where we set $\pi_s = 10^{-20}$ and $r_s = 10$ to ensure that solutions violating the nine month rule get strongly penalized while adequately guiding optimization.

- *Grave order constraints*
  We consider grave order constraints for several pairs of individuals $(i, j) \in \mathcal{T}$, of which individual i is known to have been buried prior to individual j. To aid in the parameter optimization, we use the permissive potential functions

$$f_T^{(ij)}(h_i, h_j) = f_T(h_i, h_j) = \mathrm{I}(d_i < d_j) + \pi_T \mathrm{I}(d_i \geq d_j)(1 - \exp(-r_T(d_i - d_j))).$$

  We set $\pi_T = 10^{-20}$ and $r_T = 1$ to ensure that solutions violating the constraint get strongly penalized while adequately guiding optimization.

Combining samples from multiple locations
The model described above assumes that all samples share the same potential functions. In case samples originate from multiple locations (e.g. multiple burial grounds), that assumption may be violated. However, it is straightforward to extend the model so that the parameters of select potential functions will be learned per location. In our implementation provided here, we allow for a location $\mathcal{L}(i) \in \mathcal{L}_1, \ldots, \mathcal{L}_L$ to be specified for each individual $i \in \mathcal{I}$, and then infer the parameters $\boldsymbol{\theta}_f^{(\mathcal{L}_l)}$ of the founder potential functions individually for each location $l \in \mathcal{L}_1, \ldots, \mathcal{L}_L$ to reflect that different burial grounds may have been used at different times. In this case, we infer the parameters μ_{μ_f}, $\sigma^2_{\mu_f}$ and $\lambda_{\sigma^2_f}$ using the improper, non-informative priors $\mu_{\mu_f} \propto 1$, $\sigma^2_{\mu_f} \propto 1$ on $(0, \infty)$ and $\lambda_{\sigma^2_f} \propto 1$ on $(0, \infty)$.

Data
While the model is flexible regarding the data used, we here consider three different types:

1. *Archaeological dating*
   We denote by $[m_i^{(g)}, n_i^{(g)}]$ the archaeologically estimated range of the date of death of individual $i \in \mathcal{I}$. We assume that this range reflects the $q^{(g)}$ and $1 - q^{(g)}$ quantiles of the posterior distribution $\mathbb{P}(d_i|g_i)$, where g_i denotes the archaeological information associated with the grave of individual i. We assume $\mathbb{P}(d_i|g_i)$ follows a normal distribution and that a non-informative prior $\mathbb{P}(d_i) \propto 1$ was used to obtain it. This implies that the likelihood $\mathbb{P}(g_i|d_i) \propto \mathbb{P}(d_i|g_i)$ and hence that

$$\mathbb{P}(g_i|d_i) \propto \frac{|2z_q^{(g)}|}{n_i^{(g)} - m_i^{(g)}} \exp\left(\frac{|z_q^{(g)}|}{n_i^{(g)} - m_i^{(g)}}(m_i^{(g)} + n_i^{(g)} - 2d_i)^2\right),$$

   where $|z_q^{(g)}|$ is the absolute z-score at q. For individuals for which no archaeological date was estimated, we have $\mathbb{P}(g_i|d_i) = 1$. Here, we used $z_q^{(g)} = 2$.

2. *Osteological age-at-death assessments*
   Analogously to the archaeological date, we will denote by $[m_i^{(a)}, n_i^{(a)}]$ the osteological estimate of the age-at-death of individual $i \in \mathcal{I}$ and assume this range reflects the $q^{(a)}$ and $1 - q^{(a)}$ quantiles of the posterior distribution $\mathbb{P}(l_i|r_i)$ on the lifespan $l_i = d_i - b_i$, where r_i denotes the remains of individual i. We assume $\mathbb{P}(l_i|r_i)$ follows a normal distribution and that a non-informative prior $\mathbb{P}(l_i) \propto 1$ was used to obtain it. This implies that the likelihood $\mathbb{P}(r_i|l_i) = \mathbb{P}(r_i|b_i, d_i) \propto \mathbb{P}(r_i|l_i)$ and calculated analogously to that of the archaeological date. For individuals without assessed remains, $\mathbb{P}(r_i|b_i, d_i) = 1$. Here, we used $z_q^{(a)} = 2$.

3. *^{14}C dates*

Let C_i denote the ^{14}C data (raw date and sample variance) obtained for individual i. There are two challenges associated with raw ^{14}C data:

Carbon turnover in tissue: The first challenge is that the radiocarbon present in a tissue sample may originate from different life stages depending on carbon turnover in that tissue. Consider an individual $i \in \mathcal{I}$ born on date b_i that dies on date d_i. We seek to calculate the date $t_{ir}^{(14)}$ relevant for a collagen sample taken from a bone of type r of that individual.

As was proposed previously [176], we model collagen turnover in discrete years. Let $\rho_r(a)$ denote the collagen turnover for a bone sample of type r at age a in discrete years. For a collagen sample of a bone of type r taken after death of an individual with discrete lifespan $\ell = 0, 1, 2, \ldots$ years, the fraction $p_r(a; \ell)$ of collagen that was incorporated at age a is given by

$$p_r(a; b_i, d_i) = \begin{cases} \rho_r(\ell) & \text{if } a = \ell \\ \rho_r(a) \prod_{t=a+1}^{\ell} (1 - \rho_r(t)) & \text{if } 0 < a < \ell \\ \prod_{t=1}^{\ell} (1 - \rho_r(t)) & \text{if } a = 0 \end{cases}.$$

The average age of the incorporated collagen for an individual with discrete lifespan ℓ is then

$$\bar{a}_r(\ell) = \sum_{a=0}^{\ell} a p_r(a; b_i, d_i).$$

To calculate the continuous relevant date $t_{ir}^{(14)}$ for a collagen sample of type r of individual $i \in \mathcal{I}$ with continuous lifespan $l_i = d_i - b_i$, we then use linear interpolation:

$$t_{ir}^{(14)}(b_i, d_i) = b_i + \bar{a}_r(\lfloor l_i \rfloor) + (l_i - \lfloor l_i \rfloor) \left[\bar{a}_r(\lceil l_i \rceil) - \bar{a}_r(\lfloor l_i \rfloor) \right].$$

While a fully continuous model could be envisioned (that would not need to be interpolated), the discrete model adopted here allows us to benefit from previous estimates of turnover rates. As was proposed previously [176], we model per year collagen turnover rates $\rho_r(a)$ using piecewise linear functions defined by a series of $M^{(r)}$ changepoints $(a_{r1}, \rho_{r1}), (a_{r2}, \rho_{r2}), \ldots, (a_{rM}, \rho_{rM})$ assumed to be known. While our implementation is fully flexible regarding the choice of curves, we here use (Figure S3.2):

- For *long bone* (*femur*) samples, we used the curve previously reported [176], defined through the changepoints (0, 0.5), (17, 0.137), (25, 0.030) and (100, 0.015) for men, and (0, 0.5), (15, 0.060), (19, 0.041) and (100, 0.03) for women.
- For collagen samples from *rib* bones (*costae*), the average age is assumed to date to about five years prior to death [177,178,179]. We therefore used a curve defined by the changepoints (0, 0.5), (15,0.2) and (100,0.2) for both sexes to reflect faster turnover of children and the stable turnover among adults leading to collagen predating death by five years.

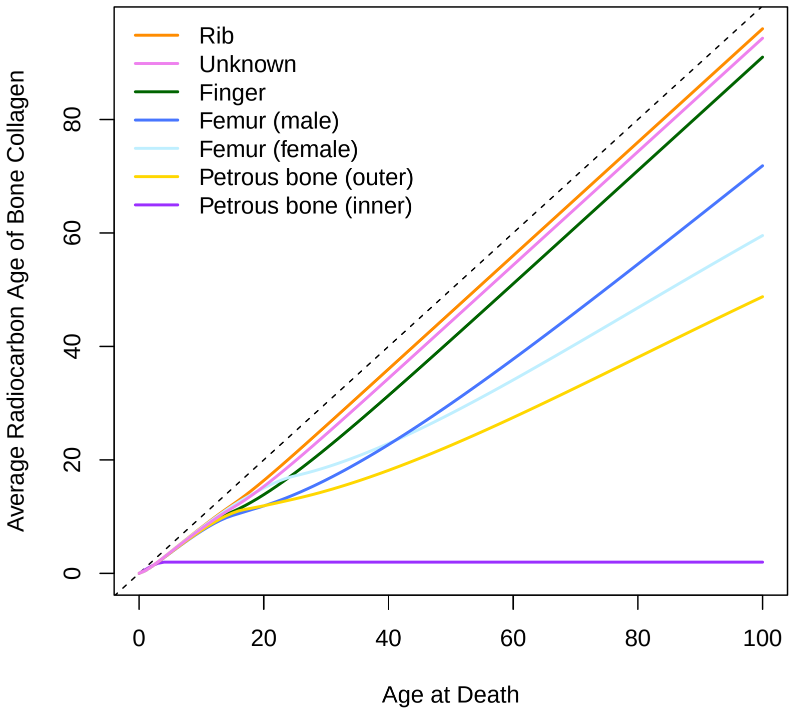

Fig. S3.2: *^{14}C turnover rates used for different types of bones. See text for details on the model.*

- Little is known about turnover rates for finger bones (*phalanges*) and we decided to adopt the curve somewhere between femur and rib, defined by the changepoints (0, 0.5), (15, 0.1) and (100,0.1) for both sexes.
- For the inner petrous bone (*pars petrosa*), it is believed that turnover stops at age two [178]. We therefore use a curve defined by the changepoints (0, 0.5), (3, 0.5), (4, 0) and (100, 0) for both sexes.
- For the outer petrous bone, we assumed turnover is faster than for the inner petrous bone, but still slower than for femurs. We therefore use a curve defined by the changepoints (0, 0.5), (17, 0.03), (25, 0.02) and (100, 0.01) for both sexes.
- For some samples, the bone that was originally used for dating is unknown. We assume that the most commonly used bones prior to aDNA work were ribs and *phalanges*, and hence use a curve intermediate between these bones with changepoints (0,0.5), (15,0.15) and (100, 0.15) for *unknown* bones.

Calibration: The second challenge is that the raw ^{14}C date needs to be calibrated due to changes in the proportion of radiocarbon in the atmosphere, among other effects. The calibration of the raw ^{14}C date is done against a so-called calibration curve $f_{^{14}C}(t)$ and we denote by $\mathbb{P}(C_i|t, f_{^{14}C}(t))$ the likelihood of observing measurements C_i at date t given the used calibration curve. To incorporate these likelihoods into the dating model proposed above, we assume that the likelihoods have been pre-calculated for each sample $i \in \mathcal{I}$ with ^{14}C data C_i at pre-defined time

points $t_1, t_2, \ldots, t_K$ using an external software. Here, we used the `R_Date` function in OxCal [180] with the IntCal20 calibration curve [181] using steps of 1/12 of a year. We then determine $\mathbb{P}(C_i|t, f_{^{14}C}(t))$ by linear interpolation as

$$\mathbb{P}(C_i|t, f_{^{14}C}(t)) = \begin{cases} \mathbb{P}(C_i|t_k, f_{^{14}C}(t_k)) + \frac{\mathbb{P}(C_i|t_k, f_{^{14}C}(t_{k+1})) - \mathbb{P}(C_i|t_k, f_{^{14}C}(t_k))}{t_{k+1}-t_k}(t - t_k) & \text{if } t_k < t < t_{k+1}, \\ 10^{-10} & \text{if } t < t_1 \text{or } t > t_K. \end{cases}$$

The full likelihood of the ^{14}C data is then given by $\mathbb{P}(C_i|b_i, d_i) = \mathbb{P}(C_i|t_{ir}^{(14)}(b_i, d_i), f_{^{14}C}(t))$.

We will denote by $\mathbb{P}(D_i|b_i, d_i) = \mathbb{P}(g_i|d_i)\mathbb{P}(r_i|b_i, d_i)\mathbb{P}(^{14}C_i|b_i, d_i)$ the full probability of all observed data for individual i.

Implementation

We implemented MCMC inference via Metropolis-Hastings updates for the proposed model in C++ using the `stattools` library (`https://bitbucket.org/wegmannlab/stattools`, commit `2e0bd28`), which provides many useful features for implementing fast MCMC samplers, including an adaptive scheme consisting of a specified amount of burn-ins after which the proposal kernels are tuned to guarantee optimal performance. The implementation of this model is available through the software `Chronograph`, a `git`-repository which is found at `https://bitbucket.org/wegmannlab/chronograph`, along with a wiki detailing its usage. For this work, we used commit `affa83f`.

Parameter updates and Hastings ratios

- *Parameters regarding life histories h_i*

  To ensure proper mixing, we update life history parameters using two updates:

  1. We propose values $h_i = (b_i, d_i, l_i) \to h'_i = (b'_i, d'_i, l_i)$ with $b'_i \sim \mathcal{N}(b_i, \sigma_b^2)$ and $d'_i = b'_i + l_i$. For a founder $i \in \mathcal{F}$, the proposed value b'_i is accepted with probability

$$h_{b_i} = \min\left(1, \frac{\pi(D_i|h'_i, \boldsymbol{\theta}_D) f_f(h'_i|\boldsymbol{\theta}_f)}{\pi(D_i|h_i, \boldsymbol{\theta}_D) f_f(h_i|\boldsymbol{\theta}_f)} \prod_{j,(i,j)\in\mathcal{P}} \frac{f_p(h'_i, h_j|\boldsymbol{\theta}_p)}{f_p(h_i, h_j|\boldsymbol{\theta}_p)} \times \prod_{o\in\mathcal{O}(i)} \frac{f_o(h_o, h'_i|\boldsymbol{\theta}_o)}{f_o(h_o, h_i|\boldsymbol{\theta}_o)} \prod_{(i,j)\in\mathcal{T}} \frac{f_T^{(i,j)}(h'_i, h_j)}{f_T^{(i,j)}(h_i, h_j)}\right),$$

  where the product for $f_p(\cdot)$ runs across all pairs of parents with founder i and the product across $f_T(\cdot)$ across all pairs of individuals with grave order constraints with founder i.

For an offspring $i \in \mathcal{O}$, the proposed value h'_i is accepted with probability

$$h_{b_i} = \min\left(1, \frac{\pi(D_i|h'_i, \boldsymbol{\theta}_D) f_o(h'_i, h_{\mathcal{P}_♀(i)}|\boldsymbol{\theta}_o) f_o(h'_i|h_{\mathcal{P}_♂(i)}, \boldsymbol{\theta}_o)}{\pi(D_i|h_i, \boldsymbol{\theta}_D) f_o(h_i, h_{\mathcal{P}_♀(i)}|\boldsymbol{\theta}_o) f_o(h_i|h_{\mathcal{P}_♂(i)}, \boldsymbol{\theta}_o)} \prod_{j,(i,j)\in\mathcal{P}} \frac{f_p(h'_i, h_j|\boldsymbol{\theta}_p)}{f_p(h_i, h_j|\boldsymbol{\theta}_p)}\right.$$

$$\left.\times \prod_{o\in\mathcal{O}(i)} \frac{f_o(h_o|h'_i, \boldsymbol{\theta}_o)}{f_o(h_o|h_i, \boldsymbol{\theta}_o)} \prod_{\substack{s\in\mathcal{O}(\mathcal{P}_♀(i))\\ s\neq i}} \frac{f_s(h'_i, h_s|h_{\mathcal{P}_♀(i)}, \boldsymbol{\theta}_s)}{f_s(h_i, h_s|h_{\mathcal{P}_♀(i)}, \boldsymbol{\theta}_s)} \prod_{(i,j)\in\mathcal{T}} \frac{f_T^{(i,j)}(h'_i, h_j)}{f_T^{(i,j)}(h_i, h_j)}\right),$$

where the product for $f_p(\cdot)$ runs across all pairs of parents that include offspring i, the product across $f_s(\cdot)$ runs across all siblings of offspring i and the product across $f_T(\cdot)$ across all pairs of individuals with grave order constraints that include offspring i.

2. We propose new values $h_i = (b_i, d_i, l_i) \to h'_i = (b_i, d'_i, l'_i)$ with $l'_i \sim \mathcal{N}(l_i, \sigma^2_{l_i})$ and $d'_i = b_i + l'_i$. The proposed values are accepted with probability

$$h_{b_i} = \min\left(1, \frac{\pi(D_i|h'_i, \boldsymbol{\theta}_D) f_l(h'_i|\boldsymbol{\theta}_l)}{\pi(D_i|h_i, \boldsymbol{\theta}_D) f_l(h_i|\boldsymbol{\theta}_l)} \prod_{o\in\mathcal{O}(i)} \frac{f_o(h_o, h'_i|\boldsymbol{\theta}_o)}{f_o(h_o, h_i|\boldsymbol{\theta}_o)} \prod_{(i,j)\in\mathcal{T}} \frac{f_T^{(i,j)}(h'_i, h_j)}{f_T^{(i,j)}(h_i, h_j)}\right),$$

where the latter product runs across all pairs of individuals (i, j) with grave order constraints that include offspring i.

- *Parameter $\boldsymbol{\theta}_l$ regarding the lifespan potential function $f_l(\cdot)$*

  – We update $\eta_♀$ with proposals $\boldsymbol{\theta}_l = (\eta_♀, \beta_♀, \eta_♂, \beta_♂) \to \boldsymbol{\theta}'_l = (\eta'_♀, \beta_♀, \eta_♂, \beta_♂)$ with $\eta'_♀ \sim \mathcal{N}(\eta_♀, \sigma^2_{\eta_♀})$, mirrored at 0 to respect $\eta_♀ > 0$, and accept with probability

$$h_{\eta_♀} = \min\left(1, \frac{\mathbb{P}(\eta'_♀)}{\mathbb{P}(\eta_♀)} \prod_{i\in\mathcal{S}_♀} \frac{f_l(h_i|\boldsymbol{\theta}'_l)}{f_l(h_i|\boldsymbol{\theta}_l)}\right).$$

  – We update $\eta_♂$ analogously with proposal kernel $\eta'_♂ \sim \mathcal{N}(\eta_♂, \sigma^2_{\eta_♂})$ and Hastings ratio

$$h_{\eta_♂} = \min\left(1, \frac{\mathbb{P}(\eta'_♂)}{\mathbb{P}(\eta_♂)} \prod_{i\in\mathcal{S}_♂} \frac{f_l(h_i|\boldsymbol{\theta}'_l)}{f_l(h_i|\boldsymbol{\theta}_l)}\right).$$

  – We update $\beta_♀$ with proposals $\boldsymbol{\theta}_l = (\eta_♀, \beta_♀, \eta_♂, \beta_♂) \to \boldsymbol{\theta}'_l = (\eta_♀, \beta'_♀, \eta_♂, \beta_♂)$ with $\beta'_♀ \sim \mathcal{N}(\beta_♀, \sigma^2_{\beta_♀})$, mirrored at 0 to respect the constraint $\beta_♀ > 0$ and accept with probability

$$h_{\beta_♀} = \min\left(1, \frac{\mathbb{P}(\beta'_♀)}{\mathbb{P}(\beta_♀)} \prod_{i\in\mathcal{S}_♀} \frac{f_l(h_i|\boldsymbol{\theta}'_l)}{f_l(h_i|\boldsymbol{\theta}_l)}\right).$$

– We update $\beta_{♂}$ analogously with proposal kernel $\beta'_{♂} \sim \mathcal{N}(\beta_{♂}, \sigma^2_{\beta_{♂}})$ and Hastings ratio

$$h_{\beta_{♂}} = \min\left(1, \frac{\mathbb{P}(\beta'_{♂})}{\mathbb{P}(\beta_{♂})} \prod_{i \in \mathcal{S}_{♂}} \frac{f_l(h_i|\boldsymbol{\theta}'_l)}{f_l(h_i|\boldsymbol{\theta}_l)}\right).$$

- *Parameter $\boldsymbol{\theta}_p$ regarding the parental potential function $f_p(\cdot)$*
  - We update μ_p with proposals $\boldsymbol{\theta}_p = (\mu_p, \sigma_p^2) \to \boldsymbol{\theta}'_p = (\mu'_p, \sigma_p^2)$ with $\mu'_p \sim \mathcal{N}(\mu_p, \sigma^2_{\mu_p})$ and accept with probability

$$h_{\mu_p} = \min\left(1, \frac{\mathbb{P}(\mu'_p)}{P(\mu_p)} \prod_{(i,j) \in \mathcal{P}} \frac{f_p(h_i, h_j|\boldsymbol{\theta}'_p)}{f_p(h_i, h_j|\boldsymbol{\theta}_p)}\right)$$

  - The update for σ_p^2 is analogous, but we propose new values $\boldsymbol{\theta}_p = (\mu_p, \sigma_p^2) \to \boldsymbol{\theta}'_p = (\mu_p, \sigma_p^{2\prime})$ with $\sigma_p^{2\prime} \sim \mathcal{N}(\sigma_p^2, \sigma^2_{\sigma_p^2})$, mirrored at 0 to respect the constraint $\sigma_p^2 > 0$

- *Parameters $\boldsymbol{\theta}_o$ regarding the offspring potential function $f_o(\cdot)$*
  - We update $\kappa_{1♀}$ with proposals $\boldsymbol{\theta}_o = (\kappa_{1♀}, \kappa_{2♀}, \kappa_{1♂}, \kappa_{2♂}) \to \boldsymbol{\theta}'_o = (\kappa'_{1♀}, \kappa_{2♀}, \kappa_{1♂}, \kappa_{2♂})$ with $\kappa'_{1♀} \sim \mathcal{N}(\kappa_{1♀}, \sigma^2_{\kappa_{1♀}})$, mirrored at 0 to respect $\kappa_{1♀} > 0$, and accept with probability

$$h_{\kappa_{1♀}} = \min\left(1, \frac{\mathbb{P}(\alpha')}{\mathbb{P}(\alpha)} \prod_{i \in \mathcal{O}} \frac{f_o(h_i, h_{\mathcal{P}_♀(i)}|\boldsymbol{\theta}'_o)}{f_o(h_i, h_{\mathcal{P}_♀(i)}|\boldsymbol{\theta}_o)}\right) = \min\left(1, \frac{\mathbb{P}(\alpha')}{\mathbb{P}(\alpha)} \prod_{i \in \mathcal{O}} \frac{f_♀(h_i, h_{\mathcal{P}_♀(i)}|\boldsymbol{\theta}'_o)}{f_♀(h_i, h_{\mathcal{P}_♀(i)}|\boldsymbol{\theta}_o)}\right).$$

  - Updates for $\kappa_{1♂}$ are analogous: we propose new values $\boldsymbol{\theta}_o = (\kappa_{1♀}, \kappa_{2♀}, \kappa_{1♂}, \kappa_{2♂}) \to \boldsymbol{\theta}'_o = (\kappa_{1♀}, \kappa_{2♀}, \kappa'_{1♂}, \kappa_{2♂})$ with $\kappa'_{1♂} \sim \mathcal{N}(\kappa_{1♂}, \sigma^2_{\kappa_{1♂}})$, mirrored at 0 to respect the constraint $\kappa_{1♂} > 0$, and accept updates with probability

$$h_{\kappa_{1♂}} = \min\left(1, \frac{\mathbb{P}(\alpha')}{\mathbb{P}(\alpha)} \prod_{i \in \mathcal{O}} \frac{f_o(h_i, h_{\mathcal{P}_♂(i)}|\boldsymbol{\theta}'_o)}{f_o(h_i, h_{\mathcal{P}_♂(i)}|\boldsymbol{\theta}_o)}\right) = \min\left(1, \frac{\mathbb{P}(\alpha')}{\mathbb{P}(\alpha)} \prod_{i \in \mathcal{O}} \frac{f_♂(h_i, h_{\mathcal{P}_♂(i)}|\boldsymbol{\theta}'_o)}{f_♂(h_i, h_{\mathcal{P}_♂(i)}|\boldsymbol{\theta}_o)}\right).$$

  - We update $\kappa_{2♀}$ and $\kappa_{2♂}$ analogously with $\kappa'_{2♀} \sim \mathcal{N}(\kappa_{2♀}, \sigma^2_{\kappa_{2♀}})$ and $\kappa'_{2♀} \sim \mathcal{N}(\kappa_{2♀}, \sigma^2_{\kappa_{2♀}})$.

- *Parameters $\boldsymbol{\theta}_f$ regarding the founder potential function $f_f(\cdot)$*
  - We update μ_f with proposals $\boldsymbol{\theta}_f = (\mu_f, \sigma_f^2) \to \boldsymbol{\theta}_f = (\mu'_f, \sigma_f^2)$ with $\mu'_f \sim \mathcal{N}(\mu_f, \sigma^2_{\mu_f})$ and accept with probability

$$h_{\mu_p} = \min\left(1, \prod_{i \in \mathcal{F}} \frac{f_f(h_i|\boldsymbol{\theta}'_f)}{f_f(h_i|\boldsymbol{\theta}_f)}\right).$$

  - The update for σ_f^2 is analogous, but we propose new values with $\boldsymbol{\theta}_f = (\mu_f, \sigma_f^2) \to \boldsymbol{\theta}_f = (\mu_f, \sigma_f^{2\prime})$ with $\sigma_f^{2\prime} \sim \mathcal{N}(\sigma_f^2, \sigma^2_{\sigma_f^2})$, mirrored at 0 to respect the constraint $\sigma_f^2 > 0$.

Initialization
Prior to running the MCMC algorithm, we initialize parameters with initial guesses as follows:

- *Death dates* d_i

  1. For individuals with ^{14}C data, set death dates to the calibrated peak likelihood year, offset by 10 years to account for collagen age
  2. For individuals with only archaeological context, set death dates to the mid point of their archaeological range. Calculate mean and standard deviation of all initialized death dates.
  3. Ensure compliance with death order constraints by adjusting violating dates.
  4. Loop over uninitialized individuals like this:
     - In case of initialized partners, set death date to the mean of their partners' death dates.
     - In case of initialized parents and offspring, set death date to the average of their means.
     - In case of initialized offspring only, set death date to the mean of their offspring's death dates, offset by the midpoint of their fertility window.
     - In case of initialized parents only, set death date to their mean plus 20 years.
     - In all cases, ensure compliance with death order constraints.
  5. Repeat this loop until no new individuals are being initialized.
  6. For uninitialized founders, group parents connected by offspring and sample their death dates from the normal distribution estimated in step 2.
  7. Perform a final top-down pass to initialize remaining offspring.
  8. Re-validate death order constraints.

- *Birth dates* b_i

  1. For individuals with age-at-death data, initialize birth date by subtracting the midpoint of the age span from the death date.
  2. Loop over all uninitialized individuals like this:
     - In case of initialized offspring, center the fertility window on their mean birth date.
     - In case of initialized partners, set the birth date to their mean birth dates.
     - Otherwise, set birth date to 50 years before death date.
  3. Adjust parental death dates to ensure they live until fertility begins.
  4. For parents, ensure overlapping fertility windows by iteratively adjusting birth and death dates toward their midpoint. Ensure all children's birth dates fall within that new window.
  5. For each sibling pair, ensure a minimum birth spacing of 9 months.

- *Lifespans* l_i

  After initializing birth and death rates, we initialize lifespans as $l_i = d_i - b_i$.

- *Parameter $\boldsymbol{\theta}_l$ regarding the lifespan potential function $f_p(\cdot)$*

  Using the initialized birth and death dates, we estimate individual lifespans and fit the parameters $\boldsymbol{\theta}_l = (\eta_♀, \beta_♀, \eta_♂, \beta_♂)$ via Nelder-Mead optimization to minimize the negative log-likelihood of the Gompertz distribution for each sex.

- *Parameters $\boldsymbol{\theta}_f$ regarding the founder potential function $f_f(\cdot)$*

  Set $\boldsymbol{\theta}_f = (\mu_f, \sigma_f^2)$ to the mean and standard deviation founder's initial birth date estimates.

- *Parameter $\boldsymbol{\theta}_p$ regarding the parental potential function $f_p(\cdot)$*

  Initialize $\boldsymbol{\theta}_p = (\mu_p, \sigma_p^2)$ to the mean and standard deviation of the age differences between partners, based on the initial birth dates.

- *Parameters $\boldsymbol{\theta}_o$ regarding the offspring potential function $f_o(\cdot)$*

  To initialize the parameters $\kappa_{1♀}$ and $\kappa_{2♀}$, we first calculate the sex-specific mean $\mu_{a♀}$ and variance $\sigma_{a♀}^2$ of the relative maternal age at birth $a_♀(h_i, h_p)$ across all pairs (p, i) of mothers p and offspring i. We then use the standard Method of Moments estimators

$$\kappa_{1♀} = \max\left[0, \mu_{a♀}\left(\frac{\mu_{a♀}(1-\mu_{a♀})}{\sigma_{a♀}^2} - 1\right) - 1\right],$$
$$\kappa_{2♀} = \max\left[0, (1-\mu_{a♀})\left(\frac{\mu_{a♀}(1-\mu_{a♀})}{\sigma_{a♀}^2} - 1\right) - 1\right],$$

  ensuring that all shape parameters are ≥ 0. The initialization of $\kappa_{1♂}$ and $\kappa_{2♂}$ is analogous.

Inference

Choice of prior distributions

- *Parameters $\boldsymbol{\theta}_l$ regarding the lifespan potential function $f_l(\cdot)$*

  We chose roughly uninformative priors by setting $\lambda_{\eta_♀} = \lambda_{\eta_♂} = \lambda_{\beta_♀} = \lambda_{\beta_♂} = 10^{-5}$.

- *Parameters $\boldsymbol{\theta}_f$ regarding the founder potential function $f_f(\cdot)$*

  We infer location-specific parameters $\boldsymbol{\theta}_f^{(\mathcal{L}_l)}$ using non-informative prior described above.

- *Choice of fertility windows $(\phi_♀, \chi_♀]$ and $(\phi_♂, \chi_♂]$*

  We set the upper bounds to $\chi_♀ = 50$ and $\chi_♂ = 90$ to reflect biological limits on fertility at older age. The lower bounds reflect both biological limits at young age, along with social practice. There is no reliable data available for the age at menarche for the relevant period and modern data likely underestimates that age as the age at menarche appears to have decreased since the beginning of the 20th century CE in many European countries [183,182]. To get insights into the likely pre-industrial age at menarche for our region, we relied on the oldest available data for Germany, which dates to the 19th century CE and was compiled by Lehmann *et al.* [182] (Table S3.1). Following [182], we assumed these ages to be roughly normally distributed and obtained maximum likelihood estimates of the mean and variance of a shared underlying

distribution across all data sets (Table S3.1). To obtain a conservative estimate of the earliest age at first birth, we calculated the lower 5% quantile of the resulting distribution (13.4 years) and added the average human gestation period of nine months, yielding an estimated minimum age of 14 years. Historical evidence, however, indicates that marriage, and possibly first reproduction, occurred at younger ages than suggested by nineteenth-century records. Multiple sources [184,185,186,187,188,189,190] cite age 12 as a marriage age, an age also supported by several historical documents (e.g. *Lex Salica*; *Capitulare missorum generale*, a. 802, c. 2; rotulus of the *Archives départementales des Bouches-du-Rhône 6 G 1*). This implies a minimum possible age at first birth of 12.75 years (12 years + 9 months gestation). However, the data do not allow reliable conclusions about the timing of menarche or about how promptly reproduction followed marriage. The onset of reproductive capacity therefore remains uncertain. Given this uncertainty, we use the conservative assumptions of $\phi_{♀} = \phi_{♂} = 14$ in our primary analyses. For completeness, we also report results using the earlier onset $\phi_{♀} = \phi_{♂} = 12.75$ below.

- *Parameters $\boldsymbol{\theta}_o$ regarding the offspring potential function $f_o(\cdot)$*

  We used $\lambda_{\kappa_{1♀}} = \lambda_{\kappa_{2♀}} = \lambda_{\kappa_{1♂}} = \lambda_{\kappa_{2♂}} = 0.1$. This implies that the resulting prior on the age at birth gives reduced weight to very young ages.

  Let $a'_p = b_i - b_p$ denote the absolute age of a mother p born at b_p when giving birth to child i, such that $b_i = b_p + a'_p$. The prior on a'_p is then given by

$$\mathbb{P}(a'_p|\kappa_{1♀}, \kappa_{2♀}) = \int\int f_{♀}(a_{♀}(b_p + a'_p, b_p)|\boldsymbol{\theta}_o)\mathbb{P}(\kappa_{1♀}|\lambda_{\kappa_{1♀}})\mathbb{P}(\kappa_{2♀}|\lambda_{\kappa_{2♀}})d_{\kappa_{1♀}}d_{\kappa_{2♀}}$$

  For our main choice $(\phi_{♀}, \chi_{♀}] = (14, 50]$, this results in $\mathbb{P}(a'_p > 15|\kappa_{1♀}, \kappa_{2♀}) = 0.983$ and $\mathbb{P}(a'_p > 20|\kappa_{1♀}, \kappa_{2♀}) = 0.857$. For our less restrictive choice $(\phi_{♀}, \chi_{♀}] = (12.75, 50]$, these probabilities are $\mathbb{P}(a'_p > 15|\kappa_{1♀}, \kappa_{2♀}) = 0.959$ and $\mathbb{P}(a'_p > 20|\kappa_{1♀}, \kappa_{2♀}) = 0.827$.

Table S3.1: *Distributions on the age at menarche reported for European communities of the 19th century CE compiled by [182]. The 5% and 95% quantiles were obtained from the reported mean and standard deviation under the assumption of a normal distribution.*

Year	Sample size	Mean	Standard deviation	Reference	5% quantile	95% quantile
1848	3,000	17.87	2.34	[183]	14.0	21.7
1850	2,439	16.92	1.99	[184]	13.6	20.2
1864	3,114	17.30	2.15	[185]	13.8	20.8
1865	3,000	16.50	2.32	[186]	12.7	20.3
1875	2,135	16.76	2.00	[184]	13.5	20.0
Combined	13,688	16.73	2.17	—	13.4	20.8

MCMC runs

We ran our method jointly on all samples from Altheim, Burgweinting, Büttelborn, Eltville, Ergoldsbach, Mattsies, Michaelsbuch, Mömlingen, Weilheim, Wölfersheim and Zschernitzsch that either had sequence data or were implied by the pedigree. However, we removed all individuals with only archaeological or osteological information as we cannot exclude that they are in fact individuals also implied by the pedigree. The archaeological dates and osteological age-at-death estimates are given in External Data Table 1. The few ambiguous cases were interpreted as described in Table S3.2.

We ran our main analysis (A_1) with $\phi_{♀} = \phi_{♂} = 14$, and conducted two additional analyses: A_2 without any pedigree information (and hence limited to sequenced individuals) and A_3 with $\phi_{♀} = \phi_{♂} = 12.75$. We ran each analysis with shared hyper parameters across sites, except for $\boldsymbol{\theta}_f$ of the founder potential function, which we inferred for each site individually (see above). We generated $M = 20,000$ posterior samples using 20 independent MCMC chains, each with 100 burn-ins of 10^3 iterations followed by a total of $2 \cdot 10^7$ iterations, of which we kept every 20,000th iteration (a thinning of 20,000). We then used all $M = 20,000$ life history posterior samples to account for the uncertainty regarding the dating in downstream analyses throughout the manuscript.

Fig. S3.3 illustrates the benefit of including pedigree information by comparing dating accuracy (span of the 90% credible interval) between our main run A_1 and run A_2. Figures S3.4-S3.6 show inferred individual life histories and S3.7 the estimated number of sequenced individuals alive through time, both obtained with our main run A_1. Figures S3.8-S3.11 visualize life history summaries obtained under both A_1 and A_3. All input files, traces and scripts to analyze and plot are available at Zenodo: `https://doi.org/10.5281/zenodo.17192653`.

Table S3.2: *Ambiguities in archaeological dating or osteological age-at-death assessments. For few samples, the data did not consist of a single range. Here we show how we interpreted these cases and list one of the individuals concerned. See External Data Table 1 for the full data and its interpretation.*

Archaeological Ambiguity	Interpreted as	Example
before 575 (stratigraphy)	missing data	Alh_124
600/10 - 630/40 or 630/40 - 670/80	600-680	EV1
540/50 - 570/80	540-580	EV2
700	675-725	Mln28a
550 - 560/70	550-570	Bur1
Early Middle Ages; 1st half of 6th century; SD 4 (510–530) - SD 5 (530-555)	510-550	W128
Early Middle Ages; 1st half of 6th century	501-550	W126
7th/ 8th century	601-800	Mat1
Age-at-Death Ambiguity	Interpreted as	Example
50+ years	50-75	Alh_114
≥ 60	60-80	Btb71
60+	60-80	Mln13
adult	19-80	Mln34
20+	20-80	Wh12
40-50 (Kat.Nr. 103-1), 46-60 (Kat.Nr. El. 103), 64-66 (Kat.Nr. E. 103)	40-66	EV2
30 - 40 (/50) years	30-50	ErgDF3

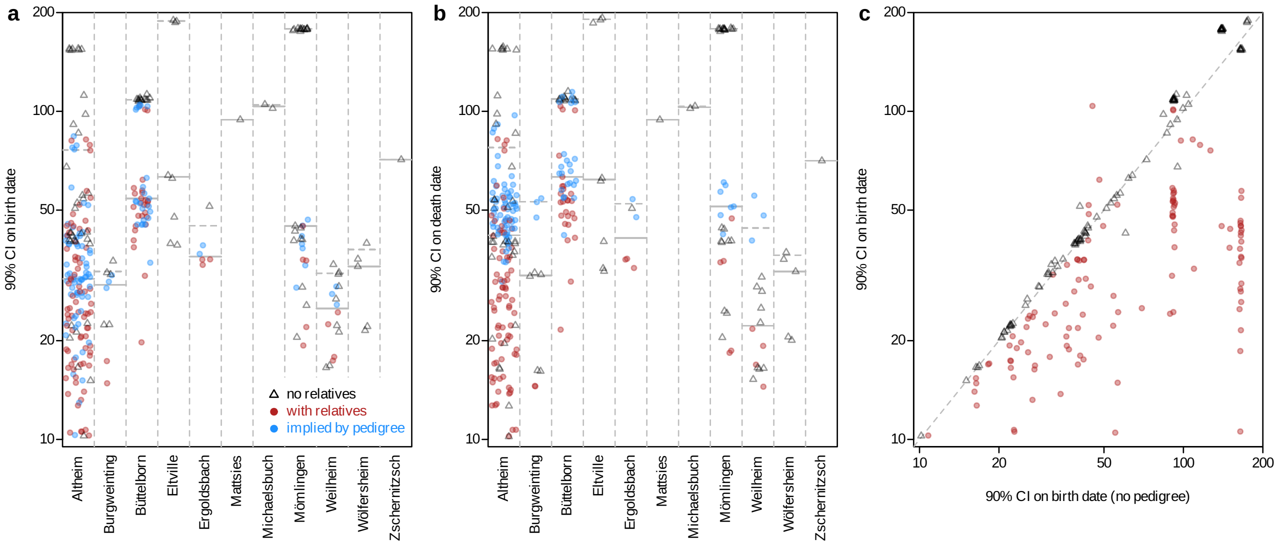

Fig. S3.3: *Estimation accuracy.* ***a*** *and* ***b****) Shown are the 90% CI on the birth (**a**) and death (**b**) dates per individual per site. Symbols and color distinguish between sampled individuals without relatives ("no relatives", black open triangles), sampled individuals with relatives ("with relatives", red circles) and unsampled individuals implied by pedigree information ("implied by pedigree", blue circles). Horizontal bars indicate the 50% and 90% quantile for each site. Confidence intervals are generally much narrower for samples with relatives, i.e. for individuals whose dates are constrained by that of other individuals. This is also true for individuals that were not sampled but only implied by the pedigree, whose CI were generally larger than for sampled individuals with relatives, but still smaller than for sampled individuals without relatives.* ***c****) The 90% CI on the birth date for the full run (y-axis, as in a) against a run that ignores pedigree information (x-axis). The strong reduction in 90% CI for most individuals with relatives illustrates the strong benefit of using pedigree information. The one outlier with strongly increased 90% CI is individual Btb73, for which pedigree information renders secondary peaks of its 14C likelihood function more plausible. Note that without pedigree information, no estimation is possible on the "implied" individuals.*

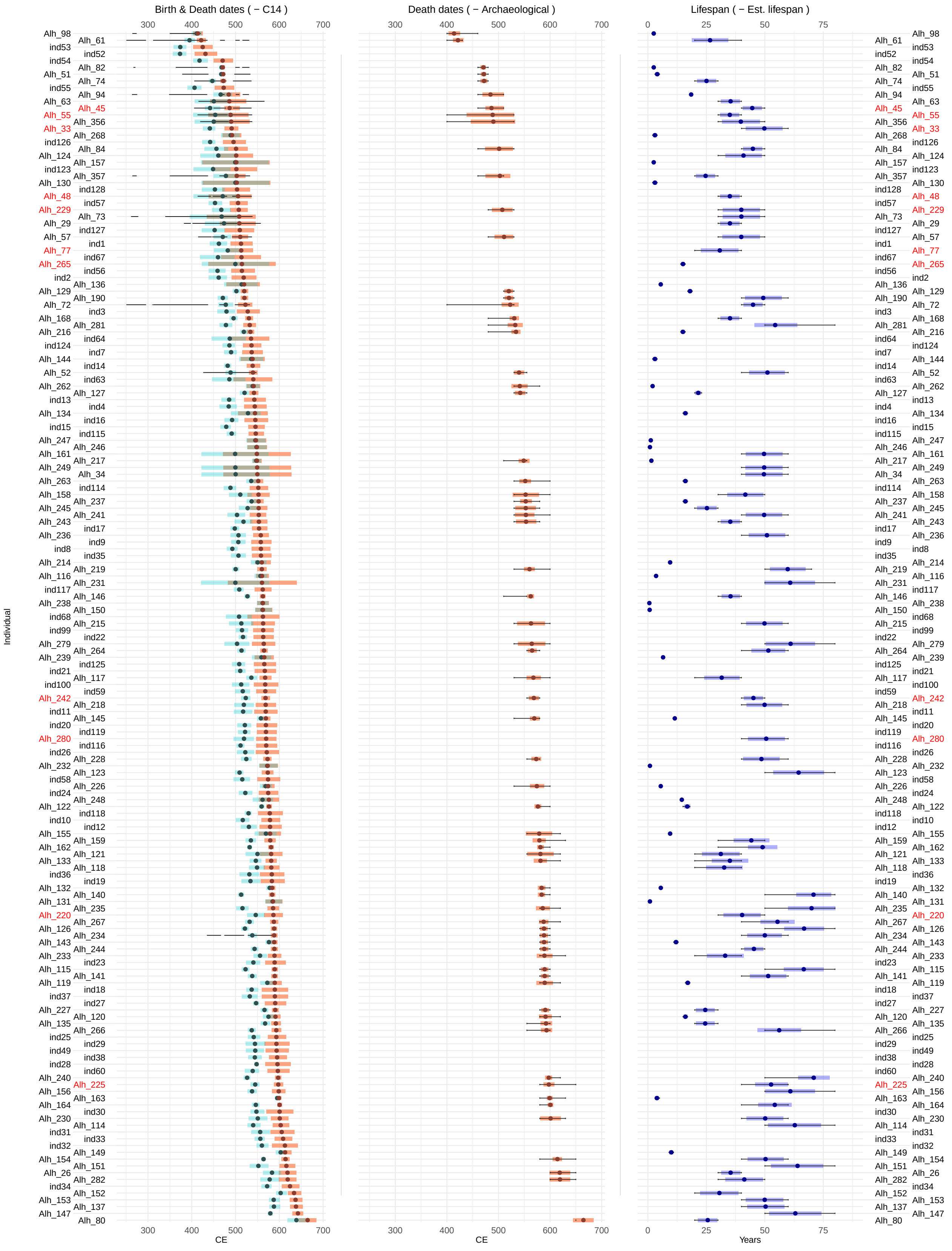

***Fig. S3.4:** Life history estimates for individuals identified at Altheim. See Fig. S3.6 for details.*

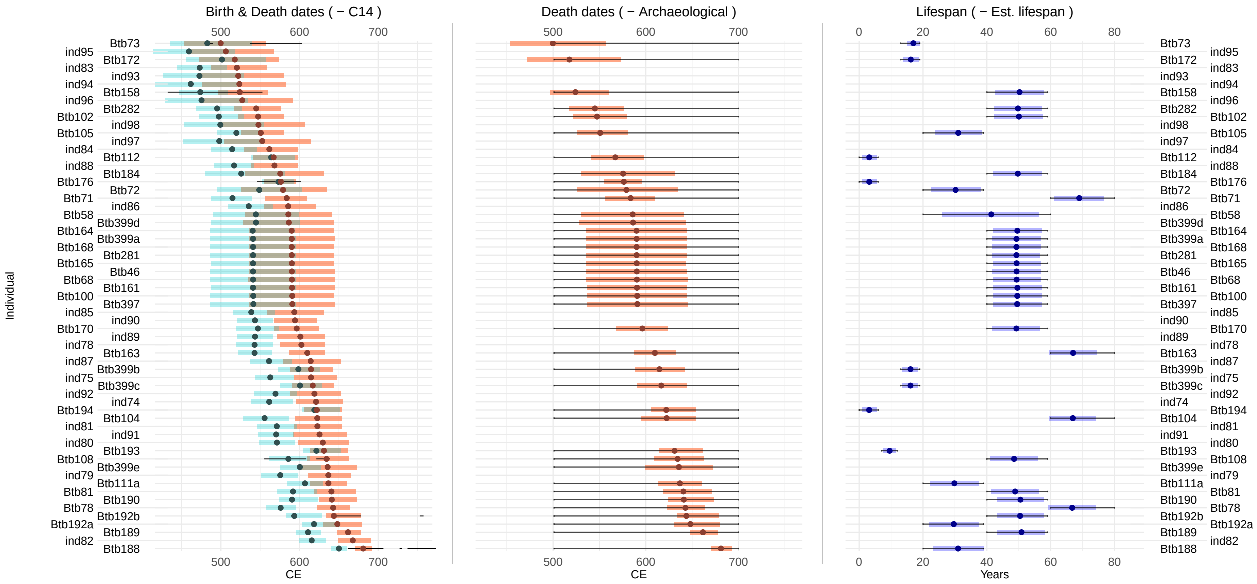

Fig. S3.5: *Life history estimates for individuals identified at Büttelborn. See Fig. S3.6 for details.*

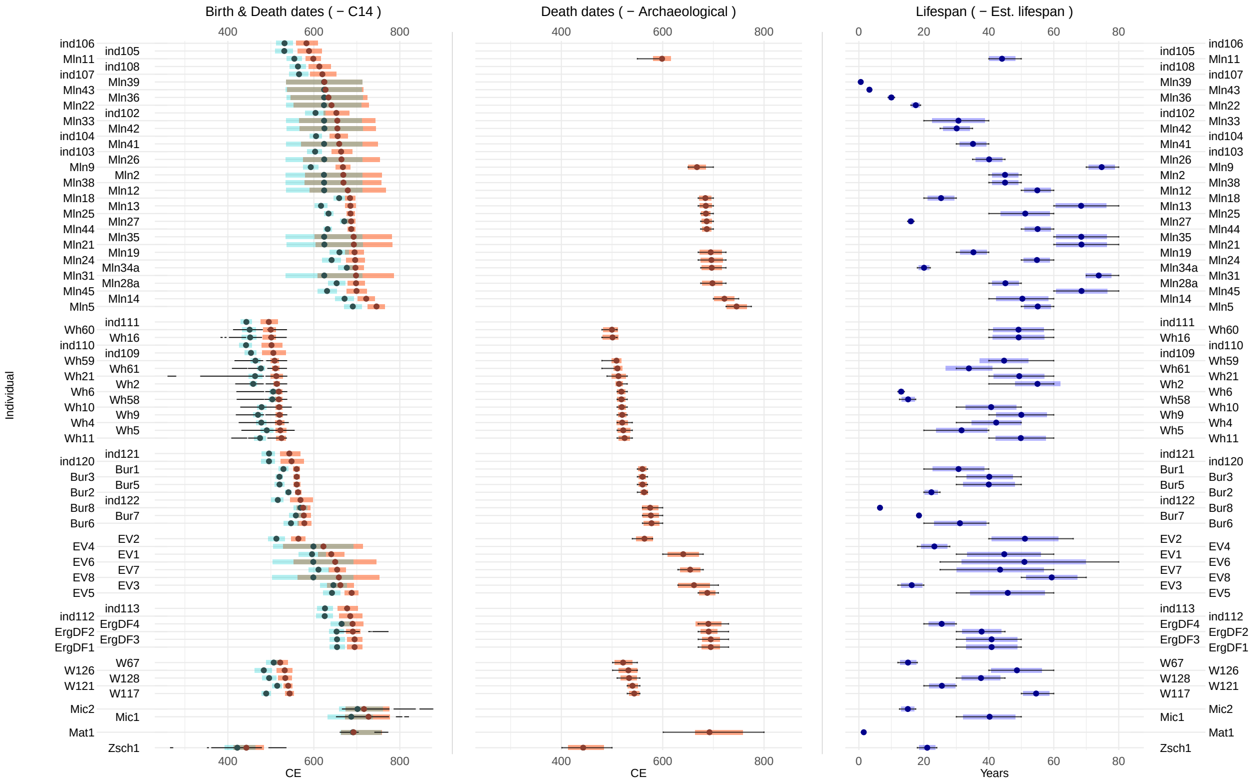

Fig. S3.6: *Life history estimates for individuals identified at all other sites. First column: posterior estimates (dot: mean, bar: 90% CI) of the birth (turquoise) and death (red) dates; 90% CI of the recalibrated ^{14}C date (black line, potentially disjunct), if available. Second column: posterior estimates of death dates; 90% CI of the archaeological date of the grave (black line), if available. Third column: posterior estimates of lifespan; 90% CI on the osteological age-at-death estimate (black line), if available. Individuals with red labels are non-locals based on isotopes. Individuals whose names start with "ind" have not been sampled but were implied by familial relationships.*

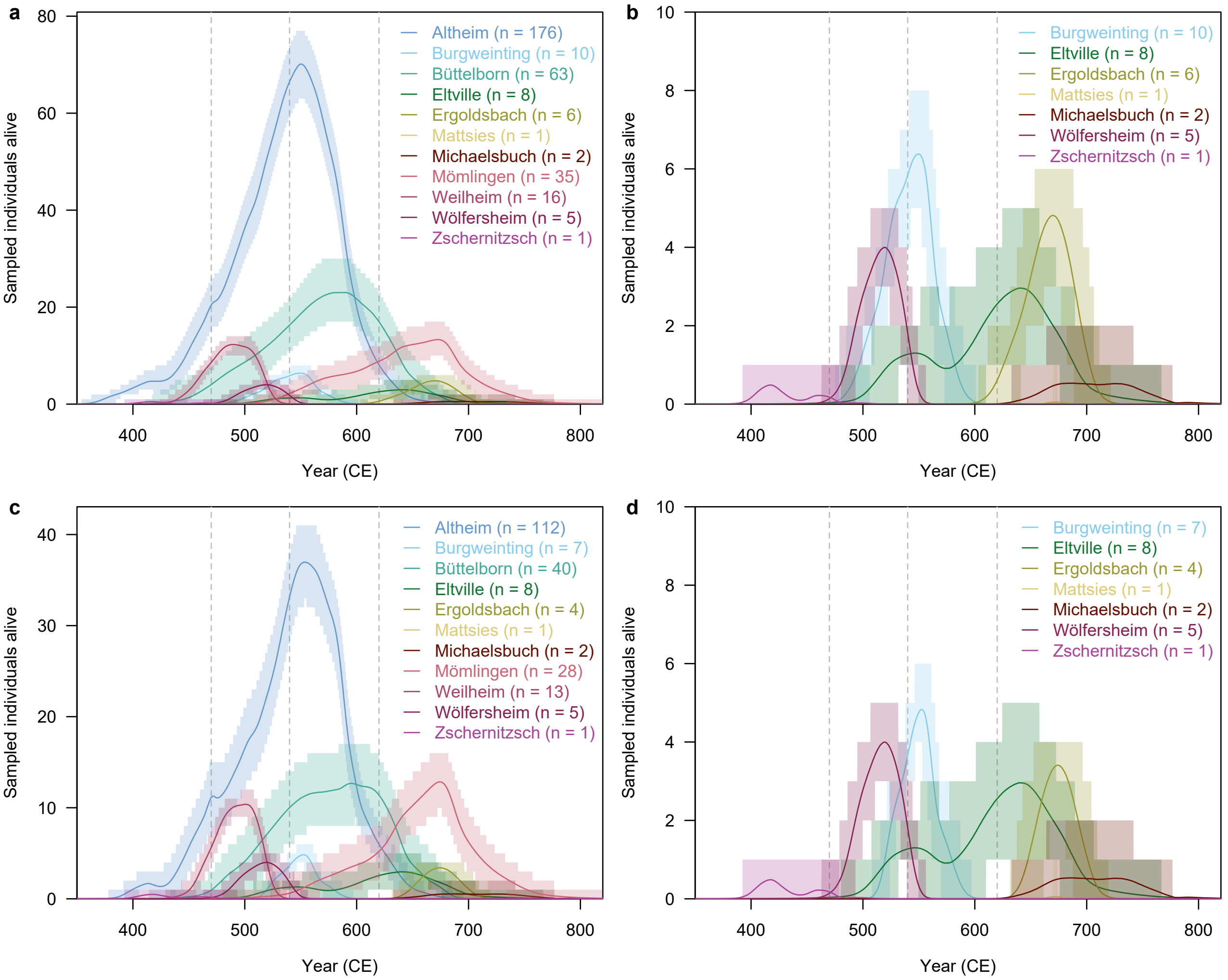

Fig. S3.7: Posterior estimates of the identified individuals alive. ***a,b****) Shown are the posterior mean (solid line) and the 90% CI (shades) of the number of identified individuals (either sampled or implied by family relationships) for all sites (****a****) or those with less than ten individuals at all times (****b****).* ***c,d****) As in a,b but for all individuals for which aDNA data was produced.*

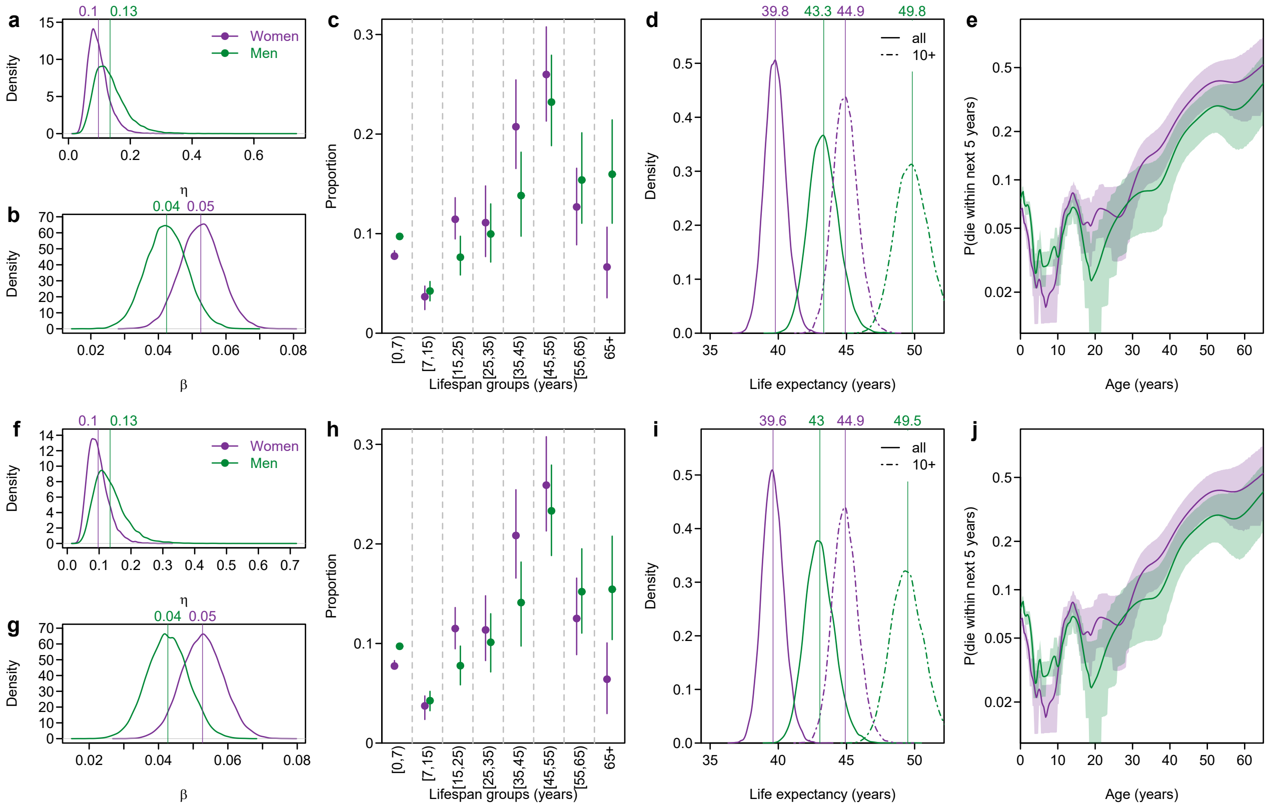

Fig. S3.8: *Posterior estimates on lifespan for women (purple) and men (green).* ***a,b****) Posterior estimates on the hyperparameters of the lifespan potential function (Gompertz distribution), $\eta_{♀}$, $\eta_{♂}$ and $\beta_{♀}$, $\beta_{♂}$, respectively.* ***c****) Posterior distribution (mean and 90% credible interval) of the fraction of samples in each of several lifespan groups.* ***d****) Posterior distribution on life expectancy (average lifespan) across either all samples (solid) or those with a $>$ 90% posterior probability to have lived at least until the age of ten (dash-dotted).* ***e****) Posterior mean (solid line) and 90% credible interval (shaded area) of the fraction of samples at a specific age (x-axis) that die within the next five years.* ***f–j****) Same as panels a–e but obtained with a more permissive lower bound of 12.75 on the fertility windows.*

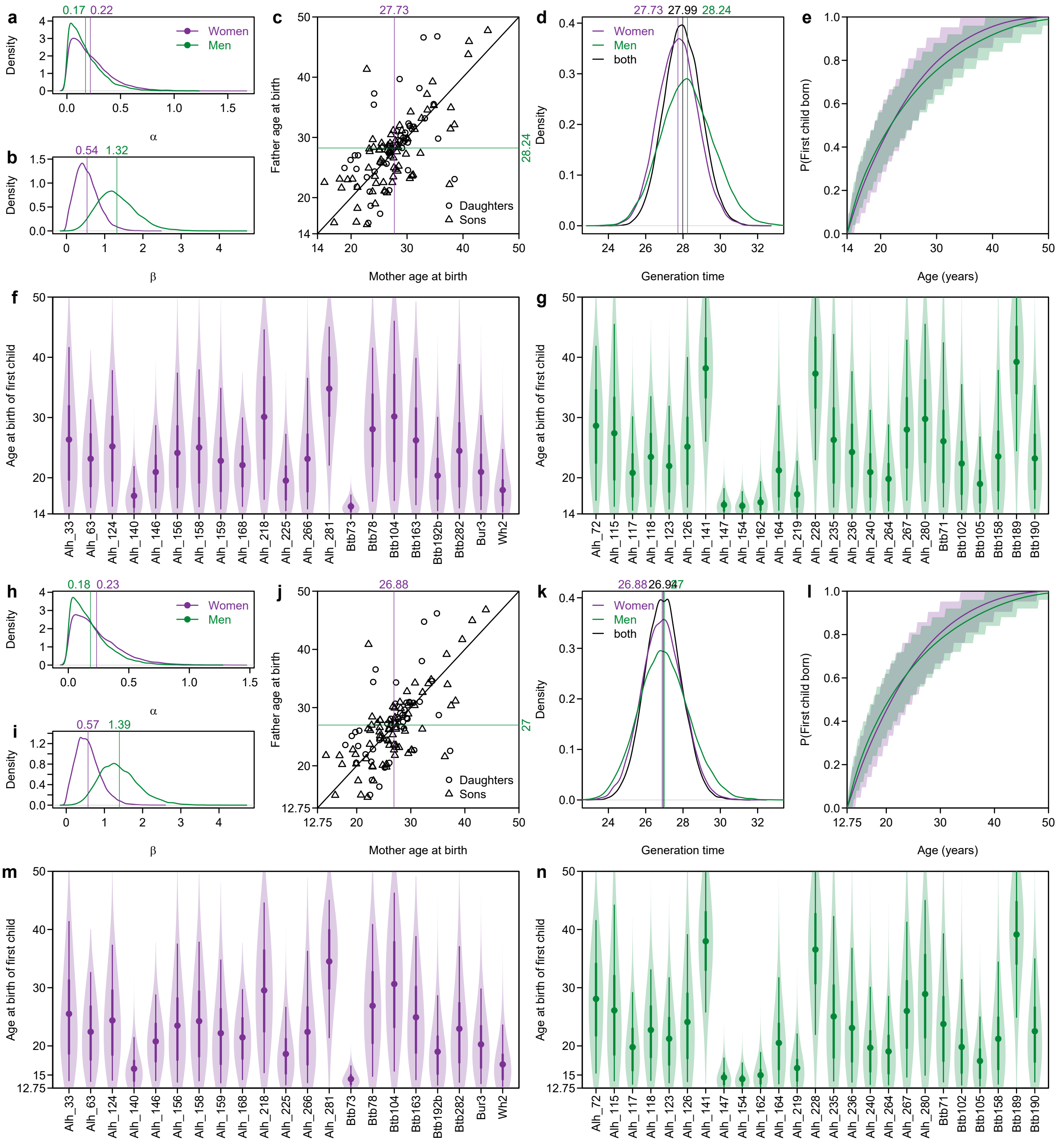

Fig. S3.9: Posterior estimates regarding generation time. ***a**,**b**) Posterior distributions and means (solid lines with values) of the hyperparameters of the offspring potential function (unimodal Beta distribution) for women ($\kappa_{1♀}$, $\kappa_{2♀}$, purple) and men ($\kappa_{1♂}$, $\kappa_{2♂}$, green).**c**) Posterior mean age at birth of the mother (x-axis) and father (y-axis) of each offspring. Solid lines with values indicate the posterior means of the mean age at birth (i.e. generation time). **d**) Posterior distributions and means (solid lines with values) on the generation time (average age at birth across all offspring) for women (purple), men (green) and both sexes (i.e. for autosomes, black). **e**) Posterior means (solid lines) and 90% CI (shades) of the fraction of all mothers (purple) and fathers (green) that had their first child prior to a specific age (x-axis). **f**,**g**) Posterior distributions (violin), means (dots) and 50% and 90% CI (thin and thick lines) for each mother (f) and father (g) on the age at their first child. **h**–**n**) As a–l but obtained with a more permissive lower bound of 12.75 years on the fertility windows.*

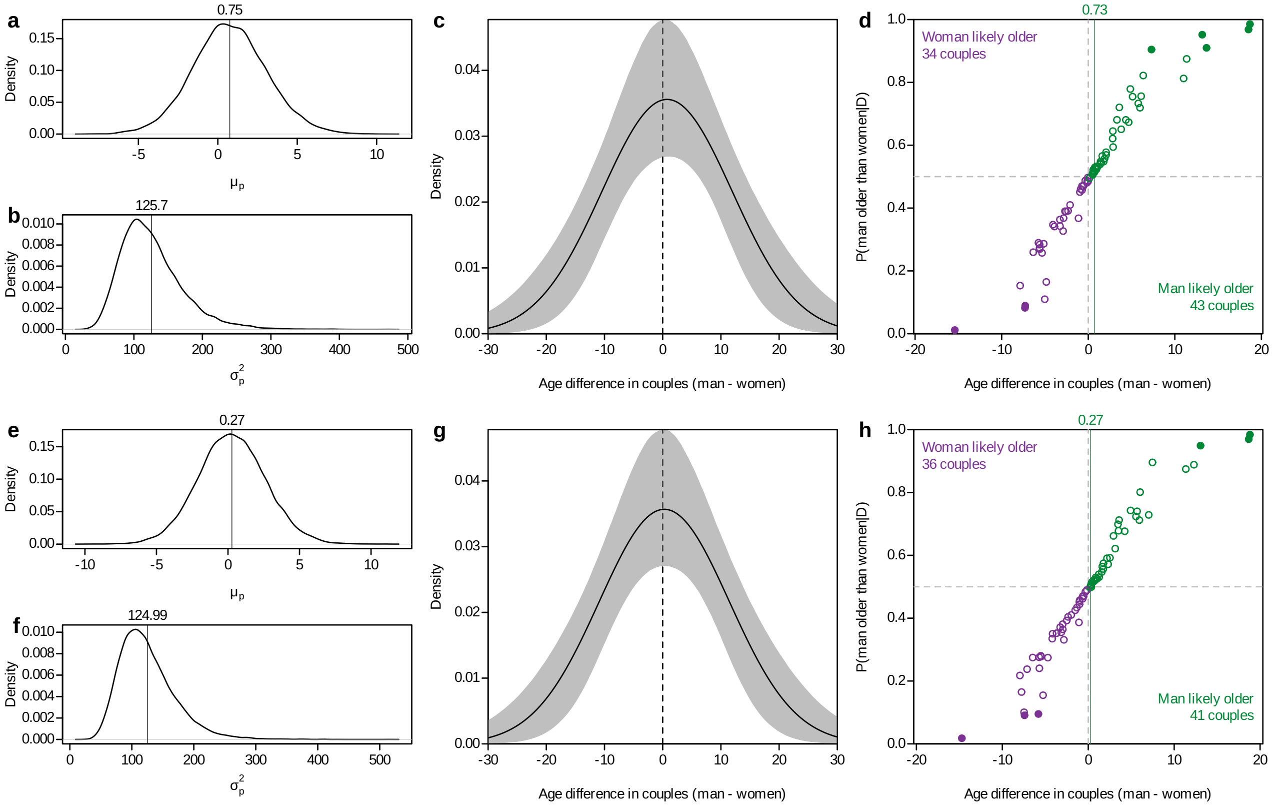

Fig. S3.10: *Posterior estimates regarding the age difference between partners.* ***a,b****) Posterior distributions and means (solid lines with values) on the hyperparameters of the parental potential function (normal distribution),* μ_p *and* σ_p^2*, respectively.* ***c****) Posterior distribution (mean and 90% credible interval) on the resulting normal distribution.* ***d****) Posterior mean estimate of the age difference (x-axis) and posterior probability* $\mathbb{P}(b_i < b_j|\boldsymbol{D})$ *that the father was older than the mother for each couple* $(i, j) \in \mathcal{P}$ *with at least one identified common child. Filled circles indicate couples for which the age difference is strongly different from zero, i.e. for which* $\mathbb{P}(b_i < b_j|\boldsymbol{D}) < 0.1$ *or* $\mathbb{P}(b_i < b_j|\boldsymbol{D}) > 0.9$*. The solid green line indicates the posterior mean on the age difference of 0.69 years.* ***e–h****) As a–d but obtained with a more permissive lower bound of 12.75 years on the fertility windows.*

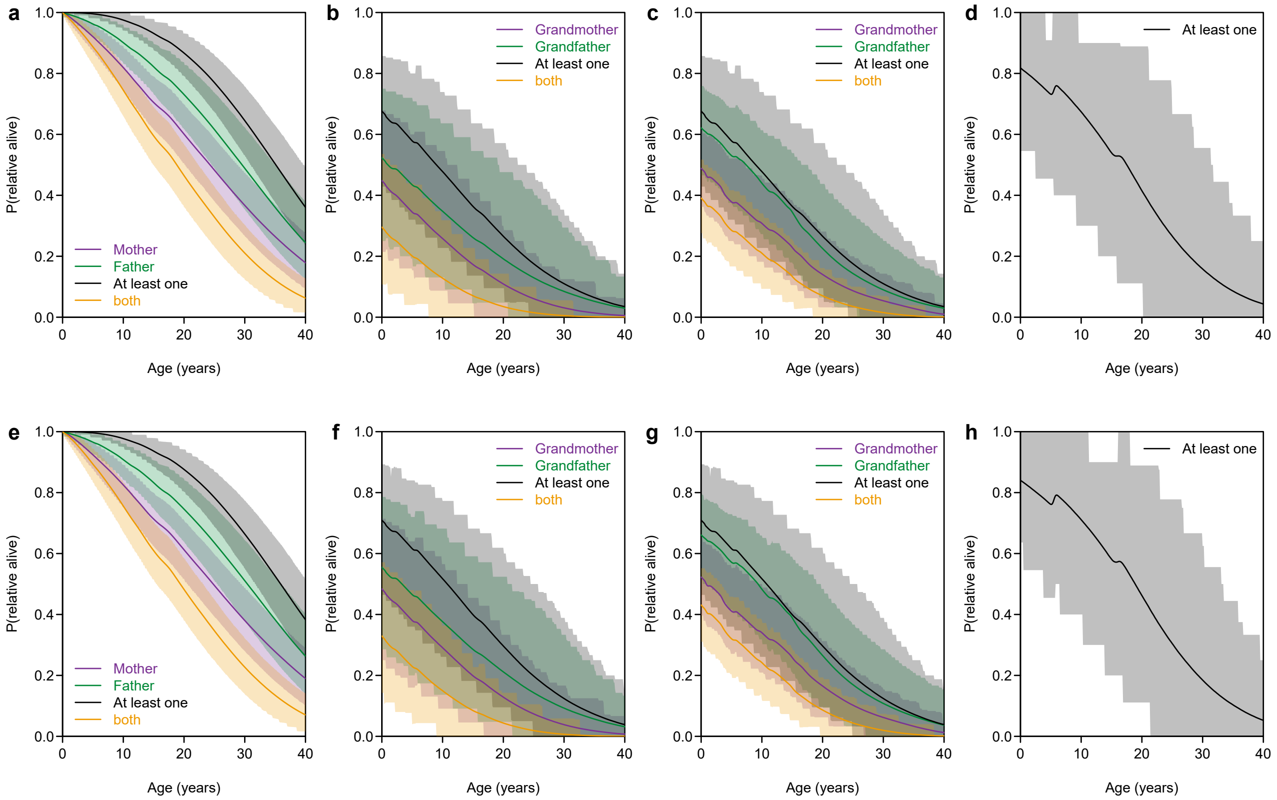

***Fig. S3.11:** Posterior estimates of relatives alive. **a**) Posterior means (solid lines) and 90% CI (shades) on the fraction of individuals of a given age (x-axis) whose mother (purple), father (green), at least one of them (gray) or both (orange) were alive. **b** and **c**) As in a but for maternal (b) and paternal (c) grandparents, respectively. **d** Posterior mean (solid line) and 90% (shades) on the fraction of individuals who had at least one of the four identified grandparents alive. In all cases the analysis was limited to individuals for which the relatives in question were identified. **e–h**) As a–d but obtained with a more permissive lower bound of 12.75 years on the fertility windows.*

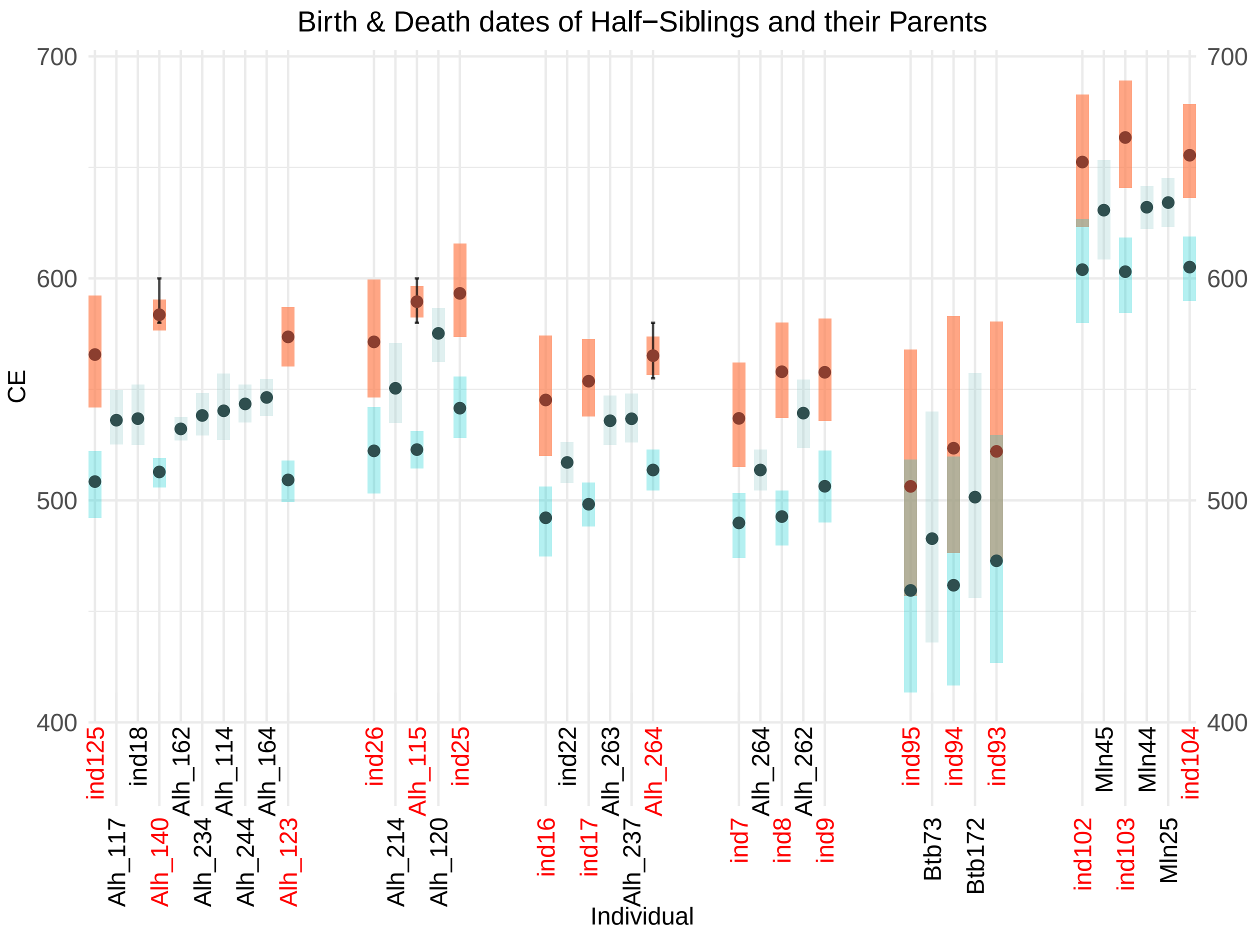

Fig. S3.12: *Life history estimates for identified half-siblings and their parents. For each cohort of half-siblings, the posterior mean (dots) and 90% CI (bar) are shown for birth and death dates for parents and birth dates for all offspring. Individuals are arranged as follows: the parent common to all half-siblings is shown in the center, their two partners at the left and right end and the common offspring in-between, ordered chronologically by their mean birth date. Labels of parents are shown in red. The 90% confidence interval of the archaeological date of the grave are also displayed if available (black line).*

S4. Whole Genome Data Production

Sample preparation

Sample preparation took place in the dedicated ancient DNA facilities of the Palaeogenetics Group Mainz and followed the protocol described in Žegarac *et al.* (2021) [198]. In deviation from this, some samples were not decontaminated by sandblasting, but instead sediment residues and the surface were removed with a saw blade during the isolation of the densest part of the bone. For the samples from Büttelborn the sampling of the dense part as well as the removal of sediment residues and the outer surface took place outside the clean room laboratory; samples were then transferred to the ancient DNA facilities where they were irradiated with UV light (254 nm) for 45 minutes from two sides before pulverization.

DNA extraction

DNA extraction either followed the protocol described in Žegarac *et al.* (2021) [198] or Zedda *et al.* (2023) [59] (indicated as "A" or "B", respectively, in column "Extraction protocol used" in Supplementary Table 1).
An additional step was added to the extraction protocol described in Zedda *et al.* (2023) [59] for some samples (indicated as "C" in column "Extraction protocol used" in Supplementary Table 1) following the protocol by Boessenkool *et al.* (2017) [199]: Prior to pre-lysis the bone powder was incubated with 990 µl Sodium Hypochlorite (0.5 %) for 15 minutes, then pelleted and the supernatant discarded. Then three wash steps with 1ml NF-H_2O (nuclease-free water) each were performed discarding the supernatant in between.

DNA library preparation and whole genome sequencing

DNA library preparation followed the protocol described in Zedda *et al.* (2023) [59]. DNA libraries were sequenced on the NovaSeq 6000 (Illumina; 100bp, SE) at the Next Generation Sequencing Platform at the University of Bern.
For sample Molz1 DNA libraries were prepared following the protocol described in Marchi *et al.* (2022) [200] and subsequently sequenced on the HiSeq3000 (Illumina; 100bp, SE) at the Next Generation Sequencing Platform at the University of Bern.

S5. Bioinformatic Processing

Alignment

Trimmomatic 0.36 [201] was used in combination with the TruSeq3 adapter set in fasta format, provided by the package to remove residual adapter sequences from the raw-reads, while retaining only reads >= 30bp. Trimmed reads for each library were aligned against the 1000 genomes project version of hg19 [73] using bwa mem [202] with default options and the output was directly converted to the binary aligned format (BAM), using samtools 1.13 [203]. PCR duplicates were removed using *sambamba* [61]. After adding individual readgroups using samtools addreplacerg [203] libraries were merged on the individual level using *sambamba* [61]. Softclipped bases were removed from the alignments, while simultaneously adjusting CIGAR strings and readgroups using a custom python script relying on pysam 0.22 (https://github.com/pysam-developers/pysam). Finally all reads were re-aligned against known SNPs and InDels with GATK 3.8 [62]. All data from this study can be found as raw reads as well as BAMs at ENA under the accession number PRJEB87112.

Modern genomes

Modern genomes from the DZHK cohorts were provided upon request from the DZHK heart bank (https://dzhk.de/dzhk-heart-bank/daten-und-bioproben/dzhkomics-ressource) via the university of Luebeck as raw alignments in BAM format. Genomes were selected from 1,300 subjects from six population-based, epidemiological cohort studies in Germany (Kiel, Hamburg, Greifswald, Mainz, Heidelberg, Munich) with the intent to build a control reference for future sequencing efforts.

Gutenberg-Gesundheitsstudie (GHS) - The Gutenberg Health Study, University Medical Center of the Johannes Gutenberg University Mainz

Hamburg City Health Study (HCHS) - The Hamburg City Health Study, University Medical Center Hamburg-Eppendorf

Heidelberg Normal Kontrollen (NOKO) - Heidelberg Normal Controls, Heidelberg University Hospital

Kooperative Gesundheitsforschung in der Region Augsburg (KORA) - Cooperative Health Research in the Augsburg Region, Helmholtz Zentrum München

Study of Health in Pomerania (SHIP) - The Study of Health in Pomerania, University Medical Center Greifswald

Institut für Epidemiologie in Kiel (IFE) - The Institute for Epidemiology in Kiel, University Medical Center Schleswig-Holstein

Individuals from local populations were selected with an average age of 57 years and an almost balanced sex ratio (48.3% females to 51.7 males). Cardiovascular risk factors were recorded for each individual, which were in the normal range for most of the participants. As a reference, representative of the northern and southern-most parts of modern day Germany, we selected the cohorts Kiel and Munich (N=379).

Paired-end reads were merged with the function “mergeOverlappingReads” from the ATLAS [60] tool-kit, while also removing soft-clipped bases in the same step. PCR-duplicates were removed with *sambamba* markdup [61] and alignments were filtered retaining only reads with a mapping quality of at least 30 using samtools [203]. Afterwards all reads were re-aligned against known SNPs and InDels using *GATK 3.8* [62].

Variant detection

All variant detection was done using *ATLAS* ([60], https://bitbucket.org/wegmannlab/atlas), which implements methods originally described in [204]. All initial processing of the data was done with commit "647daf792df01c4aa8e686de0e462ddd8f91e6c6". Single end reads were split according to their maximum sequencing length (100bp) and post-mortem damage patterns were estimated per readgroup using the PMD task. Afterwards, sequencing errors were estimated jointly for all read-groups that were sequenced on the same flow-cell, focussing on regions determined to be ultra-conserved between 88 mammal species. Pseudo-haploid allele calls for regions overlapping the 1240K capture array SNPs [65] were produced with *ATLAS*'s majorityAllele task, which picks the most common allele at each specified site while choosing one at random if no majority can be determined. To create a data-set fit for imputation we called diploid genotypes and corresponding genotype likelihoods using *ATLAS*'s MLE caller. As the majority of previously published genomes was sequenced on the 1240K SNP array, data for sequencing error-estimation was limited or rendered it impossible. Therefore we restricted the *ATLAS* pipeline for genomes from the literature to the PMD estimation. If read-group information was not present we inferred read-groups based on read names, allowing at least for the separation of reads from different flow cells. Individual VCFs were merged with bcftools 1.13 [205] on chromosome level for all downstream analyses. All modern genomes from the DZHK data-set were processed with the newer *ATLAS* commit "7d732d9a87f7ce390cbe1d5740e58982be0821ff". PMD estimation was omitted as no post-mortem damage is expected for modern genomes, the recall step was omitted as the effect of sequencing error on variant calls from genomes with an average coverage of 30X should only be minor and can therefore be neglected. Diploid genotypes were called with *ATLAS's* MLE caller, resulting VCFs were filtered for a minimal depth of 8 and a genotype-quality of >= 30. The subsequent processing was identical to all other genomes included in this study.

Imputation of ancient genomes

All imputation was done with *GLIMPSE2* [63,64] using haplotypes from the 1000 Genomes data as reference. To prevent batch-effects we processed genomes produced by shotgun sequencing separately from genomes sequenced on the 1240k capture array. All shotgun genomes were imputed for all bi-allelic sites in the 1000 Genomes data set [73] with a minor allele frequency > 0.01, while 1240k capture genomes were imputed only for the autosomal chromosomes in the 1240k sites.

S6. Uniparental markers, genetic sex determination and contamination estimation

Genetic sex for all individuals was determined using *BeXY* [139]. Mitochondrial haplotypes were determined using *haplogrep3* [66] from merged VCF files. We used *ATLAS's* majorityBase call function to produce individual calls for the mitochondrial chromosome under consideration of individual PMD distributions, while simultaneously ignoring the first and last 2 bases on each read in the alignment. The resulting calls were filtered for a minimal read depth of 10 and merged into a single file. Y-chromosomal haplotypes were inferred with *Yleaf2* [67] from individual BAM files for all male individuals, relying on ISOGG (July 2019). Contamination for all genomes was estimated with contamMix [206] and with ANGSD [207] for all genomes of male individuals.

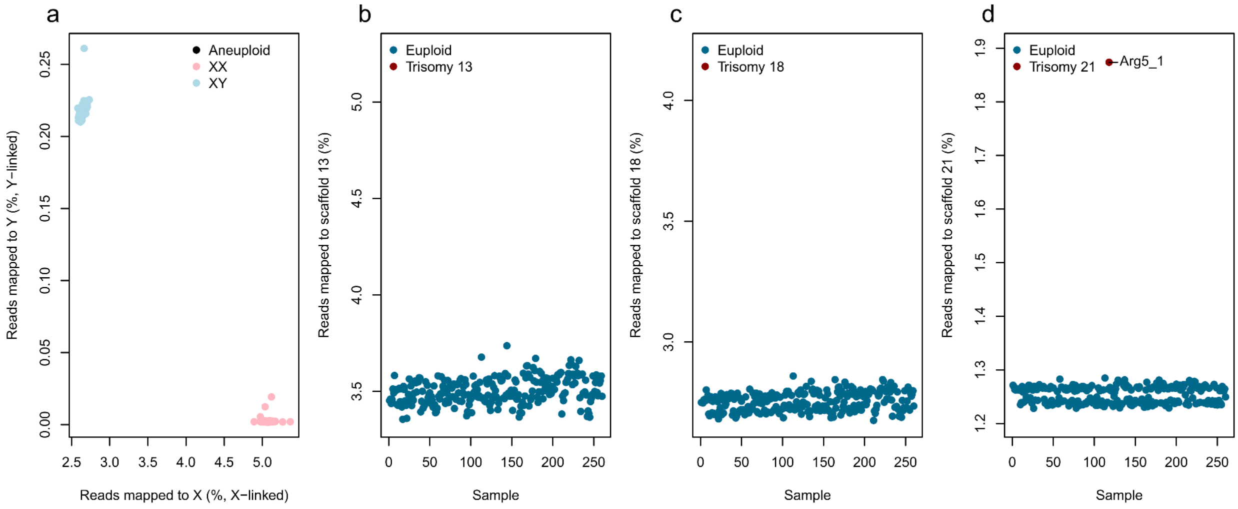

Fig. S6.1: *BeXY results showing the inferred genetic sex and the fraction of reads mapping to the X- and Y-chromosomes (a), as well as the percentage of reads mapping to chromosomes 13, 18, and 21 (b–d), with trisomies highlighted, for all individuals.*

Haplogroup diversity

We used a custom python script to estimate haplogroup diversities based on uniparental markers following the description in Žegarac *et al.* (2021) [198]. To address potential uncertainties in especially the Y-chromosomal haplotype assignment, we estimated diversities only on the haplogroup level, using the first sub-clade (R1a vs. R2b etc.). Confidence intervals were estimated based on a jackknife resampling approach.

Diversities were high for both mitochondrial as well as Y-chromosomal markers, regardless of sampling site, with MT-diversities always exceeding 0.95 (Table S6.1).

Table S6.1: *Uniparental marker diversity for sites with >20 individuals. Diversity estimates are reported for mitochondrial and Y-chromosomal haplogroups for all as well as unrelated individuals only (Unrel_*).*

Site	subgroup	H_mt	CI_l	CI_u	H_Y	CI_l	CI_u
Altheim	All	0.9588	0.9400	0.9776			
	XX	0.9706	0.9485	0.9926			
	XY	0.9502	0.9142	0.9861	0.7414	0.6675	0.8153
	Unrel_all	0.9747	0.9436	1.0059			
	Unrel_XX	0.9830	0.9537	1.0123			
	Unrel_XY	0.9394	0.7962	1.0826	0.8939	0.7327	1.0552
Büttelborn	All	0.9667	0.9331	1.0002			
	XX	0.9605	0.9014	1.0196			
	XY	0.9779	0.9041	1.0518	0.8235	0.6983	0.9487
	Unrel_all	1.0000	0.9426	1.0574			
	Unrel_XX	1.0000	0.8630	1.1370			
	Unrel_XY	1.0000	0.6917	1.3083	0.9333	0.5776	1.2891
Mömlingen	All	0.9735	0.9335	1.0136			
	XX	0.9778	0.8362	1.1193			
	XY	0.9804	0.9167	1.0441	0.6601	0.4279	0.8923
	Unrel_all	0.9631	0.9200	1.0061			
	Unrel_XX	0.9556	0.7975	1.1136			
	Unrel_XY	0.9591	0.8904	1.0278	0.5263	0.2270	0.8257

Table S6.2: *Uniparental marker diversity for sites >20 individuals. Diversity estimates are reported for mitochondrial and Y-chromosomal haplogroups..*

Site	subgroup	H_mt	CI_l	CI_u	H_Y	CI_l	CI_u
Kiel	all	0.8906	0.8562	0.9249			
	XX	0.9098	0.8714	0.9482			
	XY	0.8645	0.8102	0.9189	0.6210	0.5141	0.7278
Munich	all	0.9367	0.9182	0.9552			
	XX	0.9188	0.8804	0.9571			
	XY	0.9488	0.9273	0.9703	0.6489	0.5393	0.7586

Y haplogroup frequencies

Since the geographic structure of Y haplogroups can provide valuable support for the autosomal ancestry patterns, we compared the Y haplogroup (first three characters) frequencies of Early Medieval individuals from Germany with a positive filia-corrected f_4(Roman Southeastern Europe + Iron Age Central Italy, Northern Europe, X, Mbuti) with the frequencies of Iron Age individuals from Italy and Southeastern Europe. The latter were extracted from the Allen Ancient DNA Resource (AADR v62) metadata [68].

The results are consistent with the autosomal inference of a higher contribution from Southeastern Europe to our medieval sample. Notably, three quarters of the inspected males carry haplogroup E1b, which occurs at approximately 28% frequency in Southeastern Europe but only around 2% in Italy during the Iron Age (Fig. S6.2).

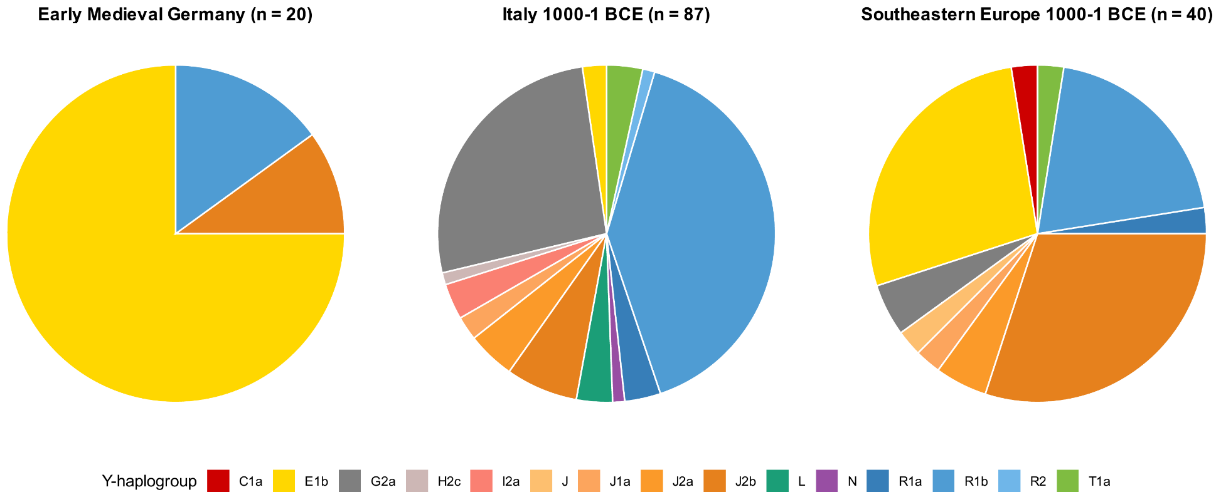

Fig. S6.2: *Y haplogroup (first three characters) frequencies in Early Medieval individuals from Germany with a positive f_4(Roman Southeastern Europe + Iron Age Central Italy, Northern Europe, X, Mbuti) compared with frequencies of Italy and Southeastern Europe in the period 1000-1 BCE.*

S7. Principal Component Analysis

We used *smartpca* [70] to project ancient genomes onto a set of reference genomes from western Eurasia, taken from the human origins data set [208] as published in the v54 from the Allen Ancient DNA Resource [68,69]. Individuals associated with the following population labels were used:

Abkhasian.HO, Adygei.HO, Albanian.HO, Armenian.DG, Armenian_Hemsheni.HO, Armenian.HO, Armenian.WGA.HO, Assyrian.HO, Assyrian.WGA.HO, Azeri.HO, Azeri.WGA.HO, Balkar.HO, Basque.DG, Basque.HO, Belarusian.HO, Bulgarian.HO, Chechen.HO, Cretan.DG, Croatian.HO, Cypriot.HO, Czech.HO, Dongxiang.HO, Druze.HO, English.HO, Estonian.HO, Ezid.HO, Finnish.HO, Finnish_o.DG, FIN_o.HO, French.DG, French.HO, Gagauz.HO, Georgian.HO, Georgian.WGA.HO, Greek.HO, Greek.WGA.HO, Hungarian.HO, IBS_CanaryIslands.HO, Icelandic.HO, Ignore_Palestinian.DG, Ignore_Palestinian.HO, Iranian_Bandari.HO, Iranian.HO, Italian_North.HO, Italian_Sardinian.HO, Italian_South.HO, Jew_Ashkenazi.HO, Jew_Georgian.HO, Jew_Iranian.HO, Jew_Iraqi.HO, Jew_Turkish.HO, Jew_Yemenite.HO, Jordanian.HO, Kumyk.HO, Kurd.HO, Lezgin.DG, Lezgin.HO, Lithuanian.HO, Maltese.HO, Mordovian.HO, Norwegian.HO, Orcadian.DG, Orcadian.HO, Ossetian.HO, Palestinian.DG, Palestinian.HO, Palestinian_oAfrican.HO, Polish.DG, Romanian.HO, Russia_Abkhasian.HO, Russian_Archangelsk_Krasnoborsky.HO, Russian_Archangelsk_Pinezhsky.HO, Russian.DG, Russian.HO, Russia_NorthOssetian.HO, Saami.WGA.HO, Samaritan.DG, Sardinian.HO, Scottish.HO, Sicilian.HO, Spanish.HO, Spanish_Lleida.HO, Spanish_North.HO, Turkish_Balikesir.HO, Turkish.DG, Turkish.HO, Ukrainian.HO, Ukrainian_North.HO

The Early Medieval individuals from Germany published in this study occupy a PCA space much larger compared to modern day populations from a comparable geographic origin. Individuals are overlapping the PCA space where modern day individuals from Northern to Central, as well as South-Western and South-Eastern Europe can be found. Several individuals overlap with the empty space between the Eastern and Western Mediterranean populations.
Compared to pre-Roman/Iron Age individuals the majority of Early Medieval individuals, with the exception of some outliers, occupy a space similar to Central/Northern Europe to South/Southeastern European genomes.

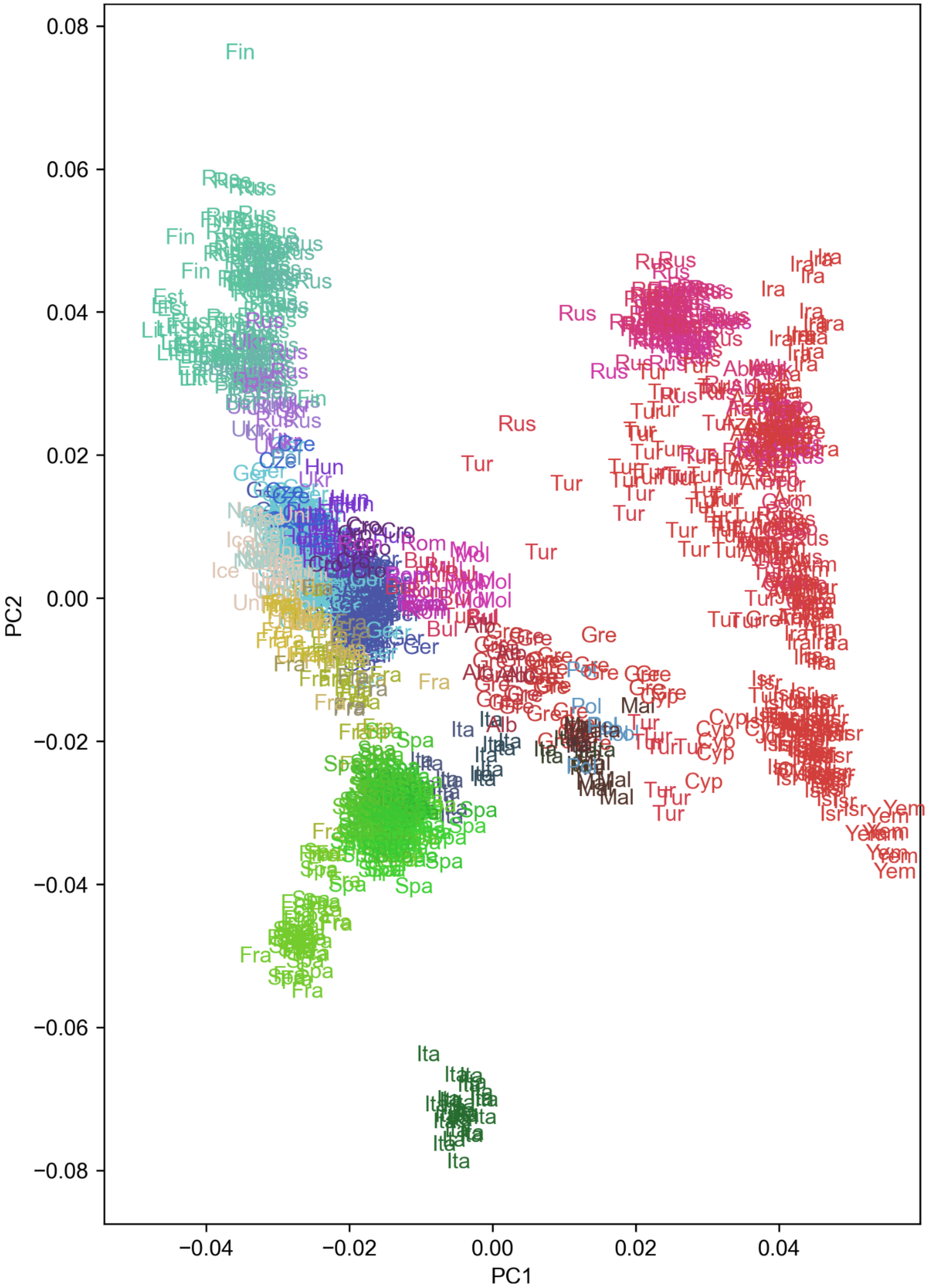

Fig. S7.1: *The first two principal components for modern reference genomes from Western Eurasia are shown. Countries are abbreviated using their first three letters, as listed in the AADR v54 file. Colors in the figure were assigned according to a colormap derived from the GPS coordinates of each sampling location. The geographic origins of the corresponding Iron Age reference genomes are depicted in Fig. 7.4.*

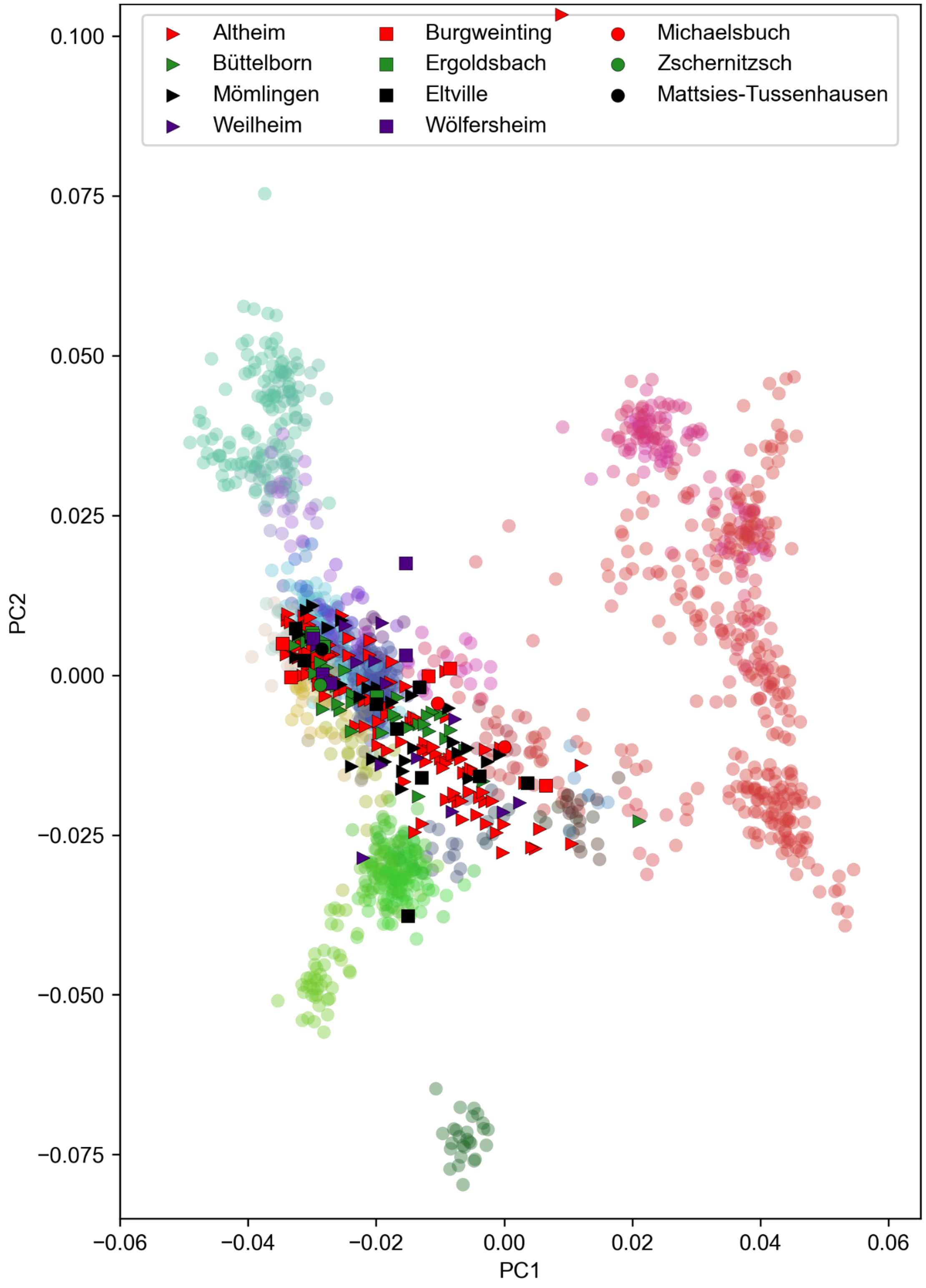

Fig. S7.2: *First two principal components for newly sequenced Early Medieval genomes, projected onto modern reference genomes from western Eurasia, depicted as colored points in the background. Colors for the reference genomes were assigned based on the lat/lon coordinates listed in the AARD file and are the same as in Fig. S7.1. Archaeological sites with more than 10 genomes are depicted as triangles, sites with between four and nine individuals are depicted as squares, sites with below four individuals are depicted as circles.*

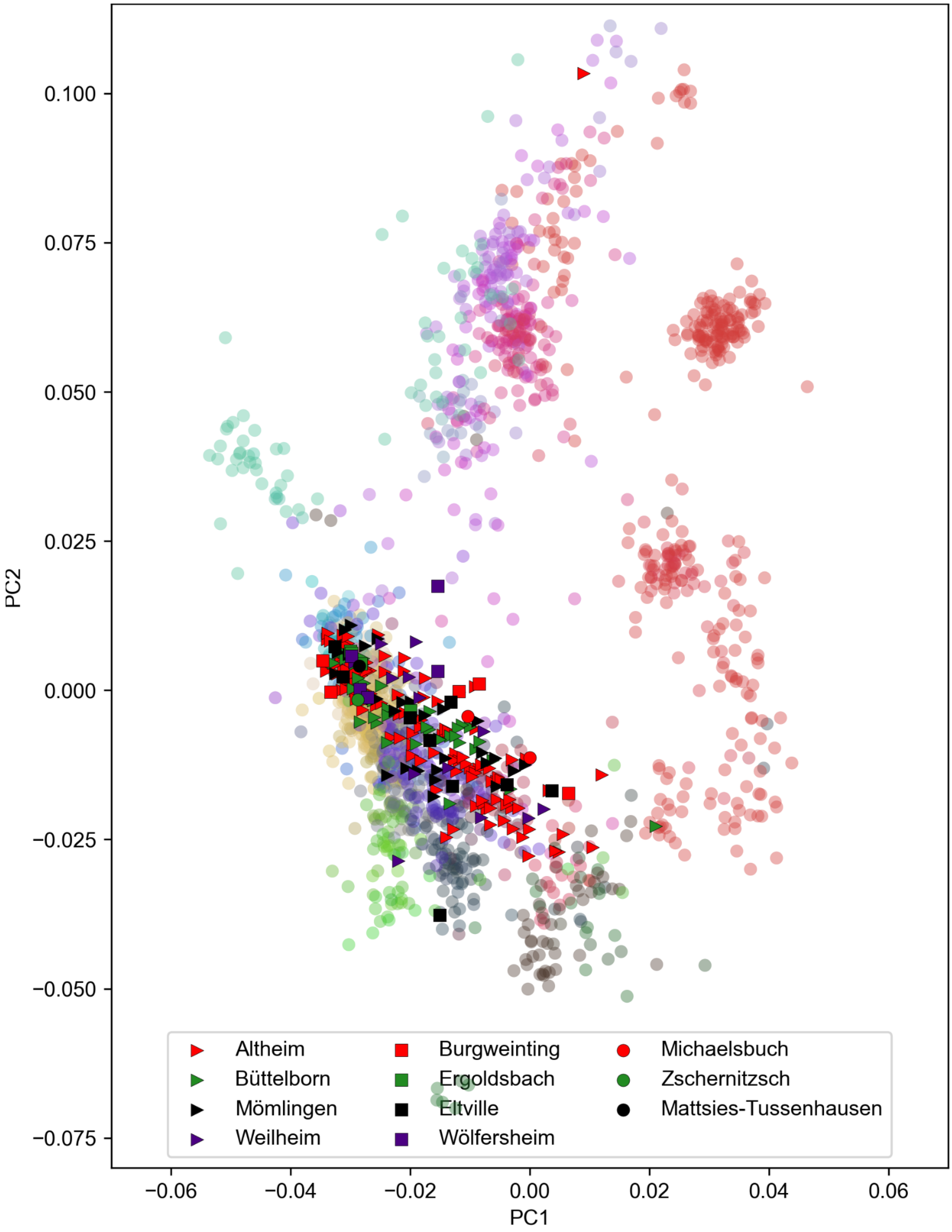

Fig. S7.3: *First two principal components for newly sequenced Early Medieval genomes alongside previously published genomes dating between 1000 BC and 0 (e.g. Iron Age to Antiquity) from western Eurasia, depicted as individual colored points in the background.. Colors for the reference genomes were assigned based on the lat/lon coordinates listed in the AARD file and are the same as in Fig. 2e. Archaeological sites with more than 10 genomes are depicted as triangles, sites with between four and nine individuals are depicted as squares, sites with below four individuals are depicted as circles.*

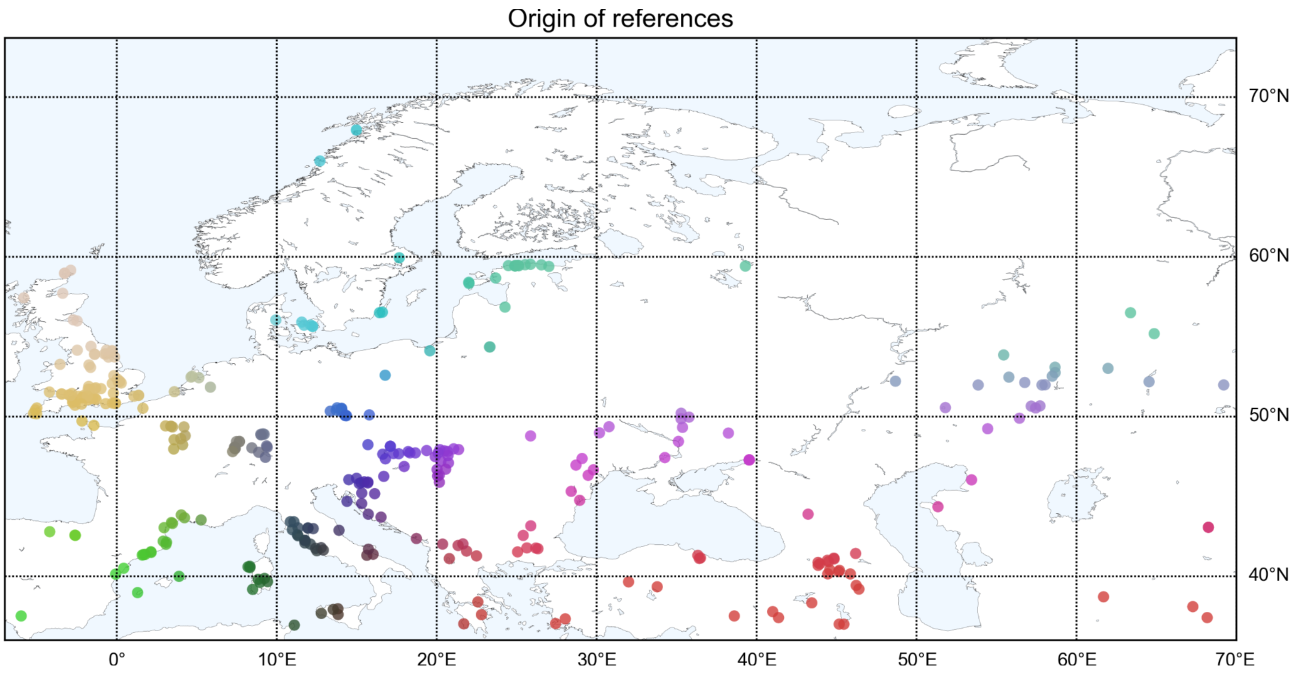

Fig. S7.4: *Map showing an extended set of the sampling locations and corresponding colors for the pre-Roman/Iron Age reference genomes used as the background in main Fig. 2. Colours were assigned based on the individual GPS coordinates.*

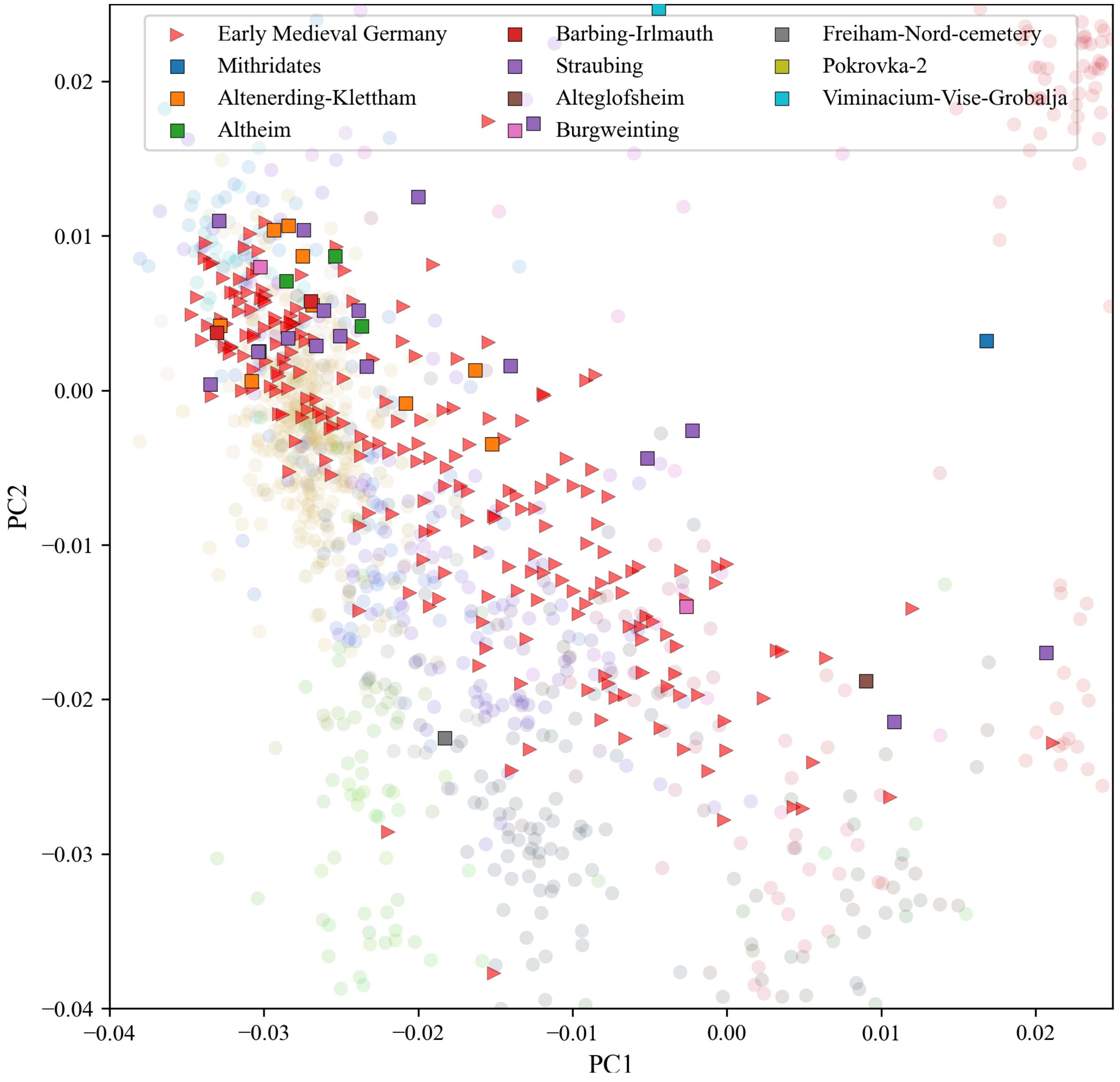

Fig. S7.5: *PCA plot showing newly sequenced Early Medieval individuals from Germany (red triangles) alongside previously published individuals (squares) from Veeramah et al. (2018)* [14]*. The previously published individuals predominantly cluster in the upper third of the PCA space.*

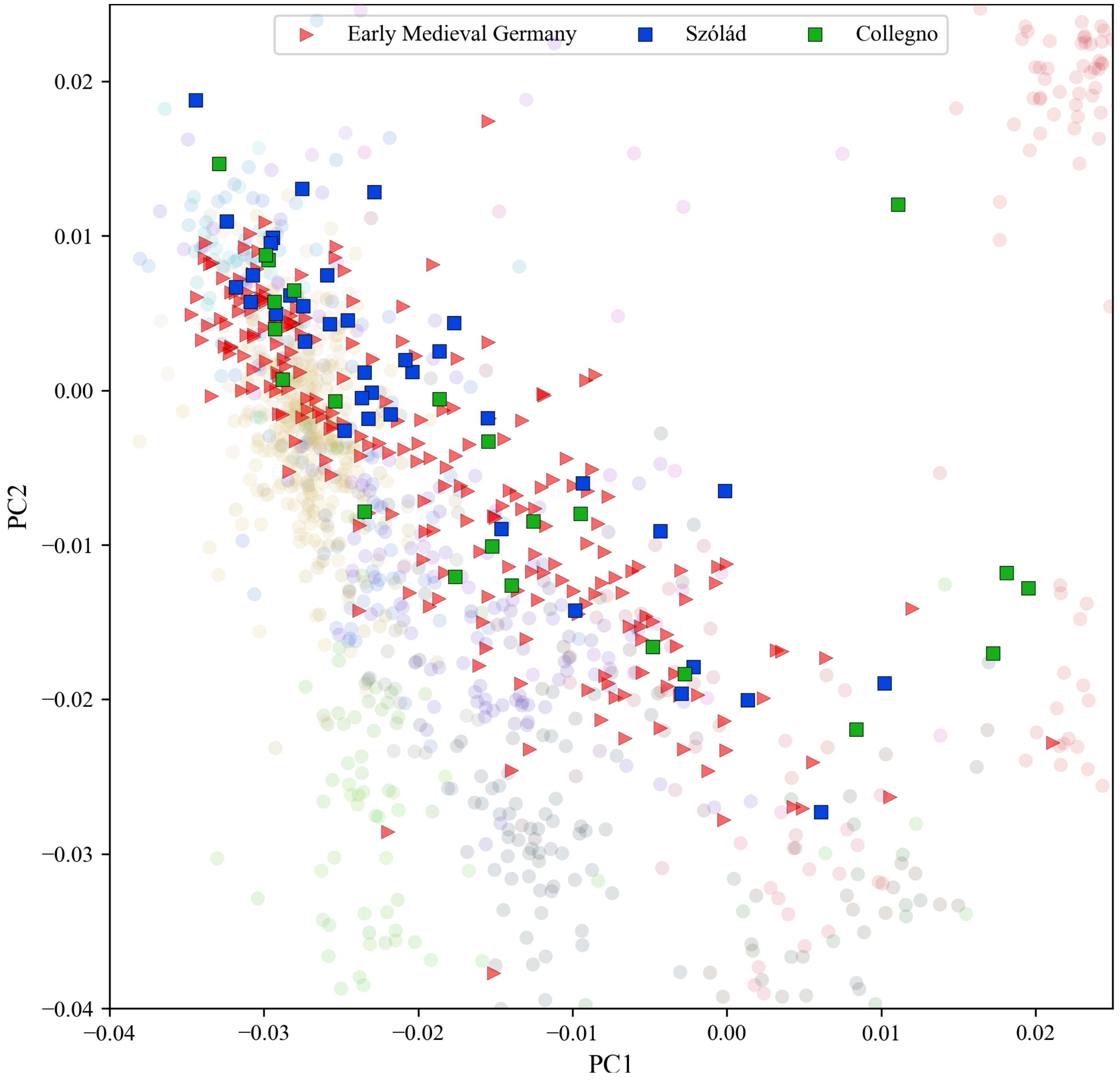

Fig. S7.6: *PCA plot showing newly sequenced Early Medieval individuals from Germany (red triangles) alongside previously published individuals from Amorim et al. (2018) [15] from Szólád, Hungary (blue squares), and Collegno, Italy (green squares). Both groups exhibit a similar distribution across the PCA space.*

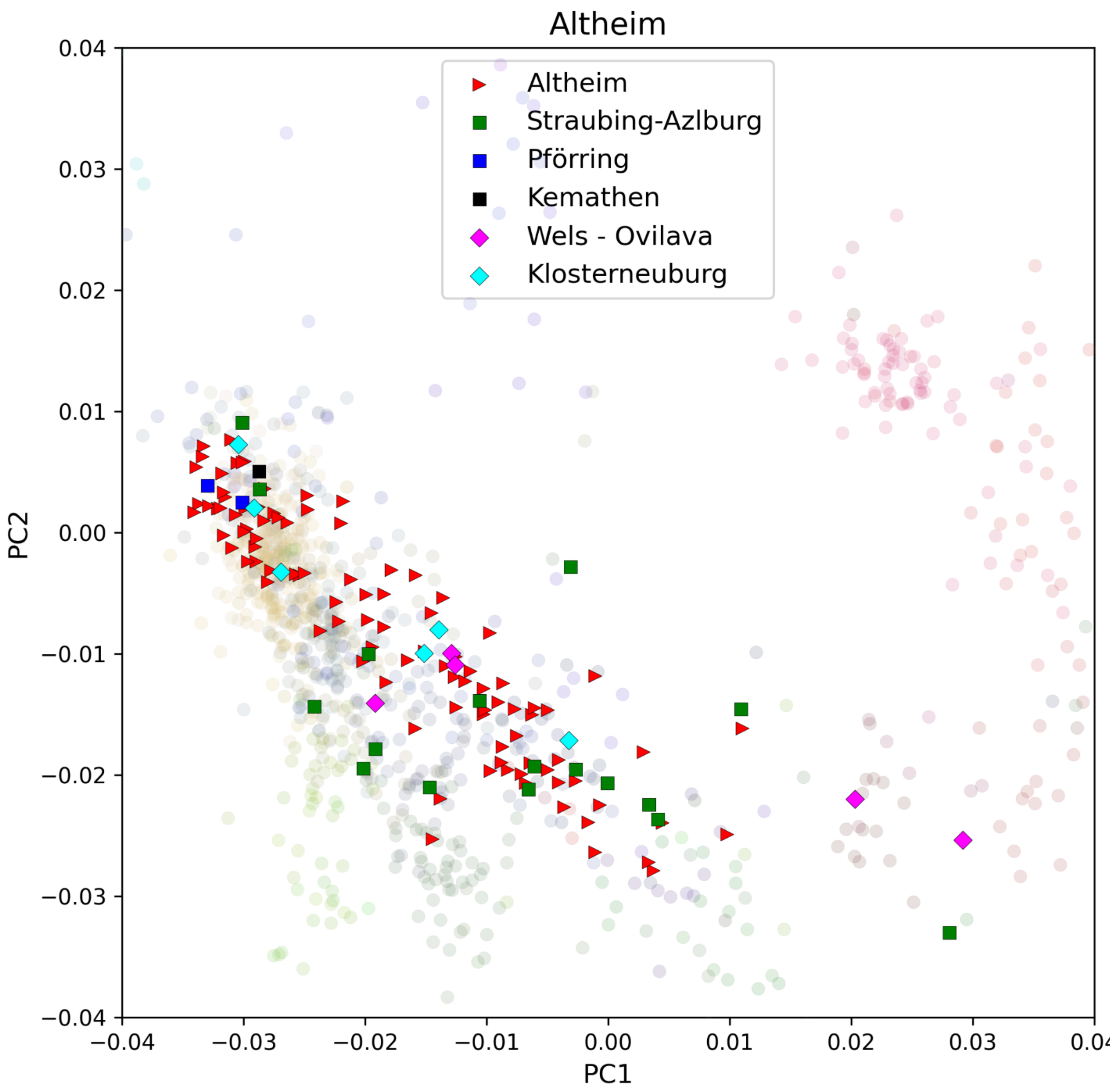

Fig. S7.7: *PCA plot showing individuals from the Altheim site (red) alongside Late Antique individuals from German sites Straubing-Azlburg, Pförring, and Kemathen, as well as Austrian sites Wels (Ovilava) and Klosterneuburg* [18].

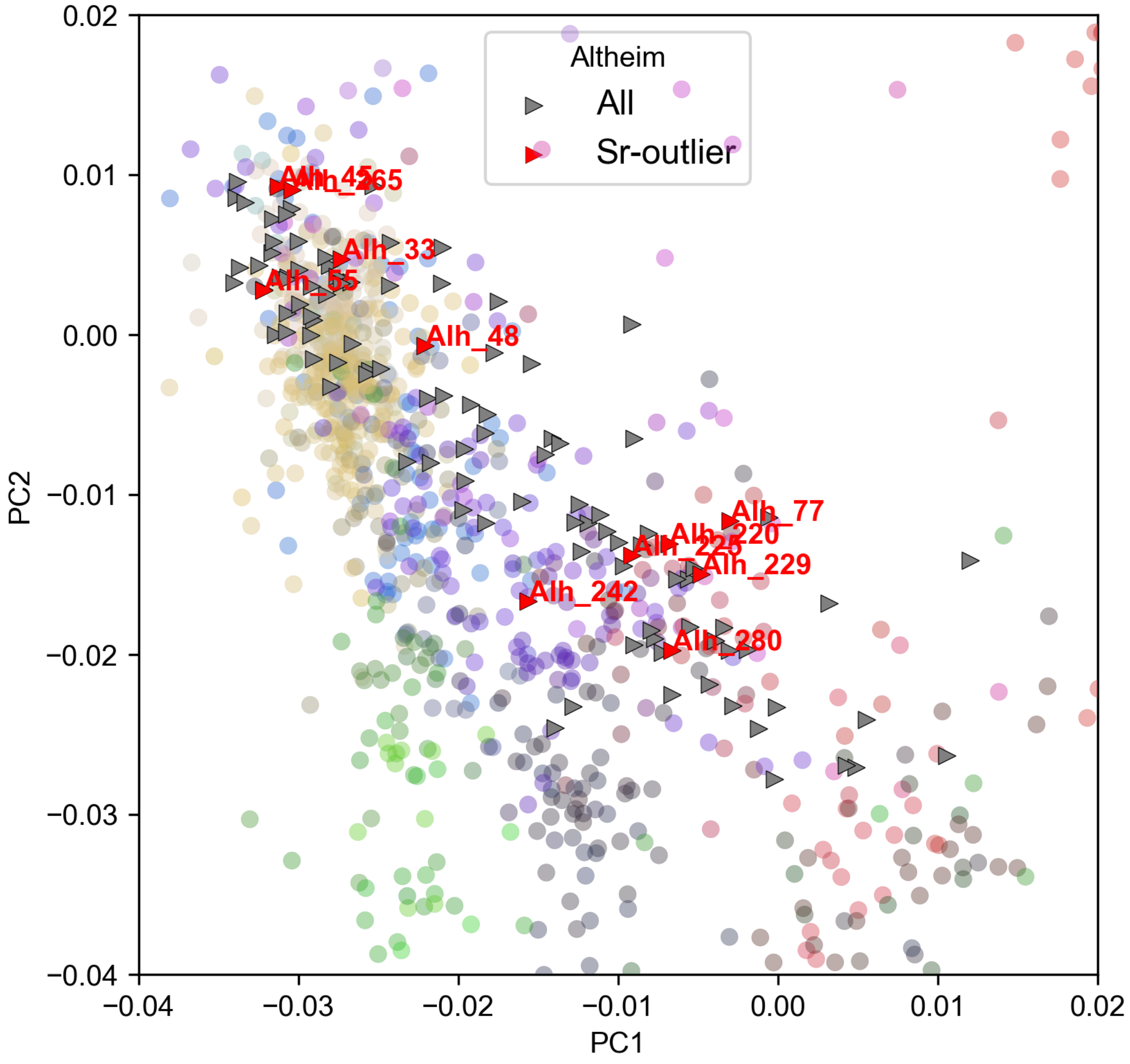

Fig. S7.8: *A plot of Altheim individuals (grey triangles), with those exhibiting a non-local strontium isotope signature highlighted in red.*

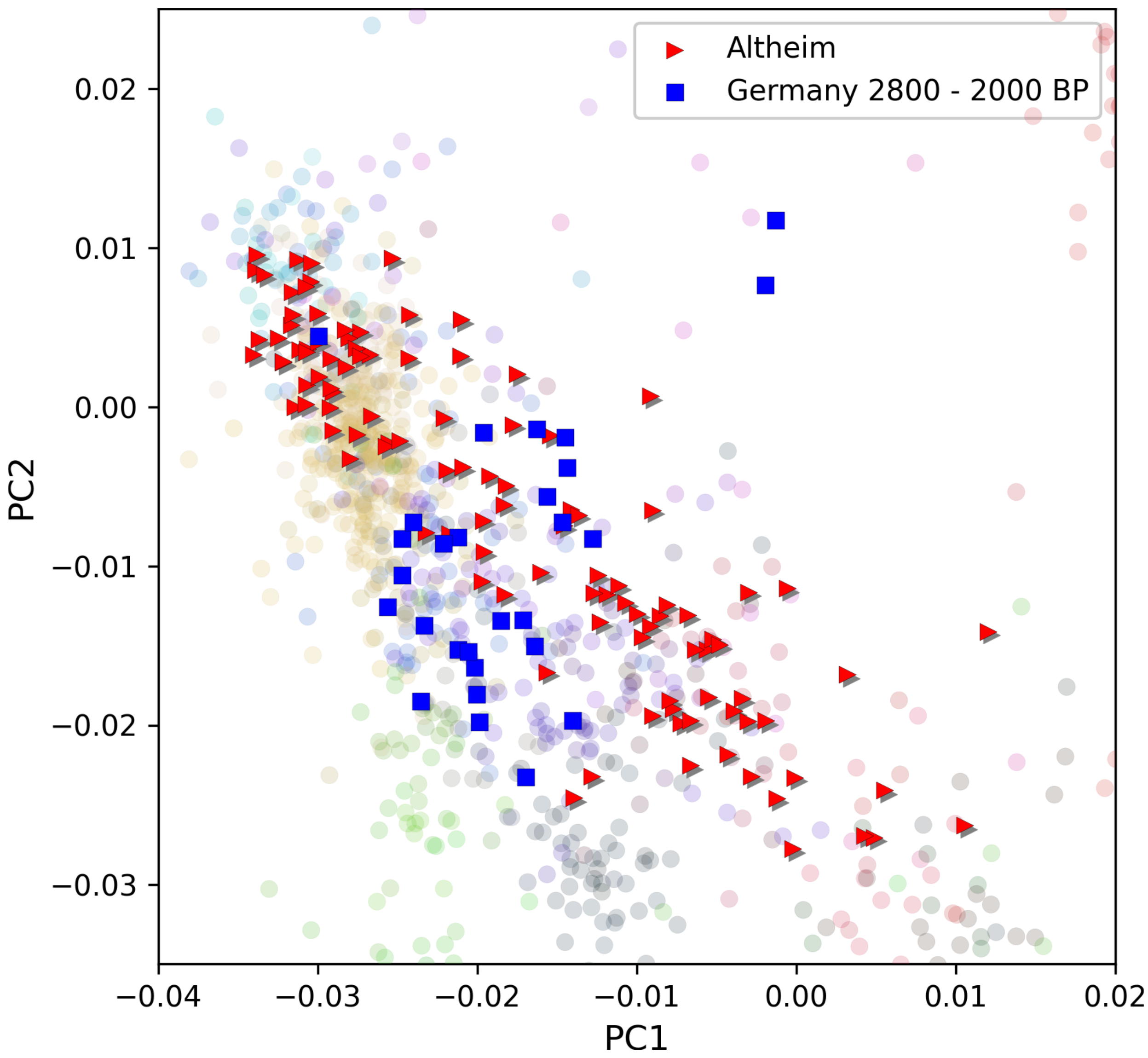

Fig. S7.9: *PCA plot showing individuals from the Altheim site (red) alongside Iron Age individuals from southern German sites (blue, [209]). Only partial overlap between the Altheim and earlier Iron Age individuals is observed.*

S8. Ancestry Modeling

Ancestry inference is common practice in population genetics and can be performed in multiple ways. Even though methods relying on allele frequencies (qpAdm, ADMIXTURE, etc.) are quite powerful in characterizing broad ancestries, if source groups are well defined, they can have a hard time detecting subtle differences. To have a more fine-scaled characterization of potential ancestries in our dataset we applied three different methods to three different datasets. To leverage the availability of genome-wide data of our shotgun sequenced genomes we applied the non negative least squares (NNLS) approach implemented in *twigstats* [22] on a set of genealogies inferred with *Relate* [23], using a set of shotgun-only genomes. In addition we used the Bayesian ancestry inference method implemented in *sourcefindV2* [74] (https://github.com/hellenthal-group-UCL/sourcefindV2) on a set of haplotype copying vectors inferred by *ChromoPainter2* implemented in *finestructure4* [24] to re-evaluate the ancestries inferred with the previous approach. To increase the number of available reference genomes we also ran the same analyses on a larger set, including individuals sequenced on the 1240K capture array, while limiting to the autosomal chromosomes of the 1240K panel. As a third step of validation we ran the NNLS approach implemented in *PANE* [25], allowing for an even larger set of reference genomes, as *PANE* does not require imputation. Here we focussed on modelling our newly sequenced individuals as mixtures of populations pre-defined by previous studies.

Relate ancestry painting (distal sources)

To further characterise the origins of the individuals investigated here, we applied the ancestry painting method based on genealogies inferred by *Relate* [23] introduced by *twigstats* [22]. We clustered available reference genomes from Iron Age and Late Antiquity using a graph-based approach utilizing genetic and geographic information. We first tested all single-source pairwise qpAdm models computed on *twigstats* f_2 statistics with a cutoff of 1,000 generation to test for cladality, using CEU, GBR, FIN, IBS, TSI, CHB and YRI populations from 1000 genomes as outgroups (right groups) [22]. Then, within broadly defined geographic regions we created an edge between two individuals if a valid qpAdm model existed ($p>0.05$), and applied Louvain clustering to the resulting graph with resolution parameter set to 0. Groups with fewer than 5 individuals were ignored. One group geographically located predominantly in Northern Poland, but also in Germany and Slovakia was overlapping, in PCA space (Fig. S8.1), with the group located in Scandinavia; for that reason and considering their geographical proximity (all but two individuals in said group come from Northern Poland) we merged the two groups in the *Northern Europe* group. We additionally included CHB as a source to act as a proxy for East Asian ancestry.

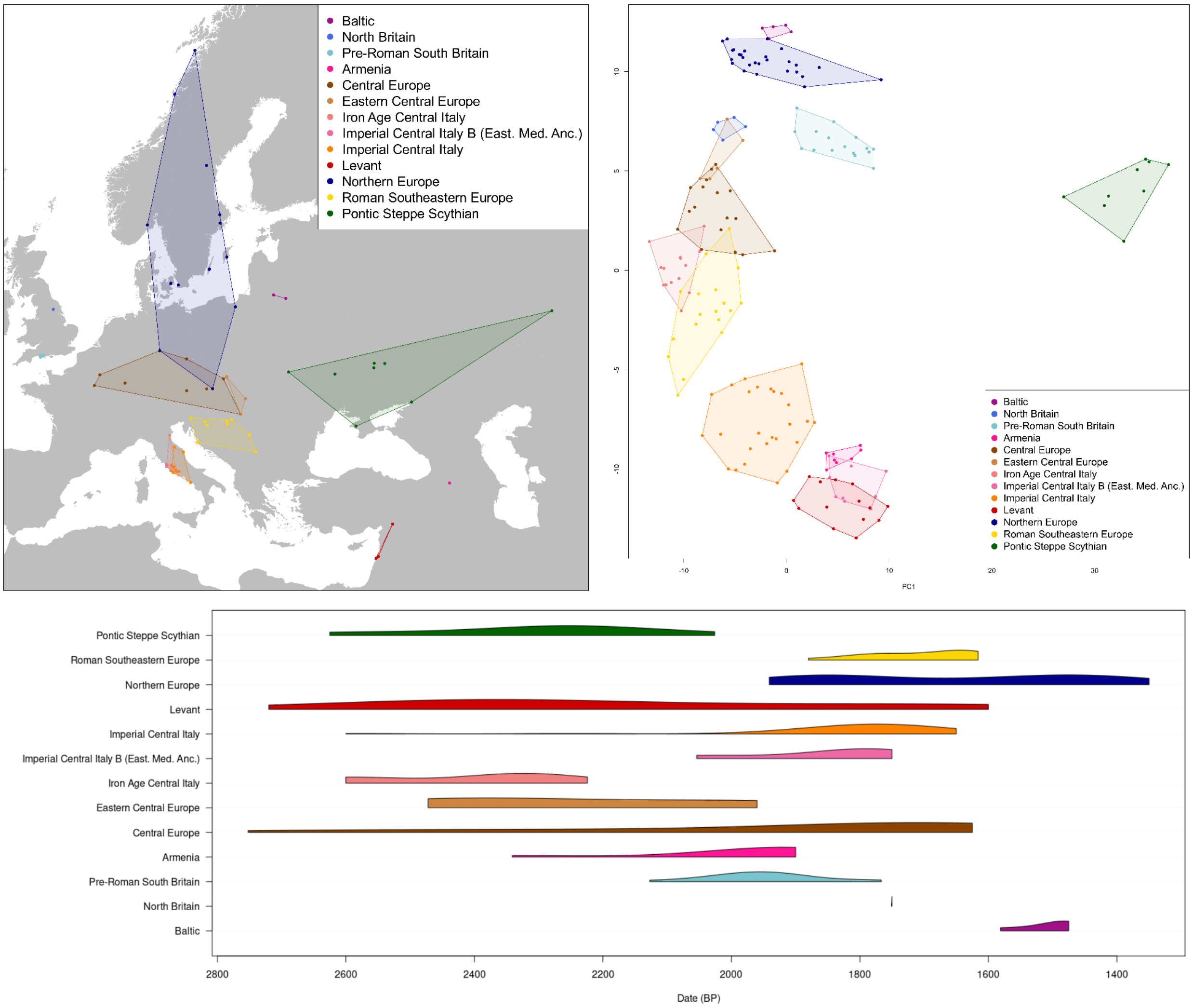

***Fig. S8.1:** Geographic distribution of the individuals used as sources for ancestry modeling (upper left panel), and their genetic structure visualised with a PCA based on pairwise f_3-outgroup statistics computed based on twigstats f_2 values using a cutoff of 1000 generations and CHB as outgroup (upper right panel). Chronological distribution is shown in the bottom panel*

The *Northern Europe* source group results as the main contributor (~33% across all individuals) to the ancestry of Early Medieval Germany (Fig. S8.2), followed by *Roman Southeastern Europe* (18%), *North Britain* (14%), *Iron Age Central Italy* (13%) and *Imperial Central Italy* (7%). Other ancestries are present but to a lower extent.

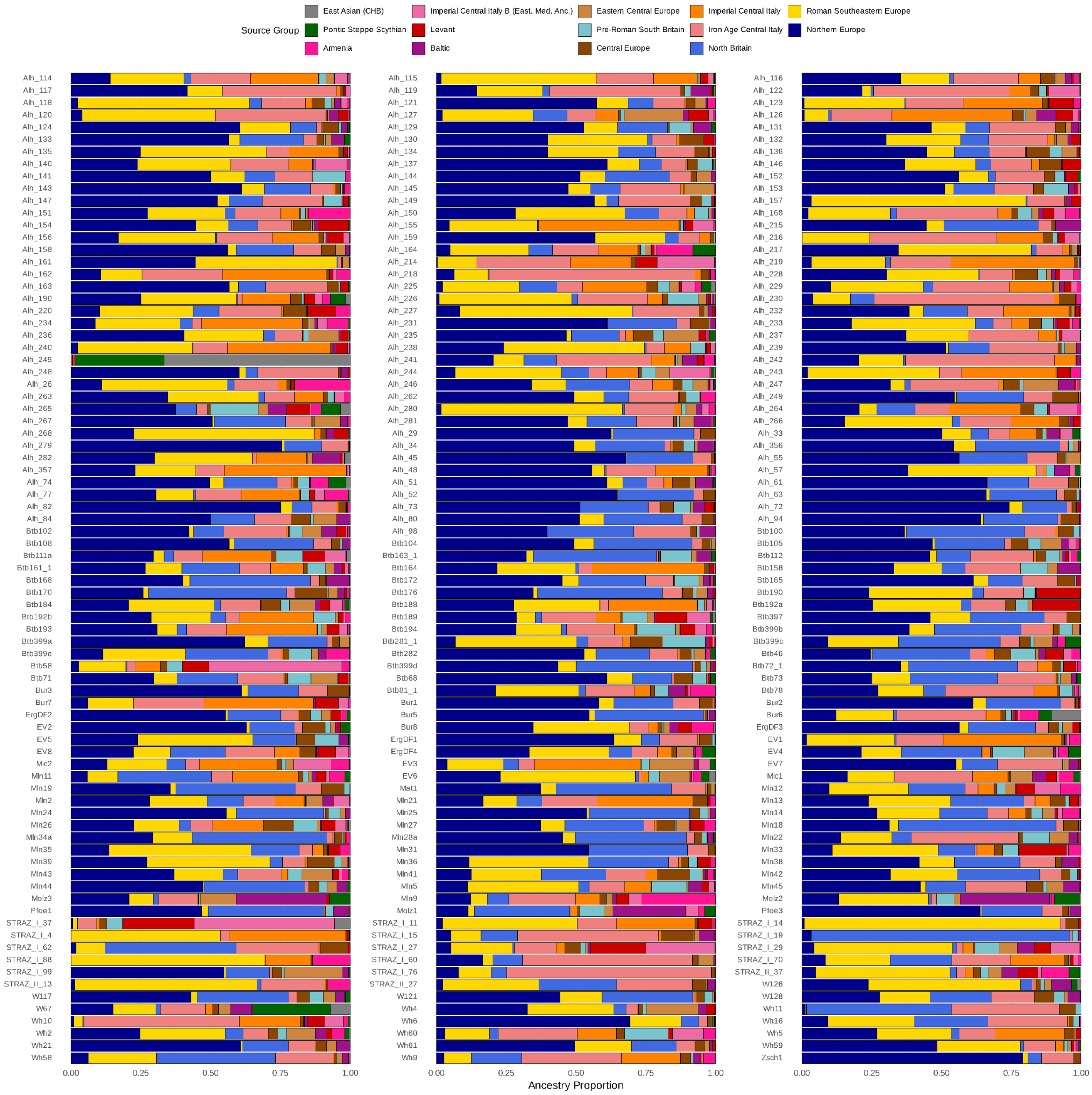

***Fig. S8.2:** Individual ancestry proportions of Late Antiquity and Early Medieval genomes from Germany, inferred with nnls regression from twigstats' paintings on Relate genealogies.*

To investigate the co-occurrence of certain ancestries in our target individuals, and due to the compositional nature of the data (i.e., ancestry proportions summing up to one), we applied a centered log-ratio (CLR) transformation to the ancestry proportion matrix. Pearson correlation coefficients were then calculated on the CLR-transformed matrix for all pairwise observations. strong positive correlation can be detected between *Northern Europe*, *North Britain* and *Central Europe*, meaning that a higher proportion of one of these sources usually results in a higher proportion of the others too (Fig. S8.3). The two most relevant "southern" source groups: *Roman Southeastern Europe* and *Iron Age Central Italy*, show a weaker but significant correlation, and *Imperial Central Italy* correlates with almost all the southern sources. Finally, ancestry from *Pontic Steppe Scytians* positively correlates with *East Asian* ancestry.

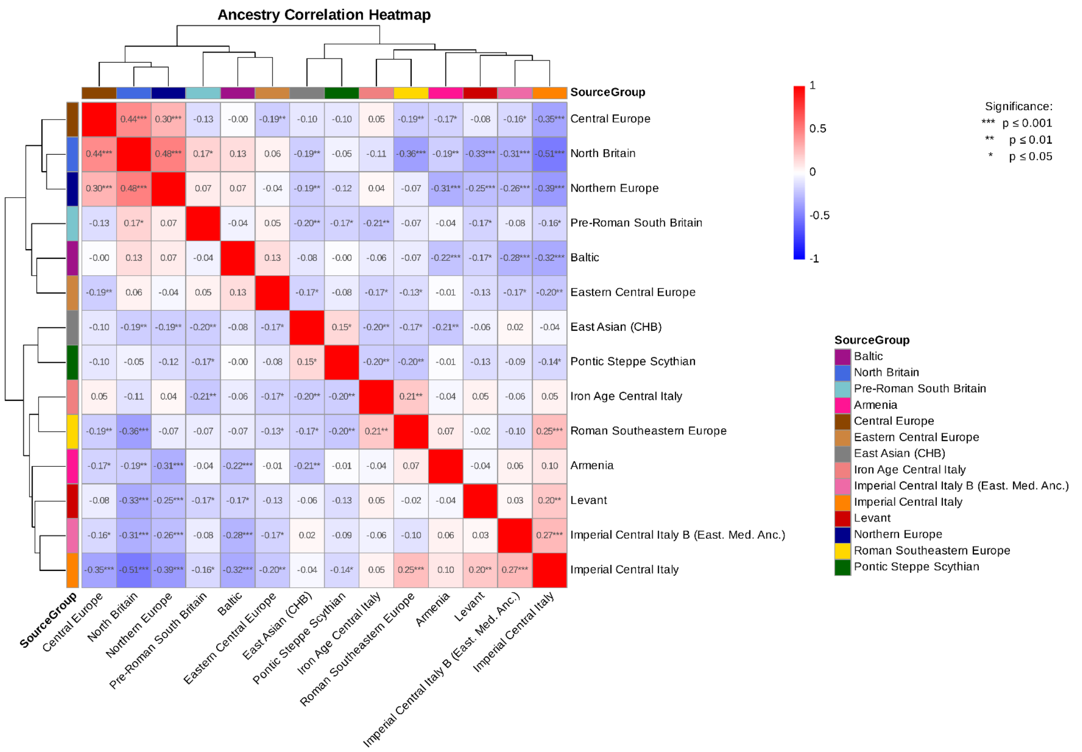

***Fig. S8.3:** Ancestry correlation in Early Medieval samples from Germany. Pearson's correlation coefficient was computed on the centered log-ratio (CLR) transformation of the ancestry proportion matrix for each pairing.*

Ancestry from the steppe region in overall limited (~1%), but a handful individuals are modeled as deriving >5% of ancestry from them (Alh_245: ~32%, W67: ~28%, Molz2: ~9%, Alh_164: ~8%, Molz3 and Alh_265: ~7%, Alh_74: ~6% and Alh_190: ~5%). A similar picture but to a slightly higher extent emerges from Baltic ancestry, with 20 individuals showing more than 5% contribution (but never more than 10%); and the three Slavic individuals from Molzbichl showing the highest proportions of such ancestry estimated between 26% and 32%. Levantine ancestry, overall contributing ~2% of ancestry, shows higher proportion in a handful of individuals (Mln33 and Btb192a: ~17%, Btb190: ~16%, Btb189 and Alh_154: ~11%, Alh_220 and Btb58: ~10). Armenian ancestry is found at >10% in four individuals (Mln9: 26%, Alh_26: 20%, Alh_151: 15%, Alh_164: ~13%). Finally, east asian ancestry is the main component of Alh_245 (~66%) and plays a minor role in two other individuals (Bur6: ~11%, W67: ~7%).

We identified some regional differences in ancestry proportions between the Danube-Isar and the Rhine-Main regions, with the former exhibiting a higher, on average, Iron Age Italian ancestry and the latter higher proportions of ancestry from Britain, Eastern Central Europe and the Baltic ($p<0.05$, one-sided Wilcoxon test, Fig. S8.4).

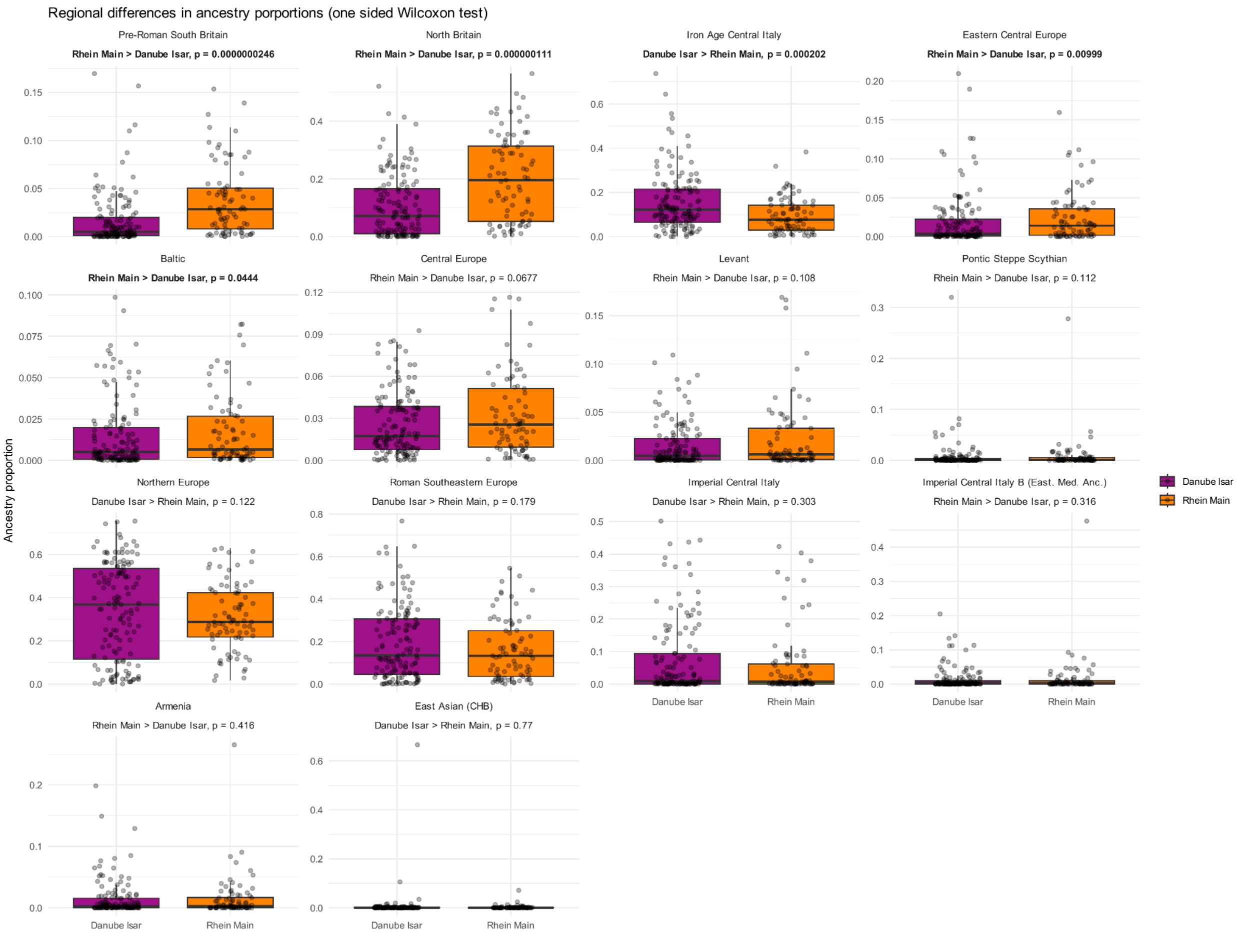

Fig. S8.4: *Boxplots comparing the inferred ancestry proportion for each source population between the Danube-Isar and the Rhine-Main regions. A one sided Wilcoxon test was used to establish significance*

For Altheim and Büttelborn, the finer chronological resolution achieved using *Chronograph* allowed us to trace ancestry changes through time with high precision (Fig. S8.5 - S8.7). In the initial phase of Altheim the predominant ancestry is the *Northern European* one, with *Roman Southeastern Europe* increasing slowly over time and then, after ~500 CE, showing a more abrupt increase. *Roman Southeastern European* ancestry (as well as all other “southern” ancestries with which it correlates) peaks in the second half of the 6th century, and then starts to decrease in the 7th century, until it eventually reaches a mean comparable to the early 5th century.

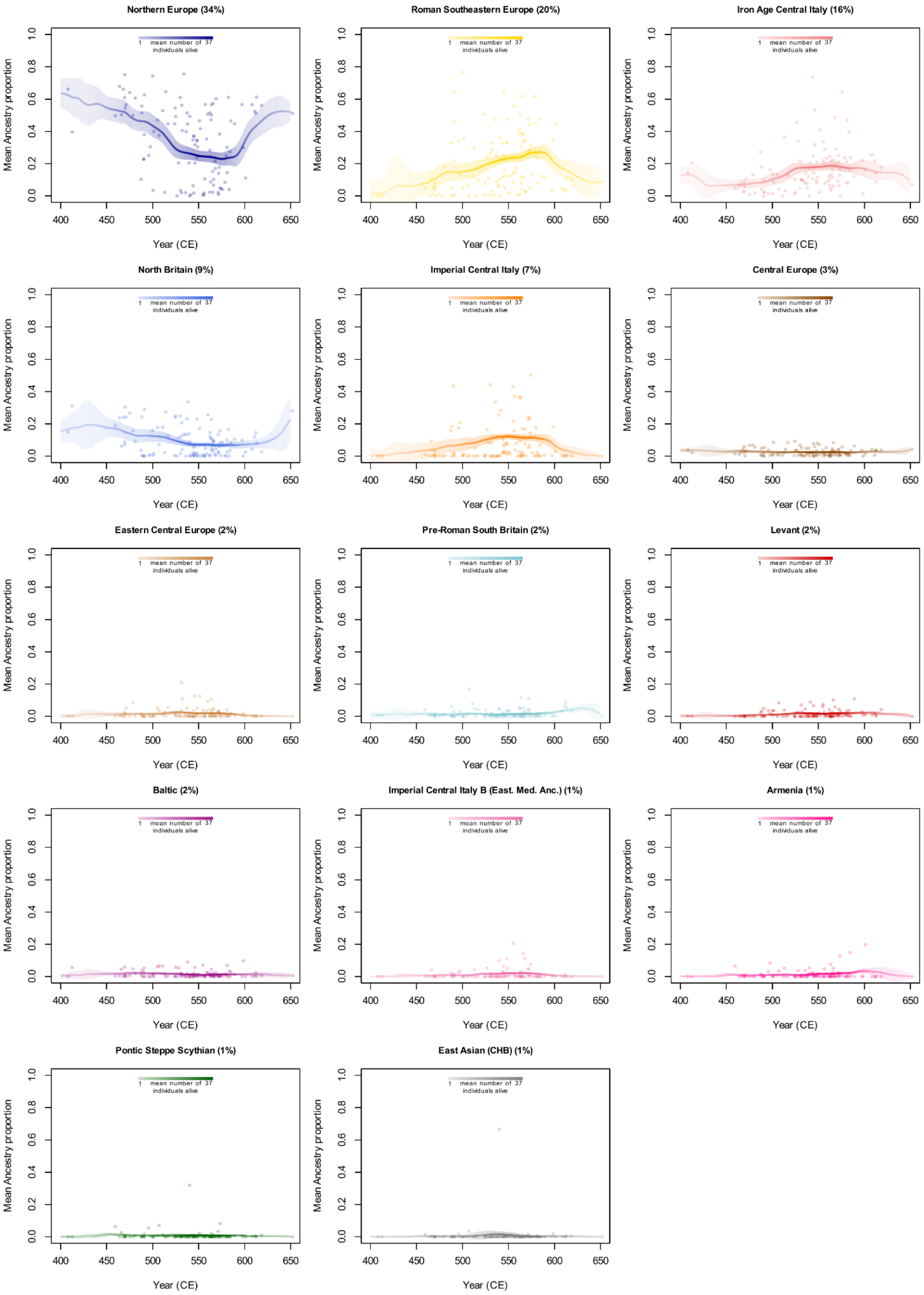

Fig S8.5: *Ancestry changes through time in the Altheim population. The line shows the mean ancestry of all individuals alive at a given time and the shaded area represents ± 1.96 standard deviations. The alpha value of the mean line is proportional to the number of individuals alive at that time. The dots represent the ancestry proportion of single individuals in the midpoint of their life.*

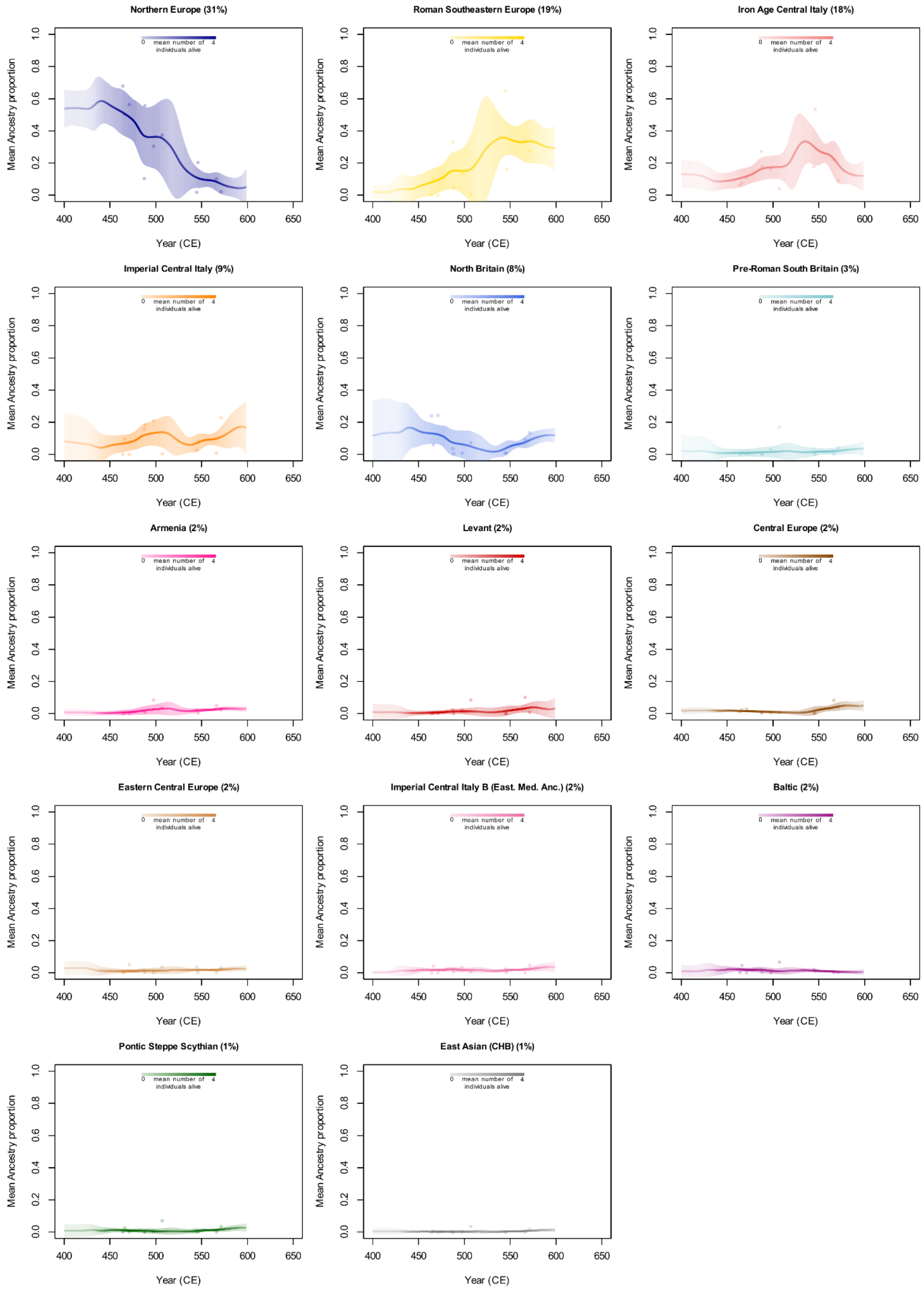

Fig S8.6: *Ancestry changes through time in the individuals of the Altheim population identified as non-locals based on isotopic evidence. The line shows the mean ancestry of all individuals alive at a given time and the shaded area represents ± 1.96 standard deviations. The alpha value of the mean line is proportional to the number of individuals alive at that time. The dots represent the ancestry proportion of single individuals in the midpoint of their life.*

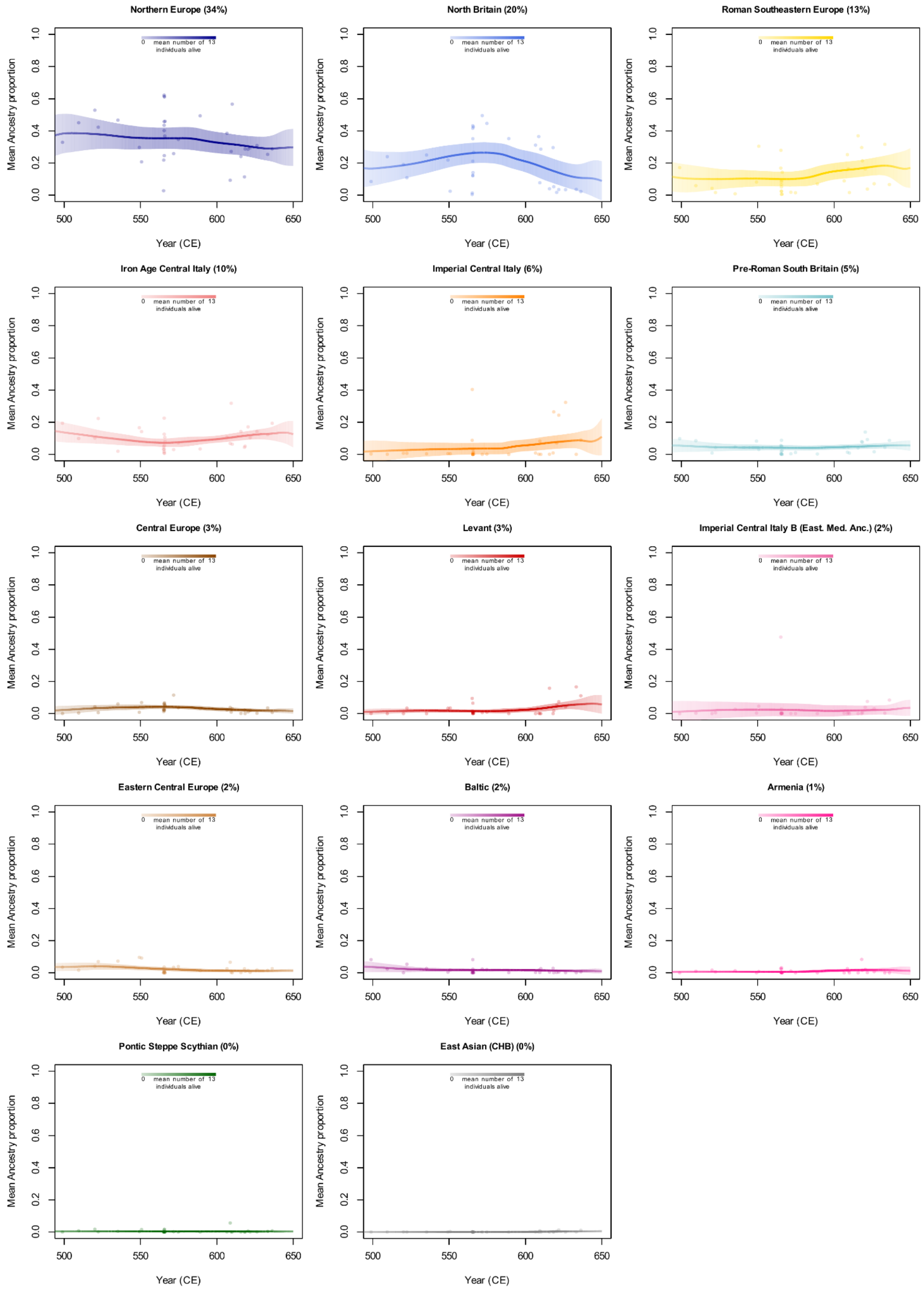

Fig S8.7: *Ancestry changes through time in the Büttelborn population. The line shows the mean ancestry of all individuals alive at a given time and the shaded area represents ± 1.96 standard deviations. The alpha value of the mean line is proportional to the number of individuals alive at that time. The dots represent the ancestry proportion of single individuals in the midpoint of their life.*

***Table S8.1**: Mean ancestry proportions for each Early Medieval site with n>1, inferred with twigsats from relate-genealogies, using distal sources.*

site	Alt	Bur	Büt	Elt	Erg	Mic	Möm	Wei	Wöl
**Baltic**	0.015	0.010	0.017	0.013	0.002	0.025	0.014	0.015	0.045
**North Britain**	0.092	0.153	0.198	0.124	0.145	0.034	0.234	0.151	0.186
**Pre-Roman South Britain**	0.017	0.005	0.045	0.036	0.016	0.009	0.029	0.021	0.026
**Armenia**	0.013	0.023	0.011	0.014	0.011	0.059	0.025	0.013	0.001
**Central Europe**	0.026	0.025	0.030	0.029	0.035	0.012	0.038	0.026	0.037
**Eastern Central Europe**	0.018	0.005	0.022	0.052	0.033	0.053	0.021	0.030	0.027
**East Asian (CHB)**	0.008	0.016	0.002	0.003	0.000	0.004	0.001	0.000	0.017
**Iron Age Central Italy**	0.155	0.128	0.102	0.084	0.083	0.165	0.092	0.203	0.072
**Imperial Central Italy B (East. Med. Anc.)**	0.014	0.006	0.023	0.014	0.001	0.071	0.011	0.015	0.000
**Imperial Central Italy**	0.075	0.068	0.057	0.119	0.017	0.202	0.051	0.066	0.007
**Levant**	0.015	0.020	0.026	0.012	0.015	0.019	0.022	0.013	0.001
**Northern Europe**	0.342	0.412	0.335	0.268	0.521	0.147	0.282	0.258	0.308
**Roman Southeastern Europe**	0.201	0.122	0.129	0.215	0.105	0.190	0.178	0.186	0.210
**Pontic Steppe Scythian**	0.008	0.007	0.005	0.016	0.016	0.011	0.002	0.003	0.063

Relate ancestry painting (local sources)

The three sources that result as the major ancestry contributor in the distal models can be substituted with sources found more locally in Late Antiquity. We replaced *Northern European* reference individuals with local Central European individuals exhibiting Northern ancestry, and substituted *Roman Southeastern Europe* and *Iron Age Central Italy* references with individuals sampled from the cemetery associated with the Roman fort at Straubing-Azlburg (Supplementary Table 2.4). These local sources can effectively substitute distal sources used in the previous model, producing minimal changes to the overall results.

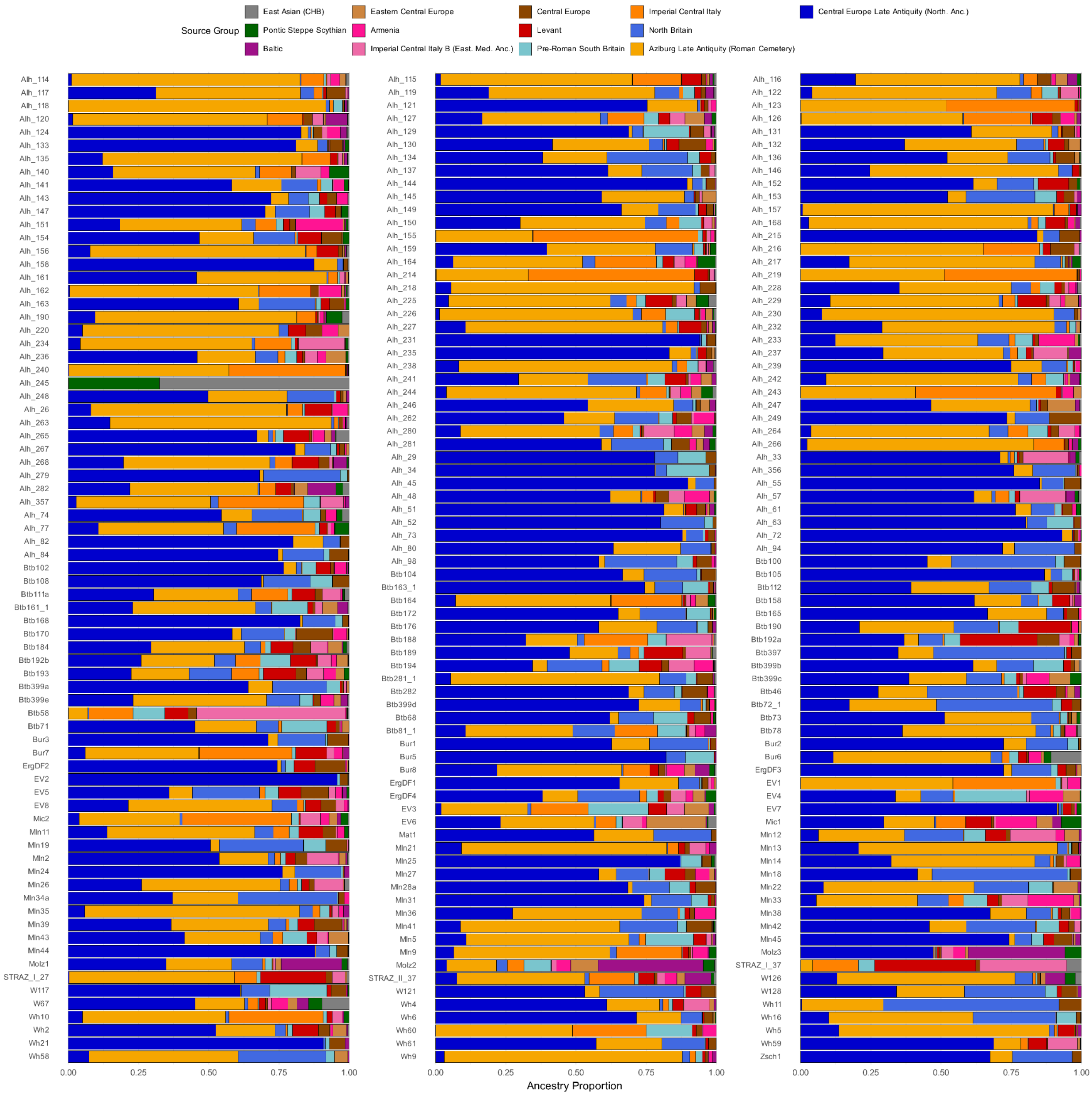

Fig. S8.8: *Ancestry proportions for Late Antiquity and Early Medieval genomes from Germany using local sources, inferred with nnls regression from twigstats' paintings on Relate genealogies.*

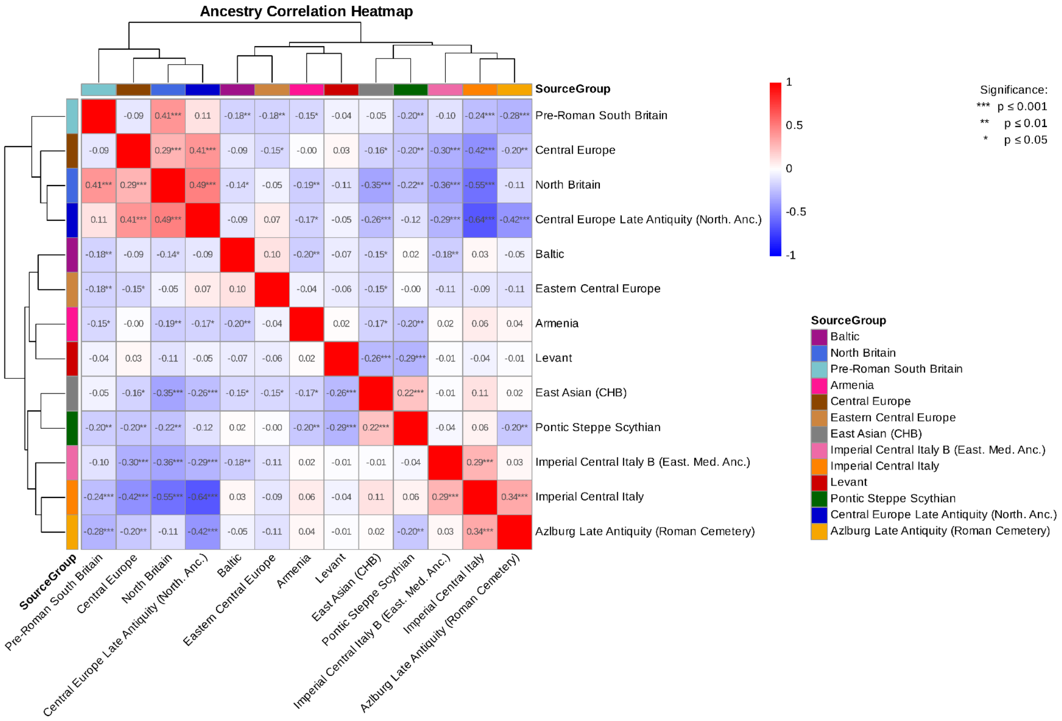

Fig. S8.9: *Ancestry correlation in Early Medieval genomes from Germany using local sources. Pearson's correlation coefficient was computed on the centered log-ratio (CLR) transformation of the ancestry proportion matrix for each pairing.*

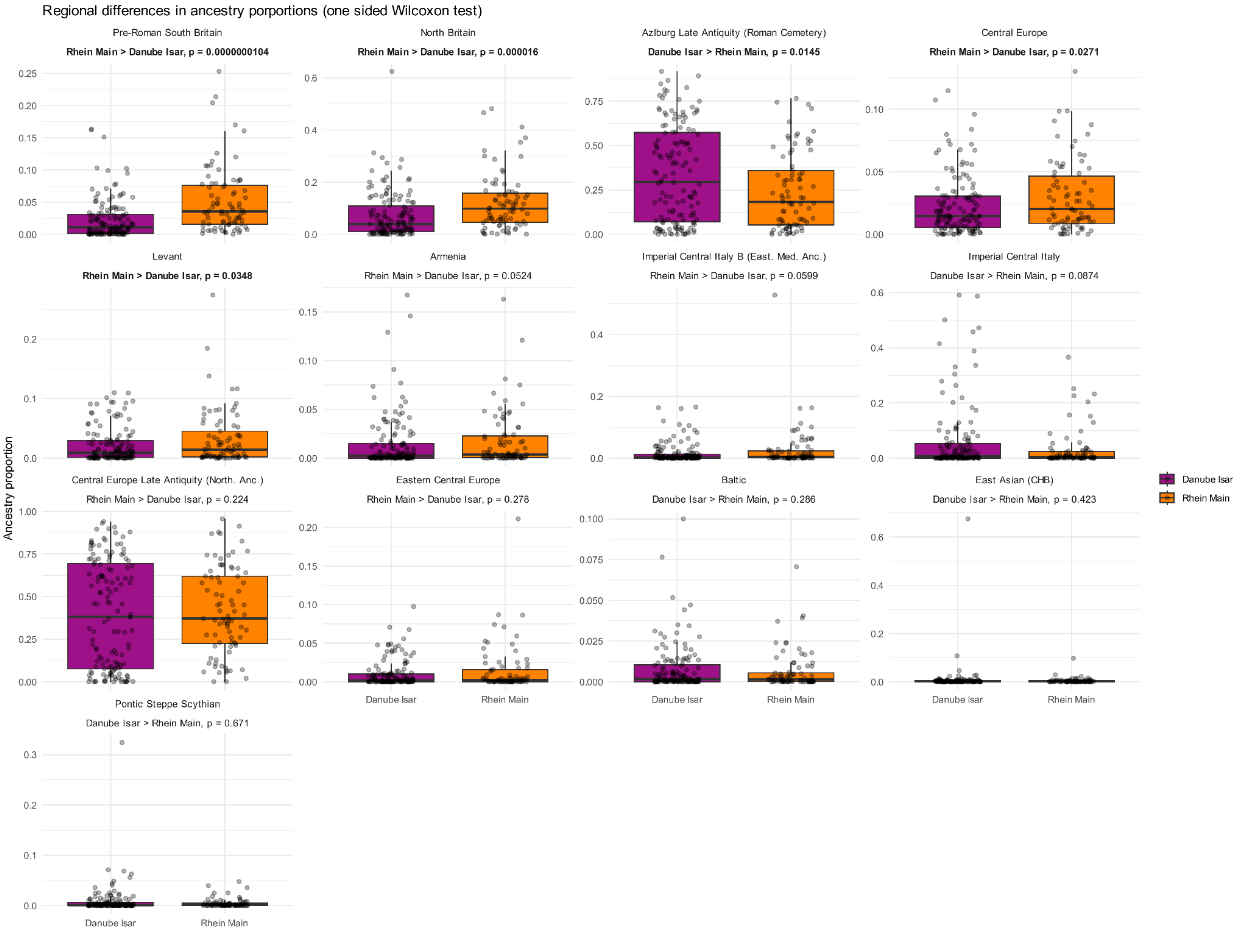

Fig. S8.10: *Boxplots comparing the inferred ancestry proportion for each source population between the Danube-Isar and the Rhine-Main regions. One sided Wilcoxon test was used to establish significance.*

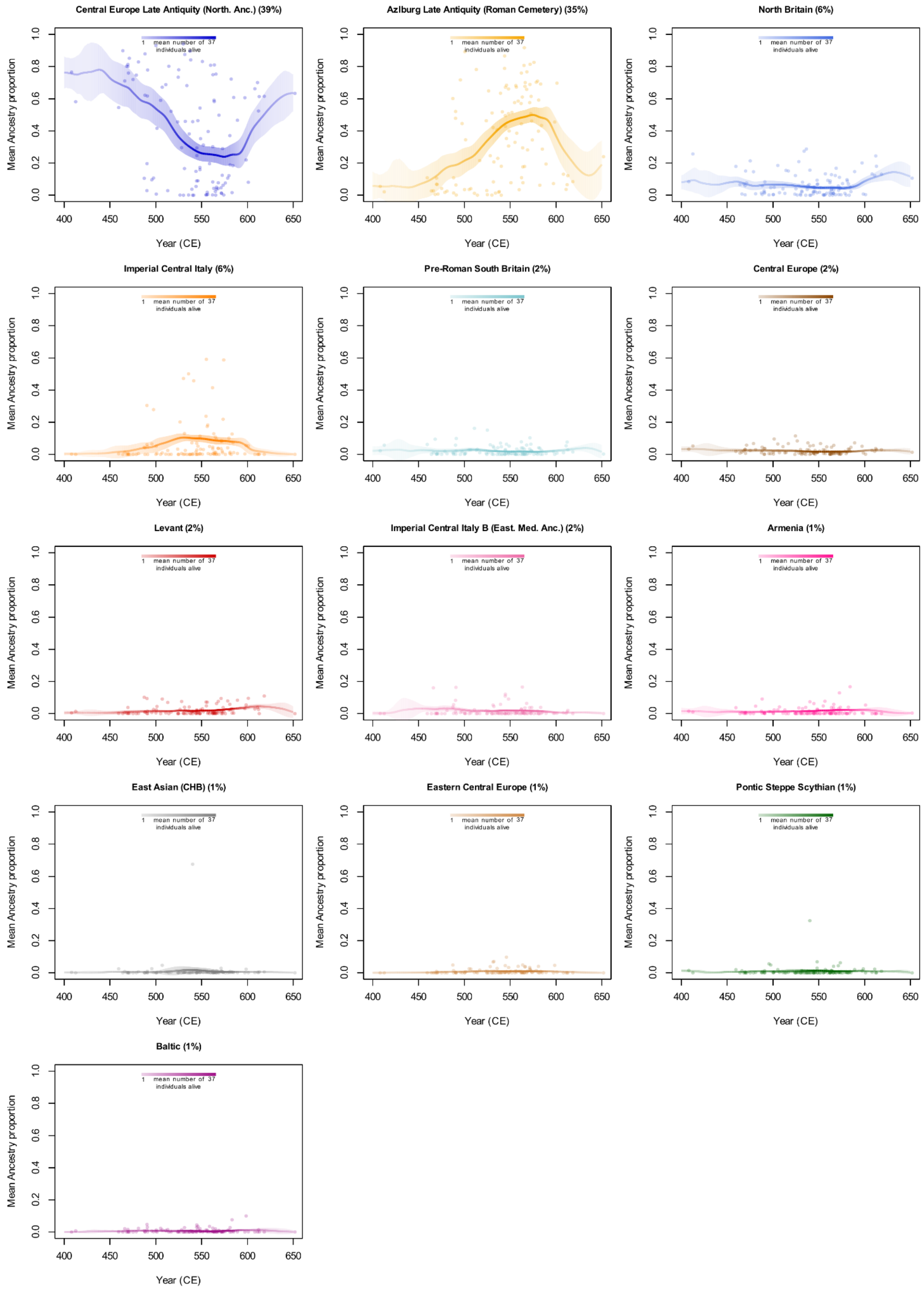

Fig S8.11: *Ancestry changes (using local sources) through time in the Altheim population. The line shows the mean ancestry of all individuals alive at a given time and the shaded area represents ± 1.96 standard deviations. The alpha value of the mean line is proportional to the number of individuals alive at that time. The dots represent the ancestry proportion of single individuals in the midpoint of their life.*

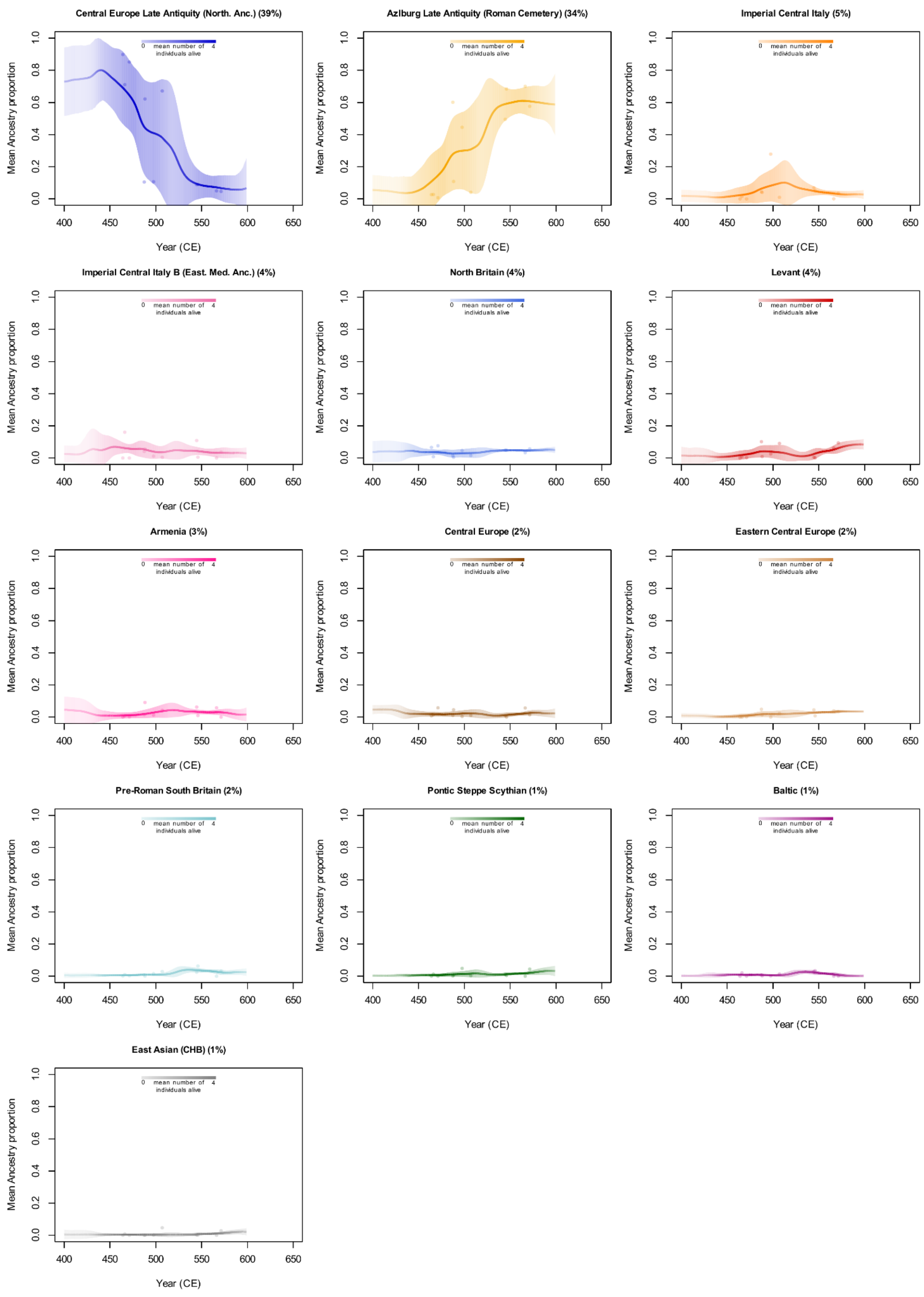

Fig S8.12: *Ancestry changes (using local sources) through time in the individuals of Altheim identified as non-locals based on isotopic evidence. The line shows the mean ancestry of all individuals alive at a given time and the shaded area represents ± 1.96 standard deviations. The alpha value of the mean line is proportional to the number of individuals alive at that time. The dots represent the ancestry proportion of single individuals in their life midpoint.*

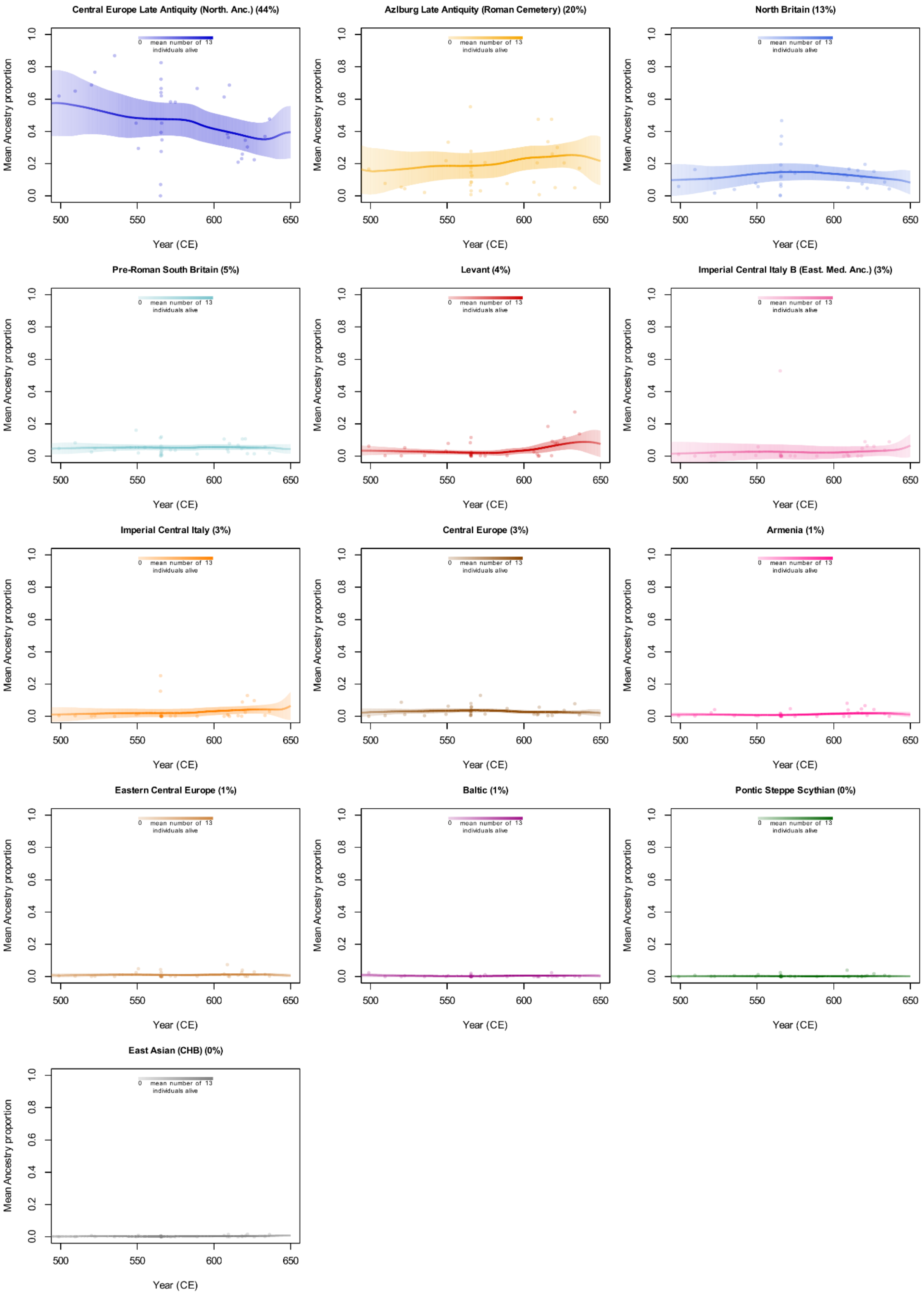

Fig S8.13: *Ancestry changes (using local sources) through time in the Büttelborn population. The line shows the mean ancestry of all individuals alive at a given time and the shaded area represents ± 1.96 standard deviations. The alpha value of the mean line is proportional to the number of individuals alive at that time. The dots represent the ancestry proportion of single individuals in the midpoint of their life.*

ChromoPainter2/sourcefind

To validate the *twigstats* results and to enable the inclusion of genomes sequenced using the 1240K capture array [65] for ancestry inference, we additionally applied the haplotype based chromosome painting approach implemented in *ChromoPainter2* [24] in combination with *sourcefindV2* to our dataset.

Validation
For the validation of the *twigstats* results we used the same VCF files used in the previous analyses and converted them to *ChromoPainter2* format. The switch rate (-n) and global mutation rate (-M) required to run *ChromoPainter2* were estimated with 10 EM iterations on 50 randomly selected individuals, using chromosomes 1,2,21,22. The resulting estimates were averaged over all chromosomes, giving a switch rate of 510 and a mutation rate of 0.0002532.

Using the clusters inferred in the *twigstats* analysis as donors we painted all individuals in the data-set, creating copying vectors that were then combined, using the provided perl-script. We used 50 independent runs of *sourcefind* for each individual, with 50,000 burn-in iterations and 200,000 sample iterations, recording every 5,000th iteration, with the expected number of surrogates set to 2, while allowing a total of 4. As final estimates we averaged the results with the highest posterior probability of each of the 30 runs.

Results for Altheim are displayed in Figure S8.14. While individual estimates slightly varied between ancestries inferred by *relate/twigstats* and *ChromoPainter2/sourcefind*, the overall patterns were highly concordant. The source groups with the highest contribution to the ancestries found in Altheim were of Northern European origin, in addition to South-Eastern European and Iron Age Italy.

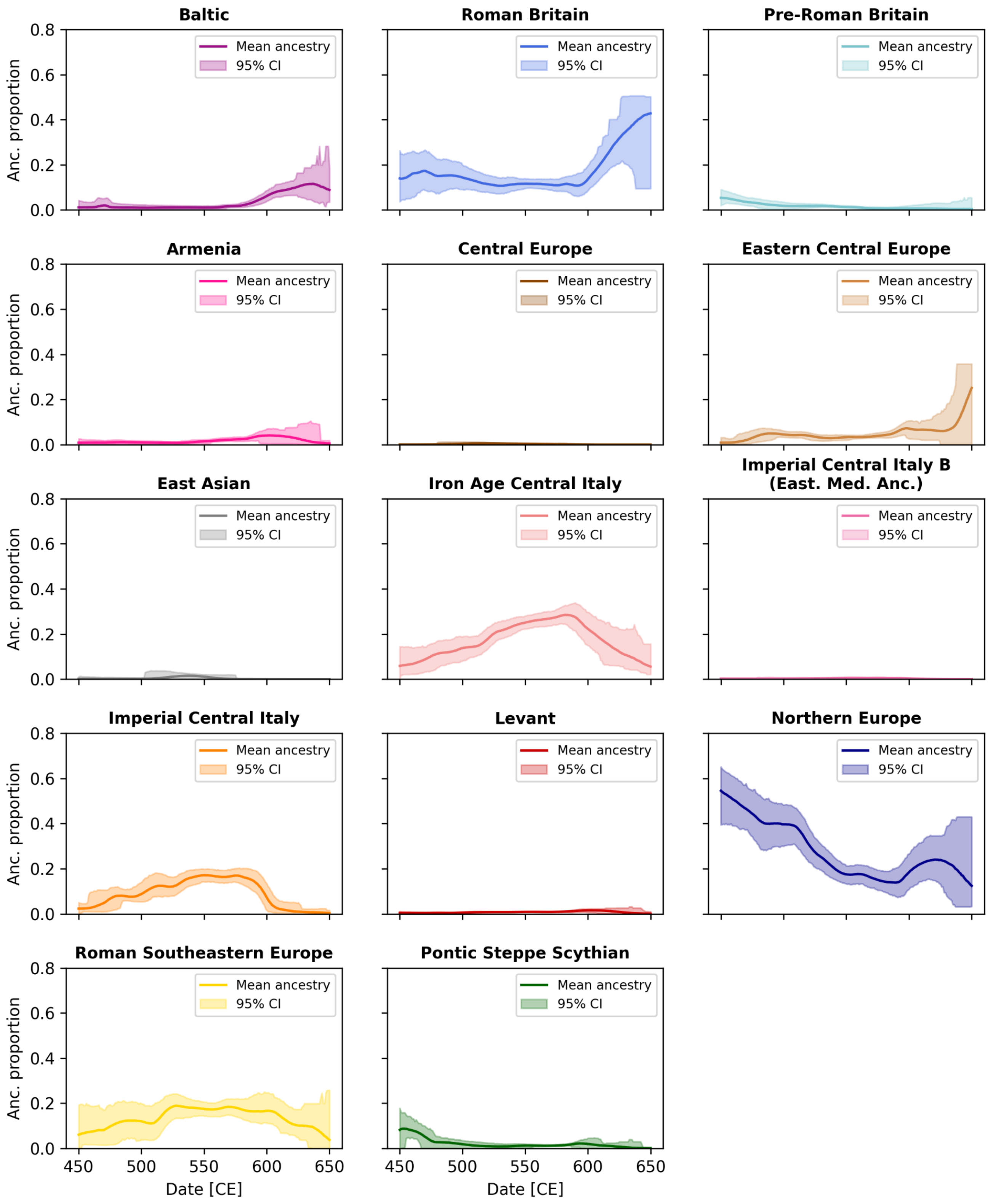

Fig. S8.14: *Average ancestry inferred by sourcefind based on ChromoPainter2 copying vectors for all individuals estimated to be alive at a given time in Altheim, sampled from 15000 MCMC chains, using the same SNPs and source-groups as in the relate/twigstats models described above. 95% CI's are computed as the 2.5th and 97.5th quantile of the data.*

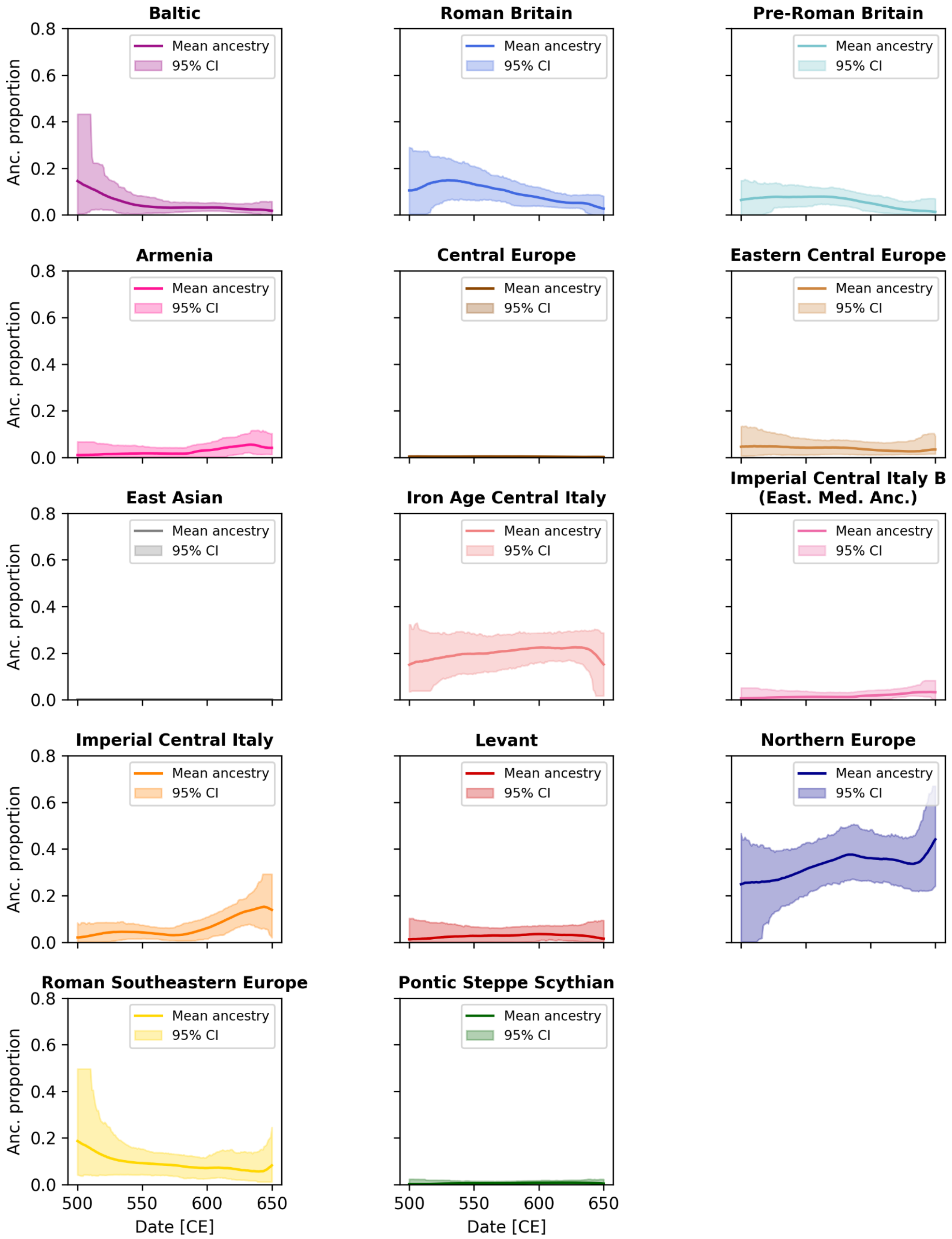

Fig. S8.15: *Average ancestry inferred by sourcefind based on ChromoPainter2 copying vectors for all individuals estimated to be alive at a given time in Büttelborn, sampled from 15000 MCMC chains, using the same SNPs and source-groups as in the relate/twigstats models described above.*

Additional source-groups

To enable ancestry estimation with a larger set of source groups, we sub-sampled our dataset to include only the autosomal chromosomes targeted by the 1240K capture array [65]. We merged this data set with all available genomes suitable for imputation from 2800 BC to 800 CE. We estimated the switch rate and mutation rate as described above, resulting in -n 207 and -M 0.00013346. Next we ran *ChromoPainter2* on all chromosomes for a subset of post-Roman individuals (Supplementary Table 2.7), allowing each individual to be painted by every other genome (-a option). We combined the resulting copying-vectors with the provided scripts and calculated the euclidean distance for all possible pairs of samples. Similar to the clustering approach described above, we built a graph, connecting all individuals that were within a euclidean distance lower or equal to the 2.5 percentile of the overall distances and were found on archaeological sites less than 500km apart from each other, using the geographical distance as weight on the edges of the graph. We applied louvain clustering and removed clusters with less than five individuals. This approach resulted in 14 clusters of 191 individuals (Supplementary Table 2.7 and Fig. S8.16-18) that spread over large parts of Western Eurasia. Finally we ran *ChromoPainter2* creating copying vectors for all of our Early Medieval genomes using the previously defined groups as sources. Following the approach described above we combined the resulting copying-vectors and ran *sourcefind* the same way.

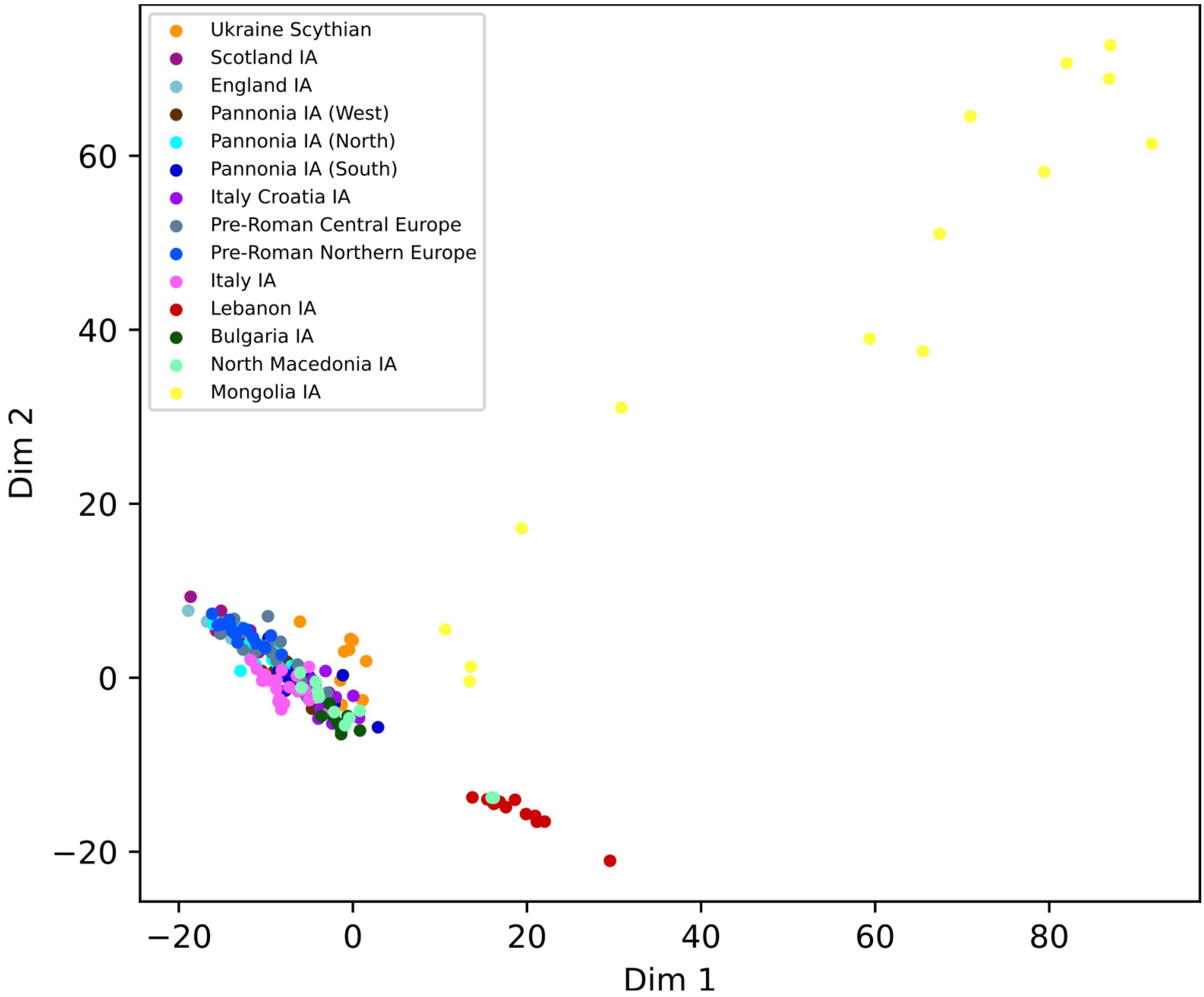

Fig. S8.16: MDS plot based on ChromoPainter2 copying vectors for the source groups identified in the geographic/genetic clustering approach.

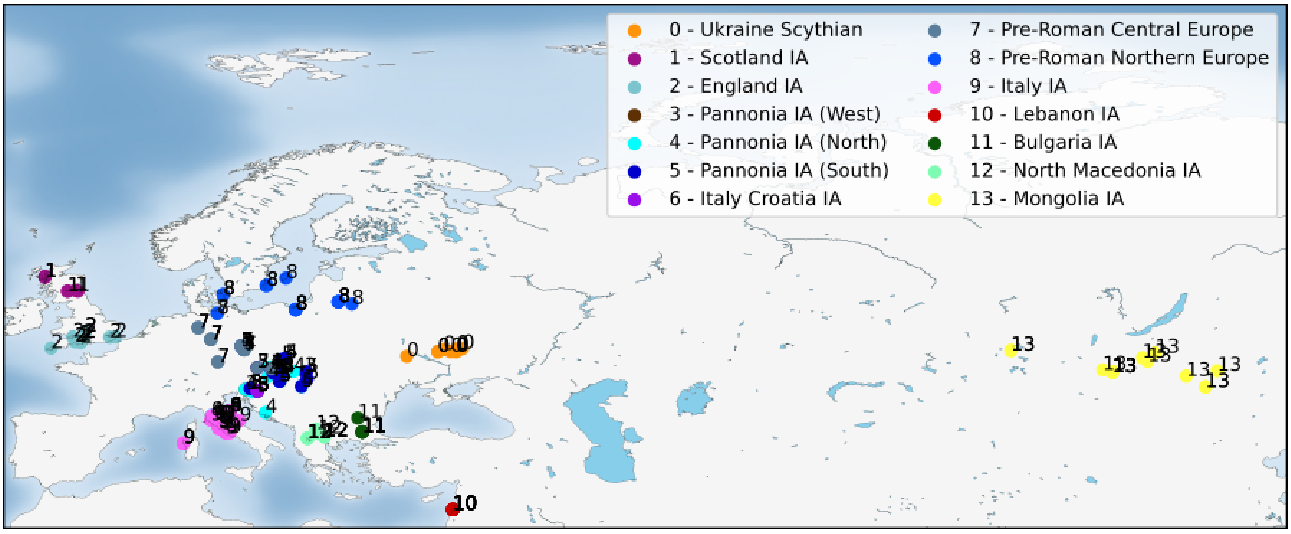

***Fig. S8.17**: Map showing the geographic origins of source groups used in the additional ChromoPainter2 analyses.*

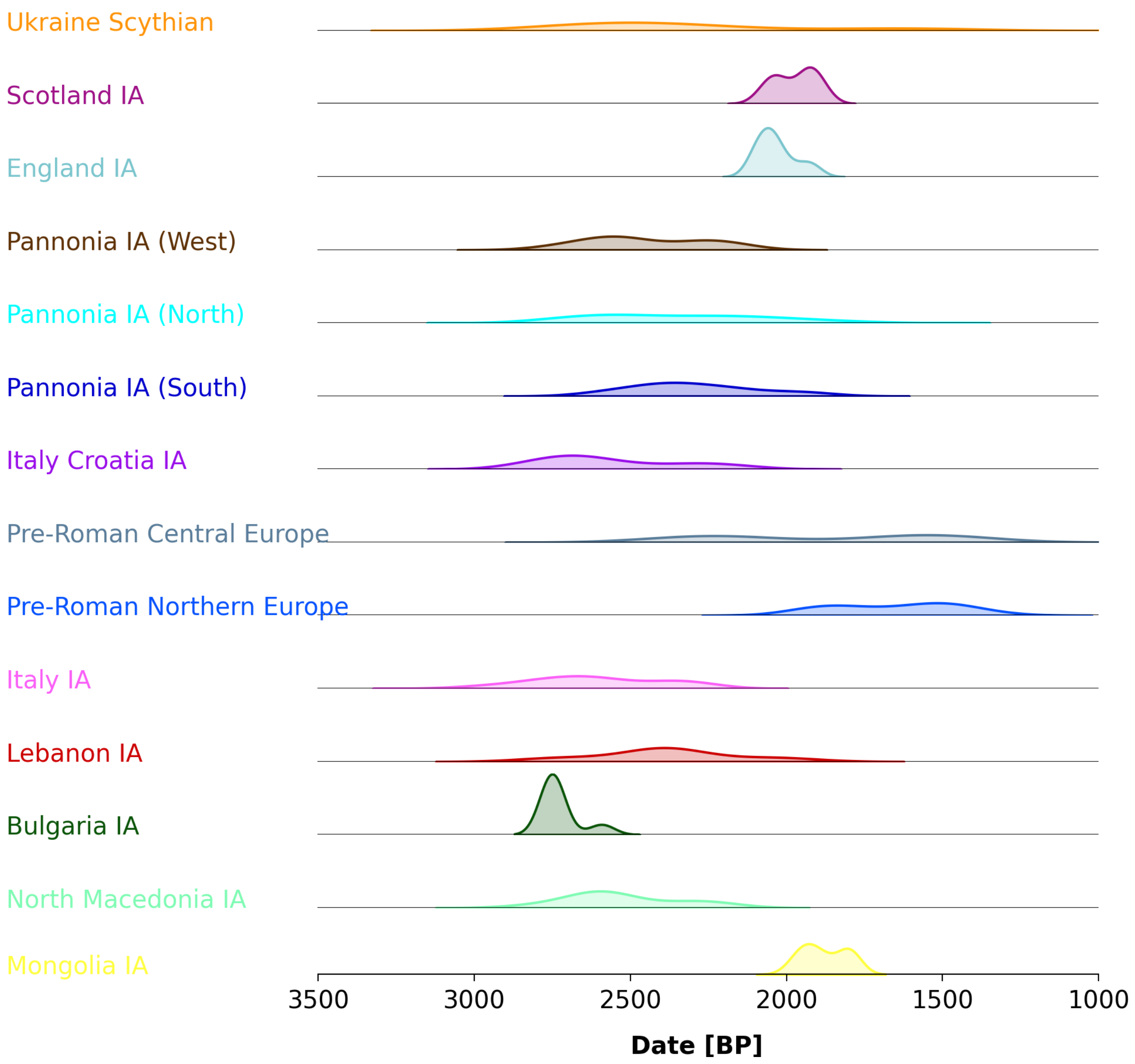

Fig. S8.18: *Distribution of dates in source groups identified by genetic/geographic clustering.*

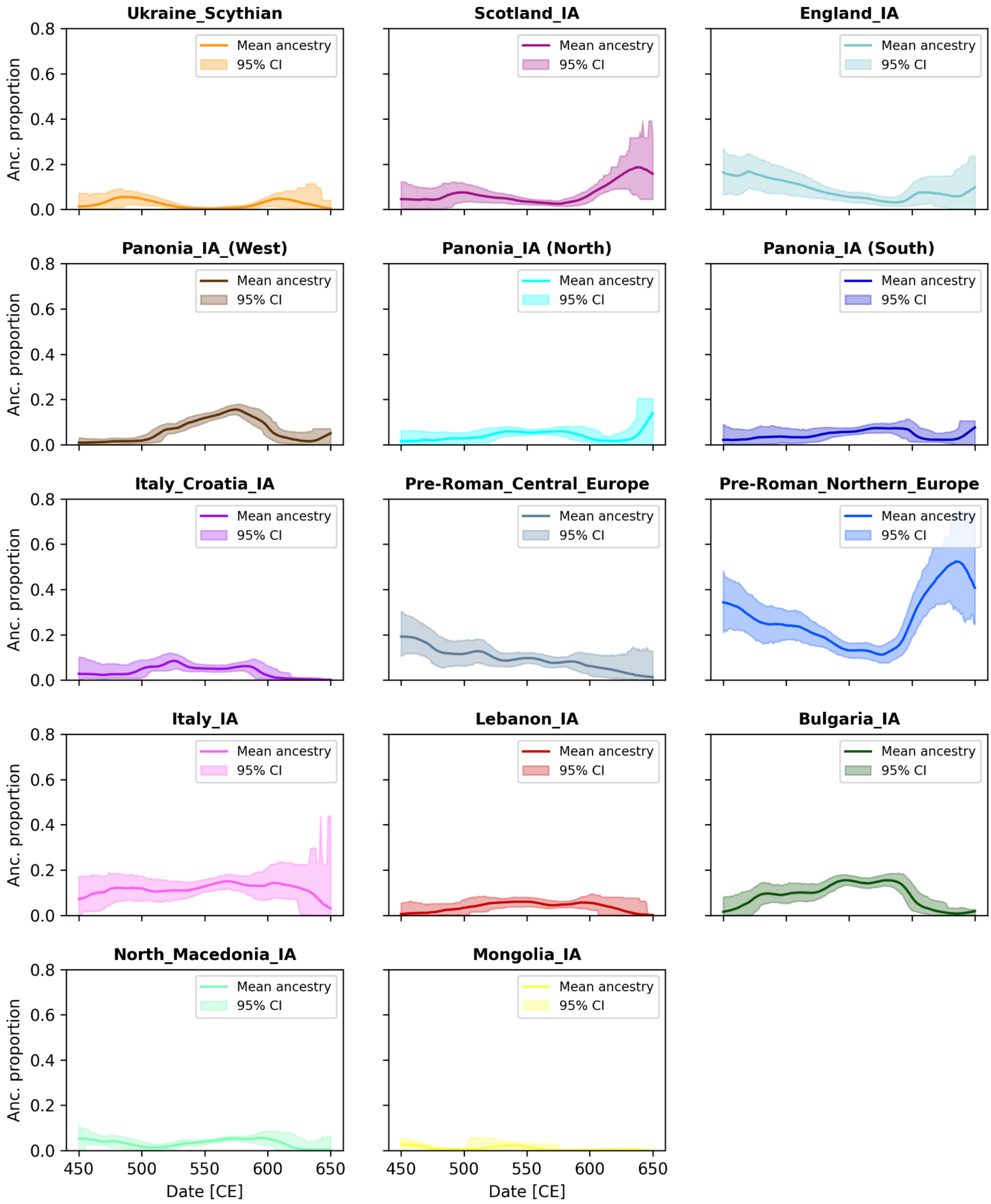

Fig. S8.19*: Average ancestry inferred by sourcefind based on ChromoPainter2 copying vectors for all individuals estimated to be alive at a given time in Altheim, sampled from 15000 MCMC chains, using the 1240k SNPs and sources defined by clustering on copying vectors and geography. 95% CI's are computed as the 2.5th and 97.5th quantile of the data.*

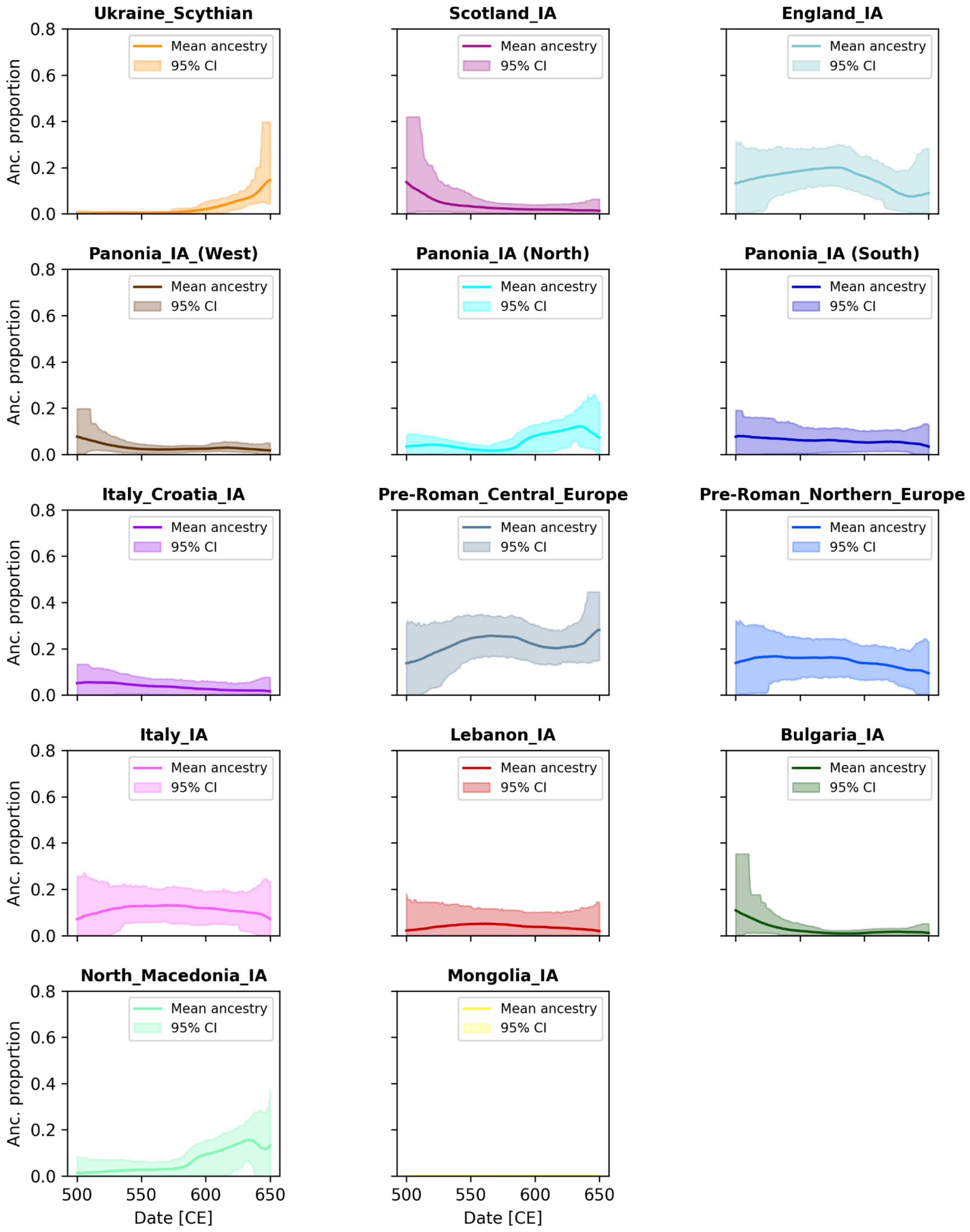

Fig. S8.20*: Average ancestry inferred by sourcefind based on ChromoPainter2 copying vectors for all individuals estimated to be alive at a given time in Büttelborn, sampled from 15000 MCMC chains, using the 1240k SNPs and sources defined by clustering on copying vectors and geography. 95% CI's are computed as the 2.5th and 97.5th quantile of the data.*

PANE

In an attempt to further validate the ancestry inferences based on phased data, we used PANE, as it allows for the incorporation of all genomes that had enough SNPs overlapping the Human Origins panel to be used in a PCA. Furthermore it allowed the modeling of individuals by pre-defined source groups also projected into the same PCA space. We choose the following 10 populations as sources: Spain_IA.AG [17,210], Italy_Tarquinia_Etruscan.SG [211], Denmark_IA.SG [212], Bulgaria_EIA.AG [213], Croatia_EIA.AG [17,213], Poland_Weklice_WielbarkCulture_Roman.SG [18], Hungary_IA_LaTene.AG [214], China_Xinjian_IA.SG [215], Russia_Sarmatian.SG [216], Estonia_IA.SG [217].

Ancestry estimates from *PANE* for genomes from Early Medieval contexts in Germany are highly similar to those inferred with *ChromoPainter2/sourcefind* and *Relate/Twigstats* (see Fig. S8.22). A general high degree of northern European ancestry can be found, represented here by genomes from Denmark and Poland, in addition to Mediterranean sources, where in the majority of cases Eastern Mediterranean ancestry is more frequent, compared to Western Mediterranean sources. Baltic ancestry, which spreads from the 6th century onwards often alongside Slavic artifacts (see Fig. S8.23, [57]) and which can be found east of our main study sites in cemeteries around Vienna, dating to the 8th century, [37] as well as further to the south in the 9th century site of Molzbichl (see Fig. S8.21), is almost absent in Early Medieval Germany. However noticeable proportions of Baltic ancestry in Genomes from modern-day northern and southern Germany indicate contact with more eastern sources at a later stage (see Fig. S8.22).

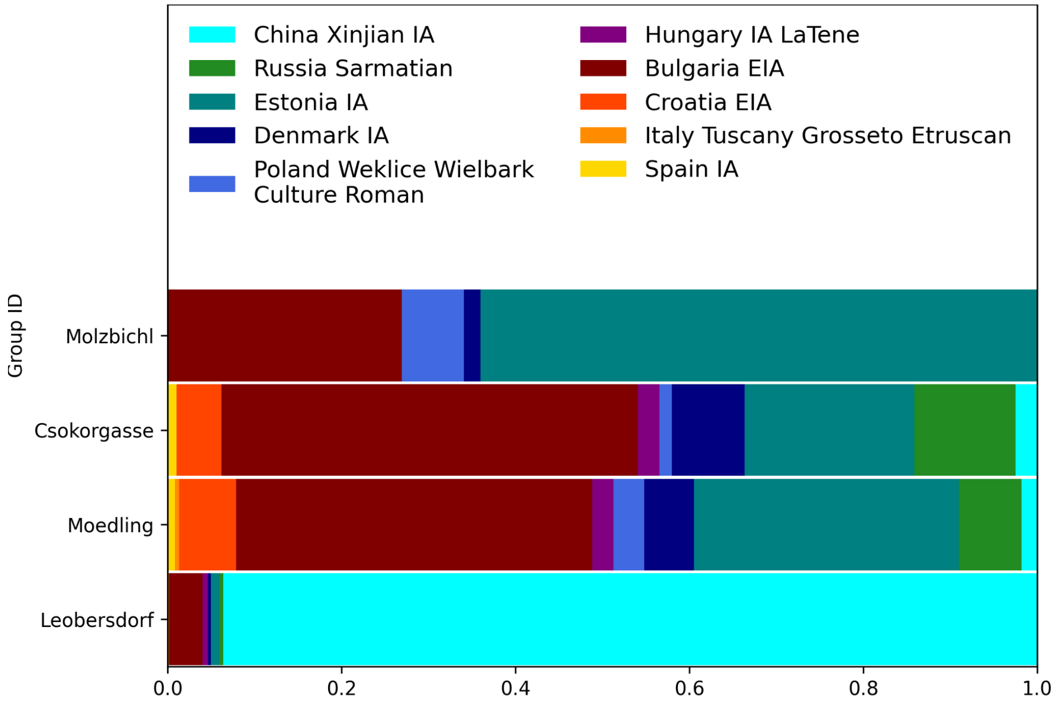

Fig. S8.21: *Results of PANE for a 10 population model. Bars represent average ancestries for four medieval sites in Austria. At all sites, except Leobersdorf* [37]*,a substantial proportion of Baltic Iron Age ancestry, represented by genomes from Estonia, is observed.*

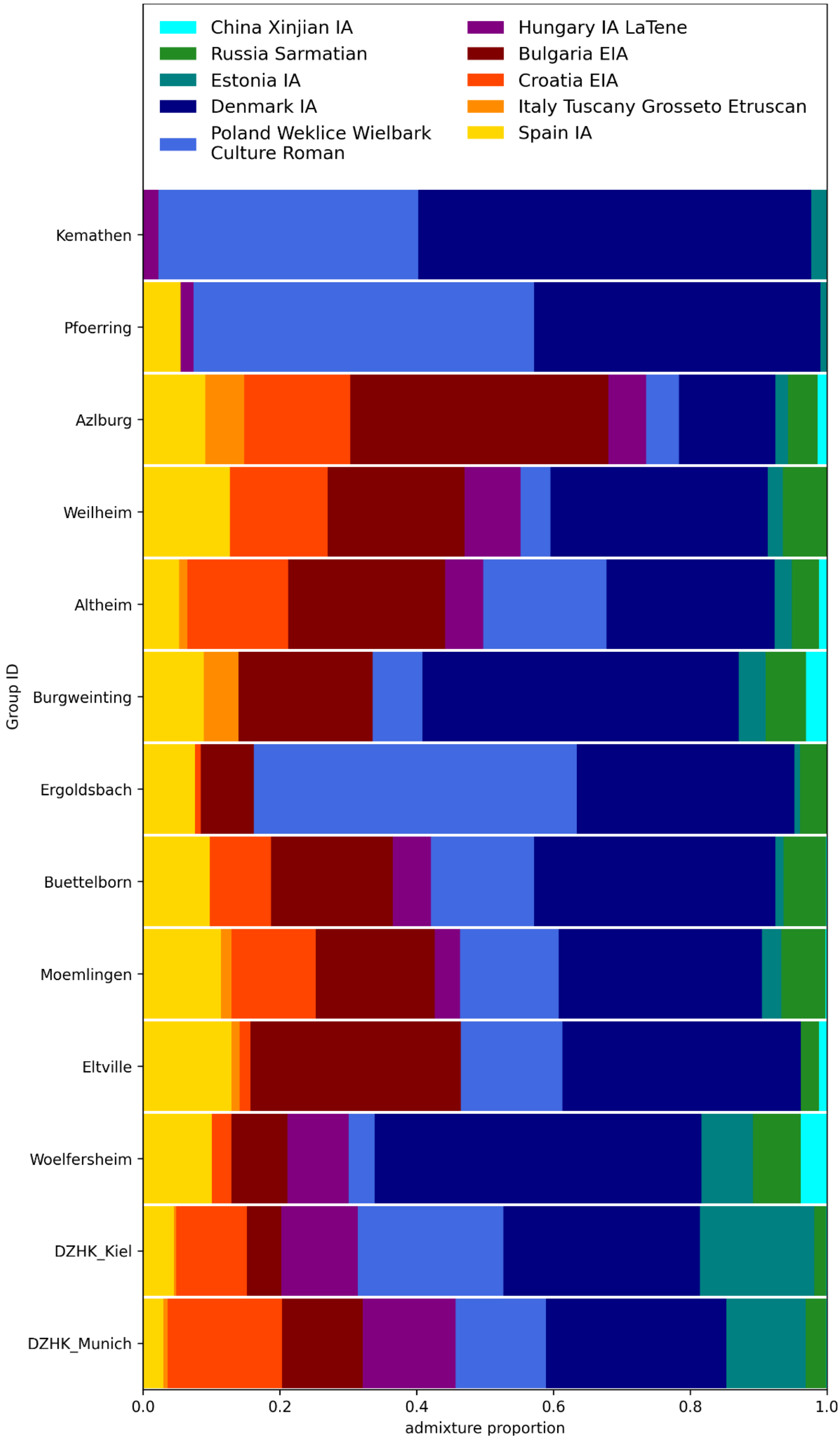

Fig. S8.22: *Results of PANE for a 10 population model. Bars represent average ancestries for the genomes from archaeological sites from Germany, sequenced in this study, spanning Late Antiquity to Early Medieval periods, as well as for the two DZHK cohorts from Munich and Kiel.*

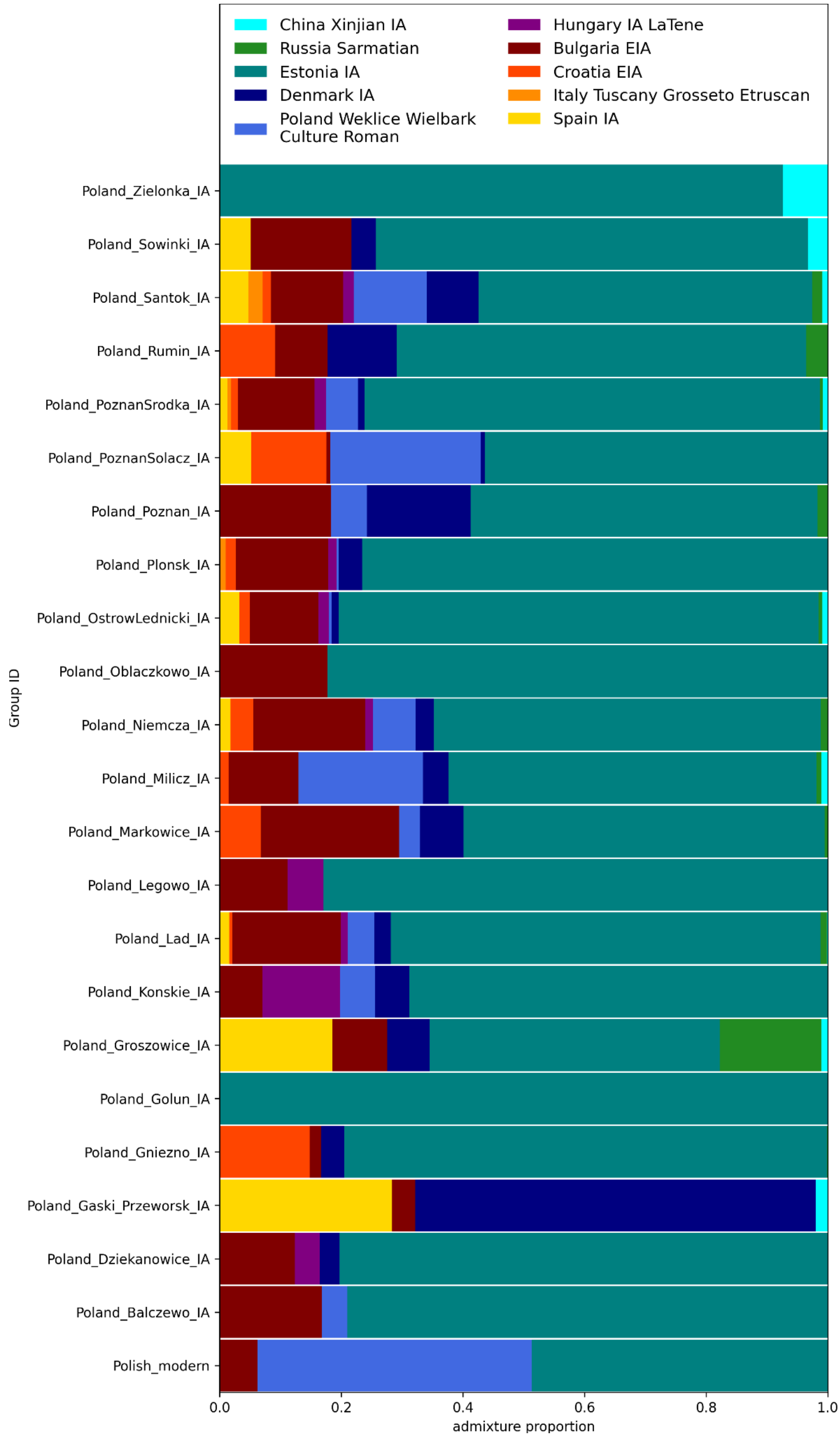

Fig. S8.23: *Results of PANE for a 10 population model. Bars represent average ancestries for genomes from archaeological sites in Poland* [218]*, as well as a modern genome from Poland from the SGDP dataset.*

S9. High genetic diversity in border regions

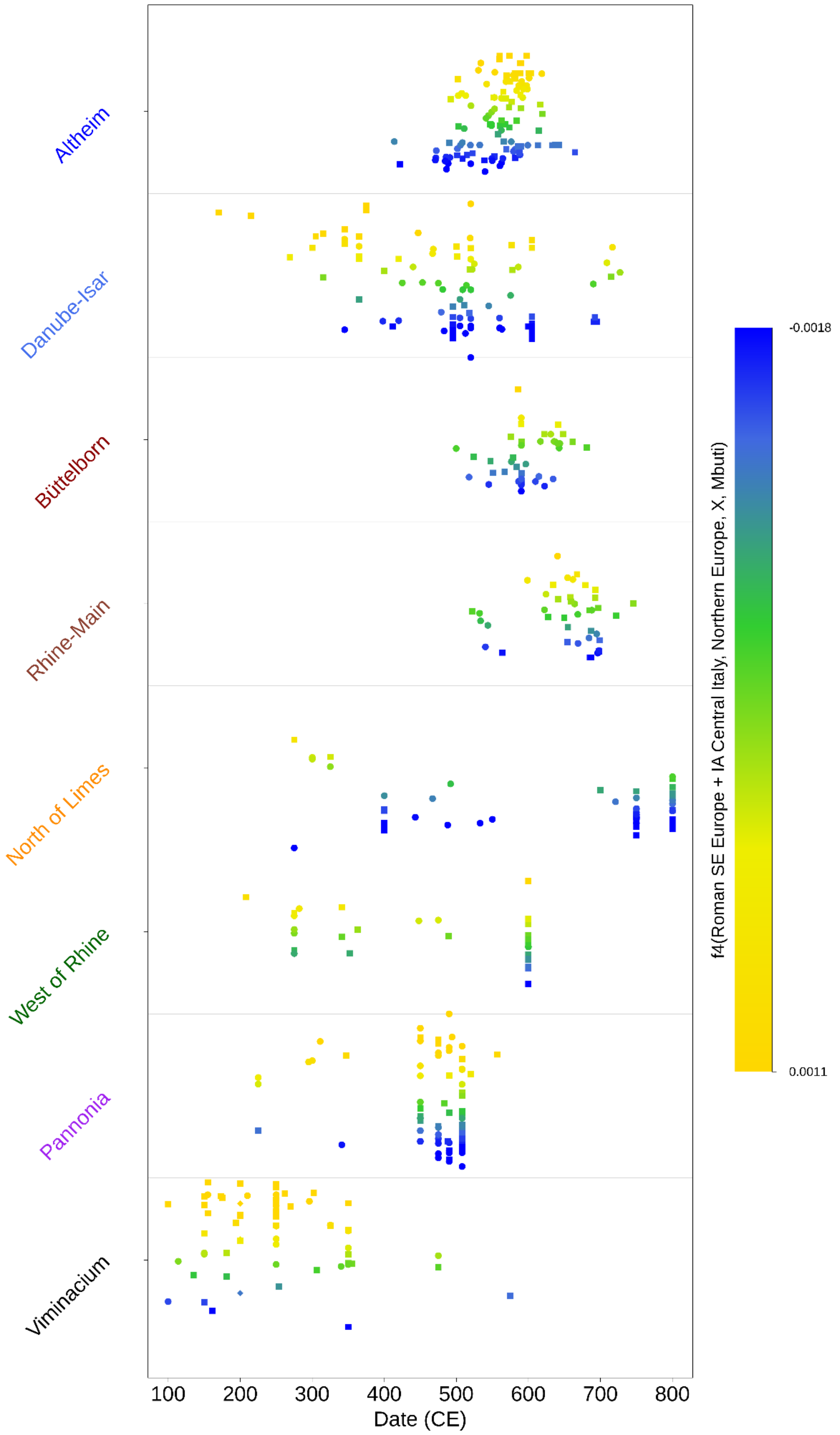

Fig. S9.1: *f_4(Roman Southeastern Europe + Iron Age Central Italy, Northern Europe, X, Mbuti) of Early Medieval and Late Antiquity individuals from former borderlands of the Roman Empire with respect to estimated birth year. The y-axis in each site/region specific subplot is the f_4-statisitics value with positive values at the top and negative ones at the bottom. A wide top to bottom spread is indicative of large genetic diversity. f_4 values of the individuals included in this figure can be found in Supplementary Table 2.11.*

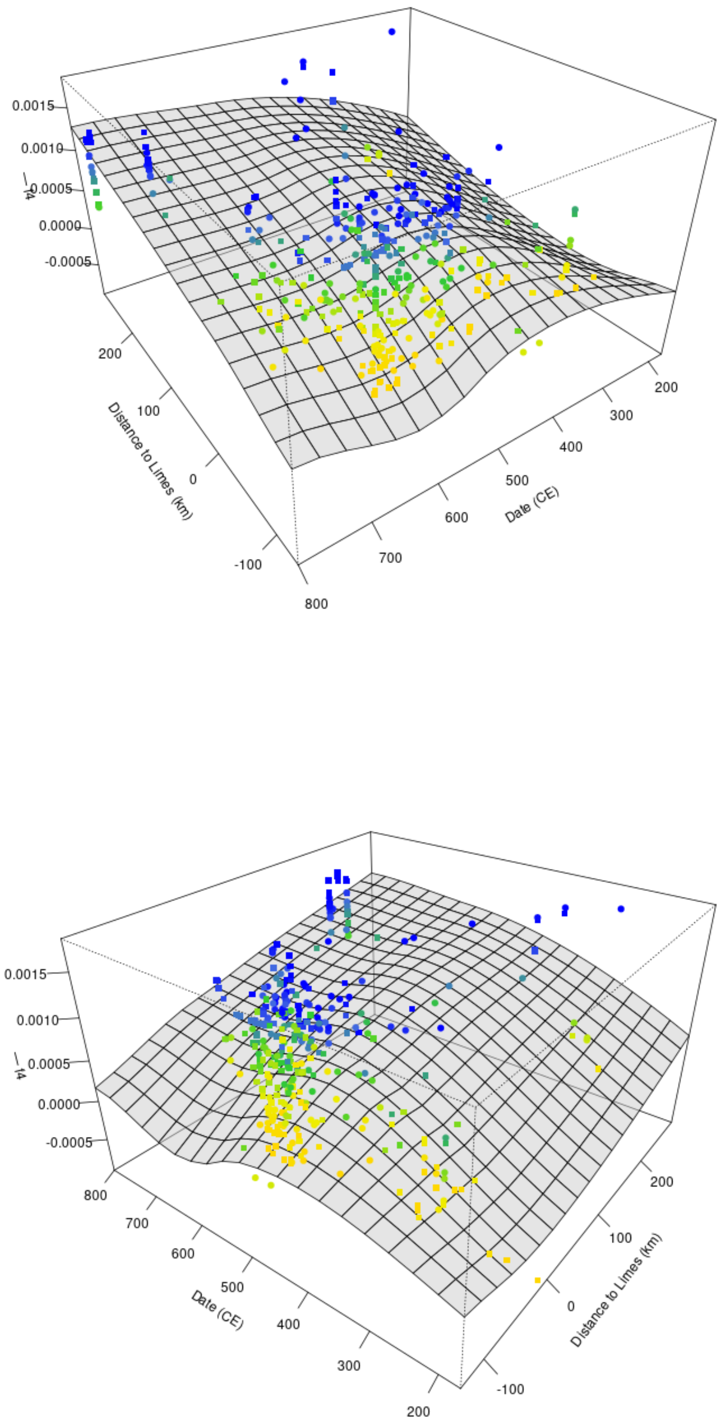

Fig. S9.2: *3D plot showing the f_4-statistics of Late Antiquity and Early Medieval individuals (includes genomes from the regions shown in the previous plot, excluding Pannonia and Viminacium) in relation to chronology and distance from the closest point of the former Roman Limes. A locally weighted regression surface was fitted to visualise the relationship between the three variables. A rotating video version of the plot is available as an electronic supplement (Supplementary Video S2). Color code matches the one of Fig. S9.1.*

S10. Analyses of shared fragments identical by descent (IBD)

We used *ancIBD* [76] to estimate shared identity-by-descent (IBD) segments between our newly sequenced individuals and a set of previously published reference genomes. To ensure temporal comparability, we only included genomes dating within 500 years, either older or younger, of the newly generated samples. Since most published data are based on the 1240K capture array [65] we restricted our analyses to these loci and followed the procedures outlined in the *ancIBD* documentation. Reference genomes were selected to represent western Eurasian populations from the corresponding time periods, where available. For comparison with present-day populations, we additionally estimated IBD sharing patterns in the DZHK cohort samples from Munich and Kiel, using the same loci as for the ancient genomes.

Modern genomes

The Munich cohort ($n = 185$) exhibited a higher degree of IBD sharing (mean: 0.2748 ± 0.0204 cM) compared to the Kiel cohort ($n = 194$, mean: 0.0541 ± 0.0062 cM; Mann–Whitney U test: 161472034, $p < 5.89 \times 10^{-34}$). In Kiel, 89 pairs shared more than 8 cM of IBD, and only 4 pairs exceeded 20 cM. In contrast, Munich had 311 pairs sharing at least 8 cM, of which 41 surpassed 20 cM. Among these, 5 pairs shared more than 50 cM (ranging from 61 to 147 cM), consistent with relatedness of approximately the 5th degree or closer. Between the two cohorts, 58 pairs were identified as sharing a single IBD segment of at least 8 cM, 6 of which extended beyond 12 cM, though none exceeded that threshold.

Ancient genomes: Intra-site comparisons

We first compared intra-site IBDs with the KIN [77] results used for pedigree reconstruction. Both approaches produced highly concordant results, and the additional IBD information helped resolve several uncertain connections where individuals could not previously be placed in pedigrees with confidence. Nonetheless, several 4th- and 5th-degree relationships were inferred that could not be integrated into pedigrees, as missing individuals prevented proper triangulation.The highest average summed pairwise IBDs > 8 cM were observed in Büttelborn (137.49 ± 18.17), followed by Altheim (85.65 ± 4.57) and Weilheim (85.94 ± 47.29), while the lowest values occurred in Mömlingen (24.99 ± 8.84). These results highlight the substantial degree of relatedness present within each site.

We next compared IBD sharing patterns between sex groups at sites with sufficient representation, applying a Mann–Whitney U test (Tables S10.2–S10.4). A significant difference was detected only in Altheim (stat: 1478680; $p < 0.0001$), where XY individuals shared on average 165.94 ± 12.74 cM ($N = 55$) compared to 30.04 ± 5.17 cM in XX individuals ($N = 57$). Moreover, only ~10% of potential female–female pairs showed IBD connections, whereas ~30% of male-male pairs shared at least 8 cM. No significant sex-based differences were observed at the other three sites. This pattern at Altheim may reflect increased female exogamy, though differences in sample size must also be considered: Altheim provided 112 genomes, increasing statistical power and potentially revealing patterns obscured by noise at other sites.

Given the high number of genomes and detailed anthropological data from Altheim, we further subdivided individuals by both sex and age category (adult vs. sub-adult; see Tables S10.4 and S10.5). Using a threshold of 18 years, we identified 36 adult men, 41 adult women, 19 sub-adult men, and 16 sub-adult women. Comparisons involving the "adult women" group consistently yielded significant results, reinforcing the earlier findings. These patterns again suggest female exogamy, with adult women more likely to be buried at the gravesite of their partners rather than alongside parents or siblings. To test the robustness of this signal, we repeated the analyses excluding IBD segments shorter than 12 cM and 16 cM, respectively, but found no changes in significance.

In addition to the total amount of shared IBDs, the number of observed pairs sharing at least one longer segment (>12cM) showed a similar distribution among the sexes. Among all individuals buried in Altheim, 18% of all potential pairs shared one or more IBD segments > 12cM, while 32% of XY/XY pairs were connected by a longer segment, in contrast to only 9% of XX/XX pairs. This pattern was even more pronounced when only individuals estimated to be above the age of 18 years were considered. Here 13% of all potential pairs shared IBD segments longer than 12cM, while 33% of all potential XY/XY shared a segment, while only 3% of adult XX/XX pairs were connected by longer IBDs.

Table S10.1: *Results of Mann-Whitney-U tests comparing the IBD sharing patterns per site between different genetically determined sex groups. In addition to the test results and p-values the mean IBD sharing of all possible male and female pairs is reported incl. the standard error and the total number of individuals in each group. Significant test results are marked in red.*

**Site**	**Stat**	**P**	**Mean XY**	**Error XY**	**N XY**	**Mean XX**	**Error XX**	**N XX**
Büttelborn	17309	0.88	177.74	53.35	17	148.94	31.87	23
Mömlingen	3378	0.62	38.28	19.10	18	10.84	7.47	10
Weilheim	135	0.14	0.00	0.00	6	129.74	100.57	7
Altheim	1478680	0.00	170.55	13.08	55	30.04	5.17	57
Burgweinting	3.5	0.69	0.00	nan	2	308.12	300.94	5
Eltville	18	1.00	0.00	0.00	4	0.00	0.00	4
Straubing-Azlburg	234	1.00	0.00	0.00	13	0.00	0.00	4
Viminacium	50	1.00	0.00	0.00	5	0.00	0.00	5

Table S10.2: *Results of Mann-Whitney-U tests comparing the IBD sharing patterns in Altheim between subgroups of different genetically determined sex groups further divided into adults (a) and sub-adults (sa). In addition to the test results and p-values the mean IBD sharing of all possible male and female pairs is reported incl. the standard error and the total number of individuals in each group. Significant test results are marked in red.*

**Group1**	**Group2**	**Stat**	**P**	**Mean Group1**	**Error Group1**	**N Group1**	**Mean Group2**	**Error Group2**	**N Group2**
XY(a)	XX (a)	335712	0.00	229.32	23.91	36	17.82	6.20	41
XY (sa)	XX (sa)	10369	0.86	57.29	11.37	19	61.40	18.64	16
XY (a)	XY (sa)	55738	0.41	229.32	23.91	36	57.29	11.37	19
XX (a)	XX (sa)	34151	0.00	17.82	6.20	41	61.40	18.64	16
XX(a)	XY (sa)	48388	0.00	17.82	6.20	41	57.29	11.37	19
XY (a)	XX (sa)	39385.5	0.39	229.32	23.91	36	61.40	18.64	16

Table S10.3: *Results of Mann-Whitney-U test comparing summed pairwise IBDs across sites between genetically male and female individuals from Altheim for different age groups. For each group mean summed IBDs with standard deviation are given along the number of observed pairs and the test results. p-values below 0.05 are marked in red.*

**Category: IBD**	**XX Mean IBDs**	**XX Std**	**XX N**	**XY Mean IBDs**	**XY Std**	**XY N**	**STAT**	**P**	**Group**
>8cM	13.50	24.91	620	12.45	10.28	599	198057.5	0.0442	all
>12cM	27.56	49.44	142	20.11	16.60	174	11962	0.6280	
>16cM	42.29	68.41	68	27.12	23.64	72	1991.5	0.0573	
>20cM	60.70	89.79	36	34.59	30.93	37	441	0.0132	
>8cM	13.95	28.67	458	13.01	12.12	388	99776	0.0020	adult
>12cM	30.48	58.67	99	20.16	18.70	132	6351	0.7165	
>16cM	50.94	83.55	44	28.27	27.26	53	923	0.0789	
>20cM	73.29	105.21	25	36.58	34.60	29	246	0.0442	
>8cM	11.43	5.32	211	12.21	7.01	162	16392.5	0.4989	Sub-adult
>12cM	19.96	6.54	42	20.84	9.02	43	910.5	0.9509	
>16cM	23.90	5.51	19	26.45	8.59	24	196.5	0.4484	
>20cM	27.36	4.36	8	32.09	7.40	11	24	0.1087	

Table S10.4: *Results of Mann-Whitney-U test comparing pairwise distances in km between pairs of individuals from Altheim and other archaeological sites connected through shared IBDs, sub-divided into genetically male and female for different age groups. For each group the mean distance [km] with standard deviation are given along the number of observed pairs and the test results.*

**Category: IBD**	**XX Mean Dist [km]**	**XX Std**	**XX N**	**XY Mean Dist [km]**	**XY Std**	**XY N**	**STAT**	**P**	**Group**
>8cM	556.48	373.07	620	521.35	363.94	599	174989	0.0805	all
>12cM	379.23	307.77	142	435.82	371.91	174	13107	0.3485	
>16cM	311.33	310.05	68	365.91	289.54	72	2777	0.1655	
>20cM	291.30	322.45	36	287.27	190.57	37	727	0.4978	
>8cM	583.04	372.19	458	535.04	367.54	388	81044.5	0.0270	adult
>12cM	387.85	304.25	99	466.42	386.41	132	7106	0.2522	
>16cM	302.59	313.08	44	398.75	311.29	53	1400.5	0.0862	
>20cM	311.09	337.48	25	290.96	196.04	29	388	0.6596	

>8cM	481.37	365.23	162	496.17	355.85	211	17780.5	0.5028	Sub-adult
>12cM	359.39	314.83	43	339.63	302.60	42	878.5	0.8316	
>16cM	327.36	303.76	24	274.29	189.64	19	220.5	0.8619	
>20cM	246.34	280.22	11	273.88	168.57	8	48.5	0.7365	

Table S10.5: *Results of Mann-Whitney-U test comparing summed pairwise IBDs across sites between genetically male and female individuals from our Early Medieval genomes. For each group mean summed IBDs with standard deviation are given along the number of observed pairs and the test results. p-values below 0.05 are marked in red.*

**Category: IBD**	**XX Mean IBDs**	**XX Std**	**XX N**	**XY Mean IBDs**	**XY Std**	**XY N**	**STAT**	**P**
>8cM	12.74	0.61	1112	12.87	0.62	1048	618516.5	0.0134
>12cM	24.60	2.56	250	23.09	2.34	267	33813	0.7966
>16cM	40.09	6.10	100	35.35	5.62	107	4778	0.1845
>20cM	56.74	10.80	54	49.78	10.58	55	1154.5	0.0455

Table S10.6: *Results of Mann-Whitney-U test comparing pairwise distances in km between pairs of individuals from our Early Medieval genomes and other archaeological sites connected through IBDs, sub-divided into genetically male and female. For each group the mean distance [km] with standard deviation are given along the number of observed pairs and the test results.*

**Category: IBD**	**XX Mean Dist [km]**	**XX Std**	**XX N**	**XY Mean Dist [km]**	**XY Std**	**XY N**	**STAT**	**P**
>8cM	576.31	11.05	1112	552.75	11.41	1048	555485	0.0602
>12cM	416.59	19.80	250	440.23	22.81	267	32992	0.8213
>16cM	336.83	31.41	100	354.87	30.26	107	5467	0.7851
>20cM	340.80	45.07	54	302.93	37.46	55	1368	0.4762

Ancient genomes: Inter-site comparisons

We used *ancIBD* to identify shared IBD segments between newly sequenced Early Medieval individuals and published contemporaneous genomes within a ±500-year window (Fig. S10.4). Across all sites, shorter IBD segments were widely shared throughout Europe, whereas longer segments (>16 cM) were predominantly shared with Northern European genomes, many associated with populations historically described as "Saxon" in Germany, the Netherlands, and the UK.[16,17]. These results parallel the PCA and f4-statistics, with several new genomes showing strong affinities to Northern European ancestry.

Comparisons of segments >20 cM revealed regional differences: individuals from the Rhine-Main region shared more IBD with genomes from northern Germany and the UK than those from the Danube-Isar region, where connections were instead concentrated further east in Austria [18,37], Hungary [19] and Serbia [14]. At Altheim, the strongest links were with two Austrian sites, Mödling An der Goldenen Stiege and Csokorgasse, cemeteries of Avar context dating from the mid-7th to late 9th century. Despite their context, individuals from these cemeteries lacked the Eastern Eurasian ancestry typical of Avar burials (e.g. nearby Leobersdorf [37]). Fourteen Altheim individuals were connected to six from Mödling, with four of the latter also related to each other, sharing 21.36–52.32 cM. Eleven Altheim individuals shared IBDs of 21.88–81.23 cM with four unrelated individuals from Csokorgasse. By contrast, the Altheim individual with predominantly East Asian ancestry (Alh_245) did not share long IBDs with Avar-associated genomes, but instead showed >20 cM connections to individuals from the Berel necropolis in present-day Kazakhstan (Supplementary Table 3). IBDs of 50–100 cM, typical of 5th-degree relatives (Fig. S12.1), suggest that common ancestors of some Altheim individuals lived locally before descendants moved eastwards.

Sex-stratified analyses at Altheim revealed no significant differences in geographic distances of pairwise IBDs, except when adult females were compared directly to adult males, where females showed slightly longer-distance connections (Table S10.4). Altheim women also shared more large IBD segments across sites than men (>8 cM and >20 cM; Table S10.3, Fig. S10.8). These patterns remained significant when restricting to adults, but not among sub-adults, and extended to the rest of the Early Medieval genomes (Table S10.5, Fig. S10.11), suggesting a wider trend of female exogamy. Assuming individuals sharing <50 cM were unrelated, 20 Altheim individuals fell into this category (16 female, 6 male). Of these, 17 were adults (12 XX, 5 XY) and three were children under ~10 years. Only five adults shared >20 cM across sites; one (Alh_161) showed close relatedness to three individuals from nearby Ergoldsbach (Supplementary Table 3).

Finally, to assess IBD sharing in a broader population-genetic framework, we examined inter-site connections relative to positions on the first two principal components. Using 20 evenly spaced bins along PC1 and PC2 covering 95% of the dataset (excluding Avar-context genomes with Eastern Eurasian ancestry; Figs. S10.14–S10.15), we measured IBD frequency as the proportion of pairs sharing >8 cM. Spearman correlations revealed strong negative associations with both PC1 ($r = -0.896$, $p < 9.0 \times 10^{-3}$) and PC2 ($r = -0.853$, $p < 1.8 \times 10^{-3}$; Figs. S10.12–S10.13).

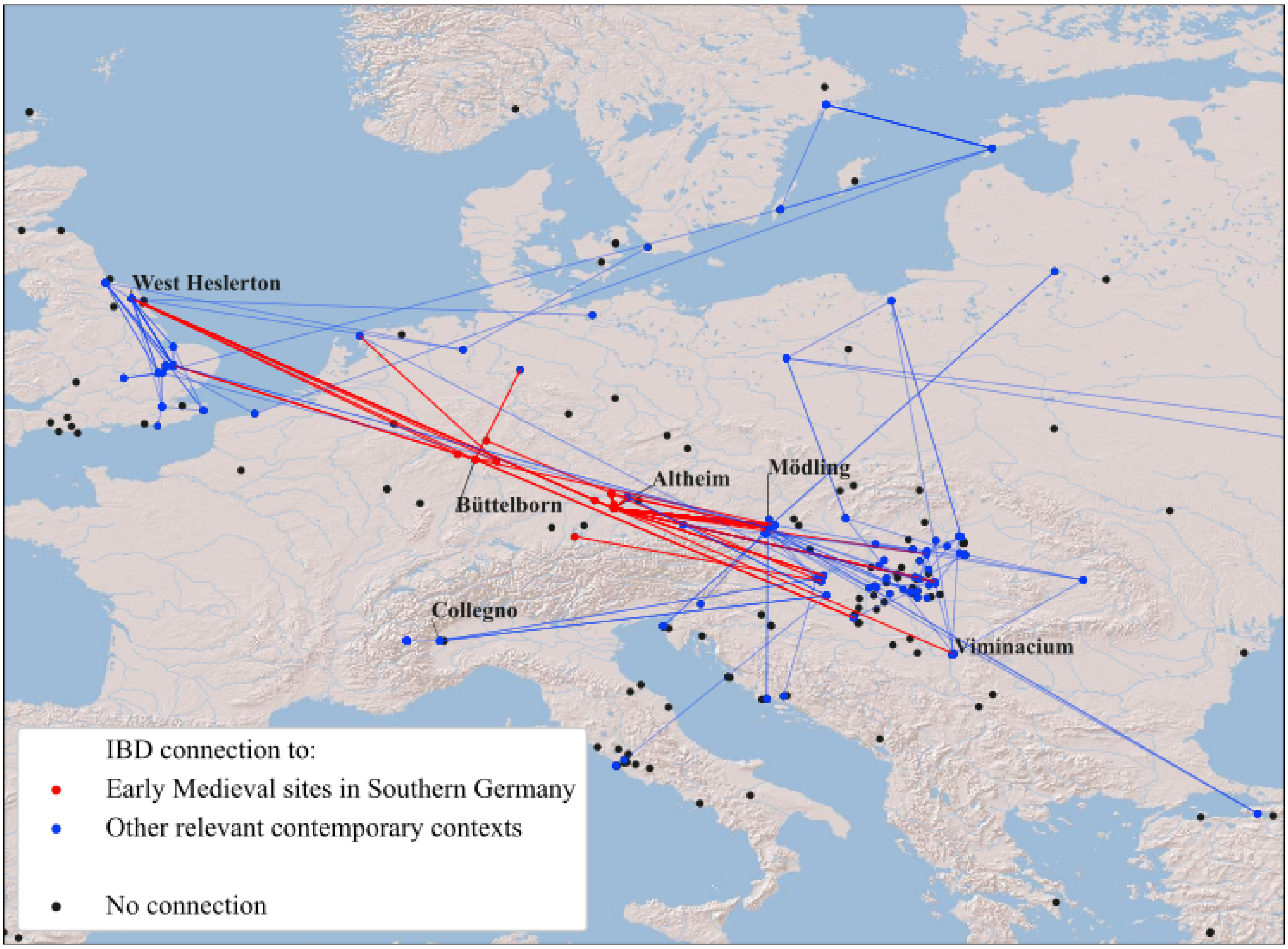

Fig. S10.1: *Map showing pairwise shared fragments identical by descent (IBD) between individuals from Antiquity and the Early Middle Ages (1–800 CE). Red lines indicate pairs from Late Antique and Early Medieval sites in southern Germany sharing at least one genomic segment of 20 cM or longer; light blue lines mark equivalent links between other relevant contemporary contexts. The vast majority of IBDs are shared between individuals with a northern European ancestry profile. The sites with the highest number of individuals in each area are indicated by name.*

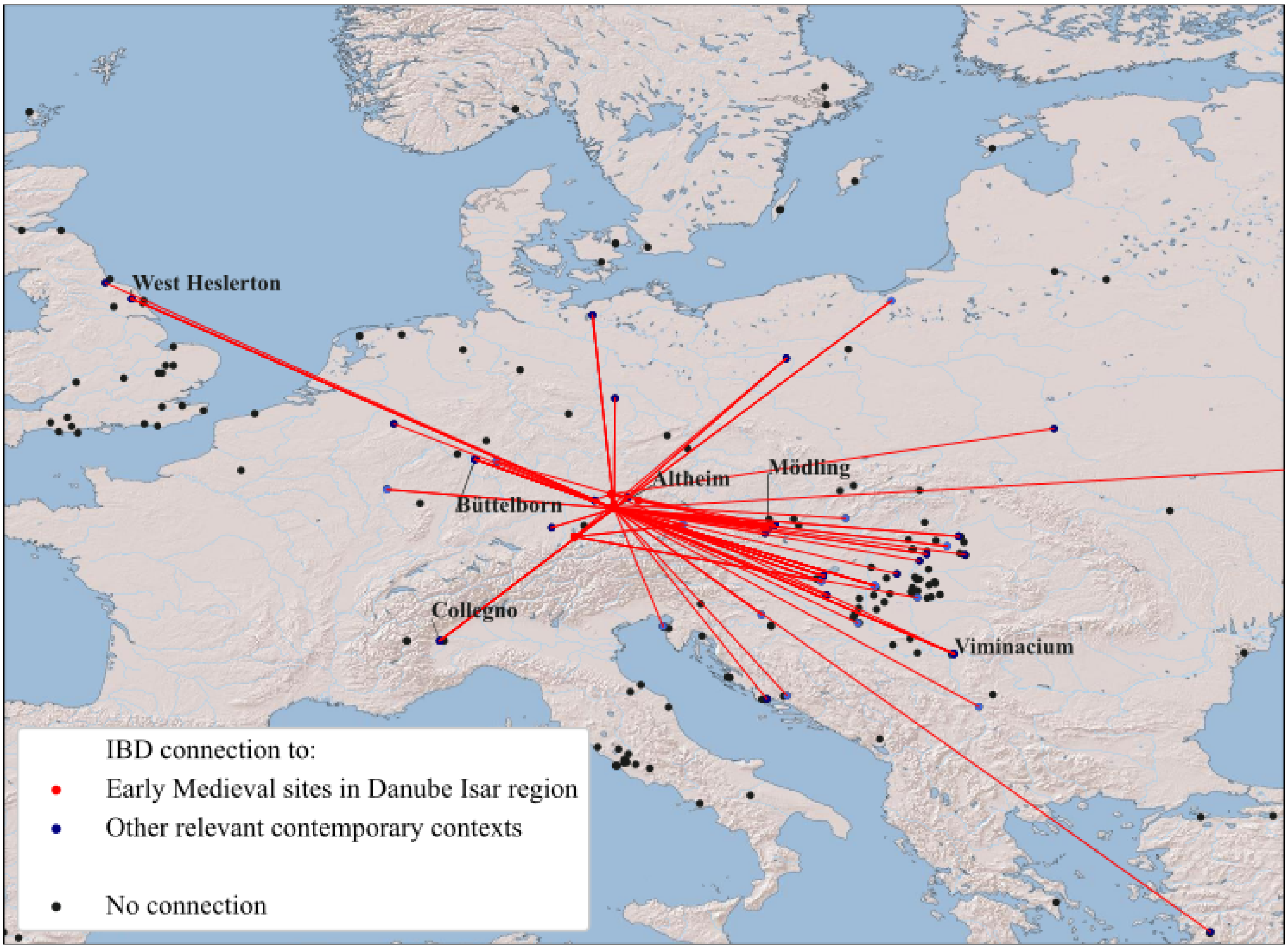

Fig. S10.2: *Shared Fragments Identical by Descent (IBD) >12 cM between the newly sequenced individuals from the Danube-Isar region. Individuals at each archaeological site are marked as dots, in case of IBD sharing they are colored blue and connected via a red line to individuals from other sites.*

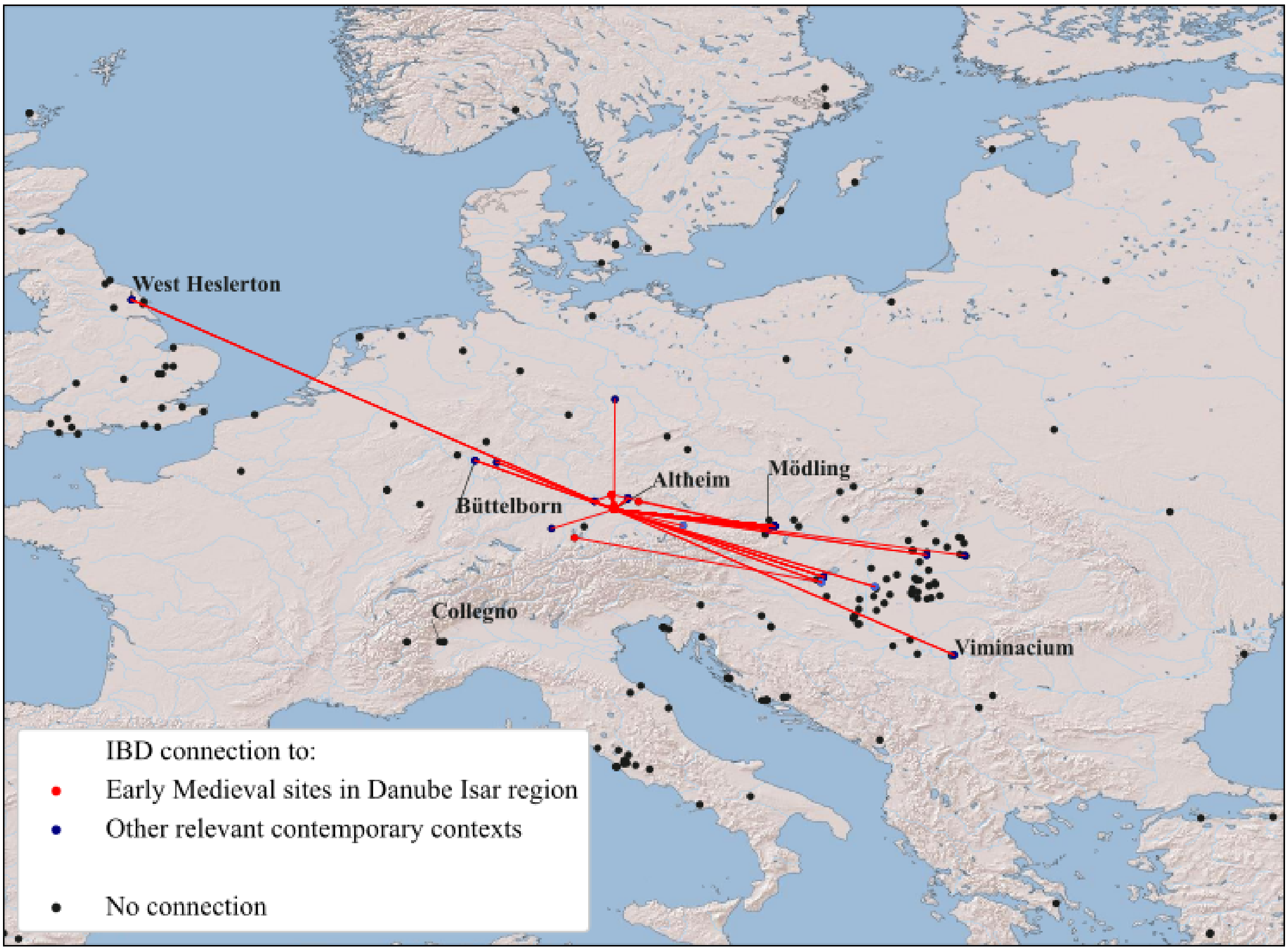

Fig. S10.3: *Shared fragments identical by descent (IBD) >16 cM between the newly sequenced individuals from the Danube-Isar region. Individuals at each archaeological site are marked as dots, in case of IBD sharing they are colored blue and connected via a red line to individuals from other sites.*

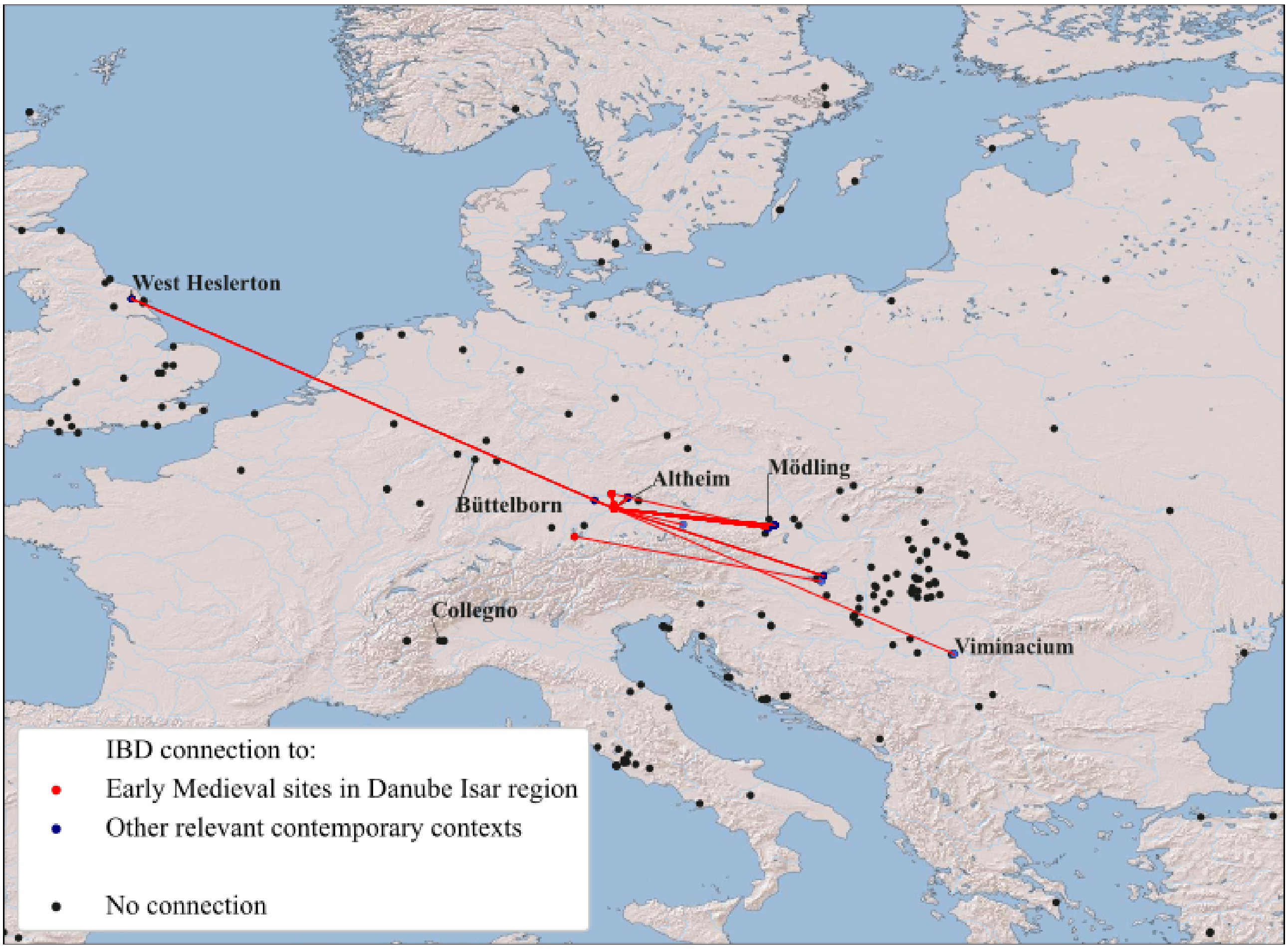

Fig. S10.4: *Shared fragments identical by descent (IBD) >20 cM between the newly sequenced individuals from the Danube-Isar region. Individuals at each archaeological site are marked as dots, in case of IBD sharing they are colored blue and connected via a red line to individuals from other sites.*

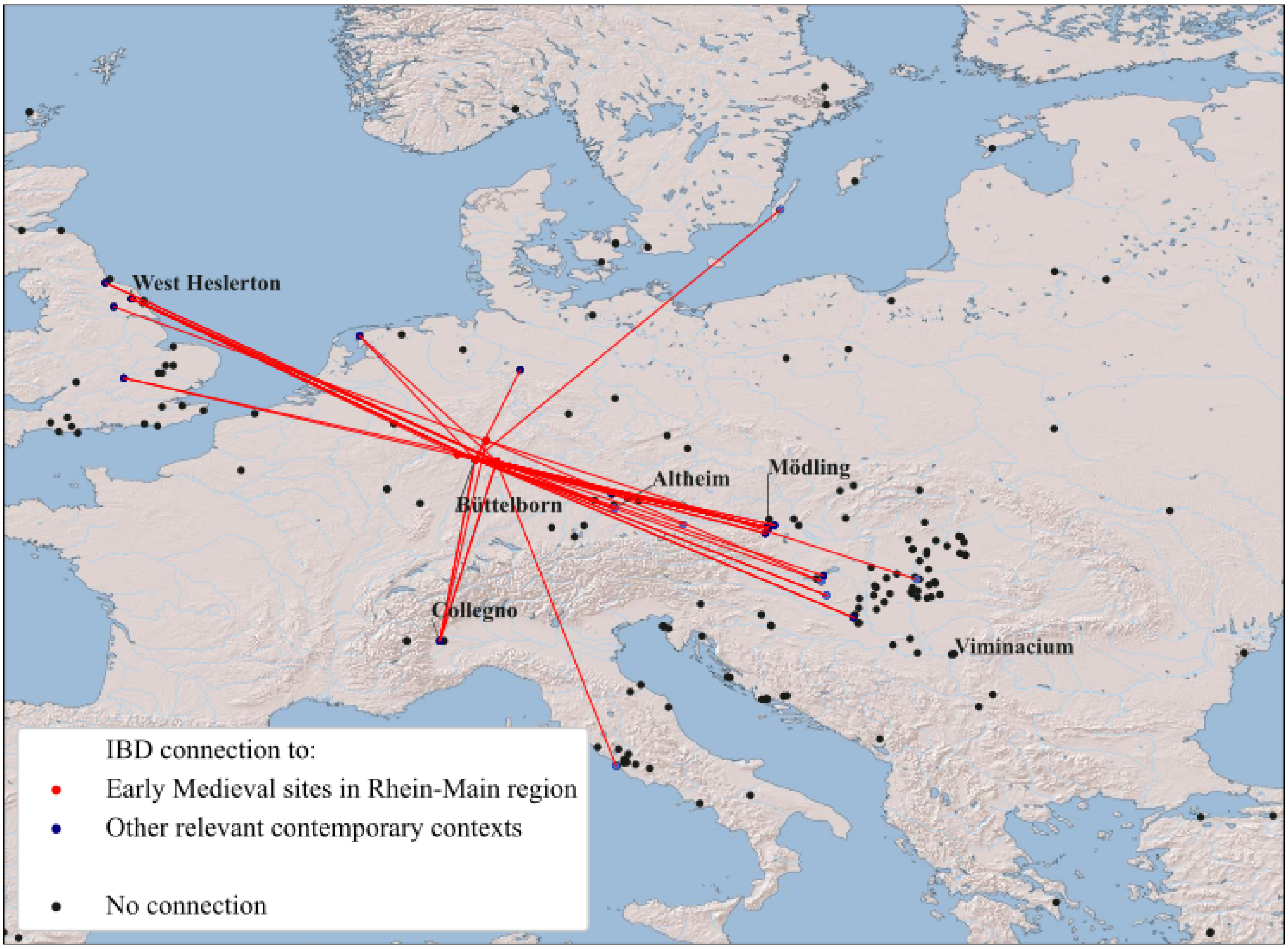

Fig. S10.5: *Shared fragments identical by descent (IBD) >12 cM between the newly sequenced individuals from the Rhine-Main region. Individuals at each archaeological site are marked as dots, in case of IBD sharing they are colored blue and connected via a red line to individuals from other sites.*

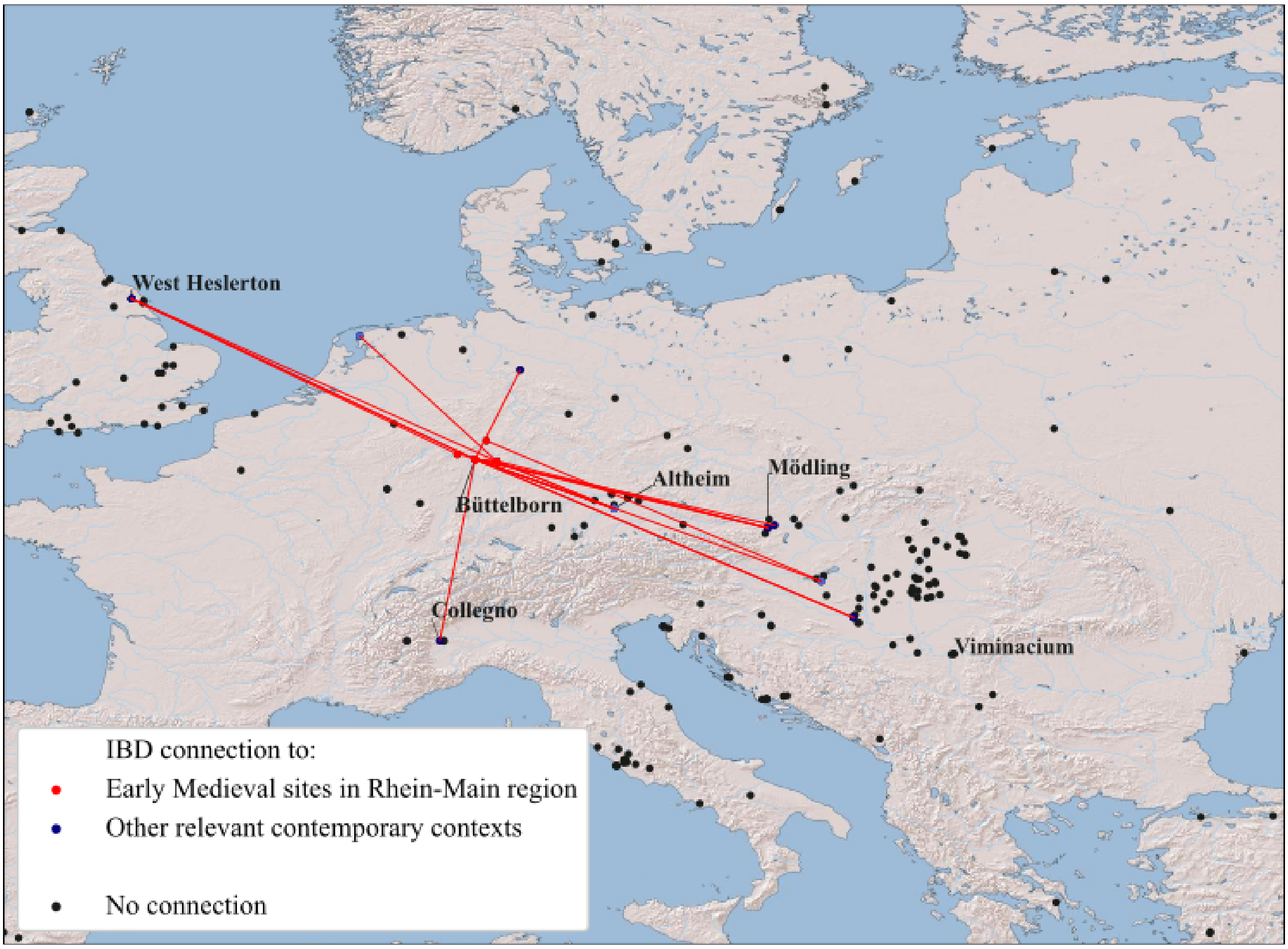

Fig. S10.6: *Shared fragments identical by descent (IBD) >16 cM between the newly sequenced individuals from the Rhine-Main region. Individuals at each archaeological site are marked as dots, in case of IBD sharing they are colored blue and connected via a red line to individuals from other sites.*

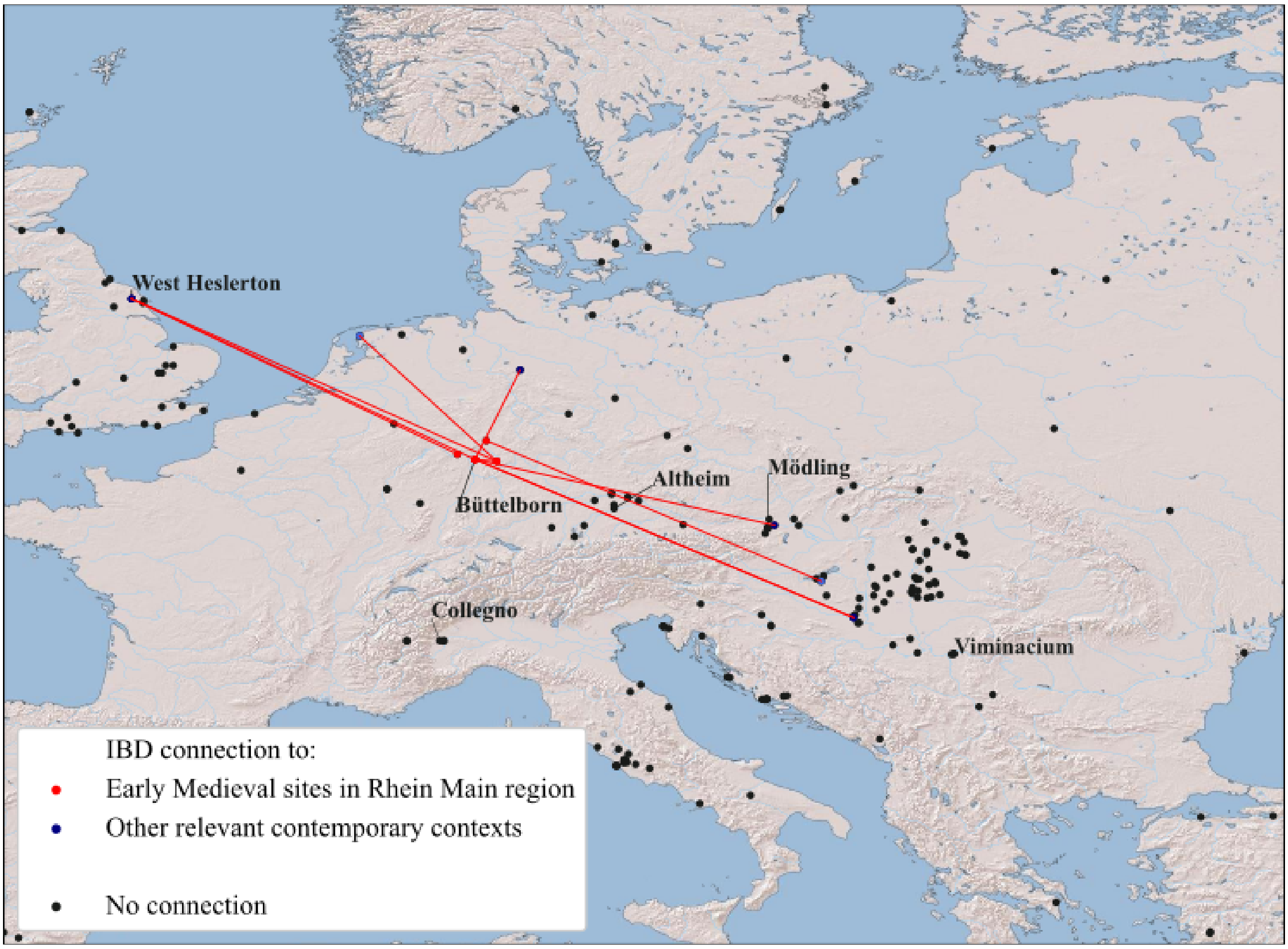

Fig. S10.7: *Shared fragments identical by descent (IBD)IBD sharing of segments >20 cM between the newly sequenced individuals from the Rhine-Main region. Individuals at each archaeological site are marked as dots, in case of IBD sharing they are colored blue and connected via a red line to individuals from other sites.*

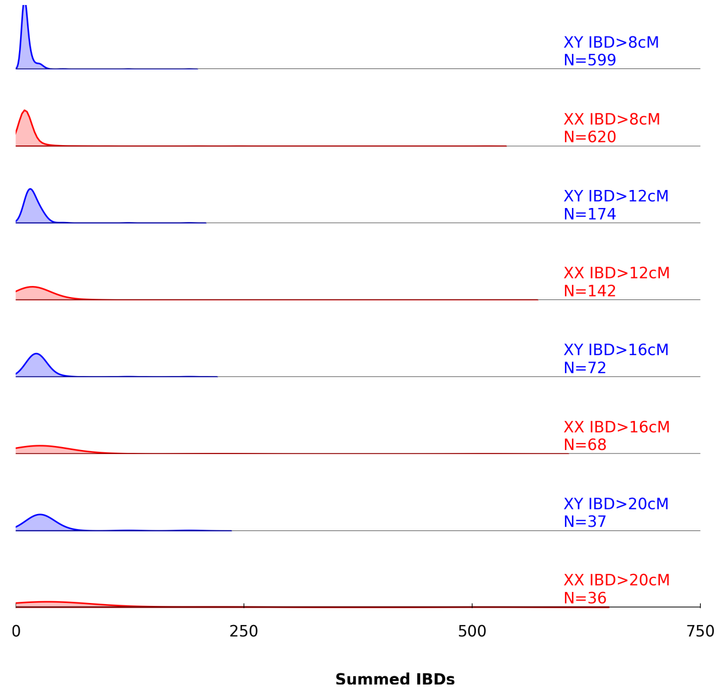

Fig. S10.8: *KDE-plots showing summed pairwise IBDs to individuals at sites outside of Altheim by sex (blue = male, red = female) at thresholds from IBDs > 8cM to IBDs > 20cM.*

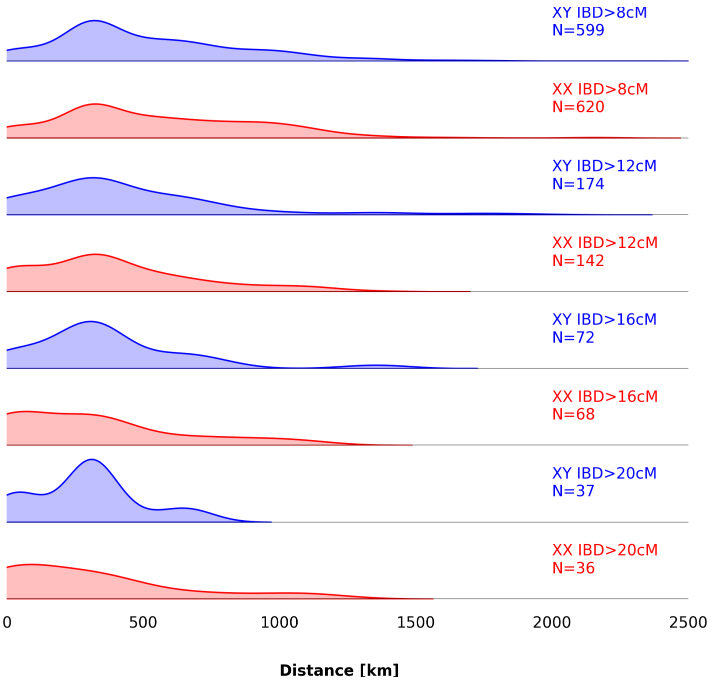

Fig. S10.9: *KDE-plots showing pairwise distances in km for Altheim individuals connected by IBDs to individuals found at other archaeological sites at different thresholds by sex (blue = male, red = female) at thresholds from IBDs > 8cM to IBDs > 20cM.*

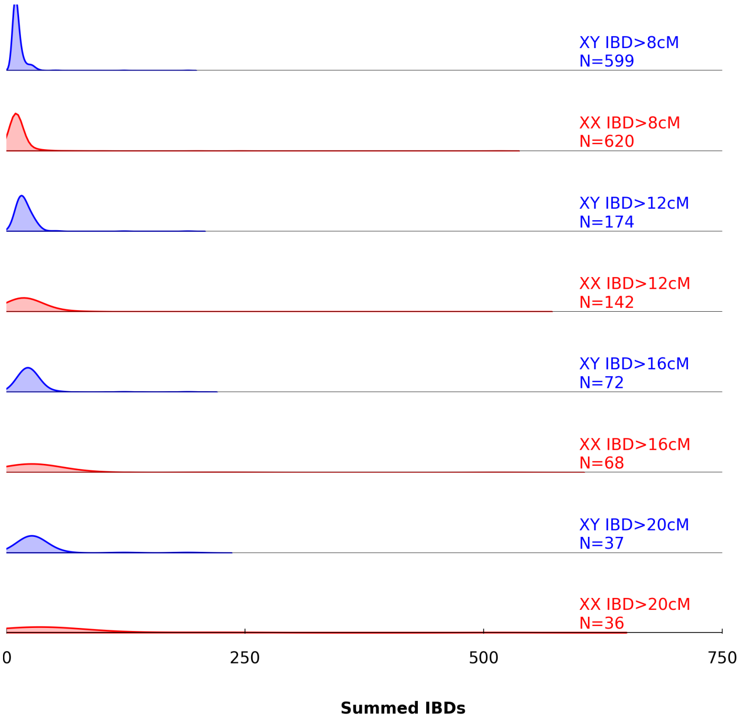

Fig. S10.10: *KDE-plots showing summed pairwise IBDs to individuals at sites outside of their own by sex (blue = male, red = female) for all Early Medieval genomes from the Rhine-Main and Danube-Isar region, at thresholds from IBDs > 8cM to IBDs > 20cM.*

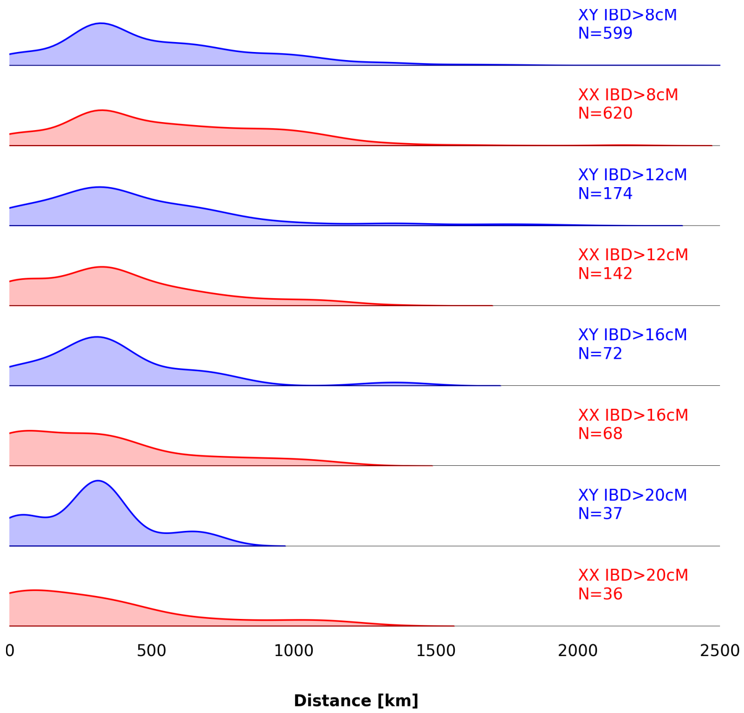

Fig. S10.11: *KDE-plots showing pairwise distances in km for Early Medieval individuals connected by IBDs to individuals found at other archaeological sites at different thresholds by sex (blue = male, red = female) at thresholds from IBDs > 8cM to IBDs > 20cM.*

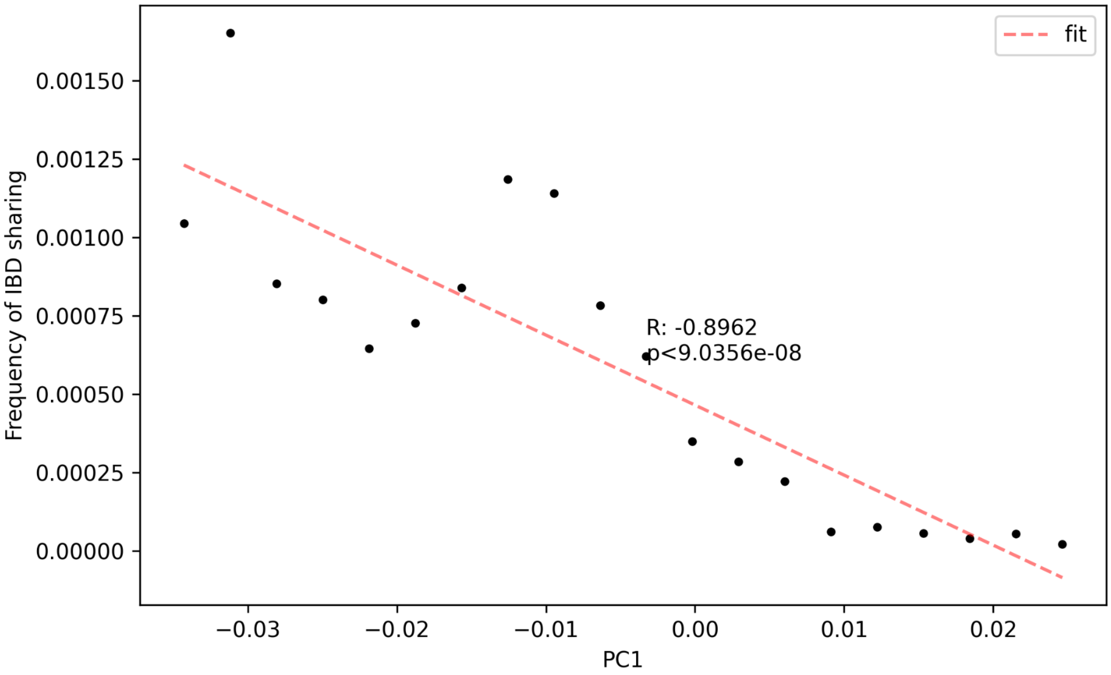

Fig. S10.12: *Frequencies of IBD sharing between Early Medieval individuals from Central Europe measured in 20 even sized bins alongside PC1, incl. best-fit trendline and Spearman's R with p-value.*

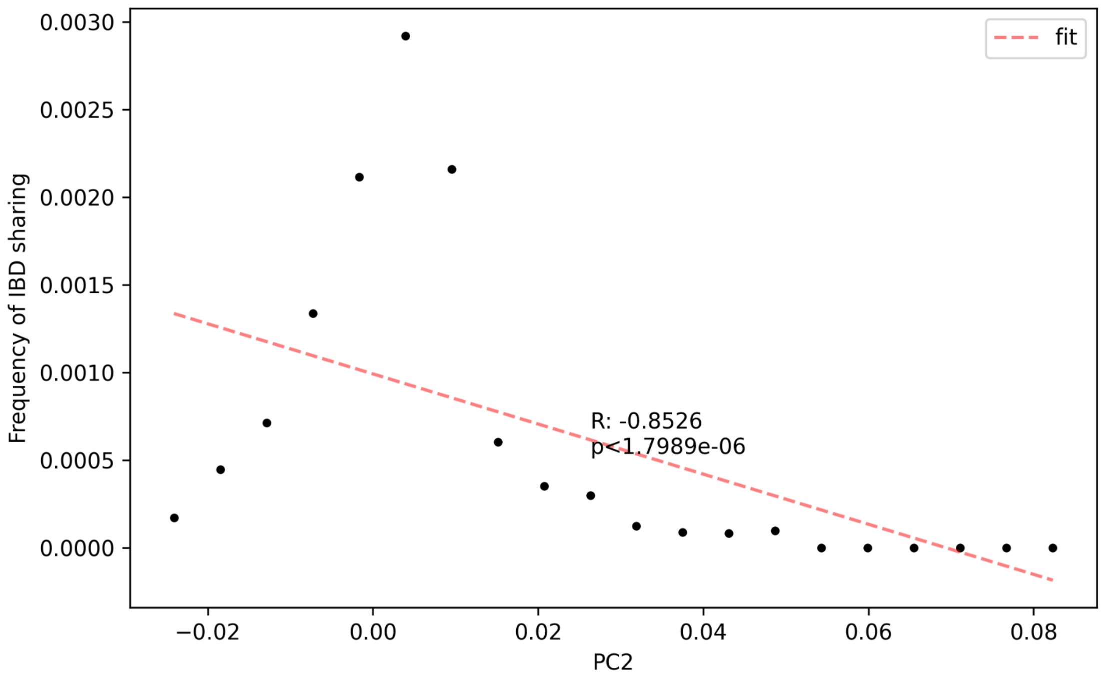

Fig. S10.13: *Frequencies of IBD sharing between Early Medieval individuals from Central Europe measured in 20 equallysized bins alongside PC2, incl. best-fit trendline and Spearman's R with associated p-value.*

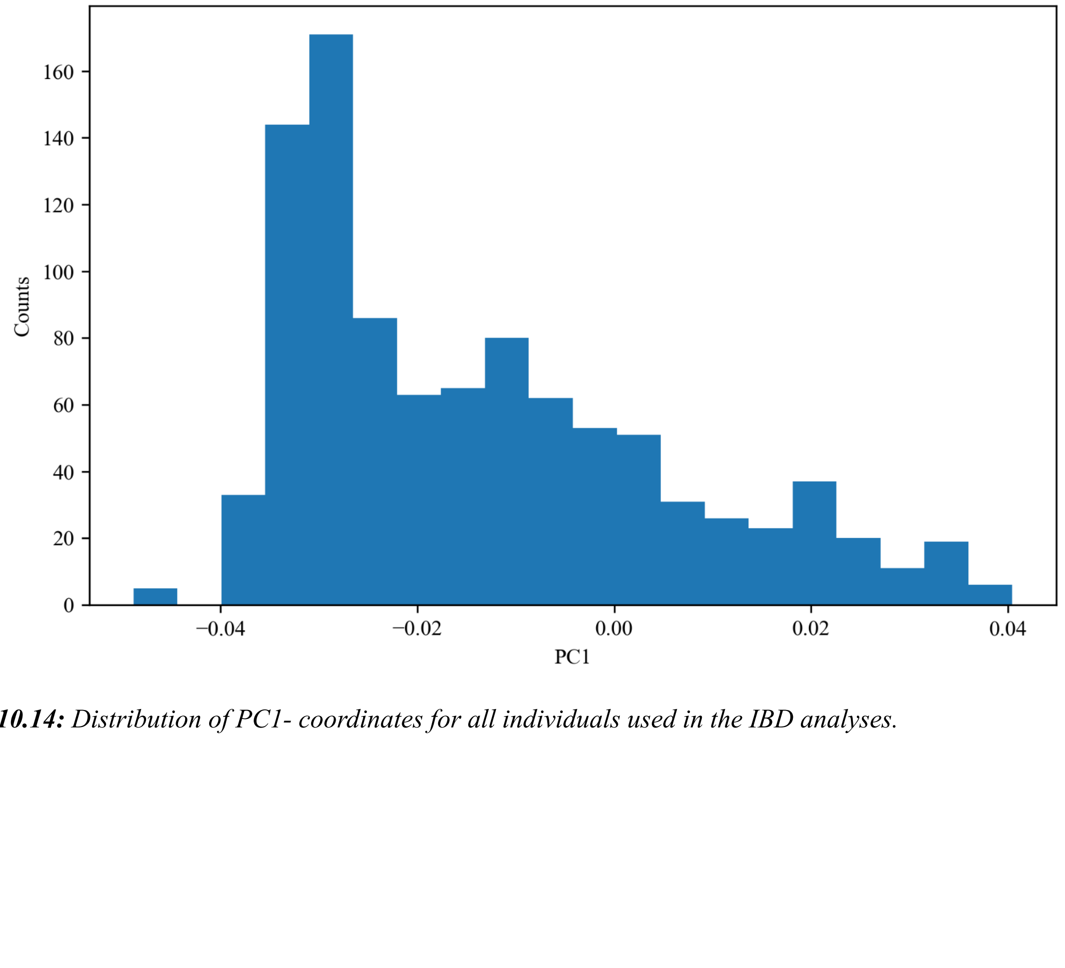

Fig. S10.14: *Distribution of PC1- coordinates for all individuals used in the IBD analyses.*

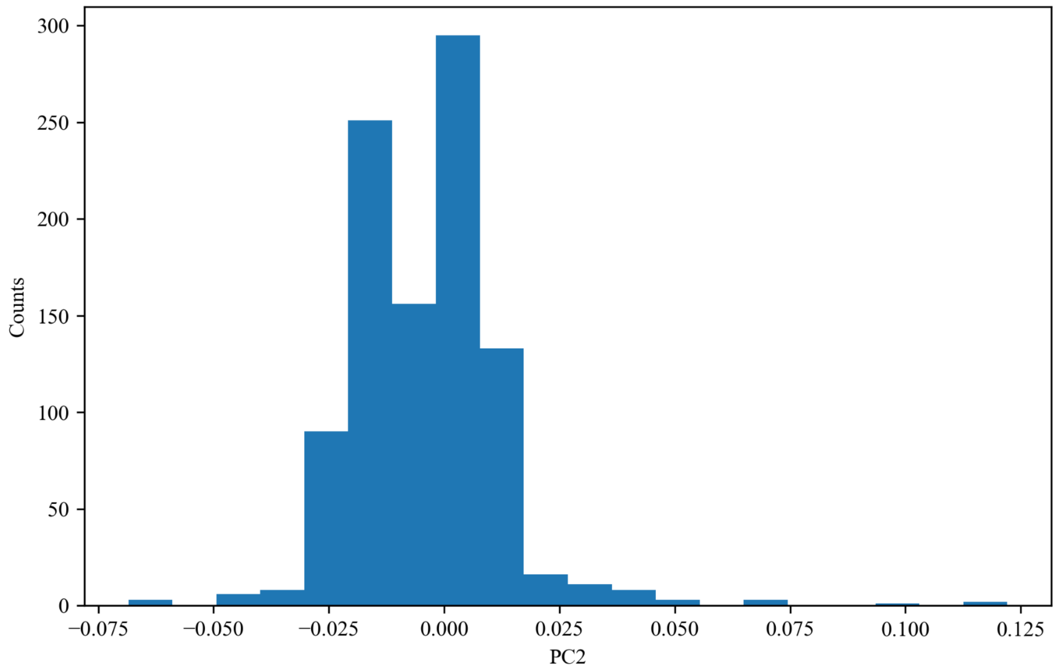

Fig. S10.15: *Distribution of PC2- coordinates for all individuals used in the IBD analyses.*

S11. Runs of homozygosity

To test for potential inbreeding we used *hapROH* [75] to estimate runs of homozygosity (ROHs) larger than 4cM. Majority allele calls produced by *ATLAS* [60] for individuals with more than 400K SNPs overlapping the 1240K capture array [65] were converted to binary plink-format [219] and subsequently to packed eigenstrat using admixtools [220]. *hapROH* was run with default settings.

For only a small fraction of all individuals ROHs larger than 4cM could be determined (Table S11.1). This is consistent with the overall high degree of genomic diversity found at the different archaeological sites. Only eight individuals showed signs of ROHs > 12cM, while the total number can still be considered as low and no sign of recent consanguinity. Interestingly all 8 individuals originated from sites in Bavaria or further to the South.

Table S11.1: *hapROH results for all newly sequenced individuals for which ROHs > 4cM were detected*

ID	max	sum>4	n>4	sum>8	n >8	sum>12	n >12	sum>20	n >20
Alh_280	22.38	85.02	6	77.13	5	58.09	3	22.38	1
Alh_248	16.86	49.73	7	16.86	1	16.86	1	0	0
Alh_230	10.04	31.15	4	18.38	2	0	0	0	0
Alh_163	21.58	21.58	1	21.58	1	21.58	1	21.58	1
Alh_152	21.58	21.58	1	21.58	1	21.58	1	21.58	1
Alh_279	8.93	14.34	2	8.93	1	0	0	0	0
Alh_243	6.73	6.73	1	0	0	0	0	0	0
Alh_356	5.97	5.97	1	0	0	0	0	0	0
Alh_144	5.95	5.95	1	0	0	0	0	0	0
Alh_129	5.55	5.55	1	0	0	0	0	0	0
Alh_249	5.19	5.19	1	0	0	0	0	0	0
Alh_281	5.14	5.14	1	0	0	0	0	0	0
Alh_131	4.97	4.97	1	0	0	0	0	0	0
Alh_61	4.82	4.82	1	0	0	0	0	0	0
Alh_136	4.68	4.68	1	0	0	0	0	0	0
Alh_72	4.56	4.56	1	0	0	0	0	0	0
Alh_231	4.05	4.05	1	0	0	0	0	0	0

Arg1	22.95	22.95	1	22.95	1	22.95	1	22.95	1
Btb72_1	5.84	5.84	1	0	0	0	0	0	0
Btb68	5.10	5.10	1	0	0	0	0	0	0
Btb100	4.43	4.43	1	0	0	0	0	0	0
Btb161_1	4.42	4.42	1	0	0	0	0	0	0
Btb46	4.37	4.37	1	0	0	0	0	0	0
Btb104	4.03	4.03	1	0	0	0	0	0	0
Dol1	10.55	16.51	2	10.55	1	0	0	0	0
ErgDF1	5.11	5.11	1	0	0	0	0	0	0
FN2	25.92	94.71	10	68.85	5	39.30	2	25.92	1
Mat1	4.18	4.18	1	0	0	0	0	0	0
Mln28a	8.47	8.47	1	8.47	1	0	0	0	0
Mln35	5.02	5.02	1	0	0	0	0	0	0
Molz3	203.99	208.28	2	203.99	1	203.99	1	203.99	1
Molz1	11.73	21.67	2	21.67	2	0	0	0	0
STR486	6.39	6.39	1	0	0	0	0	0	0
STRAZ_II_13	14.40	20.90	2	14.40	1	14.40	1	0	0
VIM5	7.51	7.51	1	0	0	0	0	0	0
Wh6	4.88	9.07	2	0	0	0	0	0	0
Zsch1	6.41	11.84	2	0	0	0	0	0	0

S12. Genetic relatedness estimation and Pedigree reconstruction

Estimating biological relatedness with *KIN* and *READ2*

Genetic relatedness among the newly sequenced Early Medieval individuals in this study was inferred using *KIN* [77] and further validated with *READ2* [78] and IBD estimates obtained via *ancIBD* [76]. Detailed results are provided in Supplementary Table 2.14-15.

Pedigree reconstructions

Following Blöcher *et al.* (2023) [79], we combined *KIN*-based relatedness estimates with genetic sex, uniparental haplogroup information, and anthropological age predictions to reconstruct potential pedigrees for the individuals buried at the different sites. We compared the pairwise IBD sharing patterns with the *KIN* results to confirm estimated relationships and their degree. For that we analyzed to summed length of all IBDs in relation to the length of the genome, as well as the total number in regard to the summed length of IBDs >8cM to see if they clustered with other pairs for which the same degree or class of relatedness was inferred (Fig. S12.1). We further tested if individuals that were connected through others in the pedigree shared IBDs on a scale that was on par with the inferred connections. Through this triangulation process the certainty could be increased and allowed for the reconstruction of several pedigrees of varying sizes. We used an iterative approach, starting by connecting all first degree relatives and adding more distant connections in the following steps, if those relationships were not already satisfied by previously drawn connections. To connect sampled individuals up to 3rd degree, we included the minimal number of unsampled individuals necessary to reflect these relationships in the pedigree, if their relationship could be resolved. We did so under the assumption that these inferred by unsampled individuals were likely part of the community (such as unsampled parents of sampled siblings). If the exact relationship could not be inferred, or if individuals were more distantly related, we only indicated that connection but did not include unsampled individuals in the pedigree. We refrained from connecting individuals to pedigrees that could not be placed with high enough certainty (3rd/4th or 5th-degree) or if those connections could not be verified by more than one method.

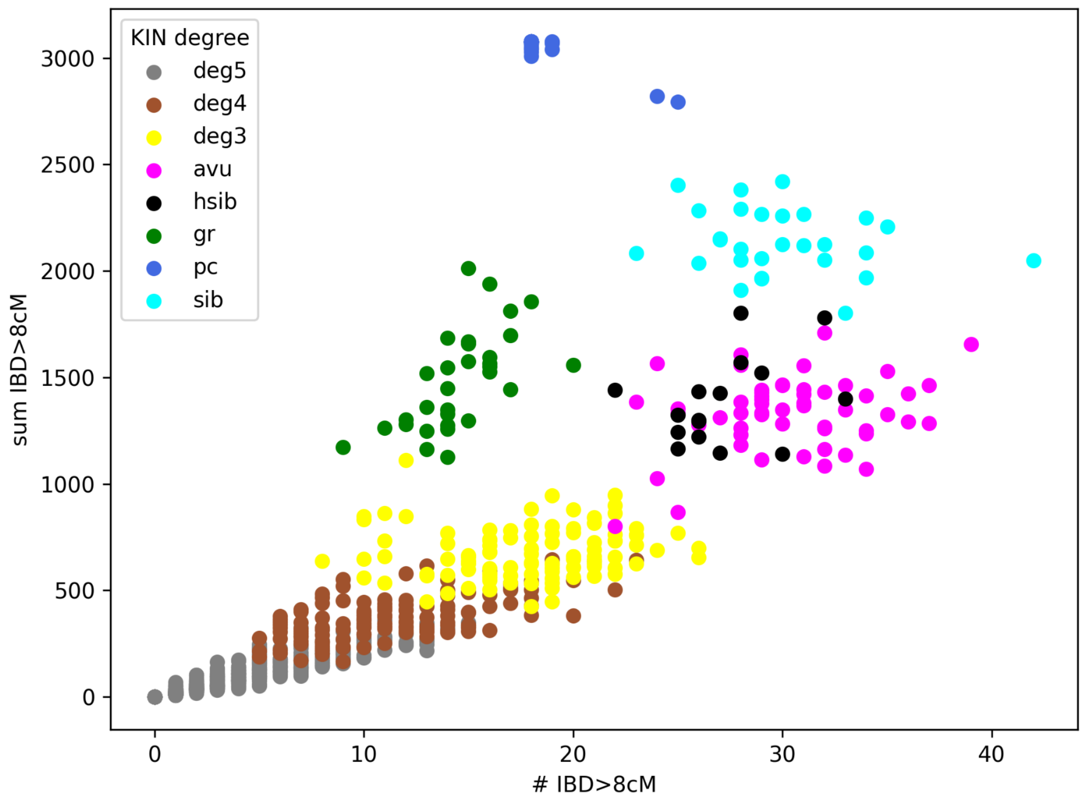

Fig. S12.1: *Summed IBD length per total number of IBDs for segments larger than 8cM for the different groups of relatedness inferred with KIN for the Altheim site.*

Two 2nd and 3rd degree relations could be resolved thanks to more distant relatedness to other family members which allowed us to exclude all possible topologies except one; we report these below.

Alh_267-Alh_133 (2nd degree - avuncular)
Alh_267 and Alh_133 have an avuncular relatedness; they share the same Y haplogroup so likely a pair of nephew-uncle on the paternal side.
Alh_267 has a 3rd degree relatedness with the older Alh_45, while Alh_133 is 4th degree related to Alh_45. Since there is only one degree of relatedness as difference it means that Alh_45 is 3rd degree related both to the uncle and to the father of the nephew, making the nephew 4th degree related; and this implies that Alh_267 is the uncle and Alh_133 the nephew.

Alh_133-Alh_154 (3rd degree)
Alh_133 and Alh_154 are 3rd degree related; Alh_267 (Alh_133's uncle) is 4th degree related to Alh_154. This means that Alh_133's father/Alh_267's brother is 3rd degree related to Alh_154, and a child of his (so a sibling of Alh_133) is 2nd degree related to Alh_154. This makes someone 1st degree related to Alh_154 also 1st degree related to Alh_133's sibling. The children of Alh_154 are not 1st degree related to Alh_133, but 4th; and the family of Alh_154 on his paternal side is unrelated/5th degree related (the IBD distribution for these two classes overlap) to Alh_133 so it can't be a full sibling of Alh_154 either. The only possibility left is that it's Alh_154's mother to be 1st degree related to Alh_133's sibling. So Alh_154's mother is 1st degree related to Alh_133's sibling, the two do not share the mt Haplogroup, so Alh_154's mother is either the half sister on the

father side of Alh_133 or the daughter of his brother; if the former was true Alh_154 would be 3rd degree related to Alh_267 (while it is 4th), so Alh_154's mother is the daughter of Alh_133's full sibling.

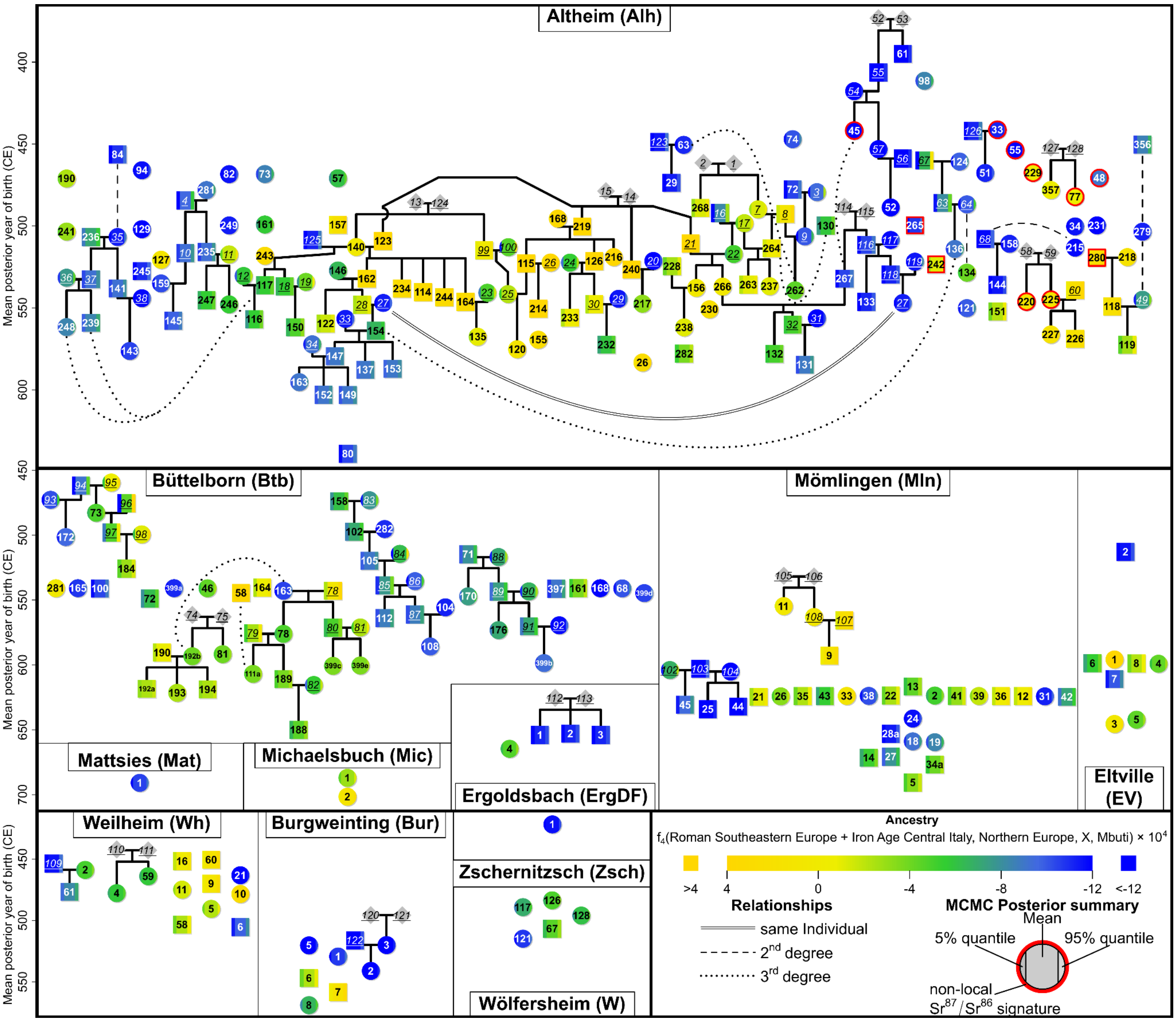

Fig. S12.2: *Reconstructed Pedigrees with colour coded f_4-statistic values presented in a chronological context. Sequenced individuals are shown in bold, with their lab code formed from the site shorthand (next to the site label) and their number. Non-sequenced individuals, inferred from the pedigree structure, are shown in italics and underlined, with IDs constructed as "ind" followed by their number. Individuals not connected to a pedigree are displayed for contextual reference.*

Long distance connections

We identified several close genetic connections between individuals from sites separated by more than 80 km.

One individual from Altenerding: AED92 [14], has a connection to SZ8 and SZ14, two siblings part of a kindred group in Szólad associated with a Longobard elite [15]. Due to the low sequencing depth of these three genomes, the degree of relatedness is uncertain and varies from 3rd to 4th degree, with 4th degree being more likely since that is the result obtained using the genome of SZ14, that has a higher overlap with the genome of AED92. Given that no relation has been identified with the relatives on the paternal side of the two siblings, that they both died before reaching adulthood [221] and that they were born around a century after AED92, the relatedness was likely mediated by their unsampled mother, though the low coverage of the genomes involved might hinder the detection of such links. On the basis of these results, the common ancestors between them likely lived at the same time of AED92 or a generation before, in the first half of the 5th century. Another connection is found between the two aforementioned Szólad siblings and two siblings from Straubing-Bajuwarenstrasse (STR491 and STR355c). The higher sequencing depth of STR355c allowed for IBD-based analyses which revealed a connection (5th to 7th degree) to some of the relatives of the two siblings on the paternal side (SZ7, SZ13, SZ22, Supplementary Table 3). Given that such IBDs are shared by all relatives on the paternal side, the most parsimonious interpretation is to consider SZ24, the father and grandfather of them all, the one with the closest relation with the two siblings from Straubing-Bajuwarenstrasse. In that light, the higher degree of relatedness that *KIN* identifies with between STR491 and SZ8-14 is probably an artifact due to the low coverage, as the *KIN* results obtained using STR355c (the sibling with the higher sequencing depth) would also suggest.

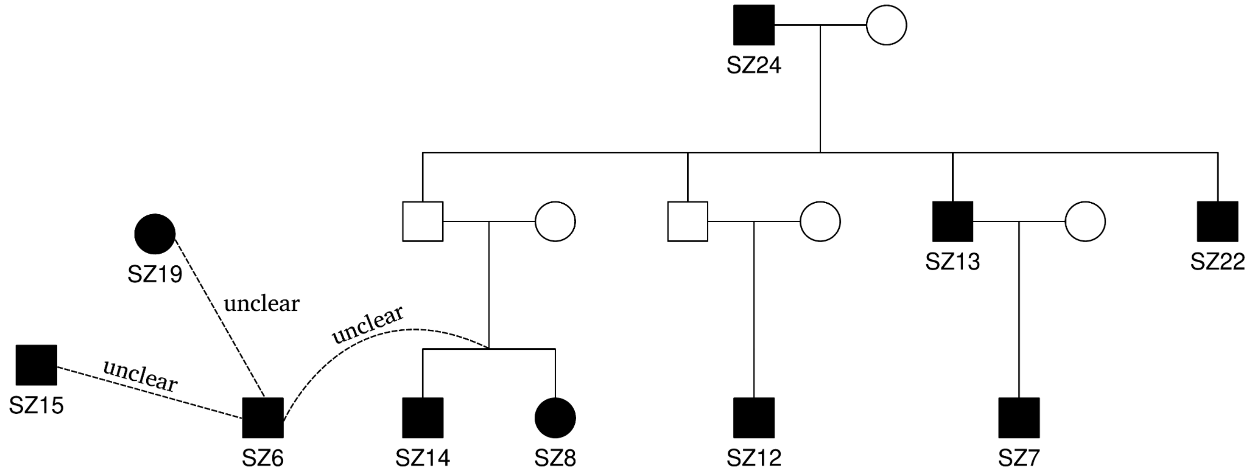

Fig. S12.3: Pedigree of the Szólad kindred group, as published in Amorim et al. (2018) [15], illustrating genetic connections to individuals in the Danube-Isar region.

Table S12.1: *KIN results for a subset of individuals, including the number of overlapping SNPs.*

ID1	ID2	Sex 1	Sex 2	Relatednes s	Second Guess	loglik ratio	SNPs overlap
AED1119	Bur2	F	F	avu	gr	25.6042	38581
AED1119	Bur3	F	F	sib	deg3	17.2819	30192
Bur2	Bur3	F	F	pc	gr	34.8382	215515
AED92b	Alh_158	M	F	deg3	deg4	1.3354	38894
AED92b	SZ14	M	M	deg4	deg3	3.5699	25878
AED92b	SZ8	M	F	deg3	deg4	1.4326	20988
AED92b	SZ13	M	M	un	deg3	16.8244	101736
AED92b	SZ22	M	M	un	deg3	16.4882	88345
AED92b	SZ24	M	M	un	deg3	16.3624	57470
STR355c	STR491	F	M	sib	gr	21.3664	15772
STR491	SZ8	M	F	deg4	deg3	1.7060	7462
STR491	SZ14	M	M	un	deg3	10.6285	9627
STR355c	SZ8	F	F	deg5	deg3	11.6176	70690
STR355c	SZ14	F	M	un	deg3	15.9001	93808
STR355c	SZ24	F	M	deg5	deg5	11.1166	246081
STR355c	SZ22	F	M	deg5	deg3	12.8944	385547
STR355c	SZ13	F	M	deg5	deg3	11.6133	449473
STR355c	SZ7	F	M	deg5	deg4	11.3989	412765
STR355c	SZ12	F	M	un	deg3	3.44148	286356
STR355c	SZ19	F	F	un	deg3	4.20427	419641

Estimating sampling completeness

Information on relationships allows us to obtain estimates on the fraction of the population buried in a cemetery for which we could obtain sequence data. To do so, we focus on all pairs of parents for which at least one child and one parent was interred and sequenced. We then assume that if one child and one parent was buried in a cemetery, also the other parent was likely interred there. Under this assumption, the fraction of pairs of parents for which only one or both parents were sequenced is then informative about the fraction of the whole population that were sequenced.

Let m denote the unknown fraction of the full population that was sequenced and let N be the number of pairs of parents of which at least one parent and child were sequenced. The probability that for pair $i = 1, ..., N$ only one ($b_i = 1$) or both ($b_i = 2$) parents were sequenced are the binomial probabilities conditioned on at least one parent sequenced and given by

$$P(b_i = 1|m) = \frac{2m(1-m)}{2m(1-m)+m^2} = \frac{2-2m}{2-m};\ P(b_i = 2|m) = \frac{m^2}{2m(1-m)+m^2} = \frac{m}{2-m}. \qquad \text{(S12.1)}$$

Let N_1 and N_2 denote the number of pairs which only one or both parents were sequenced, respectively, with $N_1 + N_2 = N$. The likelihood for $L(m)$ is given by

$$L(m) = P(N_1, N_2|m) = \left(\frac{2-2m}{2-m}\right)^{N_1}\left(\frac{m}{2-m}\right)^{N_2}. \qquad \text{(S12.2)}$$

Using a Beta prior $m \sim Beta(\alpha, \beta)$, the posterior is given by

$$P(m|N_1, N_2) \propto \frac{m^{N_2+\alpha-1}(1-m)^{N_1+\beta-1}}{(2-m)^N}. \qquad \text{(S12.3)}$$

Since there is no analytical solution for the proportionality constant (due to conditioning, no conjugate prior exists), we obtain the proportionality constant numerically using trapezoidal rule on 1000 different values for m, equally spaced values between 0 and 1, and obtain quantiles by numerical interpolation of the normalized posterior densities at those values.

Results are shown in Figure S12.4. Note that these estimates are conservative: if the main assumption was violated, i.e. if some parents were buried in different cemeteries, the true fraction of the population sampled was higher.

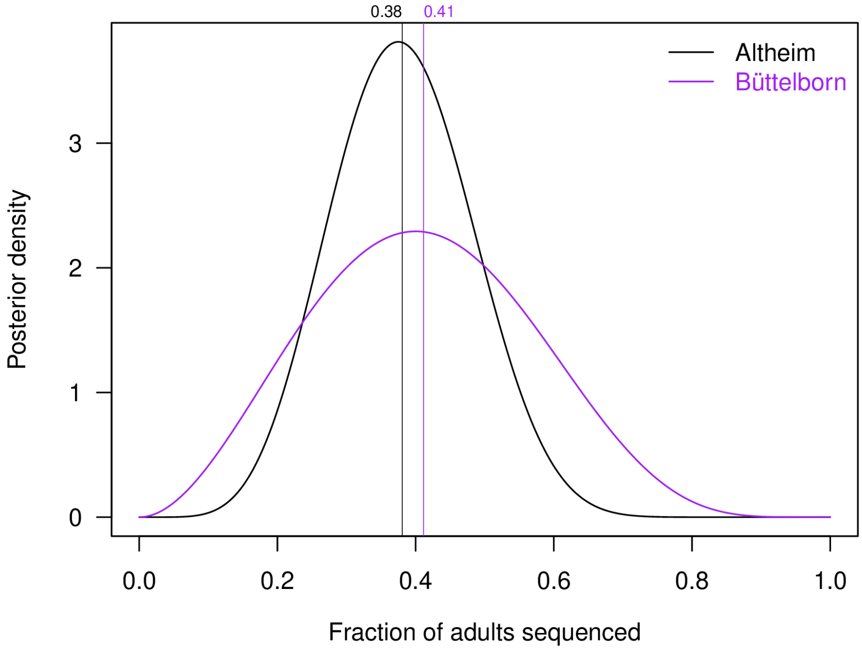

Fig. S12.4: *Posterior estimates on the fraction of adult individuals sequenced at the Altheim (black) and Büttelborn (purple) cemeteries. Numbers on top show the posterior mean estimates of 0.381 for Altheim and 0.411 for Büttelborn, the corresponding 90% CI ranges are 0.217 - 0.550 and 0.155 - 0.679, respectively.*

S13. Refining ancestry estimates on pedigrees

Here, we present *filia* (Family-tree Informed Learning of Ancestry), a novel tool that accounts for and leverages relatedness when inferring genetic ancestry. *filia* not only enhances the precision of previously obtained f_4-statistic (or D-statistic) estimates, but also allows for the imputation of these for non-sequenced individuals which are known to have existed due to the pedigree structure. f_4 and D-statistics are widely used in ancient DNA studies as a measure of shared genetic drift and are especially useful for detecting admixture events and distinguishing population structure.

The Model

We group individuals of pedigrees into two groups: *founders*, whose parents are not part of the known pedigree, and *offspring*. Founders represent the only independent observations, while offspring are descendants whose f_4 or D-values are derived from the mean of their parental values based on inheritance assumptions. Although founders provide the primary independent data points, the f_4 or D-values of offspring are still valuable, as they inform and refine the inference of founder values by providing additional constraints through familial relationships, thereby enhancing the robustness of founder estimates.

The model assumes that the true f_4 or D-values of founders are drawn from a mixture of two normal distributions, corresponding to two hypothesized source populations, each defined by separate means $\mu = (\mu_1, \mu_2)$ and variances $\sigma = (\sigma_1, \sigma_2)$. The fraction of founders belonging to the first population is denoted by π. We thus calculate the probability of the true value D_i of founder i as

$$P(D_i|\pi, \mu, \sigma^2) = \pi\phi(D_i; \mu_1, \sigma_1^2) + (1 - \pi)\phi(D_i; \mu_2, \sigma_2^2) \qquad \text{(S13.1)}$$

Offspring D-values are calculated as the mean of parental values as

$$D_j = \frac{1}{2}\,(D_{P_j^{(1)}} + D_{P_j^{(2)}}), \qquad \text{(S13.2)}$$

where we denote by $P_j^{(1)}$ and $P_j^{(2)}$ the indices of the two parents of offspring j. Observed f_4 or D-values d_i are noisy estimates of true values D_i. We assume a normally distributed error with mean zero and variance σ_D^2, which is also inferred, such that

$$d_i|D_i, \sigma_D^2 \sim N(D_i, \sigma_D^2) \qquad \text{(S13.3)}$$

We seek to obtain Bayesian estimates of all parameters of the model. As prior distributions, we assume that π follows a symmetric Beta distribution with both shape parameters set to 3, variance parameters σ_1^2, σ_2^2 and σ_D^2 an exponential distribution with rate 1000, and means μ_1 and μ_2 a normal distribution with mean 0 and variance 0.001. We note that the f_4 or D-values of two unsampled founding parents are non-identifiable as only their average is relevant for the values of the offspring. For these cases, we infer a single value for both parents.

Inference

We obtain Bayesian posterior estimates using an MCMC scheme with Metropolis-Hastings updates, which we implemented in the C++ program *filia* (we used commit "2fa4117") using the *stattools* library for statistical inference (https://bitbucket.org/wegmannlab/stattools, commit "8f10418"). Prior to running the MCMC, we obtained initial parameter guesses.

For the mixture parameters π, σ_1^2, σ_2^2, μ_1 and μ_2 we first divide the founders into two groups at the median observed f_4 or D-value and use these groups to get first estimates of all parameters. We then refine these estimates using an expectation-maximization (EM) algorithm. We then initialize values of unsampled individuals as follows: First, any unsampled offspring are set to the mean of their parents' values. Second, unsampled individuals with initialized offspring are set to the mean of their offspring's values, adjusted by the value of the other parent, if available. This is done iteratively. Third, remaining founders are sampled randomly from the mixture distribution, after which any unset offspring are assigned the means of their parents' values. After initialization, we run an MCMC chain for 100,000 burn-in iterations, followed by an additional 100,000 iterations for parameter estimation.

S14. The origin of the modern Central European kinship system

Association of inferred genetic relatedness and burial locations

Graveplans for Altheim and Büttelborn were overlaid with a grid in inkscape enabling the assignment of arbitrary X/Y coordinates to each grave midpoint and the computation of pairwise Euclidean distances. Differences in the distributions of pairwise distances were assessed using a two-sided Mann-Whitney-U test.

Altheim

In Altheim,strong spatial clustering according to genetic relationship was observed. Across 32 comparisons, significant differences in burial distances were detected, as reported in Table S14.1. First degree relatives were found to be buried closer together, compared to any other category of relatedness. Second degree relatives were also found to be buried closer together, compared to third or lesser degree relatives. No distinction between third and fourth degree relatives could be made, but the group of third degree relatives were still found to be buried closer together, compared to fifth degree relatives. This was also true for fourth degree relatives. When first and second degree categories were further sub-divided into smaller classes, grandparent-grandchild pairs were buried closer together than fourth degree relatives, siblings, as well as parent-child pairs were buried closer than third degree relatives, while avuncular related pairs were found closer together, compared to fourth or fifth degree relatives. Spouses could be distinguished from fifth degree relatives and unrelated pairs, although it has to be mentioned that we only could identify 6 spouses reliably.

Table S14.1*: Mann-Whitney-U test results with p-values < 0.05, comparing distributions of pairwise euclidean distances of individuals buried at the Altheim cemetery. Group_1/2 list the different categories of relatedness, as inferred by KIN as avuncular (avu), first degree to fifth degree (deg1, deg2, deg3, deg4, deg5), parent-child (pc), siblings (sib), half-siblings (hsib), grandparent - grandchild (gr) and unrelated (un), as well as spouses (spo) detected through common offspring. Numbers of pairs in each category (N_1, N_2) and their average euclidean distances (Dist_1, Dist_2) are also reported for each comparison.*

**Group_1**	**Group_2**	**Stat**	**P**	**N_1**	**N_2**	**Dist_1**	**Dist_2**
avu	deg1	2508	4.51E-02	54	77	56.85	40.97
avu	deg4	2818	1.05E-02	54	137	56.85	102.03
avu	deg5	23365.5	6.03E-08	54	1527	56.85	148.92
avu	un	49838	1.11E-06	54	3007	56.85	138.89
deg1	deg2	3208	4.59E-02	77	101	40.97	57.93
deg1	deg3	2566	1.04E-04	77	101	40.97	70.32
deg1	deg4	2966	1.10E-07	77	137	40.97	102.03
deg1	deg5	22793	1.12E-19	77	1527	40.97	148.92
deg1	un	49691	1.08E-17	77	3007	40.97	138.89
deg2	deg4	5072	4.37E-04	101	137	57.93	102.03
deg2	deg5	41982.5	1.62E-14	101	1527	57.93	148.92

deg2	un	89921	2.91E-12	101	3007	57.93	138.89
deg3	deg5	52184.5	5.09E-08	101	1527	70.32	148.92
deg3	un	110540	3.20E-06	101	3007	70.32	138.89
deg4	deg5	81614.5	1.99E-05	137	1527	102.03	148.92
deg4	un	171906	1.04E-03	137	3007	102.03	138.89
deg5	un	2423951.5	2.10E-03	1527	3007	148.92	138.89
gr	deg4	1428	4.49E-03	31	137	61.60	102.03
gr	deg5	11752	1.55E-06	31	1527	61.60	148.92
gr	un	25264	1.12E-05	31	3007	61.60	138.89
hsib	deg5	6865	2.55E-03	16	1527	54.43	148.92
hsib	un	14819	7.99E-03	16	3007	54.43	138.89
pc	deg3	1606	5.05E-04	49	101	40.05	70.32
pc	deg4	1877	4.81E-06	49	137	40.05	102.03
pc	deg5	14069	9.78E-14	49	1527	40.05	148.92
pc	un	30890	2.89E-12	49	3007	40.05	138.89
sib	deg3	960	9.57E-03	28	101	42.58	70.32
sib	deg4	1089	3.22E-04	28	137	42.58	102.03
sib	deg5	8724	7.70E-08	28	1527	42.58	148.92
sib	un	18801	4.47E-07	28	3007	42.58	138.89
deg5	spo	0.62999	7.99E-03	1527	6	148.92	32.08
un	spo	0.58696	1.71E-02	3007	6	138.89	32.08

Büttelborn

For Büttelborn, we limited the relatedness categories to first-, second-, and third-degree relatives due to the smaller sample size compared to Altheim. Additionally, fewer related individuals were identified overall. First degree relatives were buried significantly closer together than all other groups, whereas no significant differences in burial distances were observed for more distant degrees of relatedness.

Table S14.2: *Mann-Whitney-U test results with p-values < 0.05, comparing distributions of pairwise euclidean distances of individuals buried at the Büttelborn cemetery. Group_1/2 list the different categories of relatedness, as inferred by KIN as first degree to third degree (deg1, deg2, deg3) and unrelated (un). Numbers of pairs in each category (N_1, N_2) and their average euclidean distances (Dist_1, Dist_2) are also reported for each comparison.*

**Group_1**	**Group_2**	**Stat**	**P**	**N_1**	**N_2**	**Dist_1**	**Dist_2**
deg1	deg2	103.5	0.0057	20	21	270.17	519.99
deg1	deg3	47	0.0209	20	10	270.17	535.09
deg1	un	2811	2.77E-06	20	728	270.17	603.92
deg2	deg3	105	1.0000	21	10	519.99	535.09
deg2	un	6388	0.1990	21	728	519.99	603.92
deg3	un	3301	0.6132	10	728	535.09	603.92

Ancestry and proximity of related individuals

IBD sharing of IBDs longer than 8 cM, excluding individuals with no sharing, shows a negative correlation (Pearson's r = -0.19, pval = 6.4e-10, 95% CI: -0.26 -0.14, computed using R function cor.test() which computes confidence intervals based on Fisher's Z transform) with geographic distance on the grave field.

When computing this correlation coefficient for each individual with everyone else (if there were at least 5 data points) and then correlating that value to the f_4(Roman Southeastern Europe + Iron Age Central Italy, Northern Europe, X, Mbuti) we found a significant correlation (Pearson's r = 0.47, p=8.55e-5, 95% CI: 0.25 0.64), indicating that there is a greater proximity in burials of individuals that share IBDs if they have a higher affinity for Northern European ancestry. For these analyses only the individuals buried in the northeastern part of the cemetery were used.

Long lasting family traditions

Büttelborn illustrates smaller family units, with burial proximities spanning up to five generations. Both pairs of spouses in the sample were buried close to each other, in both cases near their child(ren). A nuclear family (father, mother, and three children) is buried in a circular pattern, with the mother's sister nearby (main text Fig. 6). Distant relatives from later generations are placed at the edge of the circle, highlighting continued ancestral ties. Another family (red) demonstrates five generations of continuity, with spouses and one male child per generation buried together, except for the first-generation male. While the red and dark blue pedigrees exhibit clear spatial clustering, the orange pedigree, including daughters, shows minimal clustering.

In Altheim, the oldest individuals were buried on the western part of the cemetery, probably before the north-eastern part (where the majority of the analysed individuals were buried) was established. Two pedigrees, the black and green ones, have their oldest identified members (Alh_84 and Alh_72-63-29, respectively) buried on the western part, and the more recent members on the northeastern part. However, these individuals are only 2nd or 3rd degree related to the rest of their relatives found in the northeastern part, and given that we only sequenced a handful of individuals buried in the western part it is possible that they have closer relatives buried nearby. The majority of the families (green, blue, red, aquamarine, orange and pink), including the two more numerous and that we record for the highest number of generations (green and blue) are geographically clustered on the burial ground, but they do not seem to follow a precise order in their distribution (Fig. S14.1). Two pedigrees, the black and purple ones, show a much looser (if at all) spatial clustering. Assuming that the burial position reflected the concept of family identity that the Altheim society had, it is clear that it could be preserved for several generations: the blue and green families, that we record in Altheim for 6 and 7 generations respectively, show close proximity in their burials for 6 generations. In some cases the family identity seems to extend beyond direct descendancy. For instance, the light and dark green families are connected through half-siblings, and individuals Alh_130 and Alh_133, who have more distant relationships to the green cluster, are also included in the family burial area. Similarly, the dark/medium blue and medium blue clusters are linked through half-siblings, all sons of Alh_140. Out of six marriages, two pairs were buried near the wife's family, and one near the husband's, though neither had both sets of parents present. In one instance, a wife and her child were buried near her family, while the husband lies farther away. It is curious to notice that the unsampled father of Alh_156 and Alh_266 is the only one of the four brothers with

predominantly southern ancestry marrying a woman with a previous recorded family history in Altheim, his descendants are buried in the graveyard area of the wife's family.

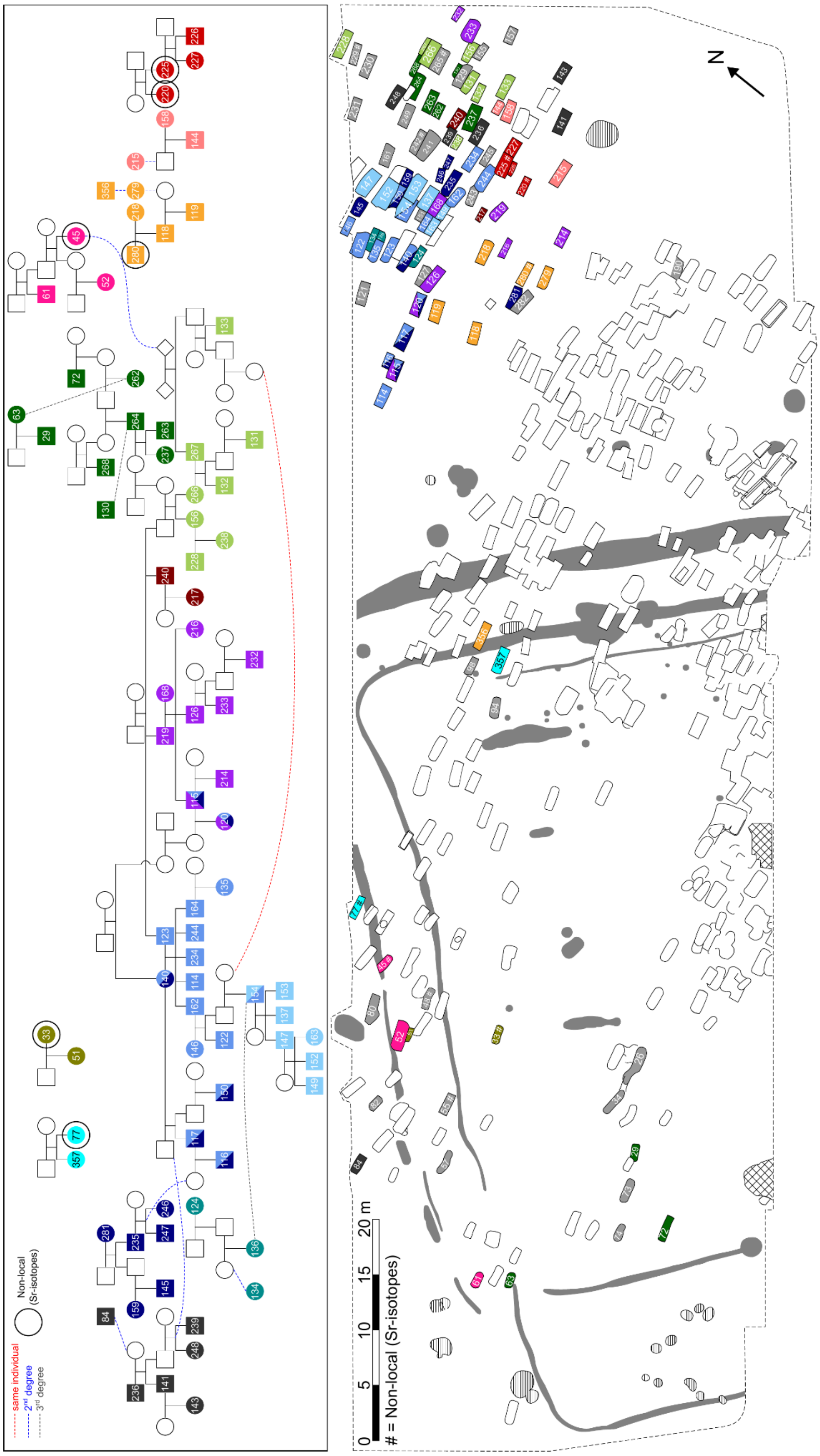

Fig. S14.1: *Position of family lineages in the Altheim graveyard; grave plan courtesy of Bayerisches Landesamt für Denkmalpflege; Dr. Johannes Sebrich*

Pedigree continuity

Table S14.3: *Pedigrees continued through the male or female line in Altheim and Büttelborn. We considered a lineage to continue through an individual if they had offspring and a sampled relative from a previous generation of the pedigree, indicating a pre-existing family history at the village*

**Altheim's pedigrees continued through**		**Büttelborn's pedigrees continued through**	
**males**	**females**	**males**	**females**
141	262's mother	189	78
248's father	141's mother	399c's father	111a
145's father	120's mother	176's father	
235	156	399b's father	
136's father	266	184's father	
117	52's mother	102	
150's father	119's mother	105	
162	154's mother	112's father	
154's father	45	108's father	
154			
147			
164			
115			
126			
118			
232's father			
131's father			
133's father			
133's brother			
267			

Table S14.4: Comparison of the kinship system of Altheim and Büttelborn with those of other european sites with extensive pedigrees that have been archaeogenetically investigated.
* *in Hazelton North, in most cases the multiple reproductive partners of females are related, which may suggest levirate, although it is not identified as such by the authors of this study.*

Site	pedigree continues through		rate of pedigree continuity through a daughter	levirate unions	single reproductive unions	multiple reproductive unions	rate of multiple reproductive unions	period
	son	daughter						
**Altheim**	20	9	0.31	no	42	4	0.087	Early Medieval
**Büttelborn**	9	2	0.182	no	16	1	0.059	Early Medieval
**Gurgy 'les Noisats' (Rivollat 2023)**	33	2	0.057	no	46	0	0	Neolithic
**Hazleton North (Fowler 2021)**	9	1	0.1	no*	9	3	0.25	Neolithic
**Frälsegården (Valeur Seersholm 2024)**	16	0	0	no	12	4	0.25	Neolithic
**Niederwünsch (Gretzinger 2025)**	24	4	0.143	no	37	7	0.159	Medieval
**Leobersdorf (Wang 2025)**	33	3	0.083	yes	42	14	0.25	Early Medieval
**Mödling (Wang 2025)**	small figure, impossible to count, described as strictly patrilocal			yes	130	31	0.193	Early Medieval
**Rákóczifalva (Gnecchi 2024)**	37	1	0.026	yes	42	24	0.364	Early Medieval
**Winterborne Kingston (Cassidy 2025)**	5	8	0.615	no	9	6	0.4	Iron Age

Evolution of the Altheim grave field

Burial dates suggest that the Altheim cemetery was founded in the western part, where the oldest individuals (including some the oldest members of some of the family clusters found in the more recent part of the cemetery) were interred. Later generations from the same families were buried in the northern corner, indicating a shift in burial location starting from the late 5th century CE (Supplementary Video S1). That part of the cemetery was completed around the second half of the 7th century, indicating a span of use lasting around one and a half centuries.
Having sampled almost the entire northeastern part of the Altheim grave field, it is possible to investigate the inhumation time to understand the evolution pattern of row graves cemetery. The

oldest graves there already occupy the majority of the cemetery area, and in the next ~150 years the empty spaces between them get filled by newer graves, with no apparent pre-ordinate pattern (Supplementary Video S1).

S15. Identifying a set of unrelated individuals for population genetic analyses

If not stated otherwise all population genetic analyses were performed using only unrelated individuals. To determine a set of unrelated individuals for each archaeological site for which more than one genome was sequenced, we build a network [222] graph in python3 connecting every pair of genomes that share at least 50 cM of summed IBD segments. We then used a greedy algorithm to retain the maximum number of individuals below this sharing threshold.

Table S15.1: *Subset of genomes identified as unrelated at each archaeological site, used in population genetics analyses.*

Site	Individuals
Altheim	Alh_121, Alh_127, Alh_129, Alh_151, Alh_155, Alh_157, Alh_158, Alh_159, Alh_161, Alh_168, Alh_190, Alh_215, Alh_218, Alh_227, Alh_228, Alh_229, Alh_230, Alh_231, Alh_236, Alh_240, Alh_241, Alh_242, Alh_243, Alh_245, Alh_249, Alh_26, Alh_268, Alh_279, Alh_280, Alh_281, Alh_282, Alh_33, Alh_34, Alh_48, Alh_55, Alh_57, Alh_63, Alh_72, Alh_73, Alh_74, Alh_77, Alh_80, Alh_82, Alh_94, Alh_98
Büttelborn	Btb100, Btb108, Btb158, Btb161_1, Btb165, Btb168, Btb172, Btb190, Btb281_1, Btb282, Btb397, Btb399a, Btb399d, Btb46, Btb58, Btb68, Btb71, Btb72_1, Btb78
Mömlingen	Mln12, Mln14, Mln18, Mln21, Mln22, Mln2, Mln24, Mln26, Mln28a, Mln31, Mln33, Mln34a, Mln35, Mln36, Mln38, Mln39, Mln41, Mln42, Mln43, Mln44, Mln5, Mln9
Weilheim	Wh10, Wh11, Wh16, Wh21, Wh58, Wh60, Wh61, Wh6, Wh9

S16. Estimating community sizes for Altheim and Büttelborn

The size of the communities in Altheim and Büttelborn were estimated by simulating populations of constant size *N*, evenly distributed in *n* villages, linked by migration with a migration rate *m*, for 10 generations, using the software SLiM [81]. In this model, mating pairs are monogamous. A thousand replicates of simulations were run and in each replicate the population size *N* was drawn in the interval [250; 500] individuals and the migration rate *m* in the interval [0; 1]. In each replicate, a number of *S* individuals was sampled in each village (*S*=120 for community size estimation in Altheim and *S*=40 for community size estimation in Büttelborn) and the percentage of related individuals was calculated for each village over 3 generations. A regression analysis was then carried out using the Python package statsmodels (v.0.14.4) to determine the relationship between the population size *N*, the migration rate *m* and the percentage of related individuals *r*. The resulting formulae were as follows:

(1) N = 1/3 * (2578.7831 + 493.2697 * m - 1696.9400 * r - 1897.8043 * m * r)
S = 120 individuals; n = 5 villages

(2) N = 1/3 * (1778.3874 - 115.3612 * m - 980.5865 * r - 1119.1278 * m * r)
S = 40 individuals; n = 5 villages

(3) N = 1/3 * (2128.6304 + 905.0604 * m - 1533.7329 * r - 1886.7032 * m * r)
S = 120 individuals; n = 2 villages

(4) N = 1/3 * (1596.6004 + 3.1817 * m - 1206.3950 * r - 502.4716 * m * r)
S = 40 individuals; n = 2 villages

Estimates of the migration rate *m* and the percentage of related individuals *r* can therefore be used to estimate the population size *N* and hence the village size N_v = *N*/5 (if n = 5 villages) or *N*/2 (if n = 2 villages). An estimate of the migration rate can be obtained by counting the percentage of individuals who have no direct ancestor in the sample. In Altheim, *r* is between 0.666 and 0.834 and the migration rate was estimated to be 0.51. Therefore, an estimate of N_v of [41; 70] individuals was obtained using formula (1) and of [84;155] individuals using formula (3). In Büttelborn, *r* is between 0.558 and 0.842 and the migration rate is estimated to be 0.575. N_v was estimated in the interval [23; 54] individuals using formula (2) and of [57;127] individuals using formula (4).

S17. Population genetic estimates of diversity

Estimation of deviation from Hardy-Weinberg-Equilibrium (F_{it})

To ensure accurate estimation of the coefficients, we used genome-wide data produced with the maximum-likelihood caller implemented in *ATLAS* [60] for the four largest groups of newly sequenced Early Medieval genomes (Altheim, Büttelborn, Mömlingen, Weilheim), as well as the two DZHK cohorts from Munich and Kiel. For greater robustness, genotype calls were filtered to include only sites with a minimum sequencing depth of two reads, omitting all transition sites. For greater comparability we further restricted calculation to sites present in at least three individuals from each ancient data-set. F_{it} was estimated per locus using scikit-allele v1.3.1 (https://github.com/cggh/scikit-allel) as 1-H_o/H_e. We computed 95% confidence intervals by resampling 1,000 times with 1,000 random loci each time. Values for main text Fig. 3A were estimated based on individuals alive at each given year sampled from 1000 *Chronograph* MCMC traces, by sampling every 15th entry from a total of 15000. Confidence intervals were estimated as the 2.5th and 97.5th quantile of the resulting distribution of F_{is} values. All F-estimates are based on only unrelated individuals, defined as described in S15. As sanity check we also estimated F based on all individuals, including closely related pairs (Fig. S17.1). As expected, higher values were obtained with increasing numbers of related pairs.

Estimation of pairwise differences (D_{xy}, π)

To ensure an accurate estimation for D_{xy} and π in the presence of missing data, we used PiXY [223] on the same transversion sites data set, filtered to only contain sites for which at least three individuals had data present. Pairwise differences were estimated for windows of 100,000 bp and then averaged for the whole autosomal genome.

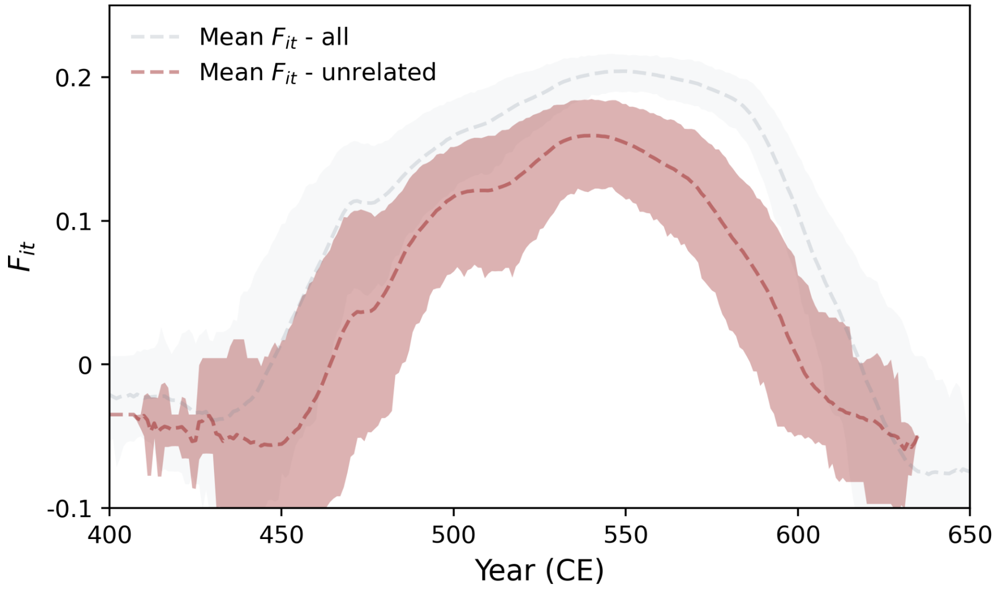

Fig. S17.1: *Deviations from Hardy-Weinberg-Equilibrium measured as mean F_{it} (solid line) for individuals potentially alive at Altheim for each given year, including 95% CI's indicated by the shaded area. Estimates are given based on all individuals (lightgrey) as well as a subset of individuals identified as unrelated (red).*

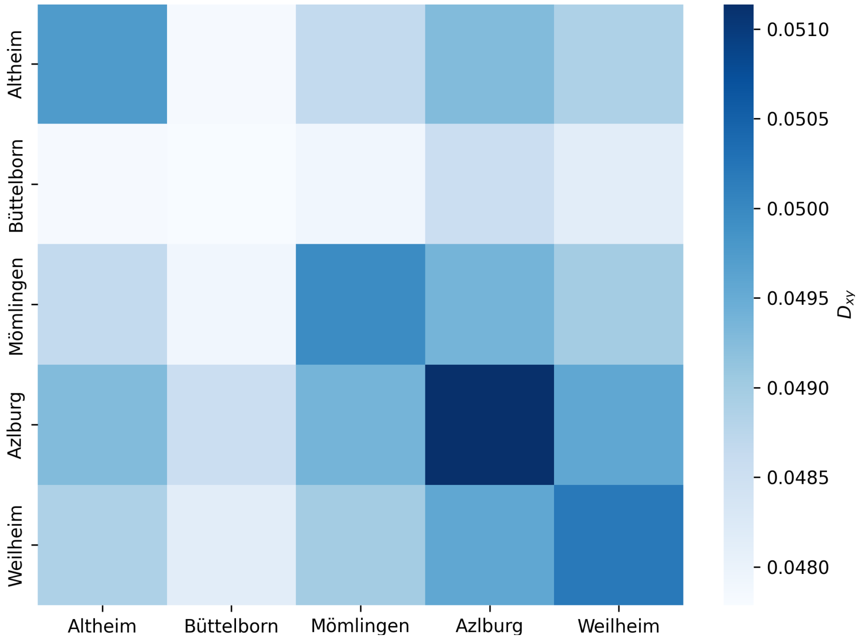

Fig S17.2: *Heatmap depicting D_{xy} and pi (diagonal) for newly published sites with more than 10 individuals.*

References

1. Meier, M. *Geschichte der Völkerwanderung. Europa, Asien und Afrika vom 3. bis zum 8. Jahrhundert n. Chr*. (C H Beck, Munich, Germany, 2021).
2. Schmidt-Hofner, S. Barbarian Migrations and the Economic Challenges to the Roman Landholding Elites in the Fourth Century CE. *J. late antiq.* **10**, 372–404 (2017).
3. Halsall, G. *Barbarian Migrations and the Roman West, 376-568*. (Cambridge University Press, 2007).
4. Bavuso, I. & Castrorao Barba, A. *The European Countryside during the Migration Period: Patterns of Change from Iberia to the Caucasus (300-700 CE)*. (De Gruyter, 2023).
5. Halsall, G. The origins of the Reihengräberzivilisation: Forty years on. in *Fifth-Century Gaul: A Crisis of Identity?* (eds. Drinkwater, J. & Elton, H.) 196–207 (Cambridge University Press, United States, 1992).
6. Brownlee, E. Grave Goods in Early Medieval Europe: regional variability and decline. *Internet Archaeol.* (2021) doi:10.11141/ia.56.11.
7. Hamerow, H. *Early Medieval Settlements: The Archaeology of Rural Communities in Northwest Europe, 400-900*. (Oxford University Press, 2004).
8. Brather-Walter, S. Social 'Mobility' or 'Distancing'? Spatial Organisation of Early Medieval Graveyards in South-Western Germany. in *The European Countryside during the Migration Period: Patterns of Change from Iberia to the Caucasus (300-700 CE)* (eds. Bavuso, I. & Castrorao Barba, A.) 201–231 (De Gruyter, 2023).
9. Theuws, F. Long-distance trade and the Rural population of Northern Gaul. in *The Oxford Handbook of the Merovingian World* (eds. Effros, B. & Moreira, I.) 883–915 (Oxford University Press, Oxford, 2020).
10. Brather, S. Pagan or Christian? Early medieval grave furnishings in Central Europe. in *Rome, Constantinople and newly-converted Europe. Archaeological and historical evidence. Volume 1* (eds. Salamon, M. et al.) 333–349 (Kraków, Leipzig, Rzeszów, Warszawa, 2012).
11. Sebrich, J. *Das spätantik-frühmittelalterliche Gräberfeld von Essenbach-Altheim. Materialhefte zur*

Bayerischen Archäologie 110. (Verlag Michael Laßleben, Kallmünz, 2019).

12. Heitmeier, I. & Haberstroh, J. *Gründerzeit: Siedlung in Bayern zwischen Spätantike und frühem Mittelalter*. (EOS Verlag, 2019).
13. Fehr, H. & Heitmeier, I. *Die Anfänge Bayerns. Von Raetien und Noricum zur frühmittelalterlichen Baiovaria*. (EOS Verlag, 2012).
14. Veeramah, K. R. *et al.* Population genomic analysis of elongated skulls reveals extensive female-biased immigration in Early Medieval Bavaria. *Proc. Natl. Acad. Sci. U.S.A.* **115**, 3494–3499 (2018).
15. Amorim, C. E. G. *et al.* Understanding 6th-century barbarian social organization and migration through paleogenomics. *Nat. Commun.* **9**, 3547 (2018).
16. Gretzinger, J. *et al.* The Anglo-Saxon migration and the formation of the early English gene pool. *Nature* **610**, 112–119 (2022).
17. Patterson, N. *et al.* Large-scale migration into Britain during the Middle to Late Bronze Age. *Nature* **601**, 588–594 (2022).
18. Antonio, M. L. *et al.* Stable population structure in Europe since the Iron Age, despite high mobility. *eLife* **13**, e79714 (2024).
19. Vyas, D. N. *et al.* Fine-scale sampling uncovers the complexity of migrations in 5th-6th century Pannonia. *Curr. Biol.* **33**, 3951–3961.e11 (2023).
20. Olalde, I. *et al.* A genetic history of the Balkans from Roman frontier to Slavic migrations. *Cell* **186**, 5472–5485.e9 (2023).
21. O'Sullivan, N. *et al.* Ancient genome-wide analyses infer kinship structure in an Early Medieval Alemannic graveyard. *Sci Adv* **4**, eaao1262 (2018).
22. Speidel, L. *et al.* High-resolution genomic history of early medieval Europe. *Nature* **637**, 118–126 (2025).
23. Speidel, L., Forest, M., Shi, S. & Myers, S. R. A method for genome-wide genealogy estimation for thousands of samples. *Nat. Genet.* **51**, 1321–1329 (2019).
24. Lawson, D. J., Hellenthal, G., Myers, S. & Falush, D. Inference of population structure using dense haplotype data. *PLoS Genet.* **8**, e1002453 (2012).
25. de Gennaro, L. *et al.* PANE: fast and reliable ancestral reconstruction on ancient genotype data with

non-negative least square and principal component analysis. *Genome Biol.* **26**, 29 (2025).

26. Lee, A. D. *War in Late Antiquity: A Social History*. (Blackwell Publishing, 2007).
27. McColl, H. *et al.* Steppe Ancestry in western Eurasia and the spread of the Germanic Languages. *bioRxiv* 2024.03. 13.584607 (2024) doi:10.1101/2024.03.13.584607.
28. Velte, M. *et al.* Between Raetia Secunda and the dutchy of Bavaria: Exploring patterns of human movement and diet. *PLoS One* **18**, e0283243 (2023).
29. Tian, Y. *et al.* The role of emerging elites in the formation and development of communities after the fall of the Roman Empire. *Proceedings of the National Academy of Sciences* **121**, e2317868121 (2024).
30. Treggiari, S. *Roman Marriage: Iusti Coniuges from the Time of Cicero to the Time of Ulpian*. (Oxford University Press, Oxford, England, 1991).
31. Frier, B. W. Demography. in *The Cambridge Ancient History: The High Empire, AD 70-192* (eds. Bowman, A. K., Garnsey, P. & Rathbone, D.) vol. 11 787–816 (Cambridge University Press, Cambridge, England, 2000).
32. Barbiera, I., Castiglioni, M. & Dalla-Zuanna, G. Demography, Peasantry, and Family in Early Medieval Provence, 813–814. *Population* **77**, 249–274 (2022).
33. Jussen, B. *Der Name der Witwe. Erkundungen zur Semantik der mittelalterlichen Bußkultur (Veröffentlichungen des Max-Planck-Instituts für Geschichte 158)*. (Göttingen, 2000).
34. Ubl, K. *Inzestverbot und Gesetzgebung: Die Konstruktion eines Verbrechens (300-1100) (Millennium-Studien 20)*. (De Gruyter, Berlin, Germany, 2008).
35. Evans-Grubbs, J. Marriage and Family Relationships in the Late Roman West. in *A companion to Late Antiquity* (ed. Rousseau, P.) 201–219 (Wiley-Blackwell, Oxford, UK, 2009).
36. Harper, K. Marriage and Family. in *The Oxford Handbook of Late Antiquity* (ed. Johnson, S. F.) 667–714 (Oxford, 2012).
37. Wang, K. *et al.* Ancient DNA reveals reproductive barrier despite shared Avar-period culture. *Nature* **638**, 1007–1014 (2025).
38. Saag, L. *et al.* North Pontic crossroads: Mobility in Ukraine from the Bronze Age to the early modern period. *Sci. Adv.* **11**, eadr0695 (2025).
39. Gnecchi-Ruscone, G. A. *et al.* Ancient genomes reveal origin and rapid trans-Eurasian migration of 7th

century Avar elites. *Cell* **185**, 1402–1413.e21 (2022).

40. Maróti, Z. *et al.* The genetic origin of Huns, Avars, and conquering Hungarians. *Curr. Biol.* **32**, 2858–2870.e7 (2022).
41. Gretzinger, J. *et al.* Ancient DNA connects large-scale migration with the spread of Slavs. *Nature* 1–10 (2025).
42. *Handbook of Critical Agrarian Studies*. (Edward Elgar Publishing, Cheltenham, England, 2021).
43. Kohl, T. *Lokale Gesellschaften: Formen der Gemeinschaft in Bayern vom 8. bis zum 10. Jahrhundert (Mittelalter-Forschungen 29)*. (Ostfildern, 2010).
44. Guyon, L., Guez, J., Toupance, B., Heyer, E. & Chaix, R. Patrilineal segmentary systems provide a peaceful explanation for the post-Neolithic Y-chromosome bottleneck. *Nat. Commun.* **15**, 3243 (2024).
45. Guyon, L., Heyer, E. & Chaix, R. Was descent in Neolithic and Bronze Age Europe patrilineal or bilateral? Preprint at https://doi.org/10.5281/ZENODO.15084652 (2025).
46. Ly, G. *et al.* From matrimonial practices to genetic diversity in Southeast Asian populations: the signature of the matrilineal puzzle. *Philos. Trans. R. Soc. Lond. B Biol. Sci.* **374**, 20180434 (2019).
47. Arjava, A. *Women and Law in Late Antiquity*. (Oxford University Press, Oxford, England, 1996).
48. Gardner, J. F. *Women in Roman Law & Society*. (Croom Helm, London & Sydney, 1986).
49. Shaw, B. D. & Saller, R. P. Close-kin marriage in Roman society? *Man* **19**, 432–444 (1984).
50. Cassidy, L. M. *et al.* Continental influx and pervasive matrilocality in Iron Age Britain. *Nature* **637**, 1136–1142 (2025).
51. Gnecchi-Ruscone, G. A. *et al.* Network of large pedigrees reveals social practices of Avar communities. *Nature* **629**, 376–383 (2024).
52. Rivollat, M. *et al.* Extensive pedigrees reveal the social organization of a Neolithic community. *Nature* **620**, 600–606 (2023).
53. Brather, S. *Ethnische Interpretationen in der frühgeschichtlichen Archäologie. Geschichte, Grundlagen und Alternativen (Ergänzungsbände zum Reallexikon der germanischen Altertumskunde 42)*. (Walter de Gruyter, Berlin, 2004).
54. Brather, S. Anfang und Ende der Reihengräberfelder. Der Wandel von Bestattungsformen zwischen Antike und Mittelalter. in *Antike im Mittelalter – Fortleben, Nachwirken, Wahrnehmung. 25 Jahre*

Forschungsverbund „Archäologie und Geschichte des ersten Jahrtausends in Südwestdeutschland“ (eds. Brather, S., Nuber, H. U., Steuer, H. & Zotz, T.) 217–234 (Ostfildern, 2014).

55. Pohl, W. Introduction – Strategies of Identification: A Methodological Profile. in *Strategies of Identification: Ethnicity and Religion in Early Medieval Europe* (eds. Pohl, W. & Heydemann, G.) 1–64 (Turnhout, 2013).
56. Schipp, O. *Der weströmische Kolonat von Konstantin bis zu den Karolingern (332 bis 861). Studien zur Geschichtsforschung des Altertums (21)*. (Verlag Dr. Kovac, Hamburg, Germany, 2009).
57. Schulz, I. *et al.* Ancient genomes provide evidence of demographic shift to Slavic-associated groups in Moravia. *Genome Biol.* **26**, 259 (2025).
58. Haubrichs, W. The Multilingualism of the Early Middle Ages: Evidence from Peripheral Regions of the *Regnum orientalium Francorum*. in *The Languages of Early Medieval Charters. Latin, Germanic Vernaculars and the Written Word* (eds. Gallagher, R., Roberts, E. & Tinti, F.) 68–116 (Leiden/Boston, 2021).
59. Zedda, N. *et al.* Biological and substitute parents in Beaker period adult-child graves. *Sci. Rep.* **13**, 18765 (2023).
60. Link, V. *et al.* ATLAS: Analysis Tools for Low-depth and Ancient Samples. *bioRxiv* 105346 (2017) doi:10.1101/105346.
61. Tarasov, A., Vilella, A. J., Cuppen, E., Nijman, I. J. & Prins, P. Sambamba: fast processing of NGS alignment formats. *Bioinformatics* **31**, 2032–2034 (2015).
62. McKenna, A. *et al.* The Genome Analysis Toolkit: a MapReduce framework for analyzing next-generation DNA sequencing data. *Genome Res.* **20**, 1297–1303 (2010).
63. Rubinacci, S., Ribeiro, D. M., Hofmeister, R. J. & Delaneau, O. Efficient phasing and imputation of low-coverage sequencing data using large reference panels. *Nat. Genet.* **53**, 120–126 (2021).
64. Rubinacci, S., Hofmeister, R., da Mota, B. S. & Delaneau, O. Imputation of low-coverage sequencing data from 150,119 UK Biobank genomes. *bioRxiv* 2022.11.28.518213 (2022) doi:10.1101/2022.11.28.518213.
65. Mathieson, I. *et al.* Genome-wide patterns of selection in 230 ancient Eurasians. *Nature* **528**, 499–503 (2015).

66. Schönherr, S., Weissensteiner, H., Kronenberg, F. & Forer, L. Haplogrep 3 - an interactive haplogroup classification and analysis platform. *Nucleic Acids Res.* **51**, W263–W268 (2023).

67. Ralf, A., González, D. M., Zhong, K. & Kayser, M. Yleaf: Software for Human Y-Chromosomal Haplogroup Inference from Next-Generation Sequencing Data. *Molecular Biology and Evolution* **35**, 1291–1294 (2018).

68. Mallick, S. *et al.* The Allen Ancient DNA Resource (AADR) a curated compendium of ancient human genomes. *Sci. Data* **11**, 182 (2024).

69. Mallick, S. & Reich, D. The Allen Ancient DNA Resource (AADR): A curated compendium of ancient human genomes. Harvard Dataverse https://doi.org/10.7910/DVN/FFIDCW (2023).

70. Patterson, N., Price, A. L. & Reich, D. Population structure and eigenanalysis. *PLoS Genet.* **2**, e190 (2006).

71. Maier, R. *et al.* On the limits of fitting complex models of population history to f-statistics. *Elife* **12**, (2023).

72. Maier, R. & Patterson, N. admixtools: Inferring demographic history from genetic data. R package version 2.0.4. *https://github.com/uqrmaie1/admixtools* (2024).

73. 1000 Genomes Project Consortium *et al.* A global reference for human genetic variation. *Nature* **526**, 68–74 (2015).

74. Chacón-Duque, J.-C. *et al.* Latin Americans show wide-spread Converso ancestry and imprint of local Native ancestry on physical appearance. *Nat. Commun.* **9**, 5388 (2018).

75. Ringbauer, H., Novembre, J. & Steinrücken, M. Parental relatedness through time revealed by runs of homozygosity in ancient DNA. *Nat. Commun.* **12**, 5425 (2021).

76. Ringbauer, H. *et al.* Accurate detection of identity-by-descent segments in human ancient DNA. *Nat. Genet.* **56**, 143–151 (2024).

77. Popli, D., Peyrégne, S. & Peter, B. M. KIN: a method to infer relatedness from low-coverage ancient DNA. *Genome Biol.* **24**, 10 (2023).

78. Alaçamlı, E. *et al.* READv2: advanced and user-friendly detection of biological relatedness in archaeogenomics. *Genome Biol.* **25**, 216 (2024).

79. Blöcher, J. *et al.* Descent, marriage, and residence practices of a 3,800-year-old pastoral community in

Central Eurasia. *Proc. Natl. Acad. Sci. U.S.A.* **120**, e2303574120 (2023).

80. Pouyet, F., Aeschbacher, S., Thiéry, A. & Excoffier, L. Background selection and biased gene conversion affect more than 95% of the human genome and bias demographic inferences. *Elife* **7**, (2018).

81. Haller, B. C. & Messer, P. W. SLiM 3: Forward genetic simulations beyond the Wright-Fisher model. *Mol. Biol. Evol.* **36**, 632–637 (2019).

82. Seabold, S. & Perktold, J. Statsmodels: Econometric and Statistical Modeling with Python. in *Proceedings of the 9th Python in Science Conference* (eds. van der Walt, S. & Millman, J.) 92–96 (2010).

83. *The GEBCO_2022 Grid - the 2022 Compilation of a Continuous Terrain Model of the Global Oceans and Land.*

84. Hunter, J. D. Matplotlib: A 2D Graphics Environment. *Comput. Sci. Eng.* **9**, 90–95 (2007).

85. Koch, U. *Das Alamannisch-Fränkische Gräberfeld Bei Pleidelsheim. Forschungen Und Berichte Zur Vor- Und Frühgeschichte in Baden-Württemberg. Band 60*. (Theiss, 2001).

86. Harbeck, M. & von Heyking, K. *Standardisierte Skelettdokumentation*. https://sam.snsb.de/sammlung-forschung/downloads/ (11.2023).

87. Göldner, H. '... südlich und nördlich des Dornheimer Pfades'. Die Wiederentdeckung des fränkischen Reihengräberfeldes bei Büttelborn. in *hessenARCHÄOLOGIE 2001. Jahrbuch für Archäologie und Paläontologie in Hessen* (ed. Landesamt für Denkmalpflege Hessen, hessenARCHÄOLOGIE) 136–140 (2002).

88. Göldner, H. Scheibenfibel und Mantelschließe - ein Nachtrag zum fränkischen Gräberfeld von Büttelborn. in *hessenARCHÄOLOGIE 2006. Jahrbuch für Archäologie und Paläontologie in Hessen* (ed. Landesamt für Denkmalpflege Hessen, hessenARCHÄOLOGIE) 109–111 (2007).

89. Göldner, H. Alles begann während einer Geschichtsstunde im Jahre 1900... in *hessenARCHÄOLOGIE 2012. Jahrbuch für Archäologie und Paläontologie in Hessen* (ed. Landesamt für Denkmalpflege Hessen, hessenARCHÄOLOGIE) 117–119 (2013).

90. Koch, R. *Bodenfunde der Völkerwanderungszeit aus dem Main-Tauber-Gebiet. Germanische Denkmäler der Völkerwanderungszeit. A 8*. (de Gruyter, Berlin, 1967).

91. Liebetrau, S. Modeschmuck der Merowingerzeit: Perlen aus dem Mömlinger Gräberfeld. in *1200 Jahre Mömlingen* (ed. Hartmann, W.) 75–85 (Mömlingen, 2017).

92. Marquart, M. Frühmittelalterliche Gräber in der Mömlinger Gemarkung. Das Reihengräberfeld in der Flur Hollerstrauch. in *1200 Jahre Mömlingen* (ed. Hartmann, W.) 63–74 (Mömlingen, 2017).

93. Piller, N. Grabungsbericht M-2019-1653-2; Weilheim, WM: Am Meisteranger, G-2019. PLANAteam Archäologie, Wasserburg a. Inn, www.planateam.de. (2020).

94. Carlichi-Witjes, N. & von Heyking, K. Anthropologischer Befund der Skelettfunde aus der Grabung „Weilheim“, Maßnahme M-2019-1653-2. AnthroArch GbR, www.anthroarch.de. (2023).

95. Breda, A. *et al.* San Pietro in Mavinas a Sirmione. in *Nuove Ricerche sulle Chiese Altomedievali del Garda. 3rd Convegno Archeologico del Garda 2010* (ed. Brogiolo, G. P.) 33–64 (2011).

96. Pollak, M. Ein gallo-fränkischer Offizier im Alpenraum? Neues zum Amtsträger der Straßenstation Iuenna / Globasnitz. *Carinthia I* **2020 (210. Jahrgang)**, 91–119 (2020).

97. Mitschke, S. *Zur Erfassung und Auswertung archäologischer Textilien an korrodiertem Metall. Eine Studie zu ausgewählten Funden aus dem Gräberfeld von Eltville, Rheingau-Taunus-Kreis (5.-8. Jh. n. Chr.). Kleine Schriften 51*. (Vorgeschichtliches Seminar der Philipps-Universität Marburg, Marburg, 2001).

98. Becker, T., Jünger, K., Sarnowski, D., Schnell, G. & Steffens, P. Abschluss der Grabungen im merowingerzeitlichen Gräberfeld von Eltville. in *hessenARCHÄOLOGIE 2016. Jahrbuch für Archäologie und Paläontologie in Hessen* (ed. Landesamt für Denkmalpflege Hessen, hessenARCHÄOLOGIE) 117–122 (2017).

99. Blaich, M. C. Das frühmittelalterliche Gräberfeld von Eltville, Rheingau-Taunus-Kreis. Beiträge zur Siedlungsgeschichte des Rheingaus vom 5. bis 8. Jahrhundert n.Chr. (Dr. Rudolf Habelt, Bonn, Germany, 2006).

100. Codreanu-Windauer, S. *Klein aber fein. Frühmittelalterliche Gräberfelder in Burgweinting*. in *Die Spuren von Jahrtausenden: Archäologische Funde und Ergebnisse der Großgrabung von Regensburg-Burgweinting* (eds. Boos, A., Ontrup, M., Wolf, G. & Zuber, J.) 305–335 (Universitätsverlag Regensburg, Regensburg, Germany, 2020).

101. Monroy Kuhn, J. M., Jakobsson, M. & Günther, T. Estimating genetic kin relationships in prehistoric

populations. *PLoS One* **13**, e0195491 (2018).

102. Korneliussen, T. S. & Moltke, I. NgsRelate: a software tool for estimating pairwise relatedness from next-generation sequencing data. *Bioinformatics* **31**, 4009–4011 (2015).

103. Lipatov, M., Sanjeev, K., Patro, R. & Veeramah, K. R. Maximum Likelihood Estimation of Biological Relatedness from Low Coverage Sequencing Data. *bioRxiv* 023374 (2015) doi:10.1101/023374.

104. Lindenthal, J. Überraschender Fund eines Gräberfelds der Merowingerzeit. in *hessenARCHÄOLOGIE 2006. Jahrbuch für Archäologie und Paläontologie in Hessen* (ed. Landesamt für Denkmalpflege Hessen, hessenARCHÄOLOGIE) 93–95 (2007).

105. Lindenthal, J. & Piffko, S. Das merowingerzeitliche Gräberfeld von Wölfersheim-Berstadt - ein erstes Fazit nach Grabungsende. in *hessenARCHÄOLOGIE 2007. Jahrbuch für Archäologie und Paläontologie in Hessen* (ed. Landesamt für Denkmalpflege Hessen, hessenARCHÄOLOGIE) 105–109 (2008).

106. Saal, E. & Lindenthal, J. Vom Armschmuck zum Kettenanhänger. in *hessenARCHÄOLOGIE 2016. Jahrbuch für Archäologie und Paläontologie in Hessen* (ed. Landesamt für Denkmalpflege Hessen, hessenARCHÄOLOGIE) 123–125 (2017).

107. Becker, T., Saal, E. & Steffens, P. Ganz schön bunt… - Zur visuellen Präsentation der Perlen aus dem frühmittelalterlichen Gräberfeld bei Wölfersheim-Berstadt. *Denkmalpflege & Kulturgeschichte* **1-2019**, 2–5 (2019).

108. Peek, C. & Saal, E. Von gemusterten Stoffen und aufwendig gefibelter Kleidung. in *hessenARCHÄOLOGIE 2022. Jahrbuch für Archäologie und Paläontologie in Hessen* (ed. Landesamt für Denkmalpflege Hessen, hessenARCHÄOLOGIE) 160–164 (2023).

109. Simoneit, J. Die frühmittelalterlichen Grabgruppen von Ergoldsbach und Weng, Lkr. Landshut. Master thesis. (Ludwig-Maximilians-Universität München, Munich, 2020).

110. Richter, T. Von Münchshöfen bis zum Frühmittelalter - Die Ausgrabungen in Ergoldsbach-'Dörnbacher Feld'. in *Vorträge des 38. Niederbayerischen Archäologentages* (eds. Husty, L. & Schmotz, K.) 173–206 (Verlag Marie Leidorf GmbH, Rahden/Westf., 2020).

111. Zäuner, S. Ergoldsbach, „SG u. GG Ergoldsbach-Süd“ Lkr. Landshut Maßnahme M-2017-1857-2_0 Befund 32-I.

112. Zäuner, S. Ergoldsbach, „SG u. GG Ergoldsbach-Süd“ Lkr. Landshut Maßnahme M-2017-1857-2_0 Befund 32-II.
113. Zäuner, S. Ergoldsbach, „SG u. GG Ergoldsbach-Süd“ Lkr. Landshut Maßnahme M-2017-1857-2_0 Befund 32-III.
114. Koch, H. *Frühmittelalterliche Adelsgräber aus Ergolding*. (Faustus, Erlangen, 2014).
115. Weller, U., Kaiser, H. & Heynowski, R. *Kosmetisches und medizinisches Gerät: Erkennen - Bestimmen - Beschreiben*. (Deutscher Kunstverlag, München, 2016).
116. Zäuner, S. Ergoldsbach, „SG u. GG Ergoldsbach-Süd“ Lkr. Landshut Maßnahme M-2017-1857-2_0 Befund 59.
117. Brendle, T. Spätmerowingerzeitliche Ohrringe vom Typ Lauterhofen und eine bajuwarische Zuwanderin an der oberen Donau. in *Archäologische Nachrichten aus Baden 88/89* 30–41 (Förderkreis Archäologie in Baden, Heidelberg, 2014).
118. Spazier, I. & Queck, T. Archäologie an der Ferngasleitung 32. in *Neue archäologische Forschungen im Altenburger Land. Sonderveröffentlichung des Thüringischen Landesamtes für Denkmalpflege und Archäologie. Band 6* (ed. Spazier, I.) 11–27 (Verlag Beier & Beran, Langenweißbach, 2023).
119. Spazier, I. Zwei frühmerowingerzeitliche Gräber bei Zschernitzsch, Lkr. Altenburger Land. in *Neue archäologische Forschungen im Altenburger Land. Sonderveröffentlichung des Thüringischen Landesamtes für Denkmalpflege und Archäologie. Band 6* (ed. Spazier, I.) 237–260 (Verlag Beier & Beran, Langenweißbach, 2023).
120. Spazier, I., Queck, T. & Neubeck, V. Auf über 20 km Archäologie durch das Altenburger Land. in *Altenburger Geschichts- und Hauskalender 31* 55–60 (2021).
121. Bock, S. Zur Anthropologie der beiden frühmerowingerzeitlichen Skelettfunde aus der Gemarkung Zschernitzsch, Lkr. Altenburger Land. in *Neue archäologische Forschungen im Altenburger Land. Sonderveröffentlichung des Thüringischen Landesamtes für Denkmalpflege und Archäologie. Band 6* (ed. Spazier, I.) 261–265 (Verlag Beier & Beran, Langenweißbach, 2023).
122. Prammer, J. Neue Forschungen zum spätrömischen Straubing. Die Gräberfelder Azlburg I und Azlburg II. in *Vorträge des 5. Niederbayerischen Archäologentages (Deggendorf 1987)* (ed. Schmotz, K.) 133ff (1987).

123. Moosbauer, G. *Kastell Und Friedhöfe Der Spätantike in Straubing - Römer Und Germanen Auf Dem Weg Zu Den Ersten Bajuwaren. Passauer Universitätsschriften Zur Archäologie, Band 10*. (VML Verlag Marie Leidorf, Rahden, 2005).

124. Fischer, T. Römer und Germanen an der Donau. in *Die Bajuwaren. Von Severin bis Tassilo 488 - 788. Gemeinsame Landesausstellung des Freistaates Bayern und des Landes Salzburg; Rosenheim/Bayern, Mattsee/Salzburg; 19. Mai bis 6. November 1988* (ed. Dannheimer, H.) 39–45 (München, 1988).

125. Schweissing, M. M. & Grupe, G. Stable strontium isotopes in human teeth and bone: a key to migration events of the late Roman period in Bavaria. *J. Archaeol. Sci.* **30**, 1373–1383 (2003).

126. Rieder, K. H. Archäologischer Beitrag zur Siedlungsgeschichte der Region Ingolstadt von der späten Römerzeit bis ins frühe Mittelalter. *Sammelblatt des Historischen Vereins Ingolstadt 99* 9 – 76 (1990).

127. Keller, E. & Rieder, K. H. Eine germanische Kriegerbestattung des frühen 5. Jahrhunderts n. Chr. aus Kemathen, Markt Kipfenberg, Landkreis Eichstätt, Oberbayern. *Das archäologische Jahr in Bayern 1991* 132 – 137 (1992).

128. Fischer, T. Mutmaßliche und gesicherte germanische Kammergräber des 3. und 5. Jahrhunderts n. Chr. aus dem Vorland des ostraetischen Limes (Berching-Pollanten, Kemathen und Irfersdorf). Mit einem Beitrag von Jörg Lang. in *Kammergräber im Barbaricum – Zu Einflüssen und Übergangsphänomenen von der vorrömischen Eisenzeit bis in die Völkerwanderungszeit. Internationale Tagung, Schleswig, 25.–27. November 2010* (eds. Abegg-Wigg, A. & Lau, N.) 271 – 308 (Neumünster/Hamburg, 2014).

129. Golubović, S., Mrdjić, N. & Speal, C. S. Killed by the arrow: grave No. 152 from Viminacium. in *Waffen in Aktion. Akten der 16. Internationalen Roman Military Equipment Conference (ROMEC), Xanten, 13. – 16. Juni 2007* (eds. Busch, A. W. & Schalles, H.-J.) vol. 16 55–63 (Verlag Philipp von Zabern, Mainz, 2009).

130. Зотовић, Љ. Некропола из времена велике сеобе народа са уже градске територије Виминацијa. *Старинар XXXI* 95–114 (1980).

131. Zotović, L. Die gepidische Nekropole bei Viminacium. in *Simposium 75 Annes de foulles a Caričin Grad, Belgrade* (Belgrade, 1987).

132. Zotović, L. Die gepidische Nekropole von Viminacium. *Starinar XLIII/XLIV, Beograd* 183–190 (1994).

133. Ivanišević, V., Kazanski, M. & Mastykova, A. *Les Nécropoles de Viminacium à L'époque Des Grandes*

Migrations. vol. 22 (Collège de France - CNRS, Centre de recherche díhistoire et civilizations de Byzance, Paris, 2006).

134. Mikić, Ž. Erste Ergebnisse anthropologischer Untersuchung des Germanenfriedhofes von Viminacium. *Starinar XLIII-XLIV (1992-1993)* 191–199 (1994).
135. Микић, Ж. Антрополошки профил Гепида са Виминацијума. *Зборник Филозофског факултета XVIII/A* 61–68 (1994).
136. Микић, Ж. Антрополошки осврт на вештачки деформисане лобање из периода велике сеобе народа. *Зборник Народног музеја (Београд) XV/1* 133–138 (1994).
137. Mikić, Ž. Die Gepiden von Viminacium in der Völkerwanderungszeit. Anthropologischer Beitrag. *Anthropologischer Anzeiger 57/3* 257–268 (1999).
138. Микић, Ж. Некрополе из периода сеобе народа у Виминацијуму - антрополошка ревизија. *Архаика 1* 209–217 (2007).
139. Caduff, M., Eckel, R., Leuenberger, C. & Wegmann, D. Accurate Bayesian inference of sex chromosome karyotypes and sex-linked scaffolds from low-depth sequencing data. *Mol. Ecol. Resour.* **24**, e13913 (2024).
140. *Der heilige Nonnosus von Molzbichl. Das Kärntner Landesarchiv 27*. (Verlag des Kärntner Landesarchivs, Klagenfurt, 2001).
141. Glaser, F. Das Münster in Molzbichl, das älteste Kloster Kärntens. *Carinthia I* **179**, 99–124 (1989).
142. Karpf, K. Zwischen Frömmigkeit und Politik. Überlegungen zum Kloster Molzbichl, zu dessen Gründern und den vermeintlichen Spuren Aquileias in Oberkärnten im Frühmittelalter. *Carinthia I* **212**, 101–124 (2022).
143. Vetterling, C. KG Molzbichl, SG Spittal an der Drau. *Fundberichte aus Österreich (FÖ)* **52**, 187–188 (2013).
144. Vetterling, C. KG Molzbichl, SG Spittal an der Drau. *Fundberichte aus Österreich (FÖ)* **53**, 180–181 (2014).
145. Jungklaus, B. Der karantanische Kirchfriedhof von Molzbichl/Kärnten – Ergebnisse der anthropologischen Untersuchung. in *Die frühen Slawen – von der Expansion zu gentes und nationes* (eds. Biermann, F., Kersting, T. & Klammt, A.) vol. 81/2 113–122 (2016).

146. Alfieri, N. *La ricerca e la scoperta di Spina*. in *Spina, storia di una città tra Greci ed Etruschi, Catalogo della mostra a Ferrara, Castello Estense 26/09/1993-15/05/1994* (eds. Berti, F. & Guzzo, P. G.) 3–20 (Comitato Ferrara Arte, 1993).
147. Gaucci, A. Organizzazione degli spazi funerari a Spina e in area deltizia con particolare riguardo al periodo tardo-arcaico. in *La delimitazione dello spazio funerario in Italia dalla protostoria all'età arcaica. Recinti, circoli, tumuli (Atti del XXII Convegno Internazionale di Studi sulla Storia e l'Archeologia dell'Etruria, Orvieto 19-21 dicembre 2014)* (ed. Della Fina, G.) 113–170 (Roma, 2015).
148. Patitucci Uggeri, S. & Uggeri, G. La Topografia della citta. in *Spina – Storia Di una città tra greci ed Etruschi* (eds. Berti, F. & Guzzo, P. G.) (Comitato Ferrara Arte, Ferrara, 1993).
149. Torelli, M. Spina E La Sua Storia. in *Spina – Storia Di una città tra greci ed Etruschi* (eds. Berti, F. & Guzzo, P. G.) (Comitato Ferrara Arte, Ferrara, 1993).
150. Hostetter, E. Banqueting Bronzes at Spina: The Archaeological Context. *Etruscan Studies* **5**, 69–94 (1998).
151. Guzzo, P. G. Ipotesi di lavoro per un'analisi dell'ideologia funeraria. in *Spina. Storia di una città tra Greci ed Etruschi (Catalogo della Mostra, Ferrara, 26 settembre 1993-15 maggio 1994)* 219–229 (Comitato Ferrara Arte, Ferrara, 1993).
152. Masotti, S., Onisto, N., Marzi, M. & Gualdi-Russo, E. Dento-alveolar features and diet in an Etruscan population (6th-3rd c. B.C.) from northeast Italy. *Archives of Oral Biology* **58**, 416–426 (2013).
153. Manzon, V. S. & Gualdi-Russo, E. Health patterns of the Etruscan population (6th–3rd centuries BC) in Northern Italy: The case of Spina. *International Journal of Osteoarchaeology* **26**, 490–501 (2016).
154. Manzon, V. S., Rinaldo, N. & Gualdi-Russo, E. Skeletal trauma in an Iron Age context: new insight into the Etruscan population from Spina (Ferrara, Italy). *Archaeol. Anthropol. Sci.* **16**, (2024).
155. Masotti, S., Mongillo, J. & Gualdi-Russo, E. Burned human remains: diachronic analysis of cremation rituals in necropolises of northern Italy. *Archaeol. Anthropol. Sci.* **12**, (2020).
156. Manzon, V. S., Onisto, N. & Gualdi-Russo, E. Spondylolisthesis in an Etruscan woman from Spina (Ferrara, Italy): an Iron Age case report. *Collegium antropologicum* **38**, 745–748 (2014).
157. Gualdi-Russo, E., Zedda, N., Esposito, V. & Masotti, S. More on molar incisor hypomineralisation (MIH) and linear enamel hypoplasia (LEH) in archaeological human remains. *Clin. Oral Investig.* **21**,

2153–2154 (2017).

158. Gualdi-Russo, E., Manzon, V. S., Saguto, I. & Rinaldo, N. Life and death in Italian prehistory: The case of the sailor from Spina. *World Neurosurg.* **139**, 106–110 (2020).

159. Manzon, V. S., Ferrante, Z., Giganti, M. & Gualdi-Russo, E. On the antiquity of Legg–Calvé–Perthes disease: Skeletal evidence in Iron Age Italy. *Homo* **68**, 10–17 (2017).

160. McGlynn, G. C. Hidden Graves and Commingled Human Remains. An anthropological examination of a Roman chamber tomb at Doliche, Turkey. in *Facetten der Osteologie* (eds. Francken, M. & Harvati, K.) 116–139 (Tübingen University Press, Tübingen, Germany, 2023).

161. Bentley, R. A. Strontium Isotopes from the Earth to the Archaeological Skeleton: A Review. *J. Archaeol. Method Theory* **13**, 135–187 (2006).

162. Toncala, A. *et al.* On the premises of mixing models to define local bioavailable 87Sr/86Sr ranges in archaeological contexts. *Sci. Total Environ.* **745**, (2020).

163. Frei, R., Frank, A. B. & Frei, K. M. The proper choice of proxies for relevant strontium isotope baselines used for provenance and mobility studies in glaciated terranes – Important messages from Denmark. *Sci Total Environ* **821**, (2022).

164. Lugli, F. *et al.* A strontium isoscape of Italy for provenance studies. *Chem. Geol.* **587**, (2022).

165. Nanci, A. *Ten Cate's Oral Histology: Development, Structure, and Function*. (Mosby, St. Louis, MO, 2003).

166. Raggatt, L. J. & Partridge, N. C. Cellular and molecular mechanisms of bone remodeling. *J. Biol. Chem.* **285**, 25103–25108 (2010).

167. Steiger, R. H. & Jäger, E. Subcommission on geochronology: Convention on the use of decay constants in geo- and cosmochronology. *Earth Planet. Sci. Lett.* **36**, 359–362 (1977).

168. Faure, G. & Mensing, T. M. *Isotopes: Principles and Applications. 3rd Edition*. (John Wiley & Sons, Inc, 2009).

169. Thirlwall, M. F. Long-term reproducibility of multicollector Sr and Nd isotope ratio analysis. *Chem. Geol.* **94**, 85–104 (1991).

170. Grupe, G. *et al.* Mobility of Bell Beaker people revealed by strontium isotope ratios of tooth and bone: a study of southern Bavarian skeletal remains. *Appl. Geochem.* **12**, 517–525 (1997).

171. Bentley, R. A. & Knipper, C. Geographical patterns in biologically available strontium, carbon and oxygen isotope signatures in prehistoric SW Germany. *Archaeometry* **47**, 629–644 (2005).

172. Alt, K. W. *et al.* Identifizierung von Einheimischen und Ortsfremden der Mehrfachbestattung Grab 244 aus Ergolding anhand standorttypischer Strontiumisotopie. in *Frühmittelalterliche Adelsgräber aus Ergolding.* (ed. Koch, H.) 70–73 (Dr. Faustus, Büchenbach, 2014).

173. Gerhard Doppler, Markus Fiebig, Walter Freudenberger, Stefan Glaser, Rolf Meyer, Thomas Pürner, Johann Rohrmüller, Klaus Schwerd. *GeoBavaria. 600 Millionen Jahre Bayern. Internationale Edition*. (Bayerisches Geologisches Landesamt, 2004).

174. Massy, K., Friedrich, R., Mittnik, A. & Stockhammer, P. W. Pedigree-based Bayesian modelling of radiocarbon dates. *PLoS One* **17**, e0270374 (2022).

175. Sedig, J. W., Olade, I., Patterson, N., Harney, É. & Reich, D. Combining ancient DNA and radiocarbon dating data to increase chronological accuracy. *J. Archaeol. Sci.* **133**, 105452 (2021).

176. Hedges, R. E. M., Clement, J. G., Thomas, C. D. L. & O'Connell, T. C. Collagen turnover in the adult femoral mid-shaft: Modeled from anthropogenic radiocarbon tracer measurements. *Am. J. Phys. Anthropol.* **133**, 808–816 (2007).

177. Ubelaker, D. H., Buchholz, B. A. & Stewart, J. E. B. Analysis of artificial radiocarbon in different skeletal and dental tissue types to evaluate date of death. *J. Forensic Sci.* **51**, 484–488 (2006).

178. Jørkov, M. L. S., Heinemeier, J. & Lynnerup, N. The petrous bone - A new sampling site for identifying early dietary patterns in stable isotopic studies. *Am. J. Phys. Anthropol.* **138**, 199–209 (2009).

179. Manolagas, S. C. Birth and Death of Bone Cells: Basic Regulatory Mechanisms and Implications for the Pathogenesis and Treatment of Osteoporosis. *Endocr. Rev.* **21**, 115–137 (2000).

180. Bronk Ramsey, C. Bayesian Analysis of Radiocarbon Dates. *Radiocarbon* **51**, 337–360 (2009).

181. Reimer, P. J. *et al.* The IntCal20 Northern Hemisphere Radiocarbon Age Calibration Curve (0–55 cal kBP). *Radiocarbon* **62**, 725–757 (2020).

182. Wiesehöfer, J. Heiratsalter. in *Der Neue Pauly: Enzyklopädie der Antike. Altertum. Band 5* (eds. Cancik, H. & Schneider, H.) 256–258 (Verlag J.B. Metzler, Stuttgart, 1998).

183. Bagnall, R. S. & Frier, B. W. *The Demography of Roman Egypt*. (Cambridge University Press, Cambridge, 1994).

184. Schmitz, W. 'Quiescit in pace': Die Abkehr des Toten von der Welt der Lebenden. Epigraphische Zeugnisse der Spätantike als Quellen der historischen Familienforschung. in *Kontinuität und Diskontinuität: Germania inferior am Beginn und am Ende der römischen Herrschaft. Beiträge des deutsch-niederländischen Kolloquiums in der Katholieke Universiteit Nijmegen (27. bis 30.06. 2001)* (eds. Grünewald, T. & Seibel, S.) 374–413 (De Gruyter, Berlin, Germany, 2012).
185. Hopkins, M. K. The age of Roman girls at marriage. *Population Studies* **18**, 309–327 (1965).
186. Shaw, B. D. The age of Roman Girls at Marriage: Some Reconsiderations. *J. Rom. Stud.* **77**, 28–46 (1987).
187. Frier, B. W. Natural fertility and family limitation in Roman marriage. *Class. Philol.* **89**, 318–333 (1994).
188. Hack, A. T. *Alter, Krankheit, Tod und Herrschaft im frühen Mittelalter. Das Beispiel der Karolinger*. (Anton Hiersemann Verlag, Stuttgart, 2009).
189. Rees, M. The age of menarche. *ORGYN* 2–4 (1995).
190. Lehmann, A., Scheffler, C. & Hermanussen, M. The variation in age at menarche: an indicator of historic developmental tempo. *Anthropol. Anz.* **68**, 85–99 (2010).
191. Kajava, Y. Die anthropologische Untersuchung des finnischen Volkes. *Anthropol. Anz.* **2**, 228–253 (1925).
192. Malmio, H. R. Über das Alter der Menarche in Finnland: eine statistische Studie. (1919).
193. Demoulin, F. Secular trend in France. in *Secular growth changes in Europe* (eds. Bodzsar, B. E. & Susanne, C.) 109–134 (Eötvös University Press, Budapest, 1998).
194. Westhoff, C. Ueber die Zeit des Eintritts der Menstruation, nach Angabe von 3000 Schwangeren in der Königl. Universitäts-Entbindungs-Anstalt zu Marburg. (Universität Marburg, Marburg, 1873).
195. Fraunhoffer, A. Über die Vorverlagerung der Menarche im ersten Drittel des 20. Jahrhunderts aufgrund statistischer Erhebungen aus der Tübinger Universitäts-Frauenklinik. (Universität Tübingen, Tübingen, 1947).
196. Hecker, K. Cited according to hand-written material, legacy W. Lenz. Unpublished. (1864).
197. Lillies, F. Dissertation, Königsberg. Cited according to hand-written material, legacy W. Lenz. Unpublished. (1886).

198. Žegarac, A. *et al.* Ancient genomes provide insights into family structure and the heredity of social status in the early Bronze Age of southeastern Europe. *Sci. Rep.* **11**, 10072 (2021).

199. Boessenkool, S. *et al.* Combining bleach and mild predigestion improves ancient DNA recovery from bones. *Mol. Ecol. Resour.* **17**, 742–751 (2017).

200. Marchi, N. *et al.* The genomic origins of the world's first farmers. *Cell* **185**, 1842–1859.e18 (2022).

201. Bolger, A. M., Lohse, M. & Usadel, B. Trimmomatic: a flexible trimmer for Illumina sequence data. *Bioinformatics* **30**, 2114–2120 (2014).

202. Li, H. & Durbin, R. Fast and accurate short read alignment with Burrows-Wheeler transform. *Bioinformatics* **25**, 1754–1760 (2009).

203. Li, H. *et al.* The Sequence Alignment/Map format and SAMtools. *Bioinformatics* **25**, 2078–2079 (2009).

204. Kousathanas, A. *et al.* Inferring heterozygosity from ancient and low coverage genomes. *Genetics* **205**, 317–332 (2017).

205. Danecek, P. *et al.* Twelve years of SAMtools and BCFtools. *Gigascience* **10**, (2021).

206. Fu, Q. *et al.* A revised timescale for human evolution based on ancient mitochondrial genomes. *Curr. Biol.* **23**, 553–559 (2013).

207. Korneliussen, T. S., Albrechtsen, A. & Nielsen, R. ANGSD: Analysis of Next Generation Sequencing Data. *BMC Bioinformatics* **15**, 356 (2014).

208. Lazaridis, I. *et al.* Ancient human genomes suggest three ancestral populations for present-day Europeans. *Nature* **513**, 409–413 (2014).

209. Gretzinger, J. *et al.* Evidence for dynastic succession among early Celtic elites in Central Europe. *Nat. Hum. Behav.* **8**, 1467–1480 (2024).

210. Olalde, I. *et al.* The genomic history of the Iberian Peninsula over the past 8000 years. *Science* **363**, 1230–1234 (2019).

211. Bagnasco, G. *et al.* Bioarchaeology aids the cultural understanding of six characters in search of their agency (Tarquinia, ninth-seventh century BC, central Italy). *Sci. Rep.* **14**, 11895 (2024).

212. Margaryan, A. *et al.* Population genomics of the Viking world. *Nature* **585**, 390–396 (2020).

213. Lazaridis, I. *et al.* The genetic history of the Southern Arc: A bridge between West Asia and Europe.

Science **377**, eabm4247 (2022).

214. Harney, É. *et al.* A minimally destructive protocol for DNA extraction from ancient teeth. *Genome Res.* **31**, 472–483 (2021).

215. Ning, C. *et al.* Ancient genomes reveal Yamnaya-related ancestry and a potential source of Indo-European speakers in iron age Tianshan. *Curr. Biol.* **29**, 2526–2532.e4 (2019).

216. Damgaard, P. de B. *et al.* 137 ancient human genomes from across the Eurasian steppes. *Nature* **557**, 369–374 (2018).

217. Saag, L. *et al.* The arrival of Siberian ancestry connecting the Eastern Baltic to Uralic speakers further east. *Curr. Biol.* **29**, 1701–1711.e16 (2019).

218. Stolarek, I. *et al.* Genetic history of East-Central Europe in the first millennium CE. *Genome Biol.* **24**, 173 (2023).

219. Purcell, S. *et al.* PLINK: a tool set for whole-genome association and population-based linkage analyses. *Am. J. Hum. Genet.* **81**, 559–575 (2007).

220. Patterson, N. *et al.* Ancient admixture in human history. *Genetics* **192**, 1065–1093 (2012).

221. Alt, K. W. *et al.* Lombards on the move-an integrative study of the migration period cemetery at Szólád, Hungary. *PLoS One* **9**, e110793 (2014).

222. Hagberg, A. A., Schult, D. A. & Swart, P. J. Exploring network structure, dynamics, and function using NetworkX. in *Proceedings of the 7th Python in Science Conference (SciPy2008)* (eds. Varoquaux, G., Vaught, T. & Millman, J.) 11–15 (Pasadena, CA USA, 2008).

223. Korunes, K. L. & Samuk, K. pixy: Unbiased estimation of nucleotide diversity and divergence in the presence of missing data. *Mol. Ecol. Resour.* **21**, 1359–1368 (2021).
