# Supplementary material for: Demography and life histories across the Roman frontier in Germany 400–700 ce
Source: Nature. 2026 Apr 29;654(8120):984–93. doi: 10.1038/s41586-026-10437-3 (PMC13293882; doi:10.1038/s41586-026-10437-3)
Supplement: Supplementary file 2 — Reporting Summary [file 41586_2026_10437_MOESM2_ESM.pdf]

nature portfolio

Corresponding author(s): Joachim Burger, Steffen Patzold; Daniel Wegmann

Last updated by author(s): 13.03.26

Reporting Summary

Nature Portfolio wishes to improve the reproducibility of the work that we publish. This form provides structure for consistency and transparency in reporting. For further information on Nature Portfolio policies, see our Editorial Policies and the Editorial Policy Checklist.

Statistics

For all statistical analyses, confirm that the following items are present in the figure legend, table legend, main text, or Methods section.

n/a	Confirmed	
☐	☒	The exact sample size (*n*) for each experimental group/condition, given as a discrete number and unit of measurement
☐	☒	A statement on whether measurements were taken from distinct samples or whether the same sample was measured repeatedly
☐	☒	The statistical test(s) used AND whether they are one- or two-sided<br>*Only common tests should be described solely by name; describe more complex techniques in the Methods section.*
☒	☐	A description of all covariates tested
☒	☐	A description of any assumptions or corrections, such as tests of normality and adjustment for multiple comparisons
☐	☒	A full description of the statistical parameters including central tendency (e.g. means) or other basic estimates (e.g. regression coefficient) AND variation (e.g. standard deviation) or associated estimates of uncertainty (e.g. confidence intervals)
☒	☐	For null hypothesis testing, the test statistic (e.g. *F*, *t*, *r*) with confidence intervals, effect sizes, degrees of freedom and *P* value noted<br>*Give P values as exact values whenever suitable.*
☐	☒	For Bayesian analysis, information on the choice of priors and Markov chain Monte Carlo settings
☒	☐	For hierarchical and complex designs, identification of the appropriate level for tests and full reporting of outcomes
☒	☐	Estimates of effect sizes (e.g. Cohen's *d*, Pearson's *r*), indicating how they were calculated

Our web collection on statistics for biologists contains articles on many of the points above.

Software and code

Policy information about availability of computer code

Data collection	Newly reported data was generated on the Illumina NovaSeq 6000 and co-analysed alongside publicly available sequence data.
Data analysis	trimmomatic 0.36<br>bwa 0.7.17-r1188<br>SAMtools 1.13<br>sambamba 0.6.8<br>GATK 3.8<br>contamMix 1.0.9<br>ATLAS commit [647daf792df01c4aa8e686de0e462ddd8f91e6c6]<br>glimpse2<br>HaploGrep3<br>Yleaf2<br>BCFtools 1.13<br>plink 1.9<br>EIGENSOFT (smartpca) v8.0.0<br>ADMIXTOOLS 2.0.8<br>filia commit [2fa4117fc22597a106769e8d12758f7c33c51f75]<br>Chronograph commit<br>[affa83ffb04c6083d6fdf71a506b09ac0300fe74]<br>scikit-allele v1.3.1<br>statsmodels v.0.14.4

SLiM 4
pixy 1.2.7
hapROH 0.64
ancIBD 0.5
twigstats 1.0.2
Relate 1.2.2
finestructure4
ChromoPainter2
SourceFind2
PANE 0.0.1
Additional code used in the analyses: https://doi.org/10.5281/zenodo.17192653

For manuscripts utilizing custom algorithms or software that are central to the research but not yet described in published literature, software must be made available to editors and reviewers. We strongly encourage code deposition in a community repository (e.g. GitHub). See the Nature Portfolio guidelines for submitting code & software for further information.

Data

Policy information about availability of data

All manuscripts must include a data availability statement. This statement should provide the following information, where applicable:

- Accession codes, unique identifiers, or web links for publicly available datasets
- A description of any restrictions on data availability
- For clinical datasets or third party data, please ensure that the statement adheres to our policy

Raw and aligned sequence reads are available at the European Nucleotide Archive under accession number PRJEB87112. The human reference genome (hg19) used during alignment is available via the 1000 genomes project repository (https://ftp.1000genomes.ebi.ac.uk/vol1/ftp/technical/reference/phase2_reference_assembly_sequence/). Genome sequences for the 379 modern day individuals from Germany are available upon request at the DZHK (https://dzhk.de/en/dzhk-heart-bank/data-and-biospecimens/dzhkomics-resource). The 1000GP phase 3 reference panel used for imputation and Relate can be downloaded from https://ftp.1000genomes.ebi.ac.uk/vol1/ftp/release/20130502/. Previously published genotype data for present-day and ancient individuals is available through the Allen Ancient DNA Resource at the Harvard dataverse (https://dataverse.harvard.edu/dataset.xhtml?persistentId=doi:10.7910/DVN/FFIDCW).

Research involving human participants, their data, or biological material

Policy information about studies with human participants or human data. See also policy information about sex, gender (identity/presentation), and sexual orientation and race, ethnicity and racism.

Reporting on sex and gender	Assignments of male/female were based on the distribution of reads aligned to the sex chromosomes. Genomes were grouped based on these assignments to draw conclusions about residence, marriage and kinship practices.
Reporting on race, ethnicity, or other socially relevant groupings	We did not categorize individuals by socially constructed categories such as ethnicity or race. Samples were grouped by geography, dating as well as funerary context. We furthermore used genomic ancestry and affinities to other geographic and temporally defined groups of individuals to assess demographic processes at local scales.
Population characteristics	Osteological assessments of age-at-death, pathologies and potential trauma was carried out for some of the individuals in this study. We determined individual ancestry, shared genomic segments and affinities to other spatiotemporaly defined groups.
Recruitment	NA
Ethics oversight	NA

Note that full information on the approval of the study protocol must also be provided in the manuscript.

Field-specific reporting

Please select the one below that is the best fit for your research. If you are not sure, read the appropriate sections before making your selection.

☐ Life sciences ☐ Behavioural & social sciences ☒ Ecological, evolutionary & environmental sciences

For a reference copy of the document with all sections, see nature.com/documents/nr-reporting-summary-flat.pdf

Life sciences study design

All studies must disclose on these points even when the disclosure is negative.

Sample size	NA
Data exclusions	NA
Replication	NA

Randomization	NA
Blinding	NA

Behavioural & social sciences study design

All studies must disclose on these points even when the disclosure is negative.

Study description	NA
Research sample	NA
Sampling strategy	NA
Data collection	NA
Timing	NA
Data exclusions	NA
Non-participation	NA
Randomization	NA

Ecological, evolutionary & environmental sciences study design

All studies must disclose on these points even when the disclosure is negative.

Study description	We generated 258 genomes with a main focus on early medieval row-gravefields from southern Germany, with the intend to characterize the populations that started these customs and their social and kinship practices. Additionally we generated data from key sites dating to late Antiquity and the Iron Age for contextualization.
Research sample	Sequence data generated from human remains from different archaeological contexts
Sampling strategy	For the four main sites we intended to generate a sample large enough to be able to capture temporal trends and identify potential kinship systems. Additional individuals from archaeological sites in the same regions were chosen for contextualization.
Data collection	The majority of newly reported data (239) was generated from individuals found during archaeological excavations in southern Germany, with additional data from individuals from northern Italy (2), Austria (3), Romania (3) Serbia (10) and Turkey(1). Bone samples were collected and transferred to the clean-room facilities of the Palaeogenetics Group of the JGU Mainz for further processing.
Timing and spatial scale	NA
Data exclusions	We created a subset of the data that excluded close-kin pairs for population genetic analyses. Data was excluded from certain analyses based on ancestry, geographical or temporal range. Specific data used and rationale for exclusion are given in the respective sections of the manuscript and the Supplementary Information.
Reproducibility	All data will be published alongside this study to ensure reproducibility
Randomization	NA
Blinding	NA

Did the study involve field work? ☐ Yes ☒ No

Field work, collection and transport

Field conditions	NA
Location	NA
Access & import/export	NA

Disturbance	NA

Reporting for specific materials, systems and methods

We require information from authors about some types of materials, experimental systems and methods used in many studies. Here, indicate whether each material, system or method listed is relevant to your study. If you are not sure if a list item applies to your research, read the appropriate section before selecting a response.

Materials & experimental systems

n/a	Involved in the study
☒	☐ Antibodies
☒	☐ Eukaryotic cell lines
☐	☒ Palaeontology and archaeology
☒	☐ Animals and other organisms
☒	☐ Clinical data
☒	☐ Dual use research of concern
☒	☐ Plants

Methods

n/a	Involved in the study
☒	☐ ChIP-seq
☒	☐ Flow cytometry
☒	☐ MRI-based neuroimaging

Antibodies

Antibodies used	NA
Validation	NA

Eukaryotic cell lines

Policy information about cell lines and Sex and Gender in Research

Cell line source(s)	NA
Authentication	NA
Mycoplasma contamination	NA
Commonly misidentified lines (See ICLAC register)	NA

Palaeontology and Archaeology

Specimen provenance	All samples were provided by co-authors/collaborators of this study who held the necessary permissions to sample those specimen for the analyses reported here.
Specimen deposition	Bone residuals from DNA sampling are stored in the clean room facilities of the Palaeogenetics Group, JGU Mainz
Dating methods	New radiocarbon dates were generated at the Curt-Engelhorn-Center for Archaeometry, Mannheim, Germany, the AMS laboratory Erlangen, Friedrich-Alexander Universität Erlangen-Nürnberg, Germany and Mass Spectrometry Laboratory, Center for Physical Sciences and Technology, in Vilnius, Lithuania. Laboratory protocols and further details can be found at each laboratories website.

☒ Tick this box to confirm that the raw and calibrated dates are available in the paper or in Supplementary Information.

Ethics oversight	All genomes published alongside this study originate from archaeological contexts and have no identifiable relationships to living persons.

Note that full information on the approval of the study protocol must also be provided in the manuscript.

Animals and other research organisms

Policy information about studies involving animals; ARRIVE guidelines recommended for reporting animal research, and Sex and Gender in Research

Laboratory animals	NA
Wild animals	NA

Reporting on sex	NA
Field-collected samples	NA
Ethics oversight	NA

Note that full information on the approval of the study protocol must also be provided in the manuscript.

Clinical data

Policy information about clinical studies

All manuscripts should comply with the ICMJE guidelines for publication of clinical research and a completed CONSORT checklist must be included with all submissions.

Clinical trial registration	NA
Study protocol	NA
Data collection	NA
Outcomes	NA

Dual use research of concern

Policy information about dual use research of concern

Hazards

Could the accidental, deliberate or reckless misuse of agents or technologies generated in the work, or the application of information presented in the manuscript, pose a threat to:

No	Yes	
☒	☐	Public health
☒	☐	National security
☒	☐	Crops and/or livestock
☒	☐	Ecosystems
☒	☐	Any other significant area

Experiments of concern

Does the work involve any of these experiments of concern:

No	Yes	
☒	☐	Demonstrate how to render a vaccine ineffective
☒	☐	Confer resistance to therapeutically useful antibiotics or antiviral agents
☒	☐	Enhance the virulence of a pathogen or render a nonpathogen virulent
☒	☐	Increase transmissibility of a pathogen
☒	☐	Alter the host range of a pathogen
☒	☐	Enable evasion of diagnostic/detection modalities
☒	☐	Enable the weaponization of a biological agent or toxin
☒	☐	Any other potentially harmful combination of experiments and agents

Plants

Seed stocks: NA

Novel plant genotypes: NA

Authentication: NA

ChIP-seq

Data deposition

- [ ] Confirm that both raw and final processed data have been deposited in a public database such as GEO.
- [ ] Confirm that you have deposited or provided access to graph files (e.g. BED files) for the called peaks.

Data access links
May remain private before publication.: NA

Files in database submission: NA

Genome browser session (e.g. UCSC): NA

Methodology

Replicates: NA

Sequencing depth: NA

Antibodies: NA

Peak calling parameters: NA

Data quality: NA

Software: NA

Flow Cytometry

Plots

Confirm that:

- [ ] The axis labels state the marker and fluorochrome used (e.g. CD4-FITC).
- [ ] The axis scales are clearly visible. Include numbers along axes only for bottom left plot of group (a 'group' is an analysis of identical markers).
- [ ] All plots are contour plots with outliers or pseudocolor plots.
- [ ] A numerical value for number of cells or percentage (with statistics) is provided.

Methodology

Sample preparation: NA

Instrument: NA

Software: NA

Cell population abundance: NA

Gating strategy	NA

☐ Tick this box to confirm that a figure exemplifying the gating strategy is provided in the Supplementary Information.

Magnetic resonance imaging

Experimental design

Design type	NA
Design specifications	NA
Behavioral performance measures	NA

Acquisition

Imaging type(s)	NA
Field strength	NA
Sequence & imaging parameters	NA
Area of acquisition	NA

Diffusion MRI ☐ Used ☐ Not used

Preprocessing

Preprocessing software	NA
Normalization	NA
Normalization template	NA
Noise and artifact removal	NA
Volume censoring	NA

Statistical modeling & inference

Model type and settings	NA
Effect(s) tested	NA

Specify type of analysis: ☐ Whole brain ☐ ROI-based ☐ Both

Statistic type for inference (See Eklund et al. 2016)	NA
Correction	NA

Models & analysis

n/a	Involved in the study
☐	☐ Functional and/or effective connectivity
☐	☐ Graph analysis
☐	☐ Multivariate modeling or predictive analysis

Functional and/or effective connectivity	NA
Graph analysis	NA
Multivariate modeling and predictive analysis	NA
