# Supplementary material for: Demography and life histories across the Roman frontier in Germany 400–700 ce
Source: Nature. 2026 Apr 29;654(8120):984–93. doi: 10.1038/s41586-026-10437-3 (PMC13293882; doi:10.1038/s41586-026-10437-3)
Supplement: Supplementary file 4 — This file provides a key to the tabs in the Supplementary Table 2 Excel spreadsheet. Individual-level genomic profiles—ancestry, quality metrics, and analytical outputs across methodologies. [file 41586_2026_10437_MOESM4_ESM.zip › Supplementary_Table_2_key.pdf]

Supplementary Table 2:

1.Modern Reference (PCA)

Modern reference individuals used in the projection PCA in Fig. 2 and throughout the PCAs in SI chapters.

2.Iron Age Reference (PCA)

Iron age individuals used in the projection PCA in Fig. 2 and throughout the PCAs in SI chapters.

3.Chronograph Results

Individual mean posterior birth and death dates estimated with chronograph

4.twigstats sources

Sources resulting from the genetic/geographic clustering approach based on the pairwise twigstats-qpAdm models

5.twigstats results (distal s.)

Individual level ancestry estimates from Relate/twigstats based paintings using distal sources

6.twigstats results (local s.)

Individual level ancestry estimates from Relate/twigstats based paintings using local sources

7.Chromopainter sources

Sources resulting from the genetic/geographic clustering approach based on the Chromopainter copying-vectors.

8.Chromopainter results (incl. 1240K)

Individual level ancestry estimates from ChromoPainter/Sourcefind2 analyses, also using 1240K data.

9.Chromopainter results (relate)

Individual level ancestry estimates from ChromoPainter/Sourcefind2 analyses, using the same (distal) sources and data as in the relate/twigstats analyses.

10.PANE results

Individual level ancestry estimates from PANE run used to produce SI Fig. S8.21 - S8.23

11.f4

Individual F4 values used in the *filia* analyses

12.f4&GraveGoods

Analysis of the grave goods of the individuals with the most extreme f4 values

13.hapROH

Results of hapROH analyses, reporting runs of homozygosity for each individual sequenced for this study with a high enough sequencing depth.

14.KIN

KIN results for Early Medieval sites for which enough genomes were produced.

15.ReadV2

Results of Read2 analysis of the whole data set

16.SeqInfo

Information on sequencing results for each individual produced for this study on per library level. Additional information on PMD, genetic sexing, contamination estimates (MT for all + X for males), uniparental markers, and individual diversity (theta) are given.
